# Tuning the steric hindrance of alkylamines: a predictive model of steric editing of planar amines†

**DOI:** 10.1039/d4sc03873h

**Published:** 2024-07-11

**Authors:** Michele Tomasini, Maria Voccia, Lucia Caporaso, Michal Szostak, Albert Poater

**Affiliations:** a Institut de Química Computacional i Catàlisi, Departament de Química, Universitat de Girona c/M^a^ Aurèlia Capmany 69 17003 Girona Catalonia Spain albert.poater@udg.edu; b Dipartimento di Chimica e Biologia, Università di Salerno Via Ponte don Melillo 84084 Fisciano Italy; c Department of Chemistry, Rutgers University 73 Warren Street Newark New Jersey 07102 USA

## Abstract

Amines are one of the most prevalent functional groups in chemistry. Perhaps even more importantly, amines represent one of the most ubiquitous moieties within the realm of bioactive natural products and life-saving pharmaceuticals. The archetypal geometrical property of amines is their sp^3^ hybridization with the lone pair of nitrogen occupying the apex of the pyramid. Herein, we present a blueprint for quantifying the properties of extremely sterically hindered alkylamines. These amines reach planarity around the nitrogen atom due to the excessive steric hindrance, which results in a conformational re-modeling of the amine moiety. Crucially, the steric properties of amines are characterized by the %*V*_Bur_ index, which we show is a general predictive parameter for evaluating the properties of sterically hindered amines. Computational studies on the acidic nature and the reactivity of organometallic Au and Pd complexes are outlined. Density functional theory calculations permit for predictive catalysis, ordering the mapping of extremely hindered tertiary amines by employing artificial intelligence *via* machine learning. Overall, the study outlines the correlation between the unusual geometry and the key thermodynamic and kinetic properties of extremely hindered alkylamines. The steric hindrance, as quantified by %*V*_Bur_, is the crucial factor influencing the observed trends and the space required to accommodate sterically hindered tertiary amines.

## Abstract 2

Amines are one of the most prevalent functional groups in chemistry.

## Graphical abstract

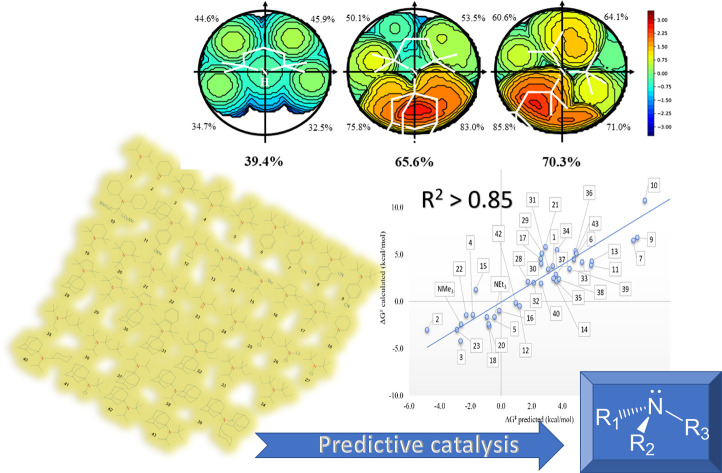

## 1. Introduction

**Scheme 1:**
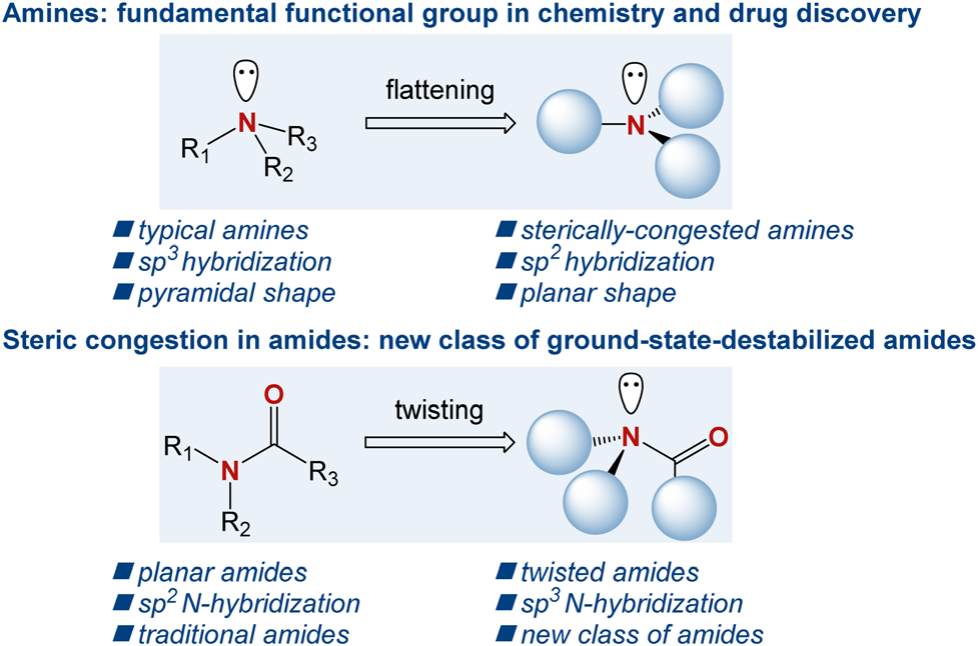
The effect of steric hindrance in amines, and sterically destabilized twisted amides.

The amine motif is one of the most fundamental functional groups within the realm of organic chemistry and drug discovery.^1^ In organic synthesis, in addition to being a fundamental part of the target products ranging from bioactive natural products to organometallic chemistry applications, amines serve as key bases and nucleophiles, where the accessibility of the free electron pair at the nitrogen atom enables their common role in synthetic reactions on a daily basis.^2^ In drug discovery, >80% of small-molecule drugs approved by the FDA contain an amino group, where the nitrogen atom serves as a key pharmacophore in drug–receptor interactions.^3^ Consequently, over the years, numerous generations of chemists have devised a wide array of synthetic methods to access the amine motif.^4^ In this context, sterically hindered amines, where the nitrogen atom significantly deviates from the sp^3^ pyramidalization, are of major synthetic and theoretical interest (Scheme 1).^5^ In a broader sense, sterically hindered amines are conceptually related to sterically hindered amides, where the typical planarity of the amide bond is geometrically transformed into a non-planar ground-state conformation by N–C(O) bond rotation and nitrogen pyramidalization.^6^ Recent years have witnessed a surge in applications of such twisted amides owing to their significantly different properties compared to their planar counterparts.^7^

**Scheme 2:**
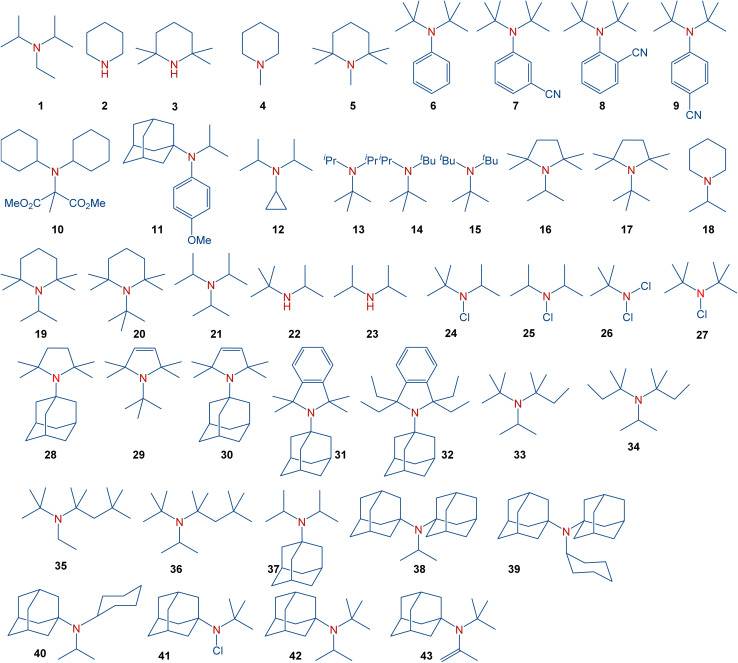
Full scope of amines in the present study.

In general, sterically hindered amines have already found diverse applications. Amines discussed in the present manuscript are summarized in Scheme 2. These amines include simple aliphatic amines, such as Hünig's base (1), sterically hindered piperidines (4–5), sterically hindered anilines (6–9), cyclopropylamines (12), aliphatic amines with gradually increasing steric hindrance (13–15), sterically hindered pyrrolidines (16–17), representative dialkylamines (18–20), formylamines (25), chloroamines (22–23), as well as amines with the most steric hindrance prepared or proposed to date (35–42). These species serve, for instance, as bases with low nucleophilicity (1 (ref. 8) and 2 (ref. 9)), precursors for persistent nitroxyl radicals (3 (ref. 10 and 11)), inhibitors in polymerization, and stabilizers (4).^12^ Moreover, sterically hindered amines have found applications in gas-treating processes,^13^ and as pharmacological agents.^14^

It should be noted that several tertiary amines with a notably high degree of steric congestion, *e.g.*, 7–9,^15^10,^16^11,^17^ encompass additional functional groups that significantly influence the molecular structure surrounding the amine nitrogen atom and, consequently, its reactivity. For example, amine 12 has been claimed to be the most hindered tertiary amine known.^18^ However, this amine derives its properties from the unique electronics and sterics of the cyclopropyl group. For years, triisopropylamine 21 appeared to set the limit for the achievable level of steric congestion in simple trialkylamines.^16^ This amine was predicted to exhibit nearly planar geometry around the nitrogen atom based on electron diffraction^19^ and NMR studies,^20^ while low-temperature crystallographic analysis^21^ indicated a flattened pyramid shape instead of a perfect planarity. Furthermore, despite claims in organic chemistry textbooks comparing bulky amines to alcohols,^22^ sterically hindered amines, such as 15 (ref. 23) typically avoid planar structures, while steric congestion in bulky amines should take into account the different bond lengths involved. Specifically, the average C–N bond is considerably shorter than C–C bonds,^24^ leading to a shortening of the distance between the groups around the nitrogen atom in sterically hindered amines. Synthetically, the stability of hindered amines should also be considered since amines with significant steric hindrance may undergo β-elimination.^25^ A recent study demonstrated the synthesis of some of the most sterically hindered amines reported to date, including 13–14, 16–18, 30–42 and 44.^26^ Among the strategies used,^27^ synthetic methods to access such sterically hindered amines include ammonium salts,^28^ alkyl^29,30^ or aryl^31^ Grignard reagents,^32^ Bruylants reaction,^19,33^ or the S_N_1 alkylation.^34^ These approaches yielded trialkylamines with exceptionally high steric hindrance, leading to restricted rotation around the C–N bond.^26^

Given the fundamental importance of extremely sterically hindered amines in various areas of chemistry and the critical role of geometry around the nitrogen atom in amines in drug discovery, herein we report a blueprint to quantify the properties of extremely sterically hindered alkylamines and correlate them with the geometry ranging from low^35^ to furthermost^36^ steric hindrance, leading to predictive catalysis.^37,38^

## 2. Computational details

DFT calculations were performed with the Gaussian16 set of programs,^39^ using the hybrid GGA functional of Becke-Lee, Parr, and Yang, *i.e.*, B3LYP,^40^ and the def2TZVP basis set for all atoms applied,^41^ except for Au and Pt, which were treated with the quasi-relativistic Stuttgart/Dresden effective core potential with an associated valence basis set (standard SDD keywords in Gaussian16).^42^ Moreover, we also included the D3 Grimme pairwise scheme to account for dispersion corrections in the geometry optimizations. Geometry optimizations were performed without symmetry constraints, and the characterization of the stationary points was performed by analytical frequency calculations. These frequencies were used to calculate unscaled zero-point energies (ZPEs) as well as thermal corrections and entropy effects at 298.15 K and 1 atm by using the standard statistical mechanics relationships for an ideal gas. We also included the solvent effects of THF solution estimated with the polarizable continuous solvation model (PCM) as implemented in Gaussian16.^43^

## 3. Results and discussion

**Fig. 1:**
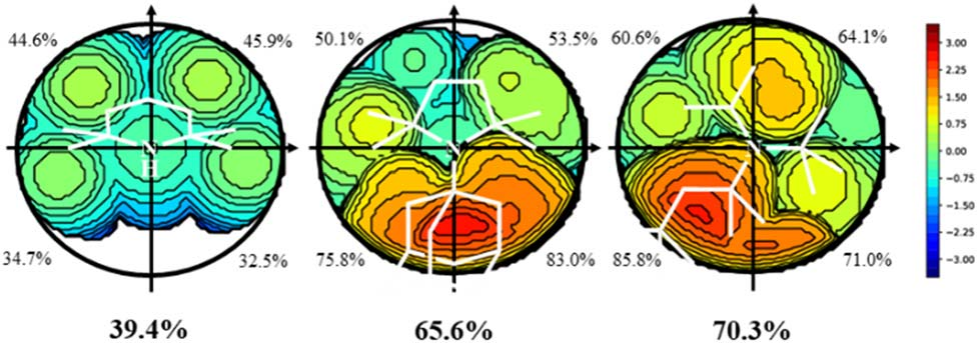
Topographic steric maps (*xy* plane) and %*V*_Bur_ of the amines 3 (left), 28 (middle), and 36 (right). The center is on the *Z* axis, where the Au–N bond would be, specifically at 2.0 Å from N, and the *XY* plane contains the plane that best defines the 3 N-coordinated atoms. The isocontour curves of the steric maps are given in Å. The radius of the sphere around the center was set to 3.5 Å, while for the atoms we adopted the Bondi radii scaled by 1.17, and a mesh of 0.1 Å was used to scan the sphere for buried voxels (the isocontour curves of the steric maps are given in Å).

All amines included in Scheme 2 were optimized by screening all potential isomers. To study the sterics, the high flexibility was overcome by means of the coordination of amines to the Au–Cl moiety, screening all isomers again. Then, the geometry of amine ligands in amine–Au–Cl complexes was evaluated by means of the %*V*_Bur_, developed by Cavallo and coworkers.^44^ The sterics were evaluated at 2.0 Å from the N atom, since this is the average value for the coordination of amines to metals.^38^ Further, only the first sphere around the metal was studied since this is where the reactivity takes place. The properties were calculated for the 45 tertiary amines, as well as NH_3_, NMe_3_, NEt_3_, and pyridine (Py) for comparison. Table 1 summarizes the overall %*V*_Bur_ (for additional details, see Table S1† divided into four quadrants to infer any asymmetry that would compensate, partially or completely, for a high overall %*V*_Bur_ value). Fig. 1 includes the odd cases 3 and 36, with a lower (39.4%) and higher occupation (70.3%), respectively, and also a third one, 28, with an intermediate value of 65.6%.

**Table 1:** Acidity and binding energies (Gibbs energies in kcal mol^−1^) for 45 amines and NH_3_, NMe_3_, NEt_3_, and Py with a proton, AuCl(P(CH_3_)_3_), Pd(P(CH_3_)_3_), and PdCl_2_(P(CH_3_)_3_). Total %*V*_Bur_ values of the amines

	H^+^^a^	H^+^^b^	Au^+^	Pd^0^	Pd^2+^	%*V*_Bur_
1	−0.3	22.8	24.0	13.8	3.9	49.8
2	−1.3	21.7	18.4	11.8	−12.7	25.2
3	−3.9	19.1	17.6	8.5	−9.1	39.4
4	−1.8	21.2	20.0	10.8	—c	36.5
5	−5.1	18.0	24.1	12.7	—c	48.0
6	−0.8	22.2	33.6	17.2	—c	55.8
7	4.2	27.2	35.0	17.4	—c	55.8
8	6.3	29.3	45.9	—c	—c	68.5
9	4.5	27.5	35.8	—c	—c	55.8
10	2.3	25.3	—c	—c	—c	63.3
11	−0.6	22.5	30.7	16.4	11.1	59.0
12	−5.3	17.8	23.5	13.4	—c	55.6
13	1.0	24.1	33.3	21.1	—c	55.0
14	−4.5	18.6	30.1	17.6	—c	61.6
15	−9.1	13.9	27.2	15.1	—c	56.2
16	−6.3	16.7	24.7	13.6	—c	54.0
17	−4.9	18.1	18.7	17.7	—c	59.3
18	−3.1	20.0	20.7	11.1	−5.8	43.3
19	−8.6	14.5	21.3	10.8	—c	51.9
20	−4.2	18.8	34.7	12.9	−2.4	46.2
21	−2.4	20.6	18.1	21.2	11.6	53.8
22	−3.3	19.8	19.4	10.5	−7.9	38.8
23	−2.1	20.9	31.8	9.7	−9.7	33.9
24	12.1	35.1	27.2	15.3	—c	43.8
25	13.2	36.2	34.8	13.1	−0.1	42.0
26	30.7	53.7	28.7	16.0	—c	38.6
27	10.0	33.0	27.0	13.5	—c	50.1
28	−8.4	14.6	32.9	14.1	—c	65.6
29	−4.5	18.6	27.5	18.1	—c	58.4
30	−5.5	17.5	30.7	—c	—c	61.0
31	−5.1	18.0	23.5	—c	—c	61.4
32	−6.5	16.5	—c	—c	—c	61.9
33	−3.7	19.4	32.2	19.5	—c	65.1
34	−7.0	16.1	31.0	—c	—c	68.2
35	−7.1	15.9	24.4	14.5	6.2	67.8
36	−6.2	16.8	—c	17.3	—c	70.3
37	−5.3	17.7	28.7	16.1	—c	63.6
38	−6.5	16.5	26.3	—c	8.0	67.4
39	−5.8	17.2	26.0	13.6	8.7	67.9
40	−5.6	17.4	—c	—c	—c	61.3
41	9.0	32.0	29.7	—c	—c	54.5
42	−7.9	15.2	25.4	13.3	—c	61.6
43	−3.4	19.6	—c	16.0	—c	63.1
NH_3_	8.4	31.4	20.7	11.0	−11.4	15.2
NMe_3_	−0.2	22.9	21.5	12.3	−7.0	29.7
NEt_3_	−3.2	19.8	21.0	11.2	0.4	44.8
Py	5.3	28.4	—c	—c	—c	21.3

a The proton is added as a free atom.

b The proton comes from acetic acid.

c Not located.

**Fig. 2:**
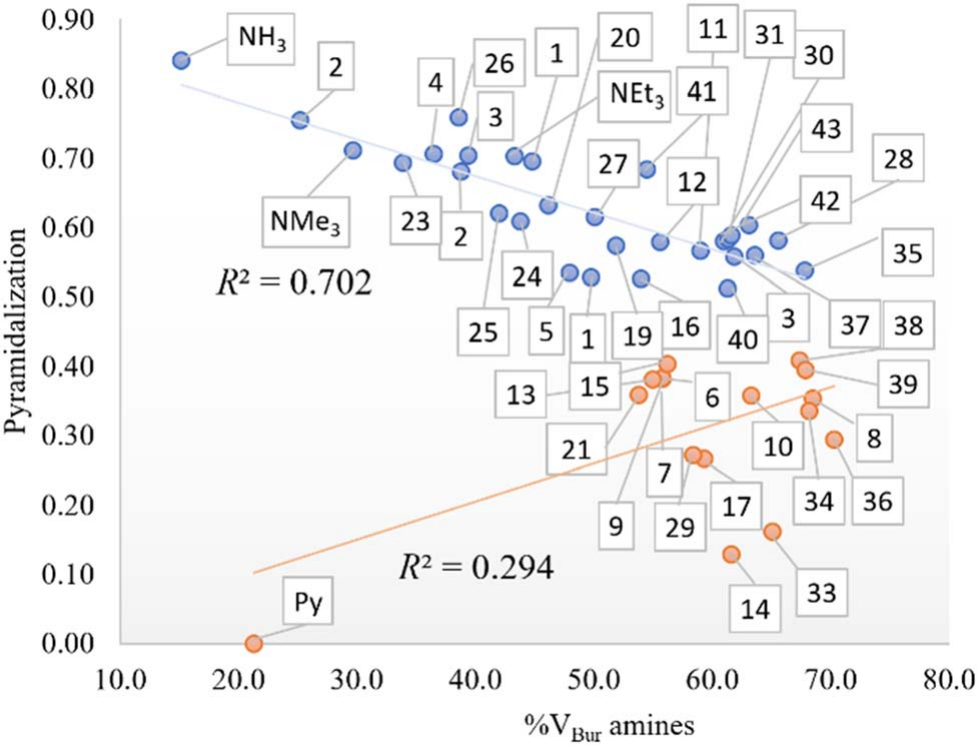
Pyramidalization *vs.* %*V*_Bur_ of the amines (in blue for a pyramidalization index higher than 0.450 and in red for lower values).

In addition, the role of sterics in tuning amine pyramidalization and the influence of C–N bond lengths were also analyzed. First, the pyramidalization was calculated according to the scheme of Radhakrishnan and Agranat.^45^ As aforementioned, in sterically hindered amines, the nitrogen atom deviates from sp^3^ hybridization to assume a planar shape in bulky amines. In the plot of pyramidalization *vs.* amines %*V*_Bur_, no correlation was found for all amines, but as shown in Fig. 2, the pyramidalization-%*V*_Bur_ trend deviates from linearity for pyramidalization values below 0.450. On the other hand, for greater values, the pyramidalization is almost directly proportional to amine %*V*_Bur_, with a Pearson coefficient (*R*^2^) of 0.702. The rest of the amines do not show any trend, but except for pyridine, with a pyramidalization index equal to zero, they have in common a *tert*-butyl moiety, which somehow in the presence of two other bulky groups favors the flattering of the amine to contrast the steric repulsion.

Apart from the sterics, amines were evaluated according to their capacity to trap protons, a fundamental property of the amine functional group, and thus acidity is also included in Table 1. Two approaches were used: either the addition of a proton (eqn (1)) or the deprotonation of an acetic acid molecule (eqn (2)). It should be noted that for the first approach for the aqueous solvation free energy of the proton, we assumed a value of −272.2 kcal mol^−1^ from the literature,^46^ to be compared with values of −250 kcal mol^−1^ in organic media.^47^ Our aim was to establish trends among the series of amines; however, it should be noted that this value is probably underestimated. We analyzed in detail how strongly the amines bond to metals using eqn (3)–(5). Next, we analyzed in detail how strongly the amines are bonded to the metals. As representative systems, the aforementioned amine–Au–Cl complexes were selected (eqn (3)) as well as palladium complexes. For the latter, the study included the oxidation state 0 as well as +2 (eqn (4) and (5)), to evaluate how the oxidation state on the metal center affects the affinity for the coordination of amines and to assist in future studies involving amine–metal(0) complexes.^48^ We must acknowledge that we omitted other relevant models, particularly the R_3_N–Ni(CO)_3_ standard in phosphine chemistry, which can be considered congeners of amines.^49^ Instead, we selected models from eqn (2)–(5), which are more typical of NHCs. Despite the significance of Ni(CO)_3_ systems, which also possess *C*_3V_ symmetry and could align with the three substituents on nitrogen, our goal was to focus on the models that reflect current research interests, especially those from the past decade.^50^

1 NR_3_ + H^+^ → (NR_3_)H^+^

2 NR_3_ + (CH_3_)COOH → (NR_3_)H^+^ + (CH_3_)COO^−^

3 AuCl(P(CH_3_)_3_) + NR_3_ → AuCl(NR_3_) + P(CH_3_)_3_

4 Pd(P(CH_3_)_3_)_2_ + NR_3_ → Pd(P(CH_3_)_3_)(NR_3_) + P(CH_3_)_3_

5 PdCl_2_(P(CH_3_)_3_) + NR_3_ → Pd(P(CH_3_)_3_)Cl_2_(NR_3_)

Faced with such a large set of similar species, in order to find trends, we had to select the key parameters. In the context of parameters, they must be robust and selective. In a systematic process, a series of reactions were carried out to evaluate the role of amines at the reactivity level.

### N-Protonation

Emphasizing the fundamental organic protonation of amines in eqn (2) and analyzing the thermodynamics of the process, the best correlation found with one variable was reached with the Mayer Bond Order (MBO) of the N–H bond, but the fitting is very modest (*R*^2^ = 0.527). By increasing the number of variables up to three, the correlations improve. In detail, the addition of the amine LUMO energy and the amine molecular area leads to better correlations with two (*R*^2^ = 0.731) and three (*R*^2^ = 0.785) variables. In general, the thermodynamics results are thus influenced by electronic factors since both amine LUMO energy and the MBO of the N–H bond are intrinsically linked to amine molecular orbitals. On the other hand, the kinetics of the proton transfer to the amine is more influenced by steric factors. The kinetics appears to be moderately correlated (*R*^2^ = 0.502) with the amine %*V*_Bur_ of the least occupied quadrant (Low %*V*_Bur_ amine) with one variable. In contrast, Table 2 shows that the steric-dependent descriptors as well as the pyramidalization on the nitrogen atom and pure steric descriptors such as amine %*V*_Bur_ and ammonium %*V*_Bur_ have some influence on kinetics when using two and three variables. The fit improved up to 0.869 (with two variables) and to 0.884 (with three variables) if the amine p*K*_a_ was also taken into account.

**Table 2:** Best correlations for the 43 tertiary amines and NH_3_, NMe_3_, NEt_3_, and Py (*R*^2^ = square of the Pearson correlation coefficient; RMSE = root mean squared error)

	Variables number		Filter	Systems number	Variables	Coefficients	Intercept	*R* ^2^
Eqn (2)	1	Δ*G*	—	45	(MBO(N–H^+^) ammonium)	[−681.7]	[626.0]	0.527
			Pyr > 0.450	29	(MBO(N–H^+^) ammonium)	[−1032.2]	[935.2]	0.715
	2		—	45	(*E*(LUMO) amine)	[−204.6]	[39.3]	0.731
					(Molecular area)	[−0.3]		
			Pyr > 0.450	29	(*E*(LUMO) amine)	[−304.7]	[45.6]	0.898
					(Molecular area)	[−0.4]		
	3		—	45	(*E*(LUMO) amine)	[−154.5]	[290.4]	0.785
					(Molecular area)	[−0.2]		
					(MBO(N–H^+^) ammonium)	[−287.1]		
			Pyr > 0.450	29	(*E*(LUMO) amine)	[−249.4]	[274.9]	0.914
					(Molecular area)	[−0.3]		
					(MBO(N–H^+^) ammonium)	[−264.5]		
	1	Δ*G*^‡^	—	39	(Low %*V*_Bur_ amine)	[0.3]	[−11.6]	0.502
			Pyr > 0.450	23	(High %*V*_Bur_ ammonium)	[0.2]	[−8.5]	0.651
	2		—	39	(p*K*_a_ amine)	[−1.6]	[−24.8]	0.869
					(%*V*_Bur_ amine)	[0.3]		
			Pyr > 0.450	23	(p*K*_a_ amine)	[−1.6]	[−24.3]	0.867
					(%*V*_Bur_ amine)	[0.3]		
	3		—	39	(p*K*_a_ amine)	[−1.4]	[−18.9]	0.884
					(Pyramidalization)	[−6.2]		
					(%*V*_Bur_ ammonium)	[0.3]		
			Pyr > 0.450	23	(p*K*_a_ amine)	[−1.6]	[493.0]	0.903
					(%*V*_Bur_ amine)	[0.2]		
					(*d*(N–H^+^) ammonium)	[−504.8]		
Eqn (3)	1	Δ*G*	—	39	(*d*(Au–N))	[117.4]	[−231.2]	0.679
			Pyr > 0.450	26	(*E*(LUMO) amine)	[−154.1]	[26.4]	0.688
	2		—	39	(*d*(Au–N))	[119.6]	[−241.2]	0.838
					(p*K*_a_ amine)	[−0.7]		
			Pyr > 0.450	26	(MBO(Au–N))	[−59.9]	[−186.5]	0.865
					(*d*(Au–N))	[112.1]		
	3		—	39	(*d*(Au–N))	[81.8]	[−168.4]	0.867
					(p*K*_a_ amine)	[−0.9]		
					(Low %*V*_Bur_ amine)	[0.2]		
			Pyr > 0.450	26	(MBO(Au–N))	[−98.7]	[85.1]	0.904
					(Pyramidalization)	[−23.8]		
					(Low %*V*_Bur_ ammonium)	[0.3]		
Eqn (4)	1	Δ*G*	—	36	(Pyramidalization Pd)	[−27.3]	[33.0]	0.603
			Pyr > 0.450	25	(MBO(Pd–N))	[−31.0]	[24.2]	0.580
	2		—	36	(MBO(Pd–N))	[−25.9]	[30.8]	0.766
					(Pyramidalization)	[−13.2]		
			Pyr > 0.450	25	(MBO(Pd–N))	[−29.1]	[20.4]	0.767
					(Low %*V*_Bur_ ammonium)	[0.1]		
	3		—	36	(MBO(Pd–N))	[−29.5]	[35.9]	0.803
					(Molecular area)	[−0.1]		
					(Pyramidalization)	[−15.2]		
			Pyr > 0.450	25	(MBO(Pd–N))	[18.4]	[406.8]	0.791
					(p*K*_a_ amine)	[−0.4]		
					(*d*(N–H^+^) ammonium)	[−431.0]		
Eqn (5)	1	Δ*G*	—	17	(Pyramidalization)	[−55.1]	[32.7]	0.808
			Pyr > 0.450	14	(%*V*_Bur_ amine)	[0.5]	[−23.0]	0.767
	2		—	17	(MBO(Pd–N))	[−91.5]	[11.0]	0.875
					(Low %*V*_Bur_ amine)	[0.5]		
			Pyr > 0.450	14	(MBO(Pd–N))	[−92.4]	[13.3]	0.883
					(Low %*V*_Bur_ amine)	[0.5]		
	3		—	17	(MBO(Pd–N))	[−140.5]	[−480.7]	0.922
					(Low %*V*_Bur_ ammonium)	[0.4]		
					(MBO(N–H^+^) ammonium)	[582.9]		
			Pyr > 0.450	14	(Low %*V*_Bur_ amine)	[0.4]	[44.6]	0.936
					(*η* amine)	[−7.4]		
					(Longest C–N bond)	[−9.9]		

**Fig. 3:**
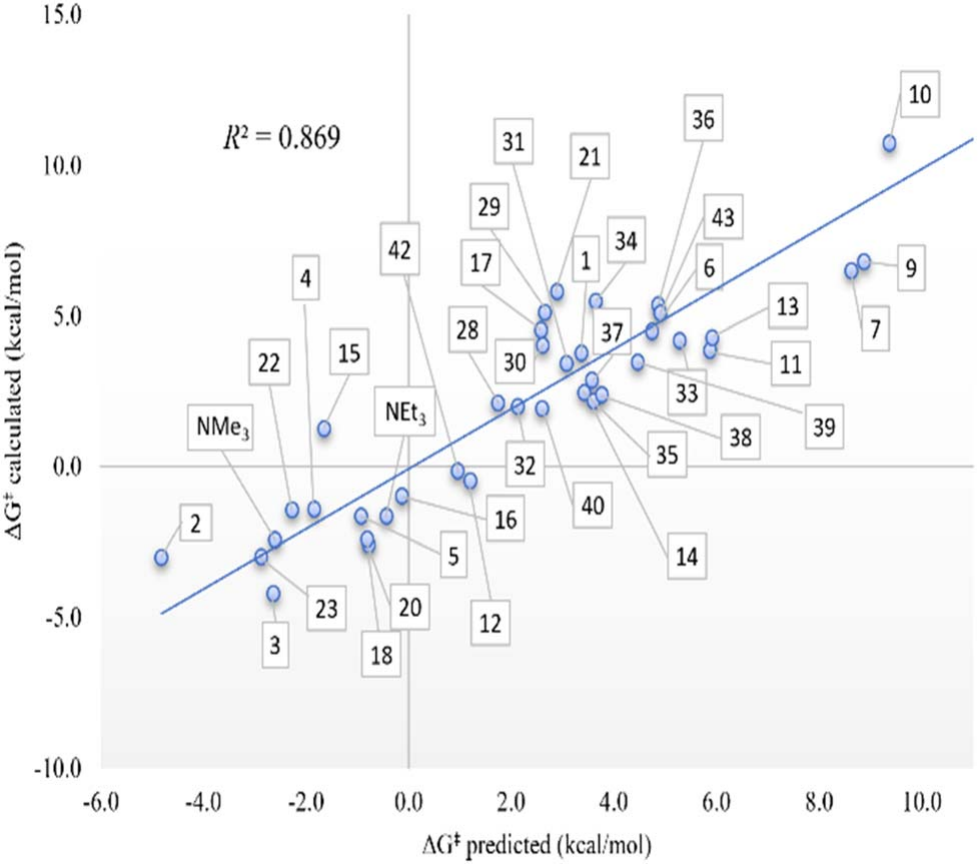
Computed theoretical Gibbs energies (Δ*G*^‡^) in kcal mol^−1^ in front of the modeled ones according to the multilinear adjustment with two variables (p*K*_a_ amine and pyramidalization) according to eqn (2).

In fact, for the kinetics, it is necessary to emphasize again the correlations, including the 39 amines. A great deal is achieved with two or three variables, with coefficients of 0.869 and 0.884, respectively. Despite the complexity of plotting all the systems in Fig. 3, we can perceive a series of facts that are remarkable in our opinion. All the substituents on the nitrogen are essentially electron-donating, perhaps unlike 10, which has electron-withdrawing ester groups that, in principle, hinder proton acceptance because the nitrogen loses electron density to stabilize more positive charge on it. It should be noted, however, that all these groups are not directly attached to N in the case of ester groups.^51^ This discussion of electrons is vaguely clear with just one system. In the case of the structural contribution, the differentiation is completely clear; thus, if the nitrogen is not substituted, *i.e.*, bearing hydrogen substituents, as in the case of amines 2, 3, 22, and 23, the protonation is in equilibrium. However, increasing the steric component of the nitrogen substituents has the opposite effect, but it is not really palpable if there are not at least two substituents that are highly sterically hindered, for example, with two adamantyl groups as in systems 38 and 39, and even more so if they are *tert*-butyls as in amines 33 and 36. Thus, for the latter systems, protonation holds significant value, kinetically speaking.

For the robustness of the results, we proceeded to perform two measurements, *i.e.*, convert the negative transition state energies to 0 and eliminate these systems. In the first case, the best correlations slightly worsened in terms of fit, with an *R*^2^ of 0.454, 0.821, and 0.842 for one, two, and three variables, respectively. On the other hand, the same variables involved in the best correlation reported in Table 2 still lead to the best correlation with two variables, whereas by using one variable, the amine LUMO energy is the descriptor that best fits the data rather than the amine Low %*V*_Bur_ descriptor in the previous case reported. The addition of NPA charge on nitrogen as the third variable only slightly improves the fitting up to 0.842. In the second case, the fit results better with the amine p*K*_a_ (*R*^2^ = 0.559), but yet with two variables, the addition of amine %*V*_Bur_ leads to a worse correlation than the previous one (*R*^2^ = 0.750 *vs. R*^2^ = 0.869). Finally, since pyramidalization on nitrogen is linearly dependent on amines %*V*_Bur_ for a pyramidalization index greater than 0.450, we looked at what occurs when removing the amines with values below 0.450. For thermodynamics, the fit improves up to 0.715, 0.898, and 0.914, respectively, for one, two, and three variables, even though we had to say the number of systems involved decreases to 29. Similar to thermodynamics, there is an improvement in fit up to 0.651, 0.867, and 0.904, respectively, for one, two, and three variables in the case of kinetics.

### Metal complexation

**Fig. 4:**
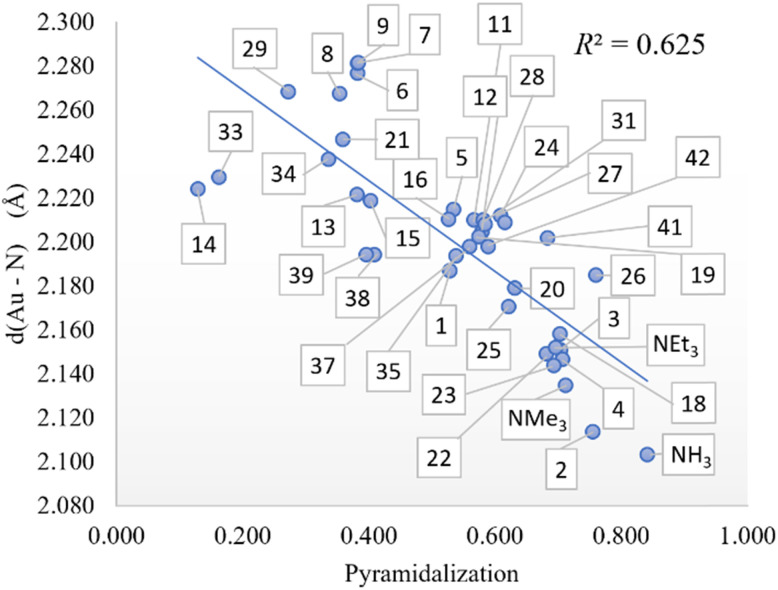
Pyramidalization *vs.* Au–N distance (in Å) correlation.

The exchange in eqn (3) of the phosphine by the amine in Au(i) complexes, using chlorine as an anionic ligand, led us to correlations that did not manage to exceed 0.679 with one variable with the Au–N distance. By increasing the number of variables, the correlations improve up to 0.838 with two variables and up to 0.867 with three variables, showing the influence of amine p*K*_a_ and Low %*V*_Bur_ amine on phosphine substitution. Furthermore, since the Au–N bond distance seems to influence more the thermodynamics of the phosphine-amine substitution, we looked for which descriptor may affect that distance more. Scanning along the previously used descriptors, the pyramidalization on the nitrogen atom (see Fig. 4) turns out to be the descriptor with the most impact in determining the Au–N distance, and as we show previously, it is indirectly linked in some way with amine %*V*_Bur_. Thus, to improve the thermodynamics of eqn (3), we can promote the shortening of the Au–N bond distance by acting on the pyramidalization and on %*V*_Bur_.

Removing the amines with a pyramidalization index below 0.450, the fit improves up to 0.688, 0.865, and 0.904, respectively, for one, two, and three variables, with the amine LUMO energy as the best descriptor with one variable, while MBO of the Au–N bond becomes more important with two and three variables, and p*K*_a_ is no longer involved in the correlation. The exchange of trimethylphosphine for the amines in eqn (4) represented our next approach. With one variable, the thermodynamics of eqn (4) led to the best correlation when nitrogen pyramidalization is involved when bonded to Pd (Pd pyramidalization in Table 2), reaching an *R*^2^ of 0.603. Using two and three variables, the pyramidalization still contributes, but the MBO of the Pd–N bond and the amine molecular area increase the fit up to 0.766 (with two variables) and up to 0.803 (with three variables). In contrast, applying the same filter as previously, pyramidalization loses importance and does not appear among the variables involved in the correlation, while MBO of the Pd–N bond still does. Unexpectedly, the best correlations have a worse overall fit. On the other hand, if we take into account the coordination of the amine not on Pd(0) as it was in eqn (4), but in Pd(ii) with two chlorides as in eqn (5), the pyramidalization on nitrogen still leads to the best correlation with one variable (*R*^2^ = 0.808). It is worth mentioning that most amines with a pyramidalization index less than 0.450 do not bond to Pd(ii). By completely excluding those amines from the correlations, the thermodynamics of eqn (5) correlate better with amine %*V*_Bur_ as it would be possible to imagine since %*V*_Bur_ and pyramidalization index are somehow dependent. However, in the case of two variables, the best correlation (*R*^2^ = 0.875) no longer involves the pyramidalization index, but the process seems to depend on steric (Low %*V*_Bur_ amine) and structural (MBO(Pd–N)) factors, while with three variables, the fit improves only slightly (*R*^2^ = 0.922). Last, applying the filter pyramidalization >0.450 with three variables, the fitting improves up to 0.936, but it is worth mentioning that MBO of Pd–N is not one of the variables, while the process depends on a steric factor (Low %*V*_Bur_ amine), an electronic factor (*η* amine), and the longest C–N bonds of amine.

## 4. Conclusions

In conclusion, steric effects surrounding the nitrogen atom of amines are of fundamental importance to the geometry and reactivity of this prominent functional group. We reported a blueprint for correlating the geometry of extremely hindered alkylamines with their key thermodynamic and kinetic properties. This is modest compared to machine learning,^52^ but also a real example of predictive catalysis.^38,53^ In particular, the optimization and evaluation of sterically hindered amines in coordination with gold and palladium highlights the focus of the study, based on the understanding of steric effects around the nitrogen atom and the ability of such amines to trap protons. The %*V*_Bur_ steric parameter of Cavallo and coworkers, directly or indirectly through the pyramidalization index, emerged as the key factor permitting the assessment of the geometrical effects of sterically hindered amines. The role of steric hindrance, represented by %*V*_Bur_ values, is highlighted, as is the importance of selective and robust parameters in understanding the reactivity of sterically hindered amines. The analysis involved various equations and statistical measures to establish correlations. For gold, the bond energies were influenced mainly by the Au–N distance, which correlates with the pyramidalization and indirectly with the %*V*_Bur_. For Pd(0), pyramidalization contributes more in combination with MBO of the Pd–N bond, while for Pd(ii), the relationship is similar to %*V*_Bur_ rather than pyramidalization. For proton thermodynamics, the LUMO is the most important parameter in combination with molecular area and N–H MBO, whereas kinetics is mainly described by %*V*_Bur_ parameters and amine p*K*_a_. Therefore, the steric contribution of sterically hindered amines can be summarized by the %*V*_Bur_ or the pyramidalization, while in some cases, additional weight should be given to the electronic part through the energy of the frontier orbitals or the structural part like distances or MBOs.

## Data availability

The data supporting the findings of this study are available within the article and its ESI.†

## Author contributions

M. T.: data curation, formal analysis, investigation, methodology, visualization, writing – original draft. M. V.: data curation, formal analysis, investigation, methodology, visualization, writing – original draft. L. C: data curation, formal analysis, investigation, validation, supervision, funding acquisition, review & editing. M. S.: conceptualization, data curation, formal analysis, investigation, funding acquisition, methodology, project administration, supervision, validation, visualization, review & editing. A. P.: conceptualization, data curation, funding acquisition, methodology, project administration, supervision, visualization, writing – original draft, review & editing.

## Conflicts of interest

There are no conflicts to declare.

## Supplementary Material

SC-015-D4SC03873H-s001
