# Supplementary material for: Tuning the steric hindrance of alkylamines: a predictive model of steric editing of planar amines
Source: Chem Sci. 2024 Jul 11;15(33):13405–14. doi: 10.1039/d4sc03873h (PMC11339794; doi:10.1039/d4sc03873h)
Supplement: SC-015-D4SC03873H-s001 [file SC-015-D4SC03873H-s001.pdf]

Electronic Supplementary Material (ESI) for Chemical Science.
This journal is © The Royal Society of Chemistry 2024

Supporting Information

for

Tuning the Steric Hindrance of Alkylamines: A Predictive Model of Steric Editing of Planar Amines.

Michele Tomasini,[a,b,†] Maria Voccia,[a,b,†] Lucia Caporaso,[b] Michal Szostak*[c] and Albert Poater*[a]

[a.] *Institut de Química Computacional i Catàlisi and Departament de Química, Universitat de Girona, c/ Mª Aurèlia Capmany 69, 17003 Girona, Catalonia, Spain.*
[b.] *Dipartimento di Chimica e Biologia, Università di Salerno, Via Ponte don Melillo, 84084, Fisciano, Italy.*
[c.] *Department of Chemistry, Rutgers University, 73 Warren Street, Newark, New Jersey 07102, United States.*

Table 1. Set of data.

AMINE	G Kinetics	G Thermodynamics	G Au	G Pd	G PdCl2
**1**	3.8	22.8	24.0	13.8	3.9
**2**	-3.0	21.7	18.4	11.8	-12.7
**3**	-4.2	19.1	17.6	8.5	-9.1
**4**	-1.4	21.2	20.0	10.8	-7.7
**5**	-1.6	18.0	24.1	12.7	-
**6**	4.5	22.2	33.6	17.2	-
**7**	6.5	27.2	35.0	17.4	-
**8**	16.8	29.3	45.9	-	-
**9**	6.8	27.5	35.8	-	-
**10**	10.8	25.3	-	-	-
**11**	3.9	22.5	30.7	16.4	11.1
**12**	-0.5	17.8	23.5	13.4	-
**13**	4.3	24.1	33.3	21.1	-
**14**	2.2	18.6	30.1	17.6	-
**15**	1.3	13.9	27.2	15.1	-
**16**	-1.0	16.7	24.7	13.6	-
**17**	4.5	18.1	-	17.7	-
**18**	-2.6	20.0	18.7	11.1	-5.8
**19**	-8.6	14.5	20.7	10.8	-
**20**	-2.4	18.8	21.3	12.9	-2.4
**21**	5.8	20.6	34.7	21.2	11.6
**22**	-1.4	19.8	18.1	10.5	-7.9
**23**	-3.0	20.9	19.4	9.7	-9.7
**24**	-	35.1	31.8	15.3	-
**25**	-	36.2	27.2	13.1	-0.1
**26**	-	53.7	34.8	16.0	-
**27**	-	33.0	28.7	13.5	-
**28**	2.1	14.6	27.0	14.1	-
**29**	5.1	18.6	32.9	18.1	-
**30**	4.0	17.5	-	-	-
**31**	3.4	18.0	27.5	-	-
**32**	2.0	16.5	-	-	-
**33**	4.2	19.4	32.2	19.5	-
**34**	5.5	16.1	31.0	-	-
**35**	2.5	15.9	24.4	14.5	6.2
**36**	5.4	16.8	-	17.3	-

**37**	2.9	17.7	28.7	16.1	-
**38**	2.4	16.5	26.3	-	8.0
**39**	3.5	17.2	26.0	13.6	8.7
**40**	1.9	17.4	-	-	-
**41**	-	32.0	29.7	-	-
**42**	-0.1	15.2	25.4	13.3	-
**43**	5.1	19.6	-	16.0	-
**NH_3**	-	-	20.7	11.0	-11.4
**NMe_3**	-2.4	22.9	21.5	12.3	-7.0
**NEt_3**	-1.6	19.8	21.0	11.2	0.4
**Py**	-0.9	28.4	21.3	10.2	-8.5

AMINE	pK_a amine	$\%V_{Bur}$ amine	High $\%V_{Bur}$ amine	Low $\%V_{Bur}$ amine
**1**	-8.3	49.8	56.6	44.6
**2**	-7.8	25.2	31.2	19.2
**3**	-6.5	39.4	47.2	29.6
**4**	-7.5	36.5	44.8	28.6
**5**	-5.9	48.0	53.7	42.1
**6**	-8.0	55.8	59.9	52.7
**7**	-10.4	55.8	59.4	52.2
**8**	-11.4	68.5	74.9	63.2
**9**	-10.6	55.8	59.6	52.2
**10**	-9.5	63.3	82.3	46.9
**11**	-8.1	59.0	67.5	48.4
**12**	-5.8	55.6	73.2	36.0
**13**	-8.9	55.0	59.2	50.6
**14**	-6.2	61.6	67.5	49.8
**15**	-3.9	56.2	58.5	53.2
**16**	-5.3	54.0	57.9	44.8
**17**	-6.0	59.3	64.2	54.4
**18**	-6.9	43.3	43.8	42.8
**19**	-4.2	51.9	53.0	50.8
**20**	-6.3	46.2	46.8	45.6
**21**	-7.2	53.8	60.0	49.2
**22**	-6.8	38.8	49.8	31.1
**23**	-7.3	33.9	35.0	32.4
**24**	-14.3	43.8	46.0	39.2
**25**	-14.8	42.0	44.8	36.3
**26**	-23.4	38.6	56.7	23.9
**27**	-13.3	50.1	62.3	28.8
**28**	-4.3	65.6	79.6	51.0
**29**	-6.2	58.4	62.6	54.2
**30**	-5.7	61.0	66.4	55.7
**31**	-5.9	61.4	74.9	46.1
**32**	-5.2	61.9	68.2	55.8
**33**	-6.6	65.1	71.5	55.4
**34**	-5.0	68.2	75.7	47.5
**35**	-4.9	67.8	84.9	55.5
**36**	-5.4	70.3	88.0	61.1
**37**	-5.8	63.6	77.3	48.6

**38**	-5.2	67.4	85.0	52.3
**39**	-5.6	67.9	86.3	52.1
**40**	-5.6	61.3	67.1	53.9
**41**	-12.8	54.5	79.1	40.4
**42**	-4.5	61.6	73.8	50.8
**43**	-6.7	63.1	72.2	53.8
**NH_3**	-12.5	15.2	15.7	14.8
**NMe_3**	-8.3	29.7	31.3	28.6
**NEt_3**	-6.8	44.8	50.1	40.2
**Py**	-11.0	21.3	21.3	21.3

AMINE	HOMO amine	LUMO amine	η amine	Tanimoto Index	Longest C-N bond
**1**	-0.20190	0.02978	6.3	1.000	1.473
**2**	-0.21454	0.02954	6.6	0.255	1.467
**3**	-0.22156	0.02449	6.7	0.176	1.484
**4**	-0.22222	0.03166	6.9	0.165	1.462
**5**	-0.20540	0.02926	6.4	0.224	1.493
**6**	-0.21000	-0.01640	5.3	0.114	1.506
**7**	-0.21397	-0.05841	4.2	0.076	1.508
**8**	-0.21530	-0.05113	4.5	0.072	1.510
**9**	-0.21374	-0.05165	4.4	0.093	1.509
**10**	-0.20546	-0.02491	4.9	0.090	1.475
**11**	-0.21228	-0.01871	5.3	0.046	1.482
**12**	-0.21936	0.03587	6.9	0.552	1.521
**13**	-0.19478	0.02694	6.0	0.725	1.484
**14**	-0.18934	0.02537	5.8	0.725	1.504
**15**	-0.21378	0.03960	6.9	0.725	1.525
**16**	-0.21166	0.02695	6.5	0.253	1.510
**17**	-0.21229	0.02870	6.6	0.250	1.501
**18**	-0.22538	0.03197	7.0	0.418	1.478
**19**	-0.18481	0.02410	5.7	0.033	1.525
**20**	-0.21949	0.02990	6.8	0.247	1.493
**21**	-0.21158	0.04245	6.9	1.000	1.463
**22**	-0.21802	0.02541	6.6	0.447	1.486
**23**	-0.22548	0.02944	6.9	0.568	1.469
**24**	-0.21937	-0.02142	5.4	0.288	1.502
**25**	-0.22994	-0.00466	6.1	0.368	1.482
**26**	-0.25356	-0.05273	5.5	0.183	1.521
**27**	-0.22911	-0.03047	5.4	0.288	1.519
**28**	-0.20360	0.01874	6.1	0.157	1.518
**29**	-0.19224	-0.00302	5.1	0.247	1.493
**30**	-0.17896	-0.00403	4.8	0.118	1.506
**31**	-0.18103	-0.00990	4.7	0.056	1.509
**32**	-0.20301	-0.01523	5.1	0.048	1.523
**33**	-0.19311	0.02977	6.1	0.416	1.510
**34**	-0.19266	0.03086	6.1	0.389	1.520
**35**	-0.18854	0.02639	5.8	0.252	1.523
**36**	-0.18587	0.02683	5.8	0.252	1.511
**37**	-0.15105	0.02364	4.8	0.174	1.486

**38**	-0.19014	0.01569	5.6	0.172	1.490
**39**	-0.18702	0.01428	5.5	0.172	1.490
**40**	-0.10848	0.00420	3.1	0.172	1.480
**41**	-0.22783	-0.03009	5.4	0.085	1.512
**42**	-0.20252	0.01977	6.0	0.172	1.524
**43**	-0.20656	-0.01116	5.3	0.133	1.516
**NH_3**	-0.27751	0.02893	8.3	0.000	0.000
**NMe_3**	-0.21548	0.03482	6.8	0.162	1.455
**NEt_3**	-0.20994	0.03704	6.7	0.541	1.466
**Py**	-0.27157	-0.04185	6.3	0.000	1.336

AMINE	Molecular Area	Pyramidalization	$\%V_{Bur}$ ammonium	High $\%V_{Bur}$ ammonium	Low $\%V_{Bur}$ ammonium
**1**	47.9	0.529	48.2	59.1	36.4
**2**	35.0	0.755	25.6	30.9	20.3
**3**	48.7	0.704	42.5	49.9	34.9
**4**	39.9	0.707	36.8	44.1	29.8
**5**	52.4	0.535	47.5	52.7	42.3
**6**	59.8	0.382	54.0	59.1	49.3
**7**	59.9	0.382	54.0	59.5	48.8
**8**	61.2	0.353	65.9	78.2	53.4
**9**	65.0	0.383	53.9	58.9	49.2
**10**	82.1	0.358	63.5	71.9	48.5
**11**	82.1	0.567	59.6	78.5	45.3
**12**	57.4	0.579	52.5	55.1	50.0
**13**	52.9	0.381	51.0	62.3	38.8
**14**	56.6	0.129	52.4	61.1	43.1
**15**	60.1	0.403	54.8	57.1	51.8
**16**	57.1	0.527	52.8	58.8	47.8
**17**	58.1	0.266	56.0	60.2	50.7
**18**	49.3	0.703	43.7	44.9	42.5
**19**	60.5	0.574	51.9	52.6	51.2
**20**	50.4	0.632	45.9	47.3	45.2
**21**	48.4	0.359	51.1	57.0	46.5
**22**	45.6	0.681	39.9	53.3	20.4
**23**	43.9	0.693	37.9	48.6	21.5
**24**	48.3	0.609	45.8	52.2	41.5
**25**	45.9	0.621	42.3	56.4	30.1
**26**	36.7	0.760	38.3	49.7	30.0
**27**	49.4	0.615	47.4	53.6	39.0
**28**	71.3	0.582	62.1	70.3	53.9
**29**	58.8	0.273	54.8	55.8	53.8
**30**	64.1	0.581	61.6	69.8	53.4
**31**	88.1	0.584	61.6	69.8	53.3
**32**	100.0	0.559	62.2	70.5	53.4
**33**	57.2	0.162	60.4	79.4	42.9
**34**	61.8	0.336	64.1	70.2	57.5
**35**	68.1	0.538	66.1	90.5	55.1
**36**	68.4	0.294	65.3	87.8	56.4
**37**	65.9	0.560	61.1	72.5	50.1

**38**	86.6	0.408	63.4	67.9	54.6
**39**	96.9	0.395	63.4	67.8	54.7
**40**	76.6	0.512	59.8	67.1	48.2
**41**	64.1	0.684	52.4	65.9	36.1
**42**	69.1	0.589	60.4	75.2	44.5
**43**	69.9	0.604	62.1	76.0	51.5
**NH_3**	12.9	0.841	16.3	16.8	15.8
**NMe_3**	29.2	0.712	30.6	31.7	29.6
**NEt_3**	44.2	0.696	45.0	50.0	40.4
**Py**	35.5	0.000	22.8	22.8	22.8

AMINE	N-NPA amine	N-NPA ammonium	MBO (N-H)	d(N-H)	MBO (Au-N)	d(Au-N)
**1**	-0.450	-0.341	0.892	1.021	0.580	2.187
**2**	-0.661	-0.496	0.883	1.021	0.535	2.114
**3**	-0.647	-0.510	0.885	1.022	0.556	2.151
**4**	-0.426	-0.319	0.881	1.023	0.574	2.147
**5**	-0.473	-0.376	0.888	1.022	0.600	2.215
**6**	-0.525	-0.451	0.889	1.020	0.561	2.277
**7**	-0.521	-0.410	0.885	1.020	0.558	2.281
**8**	-0.520	-0.429	0.870	1.022	0.600	2.268
**9**	-0.524	-0.413	0.886	1.020	0.556	2.282
**10**	-0.445	-0.374	0.886	1.023	-	-
**11**	-0.470	-0.388	0.883	1.020	0.542	2.210
**12**	-0.473	-0.383	0.882	1.020	0.622	2.205
**13**	-0.470	-0.367	0.893	1.020	0.627	2.221
**14**	-0.487	-0.405	0.893	1.020	0.638	2.224
**15**	-0.519	-0.409	0.894	1.018	0.666	2.219
**16**	-0.461	-0.368	0.886	1.021	0.596	2.210
**17**	-0.486	-0.405	0.893	1.019	-	-
**18**	-0.432	-0.333	0.881	1.024	0.596	2.158
**19**	-0.456	-0.397	0.887	1.020	0.638	2.202
**20**	-0.453	-0.354	0.886	1.023	0.593	2.179
**21**	-0.442	-0.367	0.897	1.019	0.538	2.247
**22**	-0.656	-0.504	0.889	1.021	0.565	2.149
**23**	-0.628	-0.487	0.888	1.023	0.557	2.144
**24**	-0.436	-0.347	0.882	1.023	0.479	2.212
**25**	-0.413	-0.330	0.879	1.022	0.509	2.171
**26**	-0.443	-0.389	0.857	1.026	0.412	2.185
**27**	-0.407	-0.367	0.880	1.023	0.522	2.209
**28**	-0.472	-0.405	0.889	1.020	0.591	2.210
**29**	-0.490	-0.398	0.895	1.020	0.601	2.268
**30**	-0.476	-0.394	0.890	1.020	-	-
**31**	-0.472	-0.391	0.890	1.020	0.593	2.208
**32**	-0.467	-0.397	0.891	1.019	-	-
**33**	-0.506	-0.401	0.899	1.019	0.643	2.229
**34**	-0.752	-0.422	0.904	1.018	0.627	2.238
**35**	-0.502	-0.393	0.888	1.018	0.624	2.194
**36**	-0.509	-0.409	0.896	1.018	-	-
**37**	-0.454	-0.372	0.892	1.019	0.579	2.198

**38**	-0.473	-0.383	0.885	1.019	0.582	2.194
**39**	-0.487	-0.384	0.882	1.020	0.603	2.194
**40**	-0.368	-0.365	0.889	1.021	-	-
**41**	-0.404	-0.379	0.876	1.022	0.488	2.202
**42**	-0.473	-0.409	0.890	1.019	0.597	2.198
**43**	-0.510	-0.446	0.882	1.020	-	-
**NH_3**	-1.069	-0.885	0.878	1.021	0.515	2.103
**NMe_3**	-0.418	-0.339	0.881	1.021	0.553	2.135
**NEt_3**	-0.442	-0.325	0.891	1.022	0.588	2.152
**Py**	-0.444	-0.394	0.887	1.014	-	-

AMINE	Pyramidalization Au	MBO (Pd-N)	d(Pd-N)	Pyramidalization Pd
**1**	0.764	0.387	2.364	0.734
**2**	0.797	0.377	2.214	0.790
**3**	0.781	0.395	2.277	0.764
**4**	0.784	0.402	2.272	0.773
**5**	0.683	0.395	2.395	0.645
**6**	0.645	0.310	2.570	0.572
**7**	0.647	0.302	2.577	0.570
**8**	0.665	-	-	-
**9**	0.647	-	-	-
**10**	-	-	-	-
**11**	0.755	0.262	2.467	0.715
**12**	0.722	0.411	2.407	0.689
**13**	0.611	0.386	2.424	0.558
**14**	0.624	0.399	2.452	0.569
**15**	0.634	0.427	2.490	0.576
**16**	0.728	0.381	2.428	0.685
**17**	-	0.353	2.576	0.542
**18**	0.790	0.421	2.301	0.777
**19**	0.707	0.431	2.386	0.680
**20**	0.751	0.406	2.343	0.726
**21**	0.661	0.271	2.562	0.588
**22**	0.779	0.404	2.275	0.758
**23**	0.780	0.393	2.268	0.764
**24**	0.739	0.300	2.387	0.692
**25**	0.783	0.327	2.311	0.789
**26**	0.795	0.245	2.303	0.794
**27**	0.709	0.318	2.386	0.687
**28**	0.755	0.321	2.483	0.711
**29**	0.617	0.353	2.574	0.537
**30**	-	-	-	-
**31**	0.752	-	-	-
**32**	-	-	-	-
**33**	0.625	0.394	2.454	0.577
**34**	0.629	-	-	-
**35**	0.701	0.397	2.383	0.669
**36**	-	0.351	2.490	0.574
**37**	0.617	0.311	2.448	0.537

**38**	0.716			
**39**	0.713	0.316	2.439	0.676
**40**	-	-	-	-
**41**	0.770	-	-	-
**42**	0.744	0.331	2.444	0.705
**43**	-	0.306	2.502	0.720
**NH_3**	0.819	0.376	2.187	0.826
**NMe_3**	0.780	0.399	2.247	0.770
**NEt_3**	0.785	0.421	2.287	0.774
**Py**	-	-	-	-

AMINE	G	MBO (Pd-N)	d(Pd-N)	Pyramidalization PdCl2
**1**	3.9	0.357	2.367	0.764
**2**	-12.7	0.377	2.182	0.769
**3**	-9.1	0.389	2.269	0.785
**4**	-7.7	0.369	2.301	0.786
**5**	-	-	-	-
**6**	-	-	-	-
**7**	-	-	-	-
**8**	-	-	-	-
**9**	-	-	-	-
**10**	-	-	-	-
**11**	11.145	0.317	2.397	0.776
**12**	-	-	-	-
**13**	-	-	-	-
**14**	-	-	-	-
**15**	-	-	-	-
**16**	-	-	-	-
**17**	-	-	-	-
**18**	-5.773	0.390	2.332	0.804
**19**	-	-	-	-
**20**	-2.443	0.378	2.378	0.763
**21**	11.631	0.352	2.467	0.675
**22**	-7.895	0.390	2.268	0.779
**23**	-9.736	0.391	2.239	0.765
**24**	-	-	-	-
**25**	-0.094	0.284	2.411	0.789
**26**	-	-	-	-
**27**	-	-	-	-
**28**	-	-	-	-
**29**	-	-	-	-
**30**	-	-	-	-
**31**	-	-	-	-
**32**	-	-	-	-
**33**	-	-	-	-
**34**	-	-	-	-
**35**	6.244	0.335	2.578	0.709
**36**	-	-	-	-
**37**	-	-	-	-

**38**	8.043	0.339	2.486	0.708
**39**	8.691	0.350	2.470	0.714
**40**	-	-	-	-
**41**	-	-	-	-
**42**	-	-	-	-
**43**	-	-	-	-
**NH_3**	-11.445	0.360	2.168	0.798
**NMe_3**	-6.987	0.362	2.279	0.783
**NEt_3**	0.360	0.374	2.344	0.800
**Py**	-	-	-	-

Table 2. xyz coordinates and absolute energies (in a.u.) of all computed amines.

28

1 SCF Done: -371.207506737 A.U.

N	0.000425	0.266350	0.184125
C	-0.301214	1.461063	-0.597570
C	0.266486	2.739159	0.017529
H	-1.384643	1.569313	-0.650681
H	0.043151	1.372433	-1.640939
H	-0.007145	3.605866	-0.589339
H	-0.127579	2.880926	1.025483
H	1.355520	2.714962	0.082610
C	1.389521	-0.201154	0.064457
C	1.689191	-1.073421	-1.166578
H	1.991680	0.704197	-0.036919
H	2.763154	-1.255874	-1.246413
H	1.193182	-2.043644	-1.096521
H	1.363425	-0.587205	-2.087648
C	-1.047263	-0.768285	0.199211
C	-2.135465	-0.403266	1.213987
H	-0.568347	-1.680421	0.559356
H	-2.895328	-1.186576	1.270073
H	-1.700866	-0.265277	2.204590
H	-2.638112	0.525450	0.932542
C	-1.679484	-1.101708	-1.164901
H	-2.344005	-1.962706	-1.063883
H	-2.278446	-0.270704	-1.542552
H	-0.927443	-1.342888	-1.914429
C	1.855829	-0.892152	1.348438
H	1.695689	-0.242637	2.209944
H	1.318412	-1.826955	1.522539
H	2.919112	-1.134126	1.285871

17

2 SCF Done: -252.01589795 A.U.

N	0.000174	-1.467198	-0.179517
C	1.260072	0.708591	-0.230068
C	1.216805	-0.754695	0.225943
C	-1.216625	-0.754986	0.225917
C	-1.260244	0.708317	-0.230048
H	2.081690	-1.303112	-0.154087
H	1.328786	0.738261	-1.323681
H	2.158544	1.195850	0.160016
H	-0.000161	1.524617	1.316461
H	-0.000278	2.476893	-0.159395
H	-2.081376	-1.303579	-0.154165
H	-1.271562	-0.791758	1.320216
H	-1.329024	0.738026	-1.323653
H	-2.158809	1.195331	0.160126
H	1.271689	-0.791440	1.320246
C	-0.000163	1.452884	0.222362
H	0.000207	-1.569364	-1.190099

29

3 SCF Done: -409.3454034 A.U.

N	-0.000034	-0.944342	-0.332342
C	1.253926	1.223722	-0.454901
C	1.284764	-0.262686	-0.036578
C	-1.284793	-0.262654	-0.036558
C	-1.253831	1.223742	-0.454894
H	1.278012	1.273243	-1.549460
H	2.156850	1.725180	-0.095029
H	0.000082	2.013888	1.126166
H	0.000096	2.977616	-0.331023
H	-1.277844	1.273175	-1.549459
H	-2.156745	1.725300	-0.095137
C	0.000059	1.947750	0.034722
H	0.000015	-1.159315	-1.323176
C	-1.647855	-0.398693	1.451127
H	-2.676923	-0.071017	1.612884
H	-1.560987	-1.440274	1.763298
H	-1.010844	0.200989	2.097144
C	-2.357173	-0.996275	-0.849710
H	-2.139244	-0.947051	-1.920042
H	-2.409021	-2.047164	-0.557868
H	-3.337026	-0.543953	-0.687305
C	2.357057	-0.996381	-0.849705
H	2.409211	-2.047165	-0.557558
H	2.138841	-0.947528	-1.920000
H	3.336849	-0.543769	-0.687702
C	1.647856	-0.398753	1.451075
H	1.011271	0.201436	2.097077
H	1.560478	-1.440243	1.763411
H	2.677107	-0.071579	1.612704

20

4 SCF Done: -291.341804617 A.U.

N	0.971669	-0.000002	-0.316048
C	-1.890448	0.000005	0.249710
C	-1.154923	1.253397	-0.226733
C	0.315255	1.208228	0.180614
C	0.315256	-1.208237	0.180608
C	-1.154928	-1.253393	-0.226726
H	0.845012	2.075845	-0.219810
H	-1.217451	1.322517	-1.317140
H	-1.617341	2.154651	0.183573
H	-1.931064	0.000009	1.344938
H	-2.923449	0.000001	-0.106354
H	0.845000	-2.075853	-0.219835
H	0.389280	-1.267240	1.284328
H	-1.217464	-1.322520	-1.317130
H	-1.617354	-2.154641	0.183588
H	0.389274	1.267220	1.284336
C	2.391487	0.000003	-0.006828
H	2.866268	-0.884396	-0.435983
H	2.866263	0.884412	-0.435965
H	2.591146	-0.000008	1.079930

32

5 SCF Done: -448.658629708 A.U.

N	0.000000	-0.718132	-0.377329
C	-0.000001	2.164488	-0.004008
C	1.236665	1.422634	-0.485838
C	1.288426	-0.047868	-0.031778
C	-1.288426	-0.047869	-0.031778
C	-1.236666	1.422633	-0.485837
H	1.250448	1.441615	-1.580522
H	2.148435	1.920274	-0.146202
H	-0.000001	2.250525	1.085765
H	-0.000001	3.186658	-0.391420
H	-1.250449	1.441615	-1.580522
H	-2.148436	1.920273	-0.146201
C	0.000001	-2.151246	-0.110747
H	-0.871176	-2.619240	-0.562165
H	0.871175	-2.619240	-0.562172
H	0.000006	-2.416969	0.956710
C	2.426196	-0.712878	-0.831542
H	2.133085	-0.839702	-1.874805
H	3.312504	-0.076862	-0.795861
H	2.712539	-1.684413	-0.428692
C	1.669635	-0.121041	1.465527
H	2.690512	0.241686	1.603444
H	1.018869	0.482748	2.093741
H	1.633965	-1.147539	1.832234
C	-2.426196	-0.712878	-0.831542
H	-3.312502	-0.076857	-0.795867
H	-2.133083	-0.839709	-1.874803
H	-2.712546	-1.684409	-0.428688
C	-1.669634	-0.121044	1.465527
H	-1.018866	0.482742	2.093742
H	-2.690510	0.241687	1.603446
H	-1.633967	-1.147543	1.832232

38

6 SCF Done: -602.339222173 A.U.

N	-0.546050	-0.000822	-0.273317
C	-1.168207	-1.341126	-0.000324
C	-0.411924	-2.397015	-0.832396
H	-0.897470	-3.367383	-0.716357
H	-0.419300	-2.128841	-1.889721
H	0.624007	-2.504510	-0.514289
C	-1.238855	1.315109	-0.037184
C	-2.307406	1.575850	-1.122763
H	-2.633943	2.616513	-1.069452
H	-1.882737	1.399286	-2.111689
H	-3.192617	0.958470	-1.013569
C	-1.881693	1.450641	1.356765
H	-2.319564	2.445403	1.466550
H	-2.677658	0.725417	1.515148
H	-1.139138	1.323016	2.145186
C	0.880445	0.008156	-0.114773
C	1.687214	-0.044733	-1.253632

C	1.511496	0.082130	1.131825
C	3.074028	-0.034159	-1.157893
H	1.204560	-0.093459	-2.220903
C	2.898785	0.097389	1.233805
H	0.911722	0.148523	2.028455
C	3.686875	0.035110	0.089363
H	3.675703	-0.076455	-2.057565
H	3.363758	0.161388	2.210059
H	4.766702	0.045282	0.168459
C	-1.101386	-1.784418	1.480606
H	-1.613289	-1.087470	2.140603
H	-1.576418	-2.761673	1.592615
H	-0.070452	-1.884372	1.816941
C	-2.633689	-1.407035	-0.452898
H	-2.733424	-1.146404	-1.505833
H	-2.990808	-2.429564	-0.322912
H	-3.285462	-0.763500	0.133510
C	-0.221311	2.465440	-0.187031
H	0.543453	2.453943	0.586857
H	0.274920	2.431664	-1.157322
H	-0.756421	3.412439	-0.112373

39

7 SCF Done: -694.623963076 A.U.

N	1.036965	-0.035235	0.278565
C	1.591815	-1.282378	-0.356381
C	1.151287	-2.489328	0.496637
H	1.610771	-3.397531	0.103975
H	1.465966	-2.362154	1.533276
H	0.071892	-2.634336	0.480073
C	1.587718	1.350769	0.052806
C	2.916368	1.538572	0.817745
H	3.181803	2.597508	0.829887
H	2.803273	1.200672	1.848450
H	3.749012	1.006522	0.370613
C	1.791437	1.714428	-1.429855
H	2.149882	2.742933	-1.510460
H	2.526754	1.074419	-1.913352
H	0.856225	1.643290	-1.986338
C	-0.367582	-0.109600	0.535857
C	-1.331088	0.096958	-0.451865
C	-0.809085	-0.385725	1.833001
C	-2.693920	0.025358	-0.144039
H	-1.029090	0.334817	-1.460489
C	-2.162173	-0.463036	2.142372
H	-0.063952	-0.536913	2.602207
C	-3.117044	-0.259871	1.158058
H	-4.172466	-0.315161	1.386138
H	-2.473059	-0.678179	3.156239
C	1.098582	-1.527774	-1.801078
H	1.364049	-0.711858	-2.469736
H	1.551521	-2.441376	-2.191844
H	0.018357	-1.665375	-1.835186

C	3.126371	-1.296341	-0.362030
H	3.525608	-1.170893	0.643471
H	3.461246	-2.263126	-0.739904
H	3.553201	-0.534216	-1.009736
C	0.620912	2.391545	0.655118
H	-0.333380	2.435061	0.133556
H	0.426159	2.190604	1.709192
H	1.082629	3.375873	0.578904
C	-3.659347	0.250924	-1.173379
N	-4.435809	0.432722	-2.006672

39

8 SCF Done: -694.625865996 A.U.

N	0.622336	-0.004699	-0.016713
C	1.286266	1.076783	-0.827154
C	0.417801	1.344166	-2.073016
H	0.911873	2.083581	-2.704540
H	0.282029	0.431542	-2.653247
H	-0.563922	1.739484	-1.813385
C	1.326260	-0.857238	1.011757
C	2.207711	-1.923438	0.326555
H	2.527841	-2.659601	1.066799
H	1.636244	-2.436810	-0.446460
H	3.104997	-1.511783	-0.123182
C	2.169906	-0.045326	2.010756
H	2.611954	-0.717865	2.748899
H	2.985705	0.486020	1.525080
H	1.554384	0.680741	2.543856
C	-0.768426	0.210601	0.200345
C	-1.702812	-0.549236	-0.530102
C	-1.274573	1.135693	1.115317
C	-3.081266	-0.372657	-0.363389
C	-2.642381	1.314568	1.284539
H	-0.582952	1.706894	1.716603
C	-3.549828	0.566075	0.541172
H	-3.770475	-0.973752	-0.940920
H	-3.001418	2.038197	2.005341
H	-4.614362	0.706779	0.671996
C	1.452902	2.421681	-0.082092
H	2.036781	2.319222	0.829365
H	1.967324	3.134556	-0.730047
H	0.487814	2.857762	0.172219
C	2.661499	0.635486	-1.345737
H	2.587893	-0.293461	-1.908793
H	3.043646	1.408088	-2.014178
H	3.391919	0.510115	-0.549820
C	0.284713	-1.646256	1.831663
H	-0.364381	-1.000614	2.420672
H	-0.337789	-2.274789	1.194676
H	0.816783	-2.299462	2.523041
C	-1.238094	-1.541162	-1.447956
N	-0.909473	-2.360055	-2.190420

39

9 SCF Done: -694.624155539 A.U.

N	-1.103202	-0.002276	-0.272816
C	-1.721271	-1.341813	0.029333
C	-0.987905	-2.408270	-0.809279
H	-1.475029	-3.374595	-0.671360
H	-1.019279	-2.152554	-1.869131
H	0.054310	-2.521251	-0.513486
C	-1.785185	1.322186	-0.034068
C	-2.871259	1.571473	-1.103611
H	-3.195301	2.612952	-1.055437
H	-2.464527	1.383620	-2.097870
H	-3.754643	0.956276	-0.971333
C	-2.400435	1.472972	1.369629
H	-2.834809	2.469471	1.474944
H	-3.194184	0.750994	1.551061
H	-1.644608	1.355020	2.146861
C	0.320402	0.002774	-0.146226
C	1.101720	-0.092106	-1.302034
C	0.978422	0.110420	1.085894
C	2.484849	-0.089434	-1.243470
H	0.599354	-0.166158	-2.256453
C	2.361281	0.118942	1.164757
H	0.399150	0.205989	1.992266
C	3.124453	0.014539	-0.003113
H	3.071742	-0.162614	-2.149025
H	2.852980	0.207937	2.124130
C	-1.616891	-1.763510	1.513284
H	-2.109701	-1.055961	2.176345
H	-2.091615	-2.737317	1.650797
H	-0.578259	-1.864532	1.825896
C	-3.196697	-1.405144	-0.387482
H	-3.320048	-1.157637	-1.440921
H	-3.554495	-2.424292	-0.235911
H	-3.831190	-0.751496	0.206329
C	-0.764529	2.464853	-0.217094
H	0.010709	2.466413	0.546653
H	-0.283122	2.415429	-1.194366
H	-1.294656	3.414823	-0.149966
C	4.550258	0.019490	0.069910
N	5.702339	0.023027	0.128730

54

10 SCF Done: -1060.71835807 A.U.

N	-0.202366	-0.077116	0.015417
C	-1.624602	-0.412070	-0.184734
C	-1.922470	-1.480079	-1.255638
C	-2.303783	-0.788299	1.141719
H	-2.114097	0.502932	-0.529817
C	-3.431743	-1.677893	-1.433885
H	-1.474386	-2.432877	-0.958219
H	-1.464599	-1.198021	-2.204646
C	-3.810561	-0.999664	0.965501
H	-1.856577	-1.709011	1.530779

H	-2.103429	-0.002164	1.869685
C	-4.110236	-2.043883	-0.112178
H	-3.618501	-2.449723	-2.185125
H	-3.872856	-0.751867	-1.820863
H	-4.259981	-1.293593	1.917682
H	-4.274620	-0.047784	0.681433
H	-5.189335	-2.145802	-0.254595
H	-3.742004	-3.021019	0.221447
C	0.691161	-1.216464	0.272703
C	1.456607	-1.802414	-0.932089
C	1.645183	-1.001364	1.460149
H	0.013356	-2.006577	0.602677
C	2.167827	-3.104270	-0.545452
H	2.195620	-1.082810	-1.285281
H	0.766131	-1.984811	-1.756036
C	2.349970	-2.307344	1.841161
H	2.409025	-0.261643	1.216636
H	1.077253	-0.602468	2.302641
C	3.103226	-2.901451	0.648642
H	2.723628	-3.492857	-1.402954
H	1.418623	-3.862659	-0.288973
H	3.033918	-2.130829	2.675378
H	1.605607	-3.030939	2.193366
H	3.573626	-3.847767	0.928583
H	3.912106	-2.220146	0.359567
C	0.230450	1.161139	-0.625184
C	0.194372	1.139746	-2.168673
H	-0.830635	1.001035	-2.507823
H	0.561674	2.075932	-2.584584
H	0.804548	0.323757	-2.541714
C	1.637564	1.602829	-0.131766
C	-0.662461	2.351461	-0.153542
O	2.610956	1.215893	-0.957266
O	1.832439	2.256294	0.863332
O	-0.895314	3.309473	-0.852682
O	-1.108227	2.220034	1.091165
C	3.953928	1.583793	-0.582813
H	4.592333	1.180158	-1.362538
H	4.045620	2.667358	-0.529708
H	4.207485	1.150352	0.383967
C	-1.894698	3.311585	1.603613
H	-2.171091	3.020592	2.612627
H	-1.305896	4.228008	1.616218
H	-2.783218	3.459360	0.990672

51

11 SCF Done: -909.960522644 A.U.

N	0.143588	0.987531	-0.211009
C	0.206228	2.467744	-0.232355
C	-0.781531	3.051625	-1.240821
H	-1.815509	2.905842	-0.924620
H	-0.608844	4.124781	-1.341223
H	-0.656429	2.587950	-2.220216

H	1.201713	2.716551	-0.600281
C	0.036095	3.134728	1.140441
H	0.746504	2.741456	1.869511
H	0.203331	4.210952	1.058742
H	-0.972514	2.980689	1.528379
C	-1.170916	0.450260	-0.004118
C	-1.920609	0.041724	-1.101643
C	-1.742324	0.298470	1.266197
C	-3.203074	-0.485208	-0.963286
H	-1.487852	0.139431	-2.088376
C	-3.010525	-0.233770	1.424676
H	-1.182876	0.590048	2.144865
C	-3.754828	-0.626743	0.309042
H	-3.748468	-0.786321	-1.845602
H	-3.446149	-0.352052	2.408733
O	-4.993689	-1.136134	0.565636
C	-5.794218	-1.557508	-0.532959
H	-6.725062	-1.920821	-0.104148
H	-6.007905	-0.727127	-1.212055
H	-5.312366	-2.365864	-1.090359
C	1.196870	0.299323	0.562863
H	1.041909	0.439506	1.644638
C	2.618528	0.815397	0.239416
C	1.170176	-1.224035	0.282592
H	2.682161	1.885604	0.440648
C	2.970746	0.536267	-1.230991
C	3.630891	0.102185	1.153391
H	0.173170	-1.610506	0.498327
C	1.521210	-1.504249	-1.187250
C	2.186281	-1.929125	1.195144
H	3.967777	0.931607	-1.449240
H	2.265261	1.044427	-1.892022
C	2.933139	-0.978920	-1.491359
H	3.400811	0.310940	2.202899
H	4.632397	0.498420	0.959976
C	3.600331	-1.410640	0.896787
H	0.791778	-1.024672	-1.841852
H	1.472268	-2.581666	-1.374188
H	1.935373	-1.753855	2.246128
H	2.136255	-3.009620	1.029510
H	3.182961	-1.176917	-2.537832
C	3.949148	-1.684588	-0.576383
H	4.324171	-1.910755	1.546813
H	3.939822	-2.762236	-0.768522
H	4.960997	-1.327054	-0.792956

35

12 SCF Done: -487.932754111 A.U.

N	0.008239	-0.139028	-0.236269
C	-1.421477	0.329330	-0.024823
C	-2.395806	-0.868625	-0.049189
H	-2.230783	-1.567441	0.767110
H	-3.409225	-0.478849	0.052310

H	-2.342280	-1.411090	-0.991538
C	-1.658867	1.037380	1.326134
H	-1.110823	1.971525	1.417962
H	-2.719976	1.270658	1.437161
H	-1.372951	0.390816	2.157697
C	1.159803	0.832767	-0.029146
C	0.858424	2.258529	-0.519432
H	0.066872	2.753523	0.034725
H	1.760280	2.857226	-0.386683
H	0.608495	2.268733	-1.579676
C	2.370297	0.376573	-0.876117
H	2.055325	0.130860	-1.890810
H	3.098743	1.187559	-0.929457
H	2.879421	-0.481238	-0.447718
C	1.632867	0.920727	1.439758
H	2.499314	1.582581	1.502649
H	0.864706	1.316001	2.101119
H	1.941318	-0.054129	1.816662
C	-1.887719	1.228918	-1.191021
H	-1.507141	2.242600	-1.147711
H	-1.586048	0.786425	-2.141295
H	-2.976933	1.299363	-1.178217
C	0.269420	-1.415306	0.397148
C	-0.136306	-2.723683	-0.232098
C	1.313477	-2.385096	-0.108287
H	0.177139	-1.428521	1.484101
H	-0.549399	-2.686272	-1.229730
H	-0.575029	-3.477005	0.409670
H	1.924317	-2.890126	0.629176
H	1.822295	-2.159091	-1.031181

34

13 SCF Done: -449.860041323 A.U.

N	0.143853	-0.034286	0.264361
C	-1.183156	-0.647684	0.006856
C	-1.359703	-1.877819	0.925820
H	-1.191487	-1.594905	1.965935
H	-2.375034	-2.267721	0.831245
H	-0.681299	-2.694149	0.679933
C	-2.330743	0.306777	0.372819
H	-2.344351	1.207057	-0.236006
H	-3.279743	-0.206782	0.212066
H	-2.278877	0.596292	1.422029
C	0.426464	1.397036	0.066344
C	0.067832	2.003408	-1.304420
H	-1.008275	2.081684	-1.455881
H	0.480361	3.013146	-1.375170
H	0.481527	1.414017	-2.121188
C	-0.066458	2.304893	1.206877
H	0.460523	3.261774	1.163078
H	-1.131957	2.520115	1.151656
H	0.140949	1.842288	2.172459
C	-1.370297	-1.100150	-1.459697

H	-1.304067	-0.255774	-2.144958
H	-0.612883	-1.831256	-1.745262
H	-2.347659	-1.570046	-1.593937
C	1.313974	-0.907458	0.054807
C	2.268195	-0.841352	1.254575
H	3.099008	-1.540412	1.129755
H	2.693645	0.158197	1.371588
H	1.736591	-1.091055	2.173571
C	2.088052	-0.698955	-1.260694
H	2.860366	-1.465547	-1.357379
H	1.430966	-0.771349	-2.126773
H	2.587585	0.271130	-1.293813
H	0.939065	-1.927374	0.017549
H	1.513106	1.458489	0.129243

37

14 SCF Done: -489.168228604 A.U.

N	0.000808	0.153031	-0.079607
C	1.503077	0.199340	-0.015021
C	2.044070	1.580230	0.429150
H	1.623877	1.898822	1.384323
H	3.123450	1.493378	0.557465
H	1.879106	2.362845	-0.311860
C	2.116116	-0.776446	1.016545
H	1.872650	-1.818082	0.839582
H	3.204001	-0.694239	0.981380
H	1.791682	-0.517131	2.025241
C	-0.748530	-1.137067	-0.012072
C	-0.110266	-2.252763	-0.870906
H	0.890771	-2.535317	-0.562859
H	-0.730330	-3.147939	-0.797107
H	-0.074759	-1.954822	-1.919120
C	-2.168968	-1.003040	-0.604034
H	-2.134374	-0.698739	-1.648688
H	-2.653385	-1.979111	-0.557063
H	-2.800027	-0.309033	-0.057269
C	-0.923907	-1.662597	1.437794
H	-1.412617	-0.916461	2.061715
H	-1.545911	-2.561549	1.440733
H	0.023710	-1.918926	1.902374
C	2.123678	-0.058220	-1.409832
H	1.951422	-1.069719	-1.765306
H	1.690868	0.634164	-2.134040
H	3.204728	0.103948	-1.386112
C	-0.719602	1.434835	0.010682
C	-1.535248	1.663534	1.297318
H	-1.870142	2.703691	1.335392
H	-2.422964	1.035858	1.364271
H	-0.919355	1.473154	2.177849
C	-1.518406	1.871192	-1.240371
H	-1.613767	2.960680	-1.235165
H	-0.985420	1.582924	-2.147346
H	-2.525581	1.463194	-1.289829

H	0.054623	2.183211	0.063177

40

15 SCF Done: -528.471555798 A.U.

N	-0.000056	0.000144	-0.272474
C	1.025538	1.102286	-0.029818
C	0.493757	2.543576	-0.234680
H	-0.323375	2.824569	0.418526
H	1.318975	3.217950	-0.006353
H	0.205591	2.726006	-1.267363
C	1.625216	1.100962	1.399213
H	2.094371	0.160732	1.668404
H	2.390501	1.877059	1.476007
H	0.860152	1.312131	2.145104
C	0.441867	-1.439142	-0.029736
C	1.955915	-1.698977	-0.234960
H	2.607913	-1.131806	0.418134
H	2.127558	-2.750841	-0.006904
H	2.257738	-1.540330	-1.267665
C	-0.183830	-2.400339	-1.086616
H	-0.251122	-1.894595	-2.049463
H	0.460576	-3.271655	-1.209165
H	-1.162959	-2.780563	-0.824169
C	0.140867	-1.958122	1.399183
H	0.430208	-3.008999	1.475640
H	0.706357	-1.401501	2.145269
H	-0.908008	-1.894273	1.668383
C	2.170748	1.040717	-1.086864
H	2.989810	0.383262	-0.824043
H	1.766461	0.728723	-2.049520
H	2.602907	2.034465	-1.210083
C	-1.467416	0.336997	-0.029877
C	-1.986833	1.359525	-1.086764
H	-3.063799	1.237918	-1.208607
H	-1.825571	2.397549	-0.824710
H	-1.515874	1.164242	-2.049860
C	-1.766462	0.856810	1.399120
H	-2.821445	1.130662	1.475971
H	-1.566096	0.089027	2.145216
H	-1.187412	1.733750	1.668039
C	-2.449360	-0.844292	-0.235228
H	-3.446155	-0.467066	-0.007189
H	-2.462799	-1.184922	-1.267975
H	-2.284156	-1.692511	0.417867

35

16 SCF Done: -487.985669891 A.U.

N	0.050575	0.217560	0.218060
C	-1.345871	-0.308480	-0.015743
C	-1.163170	-1.839893	-0.139078
C	0.190726	-2.105824	0.484650
C	1.035252	-0.894038	0.062637
H	-1.162577	-2.138290	-1.188891
H	-1.977563	-2.378981	0.345686

H	0.115511	-2.125927	1.574732
H	0.644878	-3.044497	0.164430
C	-2.227788	-0.019139	1.212254
H	-2.324403	1.045168	1.412220
H	-3.230925	-0.427896	1.063678
H	-1.793559	-0.486483	2.097755
C	-2.033017	0.197356	-1.296302
H	-2.958892	-0.363173	-1.441753
H	-2.295361	1.253454	-1.262812
H	-1.403317	0.030124	-2.171420
C	2.231955	-0.802714	1.024519
H	2.682729	-1.793629	1.121631
H	3.014210	-0.126871	0.689731
H	1.893728	-0.481413	2.010385
C	1.538242	-1.088027	-1.384106
H	2.073297	-0.212230	-1.748203
H	2.222961	-1.937819	-1.433349
H	0.714447	-1.278999	-2.072143
C	0.352087	1.560693	-0.312093
H	0.183230	1.593947	-1.400180
C	-0.538939	2.636997	0.329079
H	-0.268390	3.616587	-0.070003
H	-1.598441	2.495506	0.140275
H	-0.385574	2.650981	1.410545
C	1.797899	2.016434	-0.081537
H	2.532846	1.412630	-0.605276
H	1.900169	3.038044	-0.450963
H	2.042715	2.016659	0.981829

38

17 SCF Done: -527.308949638 A.U.

N	0.054104	0.008349	-0.174672
C	-0.754813	-1.246103	-0.014502
C	-2.125347	-0.727373	0.460045
C	-2.220187	0.651099	-0.147473
C	-0.797094	1.224569	-0.009469
H	-2.138437	-0.663631	1.550485
H	-2.933493	-1.396286	0.161735
H	-2.484309	0.583606	-1.205248
H	-2.955872	1.296646	0.333408
C	-0.931390	-1.958125	-1.374583
H	0.015640	-2.321004	-1.767436
H	-1.602573	-2.815469	-1.274447
H	-1.356009	-1.273139	-2.109471
C	-0.279407	-2.275391	1.025875
H	-1.040834	-3.055346	1.101883
H	0.656148	-2.765535	0.764812
H	-0.172623	-1.821201	2.010111
C	-0.646988	2.291400	-1.115023
H	-1.546020	2.913139	-1.121707
H	0.198917	2.958367	-0.972977
H	-0.556633	1.813636	-2.091232
C	-0.654613	1.908838	1.367081

H	0.343771	2.317902	1.514320
H	-1.362742	2.737252	1.448282
H	-0.853048	1.207450	2.178452
C	1.532515	0.019134	-0.031921
C	2.169608	-1.085286	-0.901198
H	3.256430	-1.031920	-0.812666
H	1.873187	-2.087274	-0.605044
H	1.904426	-0.944221	-1.949676
C	2.144371	1.332173	-0.557250
H	1.869010	2.195597	0.043604
H	3.231064	1.247539	-0.517010
H	1.861163	1.516834	-1.592091
C	2.041441	-0.152004	1.425685
H	3.121972	0.007311	1.462245
H	1.574849	0.570555	2.092539
H	1.848706	-1.146831	1.816217

26

18 SCF Done: -370.002099305 A.U.

N	-0.287432	-0.000772	-0.146582
C	2.650853	0.000830	0.118093
C	1.861766	-1.243983	-0.276618
C	0.441269	-1.200573	0.281492
C	0.440139	1.201258	0.276969
C	1.860683	1.243477	-0.281230
H	-0.091786	-2.084469	-0.059919
H	1.808621	-1.312918	-1.367819
H	2.355419	-2.151223	0.081326
H	2.807509	0.002894	1.202850
H	3.639736	0.000416	-0.346948
H	-0.093410	2.083336	-0.068327
H	0.481807	1.260057	1.382161
H	1.807413	1.308196	-1.372689
H	2.353637	2.152476	0.073195
H	0.483140	-1.254709	1.386908
C	-1.692986	-0.001397	0.311043
H	-1.715480	-0.004871	1.417567
C	-2.443565	-1.245062	-0.177724
H	-2.086917	-2.165759	0.280947
H	-3.502648	-1.153540	0.066731
H	-2.348907	-1.340330	-1.262314
C	-2.443046	1.245711	-0.169405
H	-3.502484	1.151749	0.072525
H	-2.087517	2.162856	0.297227
H	-2.346785	1.349669	-1.253065

38

19 SCF Done: -527.29140348 A.U.

N	-0.340016	0.000216	-0.291891
C	2.618924	-0.001601	0.175023
C	1.915363	-1.201641	-0.408115
C	0.411834	-1.305731	-0.056899
C	0.413339	1.305371	-0.056669
C	1.916927	1.199628	-0.407549

H	2.017155	-1.158287	-1.497342
H	2.381611	-2.136034	-0.089746
H	2.582304	-0.001858	1.267125
H	3.676084	-0.002151	-0.103375
H	2.019039	1.156746	-1.496759
H	2.384177	2.133299	-0.088523
C	-1.723786	0.000992	0.298382
H	-1.652399	0.001384	1.395255
C	-2.589467	-1.215019	-0.076344
H	-2.258194	-2.146301	0.367587
H	-3.599774	-1.039599	0.294074
H	-2.645679	-1.341061	-1.158470
C	-2.588195	1.217532	-0.077530
H	-3.599029	1.043263	0.291972
H	-2.256438	2.148688	0.366309
H	-2.643200	1.343184	-1.159757
C	0.319461	1.814922	1.399377
H	-0.705059	1.976539	1.726501
H	0.781951	1.118563	2.098465
H	0.840672	2.770484	1.486775
C	-0.073276	2.412107	-1.031333
H	0.711167	3.164909	-1.131206
H	-0.255642	1.979901	-2.015209
H	-0.965263	2.938063	-0.714845
C	0.317675	-1.815327	1.399164
H	-0.707009	-1.975448	1.726442
H	0.837620	-2.771594	1.486327
H	0.781286	-1.119640	2.098165
C	-0.076334	-2.411776	-1.031568
H	-0.258839	-1.979134	-2.015236
H	0.707427	-3.165205	-1.132152
H	-0.968650	-2.936971	-0.714776

29

20 SCF Done: -409.33262729 A.U.

N	-0.0600	0.00100	-0.223000
C	2.847976	-0.000067	0.145622
C	2.070720	-1.248268	-0.266186
C	0.633690	-1.197451	0.252280
C	0.633755	1.197605	0.252215
C	2.070838	1.248217	-0.266165
H	0.106847	-2.085802	-0.086473
H	2.047010	-1.323268	-1.357880
H	2.556732	-2.151593	0.112495
H	2.979363	-0.000085	1.233898
H	3.848172	-0.000123	-0.294889
H	0.107078	2.085951	-0.086751
H	0.656180	1.237336	1.356377
H	2.047197	1.323231	-1.357860
H	2.556921	2.151494	0.112539
H	0.656239	-1.237015	1.356434
C	-1.541067	-0.000017	-0.033974
C	-2.140717	-1.236751	-0.724783

H -1.903866 -2.163856 -0.203687
H -3.228003 -1.152125 -0.749517
H -1.776148 -1.313349 -1.750904
C -2.140677 1.238172 -0.722175
H -3.227921 1.153294 -0.747587
H -1.904297 2.164108 -0.198769
H -1.775685 1.317219 -1.747964
C -1.954401 -0.001504 1.451275
H -1.577782 0.882038 1.969204
H -3.042597 -0.000983 1.541299
H -1.578793 -0.886652 1.967188
31
21 SCF Done: -410.538509340 A.U.
N 0.000298 0.000212 -0.268251
C -1.347761 0.529839 -0.062221
C -2.287906 0.111311 -1.200457
H -2.429691 -0.971861 -1.219985
H -3.272519 0.569614 -1.079856
H -1.876205 0.415496 -2.163618
C -1.980152 0.205069 1.303398
H -1.343682 0.534744 2.124001
H -2.947528 0.703552 1.400756
H -2.151160 -0.867777 1.415448
C 1.132899 0.902524 -0.060737
C 1.240264 1.927516 -1.197647
H 0.373478 2.592440 -1.215563
H 2.129967 2.550325 -1.076913
H 1.296576 1.419806 -2.161406
C 1.167681 1.610344 1.306070
H 2.082631 2.199448 1.404078
H 0.323564 2.294033 1.419604
H 1.135903 0.892942 2.125519
C 0.215217 -1.432180 -0.062970
C 0.808680 -1.818917 1.304017
H 0.862221 -2.906024 1.399474
H 1.822203 -1.429024 1.420398
H 0.201340 -1.435313 2.123303
C 1.051053 -2.035546 -1.199585
H 1.145829 -3.117601 -1.080417
H 0.584380 -1.829380 -2.163611
H 2.060124 -1.616906 -1.215533
H -0.771305 -1.896346 -0.121927
H 2.028232 0.280185 -0.117313
H -1.256299 1.616409 -0.117890
25
22 SCF Done: -331.889165292 A.U.
N 0.210013 -0.568144 -0.390992
C -1.131603 -0.052744 -0.014072
C -2.129758 -1.158073 -0.385977
H -1.960953 -2.055877 0.214932
H -3.154697 -0.826654 -0.213822
H -2.028007 -1.424999 -1.440022

C	-1.268718	0.257817	1.486060
H	-0.614558	1.075428	1.792388
H	-2.294281	0.551766	1.718479
H	-1.022224	-0.619393	2.088613
C	1.428035	-0.085235	0.276823
C	1.767720	1.359429	-0.087908
H	2.744586	1.625803	0.321121
H	1.800122	1.480146	-1.173136
H	1.042788	2.066280	0.313136
C	2.573894	-1.013942	-0.125949
H	3.500717	-0.717661	0.368136
H	2.361654	-2.049089	0.153996
H	2.730863	-0.979055	-1.206601
H	1.333569	-0.138006	1.372207
C	-1.473481	1.198221	-0.839449
H	-0.816345	2.033905	-0.607103
H	-1.381105	0.980215	-1.904564
H	-2.499023	1.516121	-0.637541
H	0.190270	-1.574771	-0.280436

22

23 SCF Done: -292.560485416 A.U.

N	0.012283	-0.623134	0.080588
C	1.239603	0.119040	-0.233222
C	2.354791	-0.888369	-0.504006
H	2.092878	-1.552936	-1.331500
H	3.281564	-0.375603	-0.767599
H	2.537265	-1.502439	0.381043
C	-1.235880	0.136679	0.235579
C	-2.353006	-0.842789	0.589144
H	-3.289433	-0.314633	0.777234
H	-2.525097	-1.543056	-0.234223
H	-2.093225	-1.420340	1.477365
C	-1.620873	0.985499	-0.983787
H	-2.560686	1.512967	-0.805622
H	-0.860669	1.733031	-1.214719
H	-1.750110	0.349470	-1.864560
H	-1.099807	0.808177	1.087953
C	1.619455	1.055375	0.912449
H	0.870548	1.832565	1.070943
H	1.728055	0.490806	1.841840
H	2.567293	1.551099	0.694356
H	-0.130679	-1.331861	-0.632764
H	1.121581	0.732077	-1.140803

25

24 SCF Done: -791.479815573 A.U.

N	0.129324	0.163608	-0.444570
C	1.276585	-0.752803	-0.239823
C	2.486339	-0.243486	-1.024184
H	2.931954	0.630189	-0.547538
H	3.246364	-1.025139	-1.071669
H	2.198177	0.025765	-2.040651
C	1.657756	-1.089646	1.205228

H	0.880112	-1.656019	1.713609
H	2.565857	-1.695152	1.210654
H	1.854703	-0.181326	1.776212
C	-1.268184	-0.258826	-0.091210
C	-1.477930	-1.674060	-0.653204
H	-0.925014	-2.427491	-0.092563
H	-2.537209	-1.921576	-0.579686
H	-1.188670	-1.728757	-1.703750
C	-1.606863	-0.252269	1.408955
H	-2.660574	-0.504593	1.539903
H	-1.020000	-0.979609	1.965323
H	-1.441236	0.731710	1.845790
H	0.950832	-1.669510	-0.731580
Cl	0.502555	1.760831	0.247587
C	-2.220745	0.694270	-0.829412
H	-2.129355	1.714464	-0.458857
H	-2.002968	0.694091	-1.898071
H	-3.253418	0.374481	-0.682207

22

25 SCF Done: -752.150129285 A.U.

N	-0.002777	0.235531	-0.444224
C	1.266975	-0.527715	-0.493502
C	2.357885	0.365036	-1.083048
H	2.576235	1.196036	-0.409564
H	3.278357	-0.204079	-1.225709
H	2.045234	0.773260	-2.044242
C	1.751051	-1.184109	0.804325
H	0.991139	-1.809207	1.266411
H	2.618153	-1.810012	0.586022
H	2.057159	-0.428380	1.528277
C	-1.273112	-0.516041	-0.549941
C	-1.645992	-1.431164	0.620921
H	-2.635090	-1.859355	0.449322
H	-0.944761	-2.255670	0.735702
H	-1.679304	-0.868388	1.555037
H	1.044757	-1.318624	-1.216350
Cl	-0.022942	1.361326	0.926458
C	-2.413246	0.443221	-0.885938
H	-2.677834	1.056720	-0.023531
H	-2.131198	1.103740	-1.706266
H	-3.298316	-0.123061	-1.179939
H	-1.096442	-1.139606	-1.432302

16

26 SCF Done: -1133.08301282 A.U.

N	0.390149	0.000048	-0.551746
C	-1.017447	-0.000150	0.023279
C	-1.696009	-1.249325	-0.555304
H	-1.295469	-2.165333	-0.126030
H	-2.760025	-1.203288	-0.322096
H	-1.581104	-1.287128	-1.638952
C	-1.073384	-0.000019	1.548845
H	-2.116035	-0.000109	1.868182

H -0.591587 -0.885970 1.961467
H -0.591813 0.886231 1.961106
Cl 1.315829 1.424773 -0.015501
C -1.696410 1.249016 -0.555182
H -1.295834 2.165019 -0.126090
H -1.581984 1.286677 -1.638872
H -2.760274 1.202780 -0.321487
Cl 1.316088 -1.424558 -0.015371
28
27 SCF Done: -830.798655493 A.U.
N -0.008651 0.221844 -0.434954
C 1.334430 -0.359526 -0.028162
C 2.375512 0.254791 -0.982576
H 2.478958 1.328084 -0.837945
H 3.349834 -0.202371 -0.803787
H 2.089341 0.070581 -2.018782
C 1.760745 -0.093224 1.427698
H 1.101042 -0.578133 2.143311
H 2.765460 -0.492051 1.578653
H 1.788907 0.971516 1.650323
C -1.350863 -0.342813 -0.009586
C -1.503879 -0.559277 1.500842
H -2.535844 -0.832203 1.729788
H -0.863196 -1.361598 1.863765
H -1.266000 0.353860 2.047592
Cl 0.041491 1.972846 -0.055359
C -2.457289 0.617158 -0.491456
H -2.509986 1.524603 0.104411
H -2.300998 0.893054 -1.534958
H -3.418609 0.109995 -0.408962
C 1.361729 -1.876835 -0.262742
H 0.760931 -2.421703 0.461430
H 1.041316 -2.137901 -1.269867
H 2.392611 -2.210922 -0.141615
C -1.597648 -1.663611 -0.769553
H -1.036911 -2.503053 -0.376321
H -2.654487 -1.916497 -0.680257
H -1.363581 -1.546522 -1.827784
50
28 SCF Done: -759.676008919 A.U.
N -1.083232 0.004931 -0.167951
C -1.977118 -1.214148 -0.025267
C -3.416982 -0.645983 -0.006535
C -3.276926 0.756792 -0.552250
C -1.924233 1.235525 -0.005187
H -3.799867 -0.618505 1.014949
H -4.088729 1.424451 -0.260259
C -1.825470 -2.118770 -1.259887
H -0.815590 -2.505489 -1.373412
H -2.505891 -2.971579 -1.189450
H -2.070256 -1.555965 -2.162244
C -1.790699 -2.037264 1.264814

H	-2.591679	-2.777662	1.319285
H	-0.847476	-2.577339	1.314007
H	-1.866212	-1.405143	2.150319
C	-1.493813	2.430540	-0.870157
H	-2.317817	3.147857	-0.901250
H	-0.622556	2.960939	-0.495637
H	-1.291983	2.105285	-1.890642
C	-2.080360	1.690301	1.462410
H	-1.126851	1.986194	1.898262
H	-2.750747	2.550714	1.519411
H	-2.495453	0.899082	2.086538
C	0.222447	-0.076749	0.522965
H	0.055128	-0.234835	1.599095
C	1.118849	1.188175	0.421043
C	1.104196	-1.263408	0.030634
H	0.564273	2.064521	0.743175
C	1.658569	1.413241	-1.001950
C	2.313375	1.024368	1.381429
H	0.535219	-2.186766	0.047265
C	1.624541	-1.032373	-1.396094
C	2.297034	-1.434011	0.988020
H	2.251321	2.333749	-1.019943
H	0.841078	1.541447	-1.708956
C	2.515173	0.216092	-1.431220
H	1.955173	0.891774	2.407047
H	2.912456	1.940190	1.367295
C	3.172673	-0.175495	0.962289
H	0.785507	-0.908144	-2.083403
H	2.187851	-1.912807	-1.722050
H	1.937736	-1.624827	2.004264
H	2.882606	-2.307852	0.685694
H	2.889290	0.376237	-2.446750
C	3.699053	0.046053	-0.465457
H	4.012936	-0.290573	1.653307
H	4.315950	-0.804608	-0.772670
H	4.336951	0.935255	-0.495958
H	-4.098784	-1.269804	-0.585195
H	-3.232832	0.742369	-1.644047

36
29 SCF Done: -526.080502876 A.U.

N	0.011833	-0.000023	-0.177012
C	-0.816200	-1.231382	-0.009846
C	-2.198640	-0.659082	0.148505
C	-2.198980	0.657949	0.148508
C	-0.816820	1.230975	-0.009866
H	-3.059903	-1.306242	0.248353
H	-3.060582	1.304660	0.248341
C	-0.828416	-2.143505	-1.261209
H	0.079959	-2.732115	-1.364345
H	-1.664456	-2.844562	-1.194851
H	-0.956617	-1.538502	-2.158829
C	-0.519544	-2.091457	1.240024

H	-1.278693	-2.872514	1.326365
H	0.449094	-2.586050	1.186994
H	-0.553975	-1.483665	2.143949
C	-0.829512	2.143103	-1.261185
H	-1.666060	2.843562	-1.194902
H	0.078437	2.732395	-1.364214
H	-0.957212	1.538048	-2.158843
C	-0.520679	2.091187	1.240047
H	0.447658	2.586382	1.187070
H	-1.280294	2.871796	1.326335
H	-0.554847	1.483354	2.143941
C	1.488113	0.000377	-0.032944
C	2.107049	-1.222492	-0.738069
H	3.194394	-1.147844	-0.687797
H	1.829892	-2.164582	-0.272007
H	1.816244	-1.253771	-1.787943
C	2.106389	1.223723	-0.737817
H	1.828863	2.165559	-0.271439
H	3.193771	1.149542	-0.687707
H	1.815472	1.255174	-1.787643
C	1.995209	0.000349	1.433593
H	3.088041	0.000990	1.446921
H	1.659580	0.880146	1.976628
H	1.660591	-0.880069	1.976259

48

30 SCF Done: -758.447382765 A.U.

N	-1.118152	0.000014	-0.169052
C	-1.987287	-1.221891	-0.033450
C	-3.362389	-0.658900	-0.276027
C	-3.362457	0.658704	-0.275884
C	-1.987357	1.221811	-0.033524
H	-4.219579	-1.307223	-0.401046
H	-4.219686	1.306951	-0.401050
C	-1.702049	-2.284182	-1.111690
H	-0.763977	-2.811077	-0.954327
H	-2.500659	-3.030328	-1.105716
H	-1.675654	-1.816087	-2.095766
C	-1.991672	-1.877216	1.369465
H	-2.767276	-2.645038	1.407407
H	-1.044772	-2.356020	1.614463
H	-2.213063	-1.140541	2.141230
C	-1.702293	2.283906	-1.111887
H	-2.501458	3.029461	-1.106485
H	-0.764664	2.811460	-0.954207
H	-1.675076	1.815573	-2.095841
C	-1.991751	1.877482	1.369330
H	-1.044855	2.356371	1.614155
H	-2.767405	2.645246	1.407166
H	-2.213046	1.140884	2.141179
C	0.174893	0.000052	0.544124
H	-0.001887	0.000093	1.631245
C	1.060964	1.241467	0.241486

C	1.061061	-1.241420	0.241624
H	0.500202	2.153713	0.419125
C	1.573248	1.239082	-1.207950
C	2.263950	1.244563	1.202408
H	0.500332	-2.153642	0.419351
C	1.573305	-1.239182	-1.207811
C	2.264010	-1.244390	1.202535
H	2.149139	2.152624	-1.388323
H	0.734887	1.238735	-1.904931
C	2.444146	-0.000036	-1.450856
H	1.915727	1.272654	2.239727
H	2.854002	2.151460	1.037827
C	3.129158	0.000099	0.967675
H	0.734933	-1.238999	-1.904773
H	2.149276	-2.152691	-1.388070
H	1.915825	-1.272392	2.239873
H	2.854143	-2.151257	1.038058
H	2.806868	-0.000094	-2.482964
C	3.638910	0.000028	-0.483284
H	3.977445	0.000145	1.658402
H	4.264511	-0.880842	-0.659977
H	4.264493	0.880894	-0.660073

54

31 SCF Done: -912.165447840 A.U.

N	0.326887	0.000012	-0.070993
C	1.176862	1.231323	0.122703
C	2.576388	0.689710	-0.050884
C	2.576382	-0.689701	-0.050865
C	1.176852	-1.231295	0.122710
C	0.923425	2.296004	-0.960497
H	-0.039505	2.787384	-0.857420
H	1.689023	3.071695	-0.900482
H	0.975867	1.837593	-1.947837
C	1.091865	1.866750	1.532091
H	1.824629	2.671653	1.609168
H	0.112063	2.290518	1.744039
H	1.321193	1.132639	2.304129
C	0.923444	-2.295956	-0.960538
H	1.689118	-3.071569	-0.900610
H	-0.039446	-2.787410	-0.857424
H	0.975783	-1.837463	-1.947849
C	1.091804	-1.866746	1.532073
H	0.111892	-2.290221	1.744096
H	1.824336	-2.671871	1.609054
H	1.321434	-1.132728	2.304113
C	-1.011494	0.000009	0.564427
H	-0.894555	-0.000003	1.659044
C	-1.883228	-1.240572	0.215916
C	-1.883258	1.240575	0.215959
H	-1.337203	-2.154375	0.425679
C	-2.319505	-1.239128	-1.258253
C	-3.135595	-1.242829	1.112305

H	-1.337261	2.154382	0.425761
C	-2.319543	1.239186	-1.258207
C	-3.135620	1.242760	1.112356
H	-2.885960	-2.152446	-1.467066
H	-1.447246	-1.240356	-1.912312
C	-3.175904	0.000021	-1.546969
H	-2.842337	-1.270060	2.166501
H	-3.714376	-2.150877	0.917260
C	-3.988090	-0.000039	0.831639
H	-1.447290	1.240484	-1.912277
H	-2.886032	2.152497	-1.466963
H	-2.842360	1.269951	2.166553
H	-3.714424	2.150802	0.917356
H	-3.483232	0.000026	-2.596817
C	-4.420375	-0.000013	-0.644156
H	-4.871579	-0.000057	1.476673
H	-5.035655	0.880849	-0.853727
H	-5.035627	-0.880886	-0.853765
C	3.767932	-1.398479	-0.160755
C	3.767957	1.398452	-0.160789
C	4.964322	-0.697415	-0.276310
C	4.964332	0.697360	-0.276332
H	3.774450	-2.481432	-0.155255
H	5.898914	-1.236843	-0.366526
H	5.898929	1.236777	-0.366562
H	3.774517	2.481406	-0.155333

66

32 SCF Done: -1069.45936079 A.U.

N	0.168500	0.027284	-0.148032
C	1.043900	1.259347	0.040868
C	2.434775	0.687631	-0.141630
C	2.405785	-0.689128	-0.223445
C	1.010490	-1.232178	0.005839
C	0.739256	2.269833	-1.103864
H	-0.303940	2.555686	-1.049278
H	0.847560	1.716105	-2.038628
C	1.058243	1.919991	1.465684
H	2.002188	2.464263	1.520588
H	1.154829	1.114741	2.196342
C	0.741775	-2.303326	-1.115462
H	1.717710	-2.622273	-1.477502
H	0.294694	-1.764308	-1.950292
C	0.942773	-1.814588	1.455005
H	-0.033696	-2.268323	1.612500
H	0.996557	-0.973689	2.147479
C	-1.176601	0.055330	0.483345
H	-1.058437	0.128719	1.574896
C	-2.048113	-1.206349	0.234449
C	-2.075696	1.254851	0.058187
H	-1.488479	-2.094758	0.491271
C	-2.539676	-1.319444	-1.218850
C	-3.277046	-1.159359	1.163608

H	-1.543046	2.186224	0.181273
C	-2.549595	1.143203	-1.399530
C	-3.306102	1.315656	0.982682
H	-3.111693	-2.246054	-1.331320
H	-1.698535	-1.374371	-1.908333
C	-3.411554	-0.111758	-1.574424
H	-2.956481	-1.108637	2.208830
H	-3.847478	-2.086237	1.048343
C	-4.153992	0.050318	0.819537
H	-1.692167	1.096021	-2.072675
H	-3.120211	2.040456	-1.660413
H	-2.992360	1.423306	2.025487
H	-3.896200	2.203292	0.734442
H	-3.747779	-0.190463	-2.612417
C	-4.629418	-0.054927	-0.638673
H	-5.017753	0.088762	1.489690
H	-5.257299	0.805922	-0.889924
H	-5.243377	-0.951780	-0.769641
C	3.582831	-1.406059	-0.434689
C	3.653676	1.365037	-0.186442
C	4.794276	-0.732388	-0.521902
C	4.830908	0.653022	-0.381089
H	3.568845	-2.484166	-0.517869
H	5.709969	-1.287186	-0.683811
H	5.776633	1.178706	-0.424838
H	3.701841	2.436048	-0.059534
C	1.560252	3.561570	-1.181734
H	2.574364	3.391256	-1.538359
H	1.084302	4.246953	-1.886190
H	1.622262	4.076222	-0.221555
C	-0.030222	2.889926	1.932723
H	-0.252963	3.654012	1.186718
H	-0.960126	2.393623	2.197397
H	0.322989	3.408299	2.827348
C	2.014209	-2.831208	1.856900
H	2.995204	-2.367793	1.953595
H	1.758408	-3.270172	2.823613
H	2.105514	-3.647532	1.139486
C	-0.035365	-3.599218	-0.848979
H	0.293841	-4.100855	0.060638
H	-1.111069	-3.467154	-0.783228
H	0.147290	-4.288572	-1.676601

40

33 SCF Done: -528.495879614 A.U.

N	-0.223610	-0.199796	-0.036884
C	-1.373907	0.778168	-0.057740
C	-2.759871	0.081473	-0.057068
H	-2.856341	-0.638100	0.756359
H	-3.523624	0.846278	0.087043
H	-2.993991	-0.414470	-0.998890
C	-1.450347	1.715056	1.169886
H	-0.568463	2.328981	1.315368

H	-2.290549	2.397635	1.032295
H	-1.628647	1.145431	2.082145
C	1.095000	0.139858	0.578420
C	1.654951	1.499287	0.065240
H	0.934312	2.292450	0.239016
H	2.514815	1.747337	0.694659
C	2.186087	-0.895755	0.229971
H	2.322588	-1.017849	-0.838457
H	3.130204	-0.536411	0.642142
H	1.995906	-1.870998	0.669469
C	1.052702	0.171698	2.132103
H	0.555327	-0.720135	2.512233
H	2.068220	0.189300	2.535197
H	0.536612	1.040952	2.527091
C	-1.330869	1.637512	-1.341775
H	-0.479741	2.313796	-1.357711
H	-1.266159	0.989485	-2.217417
H	-2.236754	2.243274	-1.431079
C	-0.550772	-1.590477	-0.397221
C	-0.628377	-2.584156	0.778349
H	-1.056175	-3.530553	0.435787
H	0.345897	-2.806071	1.211929
H	-1.265711	-2.191615	1.572050
C	0.210465	-2.209020	-1.592853
H	-0.421398	-2.975932	-2.049061
H	0.417044	-1.449392	-2.347659
H	1.146985	-2.690787	-1.320736
H	-1.560052	-1.540722	-0.775476
C	2.087196	1.527416	-1.402973
H	2.203565	2.556645	-1.748862
H	3.041248	1.022129	-1.556636
H	1.346603	1.041554	-2.038647

43

34 SCF Done: -567.821015876 A.U.

N	0.016001	0.181706	-0.017470
C	1.016455	-0.886610	0.392968
C	2.497077	-0.388048	0.400986
H	2.597485	0.473676	1.063515
H	3.063817	-1.181789	0.891966
C	0.786100	-1.427658	1.822285
H	-0.163133	-1.945187	1.923720
H	1.567187	-2.147550	2.073852
H	0.817999	-0.620424	2.555117
C	-1.449229	0.020827	0.281983
C	-2.014750	-1.359575	-0.150953
H	-1.486060	-2.155789	0.366198
H	-3.036631	-1.403567	0.236613
C	-2.280351	1.058196	-0.504186
H	-2.051509	1.043199	-1.565761
H	-3.336610	0.811194	-0.386349
H	-2.147480	2.071056	-0.134377
C	-1.840232	0.204042	1.779987

H	-1.323743	1.042762	2.235843
H	-2.913480	0.394055	1.854310
H	-1.632658	-0.680417	2.376069
C	1.005830	-2.084421	-0.586964
H	0.116894	-2.699534	-0.501546
H	1.081547	-1.740525	-1.616832
H	1.861365	-2.732290	-0.381056
C	0.543254	1.560573	-0.154523
C	0.301389	2.547160	1.004550
H	0.892700	3.449719	0.828095
H	-0.737575	2.856841	1.105898
H	0.624337	2.118160	1.954630
C	0.280347	2.243175	-1.516104
H	1.066448	2.981886	-1.695286
H	0.317098	1.508267	-2.320973
H	-0.669896	2.769181	-1.574274
H	1.611783	1.446830	-0.155727
C	-2.051885	-1.633643	-1.655944
H	-2.198169	-2.698095	-1.850586
H	-2.869692	-1.097767	-2.139180
H	-1.125026	-1.327836	-2.138758
C	3.180813	-0.124592	-0.948004
H	4.142082	0.368142	-0.787678
H	3.377730	-1.057414	-1.476649
H	2.592273	0.504720	-1.615003

46

35 SCF Done: -607.156820970 A.U.

N	-0.777478	0.155450	0.088429
C	0.133936	-0.956767	0.550043
C	1.603903	-0.462150	0.820892
H	1.601458	0.201281	1.686338
H	2.119327	-1.358272	1.178406
C	-0.344982	-1.556422	1.895230
H	-1.361661	-1.938236	1.809024
H	0.297254	-2.384342	2.204122
H	-0.331774	-0.810315	2.691178
C	-2.194150	-0.099005	-0.409347
C	-2.543686	-1.543323	-0.819319
H	-2.470736	-2.250210	0.004058
H	-3.583576	-1.542763	-1.148337
H	-1.948432	-1.900818	-1.653826
C	-2.389178	0.735424	-1.694413
H	-1.675498	0.407756	-2.451982
H	-3.398915	0.595695	-2.089661
H	-2.240343	1.797744	-1.534055
C	-3.275004	0.281038	0.632440
H	-3.271408	1.334334	0.900804
H	-4.261258	0.061288	0.221295
H	-3.157680	-0.303480	1.546490
C	0.251784	-2.112438	-0.467934
H	-0.528438	-2.855792	-0.350611
H	0.233946	-1.750010	-1.492661

H	1.196286	-2.630281	-0.312019
C	-0.706608	1.316396	0.997331
C	-1.009187	2.701114	0.416331
H	-0.693106	3.452821	1.144348
H	-0.460211	2.876195	-0.507817
H	-2.066291	2.870663	0.220655
H	0.307453	1.374372	1.365776
H	-1.325343	1.179697	1.893991
C	2.553380	0.181030	-0.234237
C	3.846046	0.534086	0.529675
H	4.306294	-0.357838	0.962867
H	4.574860	0.995278	-0.141236
H	3.645532	1.237868	1.341228
C	1.996655	1.468419	-0.854635
H	1.053604	1.280301	-1.363653
H	1.824853	2.233706	-0.096231
H	2.712632	1.874282	-1.574497
C	2.941457	-0.777176	-1.373611
H	3.294910	-1.736542	-0.989264
H	2.112104	-0.967092	-2.052167
H	3.750314	-0.336792	-1.962250

49

36 SCF Done: -646.477918757 A.U.

N	-0.778706	0.032676	0.021475
C	0.214856	-0.874048	0.697447
C	1.627513	-0.217670	0.886769
H	1.514842	0.676763	1.498657
H	2.158413	-0.914021	1.541872
C	-0.219222	-1.265382	2.139752
H	-1.270814	-1.539426	2.177477
H	0.365265	-2.116134	2.499025
H	-0.064883	-0.445592	2.840322
C	-2.118385	-0.444408	-0.489207
C	-2.434625	-1.949575	-0.319330
H	-2.312693	-2.308047	0.699082
H	-3.482822	-2.089963	-0.584529
H	-1.857797	-2.577547	-0.990481
C	-2.200756	-0.201983	-2.015886
H	-1.353658	-0.682223	-2.507616
H	-3.120865	-0.633260	-2.419303
H	-2.190823	0.850921	-2.277374
C	-3.305056	0.264955	0.204156
H	-3.301157	1.341105	0.070467
H	-4.241290	-0.104121	-0.218224
H	-3.309408	0.051127	1.273866
C	0.456487	-2.187567	-0.082485
H	-0.289085	-2.944083	0.127032
H	0.489443	-2.017828	-1.157536
H	1.410143	-2.610506	0.226643
C	-0.634136	1.472212	0.336468
C	-1.029992	2.456166	-0.776804
H	-0.656084	3.445473	-0.502056

H	-0.579771	2.181240	-1.728577
H	-2.104849	2.551353	-0.919547
H	0.433014	1.621527	0.414786
C	2.625970	0.108896	-0.274416
C	3.646291	1.104133	0.311058
H	4.153100	0.680858	1.182458
H	4.408155	1.359312	-0.429633
H	3.157447	2.030335	0.623087
C	1.985994	0.743589	-1.518316
H	1.146262	0.155071	-1.883435
H	1.623172	1.750562	-1.317774
H	2.731009	0.821047	-2.314602
C	3.414157	-1.136736	-0.728462
H	3.819507	-1.685218	0.125374
H	2.805797	-1.822703	-1.314701
H	4.254425	-0.831153	-1.357022
C	-1.186953	1.988931	1.690053
H	-0.698124	2.937498	1.930006
H	-2.259354	2.172717	1.675192
H	-0.978439	1.297112	2.501956

46

37 SCF Done: -682.225422523 A.U.

N	1.304677	-0.049627	-0.155352
C	1.993772	-1.352315	-0.099180
C	3.007563	-1.509351	-1.237206
H	3.884410	-0.870864	-1.115520
H	3.362135	-2.541697	-1.273581
H	2.541807	-1.269174	-2.194109
H	1.237172	-2.108107	-0.293129
C	2.624050	-1.685746	1.263276
H	1.886165	-1.609138	2.064270
H	3.018933	-2.704428	1.264940
H	3.449144	-1.009920	1.498489
C	2.232063	1.098720	0.016915
C	0.021079	0.038292	0.567563
H	0.196179	0.207218	1.641298
C	-0.852178	-1.239716	0.472277
C	-0.848269	1.218201	0.051879
H	-0.296524	-2.100677	0.844650
C	-1.308252	-1.492446	-0.974987
C	-2.092112	-1.072930	1.369602
H	-0.275866	2.141883	0.098128
C	-1.293000	0.968607	-1.397313
C	-2.086395	1.379289	0.949270
H	-1.895777	-2.415156	-1.016781
H	-0.445635	-1.626149	-1.631269
C	-2.151145	-0.303896	-1.464197
H	-1.784703	-0.916525	2.408236
H	-2.678676	-1.996589	1.344715
C	-2.943321	0.108505	0.887452
H	-0.417479	0.863064	-2.039767
H	-1.865413	1.829429	-1.757666

H	-1.779744	1.576568	1.981581
H	-2.667282	2.244995	0.616333
H	-2.469431	-0.480473	-2.495830
C	-3.387592	-0.141029	-0.563796
H	-3.822117	0.219365	1.529434
H	-3.999626	0.695301	-0.916487
H	-4.009892	-1.040321	-0.614832
H	3.235375	0.677550	-0.031804
C	2.176296	1.819577	1.373632
H	1.250305	2.375233	1.522774
H	2.286717	1.117403	2.200675
H	2.999370	2.534899	1.431005
C	2.155964	2.089396	-1.152560
H	1.213980	2.636273	-1.174865
H	2.965300	2.821207	-1.080045
H	2.257281	1.557274	-2.099142

61

38 SCF Done: -953.896448974 A.U.

N	0.076433	0.932460	-0.036276
C	-0.149661	2.391193	-0.070668
C	-0.937918	2.912786	-1.274827
H	-2.004655	2.713777	-1.213489
H	-0.816512	3.996990	-1.339904
H	-0.558709	2.469388	-2.196515
H	0.849983	2.795035	-0.226933
C	-0.621038	3.044166	1.245054
H	-0.064525	2.646310	2.095704
H	-0.439724	4.121213	1.202548
H	-1.681736	2.900323	1.436117
C	1.465858	0.682896	0.441847
H	1.675386	1.414212	1.235979
C	2.504579	0.940147	-0.687277
C	1.812533	-0.688118	1.073466
H	2.262639	1.877938	-1.191116
C	2.485555	-0.196009	-1.719417
C	3.906711	1.053706	-0.066242
H	1.105443	-0.919183	1.873401
C	1.846711	-1.835422	0.044911
C	3.217293	-0.572893	1.704890
H	3.180814	0.035323	-2.532572
H	1.488152	-0.275257	-2.158583
C	2.877052	-1.521510	-1.048619
H	3.931795	1.877043	0.654865
H	4.641388	1.285016	-0.843793
C	4.268308	-0.268241	0.625681
H	0.871252	-1.991832	-0.412572
H	2.109544	-2.766858	0.556442
H	3.224020	0.210335	2.468563
H	3.460774	-1.513681	2.208078
H	2.878227	-2.325516	-1.790361
C	4.273738	-1.397098	-0.418570
H	5.256077	-0.188398	1.088856

H	4.556259	-2.343924	0.052772
H	5.019000	-1.186286	-1.192145
C	-0.954027	-0.086150	0.245407
H	-0.408599	-0.862080	0.764927
C	-1.537131	-0.814869	-1.001905
C	-2.140527	0.201342	1.211619
H	-0.728942	-0.955506	-1.722655
C	-2.696990	-0.060613	-1.666743
C	-2.064791	-2.187010	-0.532522
H	-1.777504	0.757292	2.076696
C	-3.315011	0.943811	0.550765
C	-2.669048	-1.171437	1.688428
H	-3.063682	-0.642296	-2.519211
H	-2.359208	0.893498	-2.065360
C	-3.837470	0.146133	-0.655268
H	-1.255833	-2.773968	-0.087282
H	-2.438047	-2.756700	-1.389464
C	-3.193507	-1.986204	0.492913
H	-3.028985	1.944455	0.237095
H	-4.117343	1.068939	1.285579
H	-1.874538	-1.722537	2.200874
H	-3.474247	-1.022032	2.414910
H	-4.654224	0.697793	-1.130008
C	-4.347740	-1.221174	-0.174152
H	-3.549328	-2.959658	0.843265
H	-5.170310	-1.086829	0.535624
H	-4.740010	-1.796158	-1.019009

68
39 SCF Done: -1070.68189454 A.U.

N	0.094691	0.430284	-0.005380
C	1.512921	0.411363	0.451420
H	1.609264	1.167811	1.243610
C	2.479402	0.837227	-0.690638
C	2.089948	-0.882401	1.077077
H	2.076102	1.720586	-1.189564
C	2.638434	-0.287155	-1.723202
C	3.849895	1.184792	-0.086339
H	1.439948	-1.227345	1.884490
C	2.304289	-2.008797	0.047499
C	3.463402	-0.533287	1.691675
H	3.275620	0.056035	-2.544405
H	1.663423	-0.532604	-2.150784
C	3.254021	-1.527781	-1.058125
H	3.745192	2.001526	0.635151
H	4.526524	1.534988	-0.872092
C	4.435640	-0.057442	0.600073
H	1.363941	-2.328169	-0.398155
H	2.726309	-2.882031	0.555177
H	3.348264	0.240626	2.456190
H	3.867360	-1.419613	2.190506
H	3.380823	-2.320989	-1.800685
C	4.617812	-1.170297	-0.445363

H	5.401443	0.187084	1.051806
H	5.061226	-2.055798	0.021245
H	5.307395	-0.838067	-1.227932
C	-0.747264	-0.750540	0.265686
H	-0.080729	-1.425305	0.786035
C	-1.191805	-1.559416	-0.989275
C	-1.971266	-0.673842	1.224755
H	-0.367081	-1.557948	-1.704809
C	-2.455910	-1.002341	-1.658631
C	-1.486389	-3.003492	-0.532849
H	-1.711101	-0.071955	2.096241
C	-3.249812	-0.133533	0.560286
C	-2.265744	-2.118966	1.688831
H	-2.714553	-1.628330	-2.519288
H	-2.277531	-0.001181	-2.045742
C	-3.622405	-0.997336	-0.655583
H	-0.594004	-3.450577	-0.084608
H	-1.753264	-3.621045	-1.396363
C	-2.639634	-3.001010	0.484675
H	-3.133499	0.903601	0.256697
H	-4.066499	-0.151534	1.289768
H	-1.393417	-2.533678	2.203256
H	-3.088875	-2.111396	2.410503
H	-4.516367	-0.586045	-1.133635
C	-3.900512	-2.434416	-0.187709
H	-2.830780	-4.022633	0.826353
H	-4.739336	-2.444826	0.515654
H	-4.184581	-3.060552	-1.039486
C	-0.367401	1.829524	-0.029978
C	-1.228263	2.243087	-1.230998
C	-0.941919	2.402238	1.284830
H	0.553572	2.396783	-0.180389
C	-1.358234	3.769755	-1.302908
H	-2.227458	1.814610	-1.168456
H	-0.770448	1.854242	-2.143565
C	-1.060611	3.928394	1.202085
H	-1.928170	1.989702	1.491837
H	-0.295360	2.115170	2.118436
C	-1.911219	4.351426	0.001243
H	-1.998186	4.049282	-2.144195
H	-0.372521	4.207095	-1.500487
H	-1.487849	4.323285	2.127994
H	-0.060137	4.366382	1.106179
H	-1.964156	5.441705	-0.062593
H	-2.937607	3.994593	0.146543

53

40 SCF Done: -799.005825647 A.U.

N	0.513493	0.582188	0.198445
C	0.692281	2.036520	0.341199
C	1.713863	2.412023	1.416720
H	2.736212	2.168690	1.129702
H	1.671814	3.489796	1.589029

H	1.490573	1.905334	2.356289
H	-0.256916	2.398414	0.730059
C	0.946426	2.843495	-0.953312
H	0.677708	3.890047	-0.787541
H	1.989217	2.817567	-1.264738
H	0.339900	2.472125	-1.780435
C	-0.682227	0.146045	-0.557407
H	-0.441671	0.106328	-1.634560
C	-1.921149	1.069009	-0.416836
C	-1.141894	-1.281800	-0.137853
H	-1.665335	2.089016	-0.701917
C	-2.479145	1.056660	1.018648
C	-3.019708	0.578871	-1.379622
H	-0.304117	-1.976467	-0.198387
C	-1.680401	-1.284079	1.300674
C	-2.237785	-1.769859	-1.099731
H	-3.332863	1.739183	1.078990
H	-1.730893	1.413078	1.729537
C	-2.907993	-0.367444	1.398635
H	-2.653106	0.603599	-2.410394
H	-3.873207	1.261837	-1.326740
C	-3.457855	-0.844238	-1.008683
H	-0.901188	-0.942379	1.984986
H	-1.946696	-2.306195	1.588105
H	-1.856072	-1.790879	-2.125489
H	-2.520522	-2.795075	-0.841456
H	-3.294879	-0.373754	2.421876
C	-3.999557	-0.854621	0.430837
H	-4.235746	-1.183653	-1.698862
H	-4.317807	-1.866077	0.702928
H	-4.880257	-0.208458	0.504075
C	1.689387	-0.270771	-0.079705
C	2.749234	0.262314	-1.063351
C	2.357831	-0.801167	1.200787
H	1.293119	-1.155636	-0.578499
C	3.791119	-0.818465	-1.377344
H	3.263045	1.129392	-0.643803
H	2.264635	0.590346	-1.984992
C	3.406270	-1.871858	0.882084
H	2.836386	0.016174	1.743574
H	1.582928	-1.204729	1.856152
C	4.453641	-1.350211	-0.104480
H	4.545677	-0.418858	-2.060456
H	3.303131	-1.648718	-1.901178
H	3.886267	-2.213796	1.803088
H	2.908317	-2.745994	0.445668
H	5.171954	-2.137070	-0.350430
H	5.021923	-0.541058	0.369139

40

41 SCF Done: -1063.16516181 A.U.

N	1.292644	0.229449	-0.335611
C	2.529691	-0.573332	-0.000471

C	3.685241	-0.076425	-0.880034
H	4.021229	0.917477	-0.592317
H	4.532201	-0.757267	-0.784220
H	3.377853	-0.049733	-1.926439
C	2.925548	-0.496529	1.482647
H	2.139887	-0.883905	2.132847
H	3.821892	-1.094312	1.654581
H	3.141223	0.529687	1.777768
C	0.030124	-0.257847	0.314690
H	0.319643	-0.935749	1.121093
C	-0.796438	-1.065205	-0.720461
C	-0.915256	0.775769	0.971355
H	-0.145340	-1.761047	-1.244432
C	-1.455568	-0.131165	-1.745492
C	-1.876989	-1.854525	0.039324
H	-0.349918	1.379691	1.681392
C	-1.622060	1.683632	-0.052371
C	-1.995371	-0.018454	1.736911
H	-1.988236	-0.731190	-2.489482
H	-0.685170	0.434811	-2.275440
C	-2.425671	0.827949	-1.039693
H	-1.410522	-2.535620	0.758387
H	-2.442381	-2.471278	-0.665527
C	-2.816660	-0.878543	0.762072
H	-0.903255	2.296179	-0.594333
H	-2.285923	2.368402	0.484751
H	-1.531488	-0.651804	2.499032
H	-2.650473	0.684363	2.259829
H	-2.906599	1.475206	-1.778464
C	-3.495295	0.027851	-0.278583
H	-3.575142	-1.437540	1.317207
H	-4.190919	0.712586	0.216548
H	-4.081014	-0.579212	-0.976115
Cl	1.637858	1.952245	-0.003242
C	2.266832	-2.040764	-0.381103
H	1.481318	-2.499713	0.216952
H	2.007974	-2.132037	-1.435773
H	3.182295	-2.606771	-0.206187

49

42 SCF Done: -721.536758970 A.U.

N	1.259860	0.146096	-0.232535
C	2.210747	-1.030698	-0.048857
C	3.684933	-0.663376	-0.331616
H	4.115157	-0.003201	0.421774
H	4.269104	-1.584008	-0.317375
H	3.813337	-0.212088	-1.316984
C	2.198018	-1.620812	1.380320
H	1.233023	-2.026601	1.673983
H	2.924073	-2.433481	1.446417
H	2.479666	-0.864288	2.113709
C	-0.040523	0.032494	0.493337
H	0.156690	0.050038	1.575293

C	-0.857197	-1.265243	0.222975
C	-1.027317	1.205756	0.221487
H	-0.241662	-2.141852	0.389507
C	-1.421098	-1.304109	-1.206812
C	-2.023936	-1.346600	1.225730
H	-0.533679	2.156282	0.385585
C	-1.592294	1.166561	-1.207760
C	-2.194471	1.128834	1.224027
H	-1.938374	-2.256052	-1.365256
H	-0.611424	-1.248366	-1.935315
C	-2.384208	-0.129418	-1.419294
H	-1.638171	-1.346518	2.250173
H	-2.551863	-2.294609	1.082733
C	-2.982814	-0.169174	1.021310
H	-0.784781	1.223137	-1.937637
H	-2.235937	2.038256	-1.365368
H	-1.812808	1.183302	2.248496
H	-2.847090	1.995480	1.079098
H	-2.781927	-0.157575	-2.438070
C	-3.541667	-0.209183	-0.410682
H	-3.804151	-0.225356	1.741792
H	-4.232385	0.625822	-0.566364
H	-4.110572	-1.131762	-0.564778
C	1.896165	-2.127667	-1.091327
H	0.920168	-2.582444	-0.974105
H	1.949518	-1.701230	-2.094075
H	2.635132	-2.928928	-1.019635
C	1.914136	1.479884	-0.101856
H	2.949199	1.314741	-0.356528
C	1.956138	2.083117	1.314826
H	0.976675	2.372967	1.693806
H	2.392275	1.379454	2.025057
H	2.582168	2.978472	1.308623
C	1.487453	2.512919	-1.160105
H	0.519208	2.975220	-0.984561
H	2.229192	3.316089	-1.180613
H	1.468815	2.046721	-2.145858

47

43 SCF Done: -720.319430687 A.U.

N	1.218997	0.157672	-0.142479
C	2.209415	-0.983647	-0.015923
C	3.618762	-0.517469	-0.433573
H	4.000875	0.280010	0.199582
H	4.301092	-1.364409	-0.354176
H	3.632936	-0.180365	-1.469973
C	2.307588	-1.525404	1.422533
H	1.354807	-1.904286	1.789797
H	3.025919	-2.346737	1.460928
H	2.652718	-0.749532	2.106682
C	1.819114	1.456516	0.103934
C	2.087011	2.269574	-1.130896
C	2.061520	1.938039	1.322004

H	1.140123	2.521955	-1.615171
H	1.830149	1.382002	2.219920
C	-0.094357	0.051148	0.558593
H	0.071082	0.089599	1.644745
C	-0.918093	-1.233553	0.291643
C	-1.014850	1.252348	0.199302
H	-0.325560	-2.113924	0.525830
C	-1.418043	-1.309582	-1.160688
C	-2.134741	-1.235917	1.239246
H	-0.469112	2.182543	0.346665
C	-1.501936	1.170944	-1.256848
C	-2.230892	1.251988	1.141360
H	-1.965981	-2.246292	-1.305261
H	-0.578880	-1.314553	-1.855612
C	-2.324478	-0.108378	-1.462453
H	-1.799026	-1.209041	2.280612
H	-2.689316	-2.169806	1.105913
C	-3.042834	-0.034986	0.944710
H	-0.650516	1.173949	-1.939583
H	-2.107361	2.054017	-1.484840
H	-1.900320	1.335425	2.181406
H	-2.853388	2.127341	0.931575
H	-2.672446	-0.162092	-2.498216
C	-3.532245	-0.111391	-0.511120
H	-3.899955	-0.042760	1.624401
H	-4.185598	0.738938	-0.731307
H	-4.124042	-1.020297	-0.660028
C	1.863074	-2.122580	-0.994522
H	0.953625	-2.656755	-0.741699
H	1.759192	-1.725060	-2.004612
H	2.674674	-2.853130	-0.997533
H	2.483514	2.926158	1.454979
H	2.617330	3.194834	-0.901639
H	2.666396	1.702671	-1.859839

4

NH3 SCF Done: -56.5901966466 A.U.

N	0.000000	0.000000	0.114997
H	0.000000	0.939606	-0.268327
H	-0.813723	-0.469803	-0.268327
H	0.813723	-0.469803	-0.268327

13

NMe3 SCF Done: -174.552269352 A.U.

N	0.000008	-0.000061	-0.377391
C	-0.049326	1.386625	0.060850
H	0.815954	1.929551	-0.323414
H	-0.052622	1.484367	1.161584
C	1.225596	-0.650596	0.060876
H	1.263181	-1.671380	-0.323405
H	1.312082	-0.696487	1.161584
C	-1.176283	-0.736005	0.060877
H	-1.259374	-0.787760	1.161591
H	-2.079006	-0.258030	-0.323526

H -1.140962 -1.757308 -0.322697
H -0.951521 1.866579 -0.322730
H 2.092288 -0.109249 -0.322871
22
NEt3 SCF Done: -292.546375710 A.U.
N 0.000180 -0.000030 0.009335
C -0.375495 1.350561 0.438160
H 0.511322 1.982806 0.385604
H -0.689825 1.350721 1.496706
C -1.461853 1.975845 -0.430040
H -2.387683 1.398618 -0.408877
H -1.693884 2.983668 -0.078483
H -1.127826 2.038681 -1.467179
C 1.357629 -0.349615 0.438716
H 1.461624 -1.433834 0.387725
H 1.514941 -0.075961 1.496851
C 2.442329 0.277142 -0.430464
H 2.404582 1.367574 -0.411948
H 3.431233 -0.024404 -0.077952
H 2.330211 -0.046101 -1.466841
C -0.981266 -1.000689 0.439067
H -0.822307 -1.274058 1.497025
H -1.972212 -0.548573 0.388850
C -0.981426 -2.253226 -0.430434
H -0.018262 -2.765892 -0.412434
H -1.736951 -2.958850 -0.077718
H -1.205731 -1.994291 -1.466699
12
Py SCF Done: -248.38765066 A.U.
N -0.000131 -1.414139 -0.000093
C -1.141566 -0.719447 -0.000216
C -1.194357 0.670161 -0.000122
C 0.000130 1.379561 0.000096
C 1.194482 0.669951 0.000212
C 1.141433 -0.719646 0.000125
H 0.000199 2.462128 0.000172
H -2.057474 -1.300931 -0.000373
H -2.149543 1.178007 -0.000241
H 2.149781 1.177586 0.000348
H 2.057227 -1.301305 0.000184
H -0.000219 -2.414138 -0.000161
29
1H+ SCF Done: -371.659699457 A.U.
N 0.019883 0.199607 0.205971
C 0.006493 1.503081 -0.566761
C 1.217453 2.381280 -0.312685
H -0.898553 2.028131 -0.273422
H -0.078574 1.238619 -1.617711
H 1.064471 3.321795 -0.841778
H 1.333358 2.611705 0.747401
H 2.141351 1.941700 -0.683948
C 1.209634 -0.711176 -0.228577

C	0.844897	-2.171654	-0.452319
H	1.540092	-0.286619	-1.174431
H	1.743378	-2.668390	-0.819292
H	0.552922	-2.669607	0.472214
H	0.068751	-2.314236	-1.199805
C	-1.376397	-0.449213	0.265159
C	-2.217390	0.260983	1.318415
H	-1.190189	-1.460146	0.613441
H	-3.166816	-0.265699	1.411579
H	-1.733273	0.245330	2.296597
H	-2.439064	1.293792	1.049389
C	-2.054682	-0.492097	-1.096269
H	-2.917009	-1.153849	-1.021464
H	-2.416526	0.489342	-1.400508
H	-1.405544	-0.879645	-1.879461
C	2.311821	-0.587422	0.817863
H	2.610572	0.443685	0.995853
H	1.992941	-1.030485	1.764314
H	3.189863	-1.133094	0.474340
H	0.217699	0.447729	1.175933

18

2H+ SCF Done: -252.468169751 A.U.

C	-1.485419	0.080352	0.225356
C	-0.676656	1.297822	-0.228934
C	0.765435	1.220102	0.245093
C	0.628231	-1.294508	0.243419
C	-0.814834	-1.218786	-0.227930
H	1.377861	2.030645	-0.144076
H	-0.691484	1.372922	-1.320196
H	-1.108189	2.221808	0.157036
H	-1.570987	0.085107	1.316001
H	-2.497683	0.135998	-0.175399
H	1.151091	-2.165746	-0.144337
H	0.697268	-1.294008	1.330494
H	-0.844535	-1.298681	-1.318693
H	-1.341319	-2.088914	0.165531
H	0.836531	1.213280	1.332072
N	1.387603	-0.074401	-0.226123
H	1.421528	-0.073454	-1.248447
H	2.356158	-0.128051	0.090846

30

3H+ SCF Done: -409.800943853 A.U.

C	-0.000050	1.952786	0.074589
C	1.256021	1.250608	-0.438569
C	1.348223	-0.224413	-0.031686
C	-1.348256	-0.224409	-0.031680
C	-1.256047	1.250563	-0.438673
H	1.279340	1.318881	-1.530440
H	2.156851	1.746108	-0.074378
H	-0.000104	2.000894	1.164782
H	-0.000049	2.985623	-0.275190
H	-1.279244	1.318736	-1.530557

H	-2.156928	1.746074	-0.074616
N	0.000026	-0.887112	-0.397451
H	-0.000014	-0.987460	-1.414439
C	-1.641087	-0.413427	1.455498
H	-2.675682	-0.123392	1.634507
H	-1.534378	-1.457638	1.753568
H	-1.014989	0.197309	2.097747
C	-2.401483	-0.962268	-0.859643
H	-2.193908	-0.896667	-1.929225
H	-2.467441	-2.014552	-0.576671
H	-3.374392	-0.505362	-0.682668
C	2.401535	-0.962148	-0.859663
H	2.467582	-2.014430	-0.576703
H	2.193962	-0.896556	-1.929233
H	3.374405	-0.505153	-0.682658
C	1.641100	-0.413526	1.455472
H	1.015113	0.197277	2.097754
H	1.534204	-1.457723	1.753484
H	2.675745	-0.123671	1.634459
H	0.000012	-1.841115	-0.031240

21

4H+ SCF Done: -291.795089078 A.U.

C	-1.909491	0.000012	0.269147
C	-1.184917	1.255652	-0.216099
C	0.277891	1.249935	0.197697
C	0.277911	-1.249945	0.197652
C	-1.184931	-1.255655	-0.216057
H	0.823523	2.098393	-0.210614
H	-1.254766	1.336399	-1.304902
H	-1.641473	2.156860	0.195077
H	-1.946378	0.000042	1.362493
H	-2.939807	0.000003	-0.086928
H	0.823497	-2.098392	-0.210751
H	0.388818	-1.251388	1.281997
H	-1.254856	-1.336462	-1.304846
H	-1.641453	-2.156831	0.195227
H	0.388737	1.251282	1.282049
N	0.974834	-0.000004	-0.293513
C	2.427377	0.000011	0.058281
H	2.889725	-0.891027	-0.357411
H	2.889701	0.891054	-0.357424
H	2.520747	0.000021	1.141017
H	0.907112	0.000013	-1.314122

33

5H+ SCF Done: -449.11643974 A.U.

C	-0.000005	2.166809	0.048870
C	1.243150	1.451787	-0.458769
C	1.353189	-0.015758	-0.021080
C	-1.353189	-0.015765	-0.021080
C	-1.243155	1.451780	-0.458771
H	1.257299	1.496323	-1.552407
H	2.149411	1.953871	-0.118253

H	-0.000006	2.227879	1.138467
H	-0.000007	3.195008	-0.315106
H	-1.257300	1.496312	-1.552409
H	-2.149419	1.953861	-0.118260
N	0.000001	-0.727700	-0.392193
C	0.000010	-2.184251	-0.037571
H	-0.880638	-2.654202	-0.457712
H	0.880693	-2.654179	-0.457666
H	-0.000015	-2.298493	1.038698
H	0.000001	-0.693584	-1.413536
C	2.458706	-0.691213	-0.842164
H	2.188677	-0.781784	-1.896368
H	3.348044	-0.064380	-0.786602
H	2.730898	-1.672967	-0.459713
C	1.673501	-0.143546	1.466259
H	2.670510	0.269238	1.618681
H	0.991930	0.410068	2.103528
H	1.701592	-1.179814	1.797868
C	-2.458707	-0.691219	-0.842164
H	-3.348045	-0.064386	-0.786599
H	-2.188679	-0.781784	-1.896369
H	-2.730897	-1.672974	-0.459716
C	-1.673504	-0.143554	1.466259
H	-0.991907	0.410020	2.103532
H	-2.670494	0.269273	1.618687
H	-1.701642	-1.179824	1.797856

39

6H+ SCF Done: -602.790039580 A.U.

N	-0.544887	-0.005951	-0.372476
C	-1.204134	-1.419032	0.014719
C	-0.483325	-2.449315	-0.861224
H	-0.941652	-3.421453	-0.683637
H	-0.590522	-2.223438	-1.923533
H	0.574257	-2.529960	-0.621273
C	-1.236020	1.409114	-0.020189
C	-2.395582	1.626203	-0.998851
H	-2.715788	2.663122	-0.905863
H	-2.085709	1.478709	-2.035473
H	-3.257941	1.003091	-0.795405
C	-1.714617	1.465680	1.422678
H	-2.130429	2.459828	1.588179
H	-2.502173	0.747348	1.632322
H	-0.904722	1.332188	2.136295
C	0.919577	-0.006122	-0.157190
C	1.735796	-0.055034	-1.279377
C	1.469878	0.070086	1.115197
C	3.116701	-0.045674	-1.128953
H	1.304475	-0.098145	-2.272207
C	2.851793	0.080963	1.257670
H	0.846405	0.136785	1.991212
C	3.677422	0.019024	0.140769
H	3.748336	-0.086796	-2.005903

H	3.279665	0.142262	2.249146
H	4.752532	0.026716	0.259936
C	-1.012828	-1.773749	1.485653
H	-1.425079	-1.033871	2.164271
H	-1.547495	-2.707850	1.658030
H	0.029969	-1.950929	1.733959
C	-2.686130	-1.413328	-0.350132
H	-2.852125	-1.137980	-1.390808
H	-3.056356	-2.429571	-0.219851
H	-3.277991	-0.770289	0.294785
C	-0.193674	2.502829	-0.278803
H	0.634157	2.473632	0.423874
H	0.204624	2.455456	-1.292348
H	-0.695698	3.462600	-0.163038
H	-0.645680	-0.019668	-1.387139

40

7H+ SCF Done: -695.066860902 A.U.

N	-1.056909	0.044707	0.377432
C	-1.598924	1.372644	-0.359400
C	-1.195217	2.531297	0.558102
H	-1.602253	3.449001	0.135665
H	-1.607745	2.420718	1.562430
H	-0.116397	2.650488	0.628714
C	-1.574677	-1.445888	0.009231
C	-2.952427	-1.625720	0.653789
H	-3.229332	-2.674518	0.554181
H	-2.929049	-1.400401	1.722468
H	-3.733352	-1.040353	0.183974
C	-1.625555	-1.675385	-1.493056
H	-1.938271	-2.707644	-1.651160
H	-2.348696	-1.037809	-1.993578
H	-0.653581	-1.560366	-1.967623
C	0.399805	0.109241	0.605865
C	1.297390	-0.081113	-0.430986
C	0.851762	0.341269	1.898967
C	2.666240	-0.020342	-0.160454
H	0.967335	-0.289901	-1.434365
C	2.214545	0.401700	2.159699
H	0.148099	0.474905	2.710653
C	3.129871	0.225331	1.134796
H	4.191956	0.271066	1.328667
H	2.558824	0.585408	3.167571
C	-0.981000	1.569282	-1.739237
H	-1.155826	0.736620	-2.412925
H	-1.458004	2.446578	-2.176108
H	0.084032	1.780054	-1.690531
C	-3.120525	1.318352	-0.451567
H	-3.586705	1.161454	0.519907
H	-3.457035	2.286719	-0.820046
H	-3.474865	0.566892	-1.150865
C	-0.613904	-2.440239	0.669658
H	0.370851	-2.451061	0.210698

H	-0.503058	-2.254128	1.737905
H	-1.044661	-3.433327	0.549388
C	3.596844	-0.217560	-1.228171
N	4.345302	-0.375839	-2.089272
H	-1.466389	0.160319	1.304453

40

8H+ SCF Done: -695.065731934 A.U.

N	-0.620871	0.039148	0.145403
C	-1.313036	1.348088	-0.501719
C	-0.472289	2.529262	-0.007584
H	-0.939702	3.444343	-0.368639
H	-0.440944	2.582845	1.081253
H	0.544702	2.506216	-0.393686
C	-1.359805	-1.403159	0.240336
C	-2.383394	-1.319303	1.377423
H	-2.706864	-2.334281	1.604022
H	-1.948531	-0.908160	2.290658
H	-3.267615	-0.748938	1.120140
C	-2.019489	-1.792060	-1.072678
H	-2.458138	-2.780432	-0.931759
H	-2.824194	-1.118715	-1.354051
H	-1.309269	-1.870858	-1.892720
C	0.798637	-0.066236	-0.233781
C	1.782759	0.209078	0.729795
C	1.179883	-0.455892	-1.505712
C	3.136349	0.112928	0.395911
C	2.528888	-0.551893	-1.828346
H	0.441569	-0.701733	-2.249905
C	3.506368	-0.262800	-0.884819
H	3.882038	0.331166	1.146906
H	2.809906	-0.859245	-2.826145
H	4.553642	-0.336531	-1.142117
C	-1.317875	1.318093	-2.025400
H	-1.818586	0.447760	-2.437247
H	-1.871277	2.197659	-2.354394
H	-0.318919	1.396955	-2.445550
C	-2.732007	1.482869	0.040212
H	-2.760578	1.474204	1.128838
H	-3.112066	2.449763	-0.287687
H	-3.404097	0.723938	-0.349590
C	-0.293960	-2.432321	0.630609
H	0.435067	-2.609112	-0.155443
H	0.230422	-2.157532	1.545762
H	-0.809402	-3.372785	0.819928
C	1.407411	0.570904	2.057641
N	1.052108	0.849151	3.118448
H	-0.616462	0.300039	1.133153

40

9H+ SCF Done: -695.067218852 A.U.

N	-1.104403	-0.006007	-0.374394
C	-1.749962	-1.423990	0.042340
C	-1.065530	-2.453459	-0.861901

H	-1.512814	-3.426049	-0.660564
H	-1.223182	-2.231688	-1.918491
H	0.001934	-2.534044	-0.669572
C	-1.780003	1.416609	0.004310
C	-2.968404	1.633368	-0.937548
H	-3.289935	2.668094	-0.826704
H	-2.688825	1.495321	-1.983800
H	-3.820971	1.004666	-0.711882
C	-2.211897	1.469105	1.461304
H	-2.624497	2.462225	1.640213
H	-2.990691	0.749089	1.695392
H	-1.380128	1.338753	2.149699
C	0.362555	-0.006899	-0.211830
C	1.143051	-0.061281	-1.359661
C	0.953423	0.070845	1.043088
C	2.524249	-0.057271	-1.259546
H	0.685246	-0.104774	-2.339629
C	2.334053	0.076631	1.149743
H	0.360639	0.142567	1.939184
C	3.124936	0.007637	-0.000427
H	3.131732	-0.101898	-2.151941
H	2.796794	0.138621	2.124255
C	-1.500133	-1.774397	1.504727
H	-1.876575	-1.029008	2.197929
H	-2.036785	-2.702227	1.702384
H	-0.450170	-1.964567	1.710598
C	-3.243809	-1.411602	-0.266888
H	-3.446104	-1.136931	-1.301253
H	-3.611909	-2.426702	-0.122520
H	-3.809349	-0.766475	0.399037
C	-0.742636	2.506516	-0.286355
H	0.105382	2.479331	0.392260
H	-0.375723	2.461146	-1.311901
H	-1.239455	3.466963	-0.155400
C	4.550522	0.009603	0.111295
N	5.698858	0.010495	0.201648
H	-1.248289	-0.022318	-1.383965

55

10H+ SCF Done: -1061.16454338 A.U.

N	-0.222593	0.023084	-0.171138
C	-1.726545	0.207970	0.171976
C	-2.015145	1.212592	1.286664
C	-2.481059	0.555263	-1.111179
H	-2.056889	-0.775533	0.502844
C	-3.526038	1.235104	1.560669
H	-1.703408	2.215283	0.989733
H	-1.477778	0.963282	2.198564
C	-3.988933	0.614277	-0.839104
H	-2.141766	1.522897	-1.491877
H	-2.270243	-0.192819	-1.878603
C	-4.318666	1.580388	0.299513
H	-3.728073	1.958464	2.352074

H	-3.840707	0.257296	1.939361
H	-4.505035	0.905553	-1.755265
H	-4.344459	-0.388649	-0.581896
H	-5.389505	1.558433	0.510413
H	-4.077281	2.603022	-0.009607
C	0.526204	1.384175	-0.294293
C	1.309495	1.820382	0.937638
C	1.370395	1.445732	-1.568534
H	-0.293675	2.084003	-0.432844
C	1.833752	3.248070	0.718882
H	2.153138	1.150423	1.111939
H	0.680851	1.802439	1.825454
C	1.867644	2.881484	-1.774871
H	2.226367	0.778029	-1.502562
H	0.776073	1.126463	-2.428444
C	2.661869	3.365755	-0.560747
H	2.423145	3.542352	1.588677
H	0.982622	3.935160	0.667777
H	2.480382	2.919047	-2.676997
H	1.012958	3.544907	-1.944693
H	2.980599	4.399726	-0.706078
H	3.571901	2.764433	-0.461201
C	0.404336	-1.156434	0.590625
C	0.293835	-1.072482	2.105407
H	-0.745781	-1.051214	2.420668
H	0.743091	-1.969787	2.519955
H	0.818515	-0.214026	2.505188
C	1.856150	-1.349500	0.070374
C	-0.290554	-2.469149	0.108878
O	2.748761	-1.334909	1.038895
O	2.083972	-1.527507	-1.099695
O	-0.271321	-3.473160	0.765415
O	-0.825549	-2.341057	-1.097566
C	4.128311	-1.572069	0.651585
H	4.694696	-1.519107	1.574742
H	4.214663	-2.555700	0.194800
H	4.450129	-0.805282	-0.050163
C	-1.415116	-3.529343	-1.687330
H	-1.803650	-3.207337	-2.647251
H	-0.647596	-4.290150	-1.810336
H	-2.212106	-3.899258	-1.046131
H	-0.239546	-0.324162	-1.133579

52

11H+ SCF Done: -910.412126116 A.U.

N	0.113821	0.976338	-0.288060
C	0.164799	2.525374	-0.223672
C	-0.858858	3.103290	-1.187948
H	-1.878326	2.929333	-0.846143
H	-0.697665	4.179216	-1.249830
H	-0.750355	2.691806	-2.192551
H	1.160354	2.758580	-0.592738
C	0.005857	3.057978	1.188261

H	0.713966	2.619033	1.889375
H	0.195363	4.130865	1.159882
H	-1.006239	2.911433	1.561779
C	-1.233446	0.414548	-0.057929
C	-1.967220	-0.019288	-1.148108
C	-1.758567	0.315808	1.227143
C	-3.240879	-0.547611	-0.974030
H	-1.562531	0.047861	-2.150308
C	-3.023666	-0.207098	1.407364
H	-1.193368	0.631094	2.090968
C	-3.779323	-0.640824	0.310927
H	-3.793062	-0.879965	-1.839452
H	-3.447546	-0.291675	2.398430
O	-5.002337	-1.133191	0.597051
C	-5.830686	-1.593584	-0.472897
H	-6.750941	-1.935079	-0.007523
H	-6.053030	-0.784564	-1.172635
H	-5.359888	-2.423857	-1.004408
C	1.203639	0.245839	0.523802
H	0.920038	0.386961	1.563536
C	2.610506	0.820767	0.288733
C	1.193155	-1.255809	0.179900
H	2.633808	1.884440	0.523166
C	3.072164	0.578884	-1.159415
C	3.567184	0.096651	1.258062
H	0.190008	-1.657856	0.321096
C	1.659383	-1.487894	-1.268560
C	2.161444	-1.962813	1.146284
H	4.073610	0.995929	-1.284897
H	2.436611	1.106030	-1.879794
C	3.078599	-0.929630	-1.458324
H	3.260594	0.278322	2.291968
H	4.567698	0.519042	1.140896
C	3.580999	-1.408727	0.960143
H	0.972430	-1.034521	-1.992112
H	1.639039	-2.559630	-1.477188
H	1.830008	-1.822766	2.178969
H	2.139954	-3.036107	0.944288
H	3.402116	-1.095347	-2.488023
C	4.039418	-1.640179	-0.489427
H	4.263548	-1.910986	1.649131
H	4.063405	-2.711227	-0.707891
H	5.055291	-1.259509	-0.626095
H	0.330040	0.770207	-1.263735

36

12H+ SCF Done: -488.390275302 A.U.

N	0.003217	-0.131738	-0.262099
C	-1.495921	0.382975	-0.009465
C	-2.453836	-0.814570	-0.076067
H	-2.300255	-1.529360	0.726291
H	-3.462137	-0.416535	0.029918
H	-2.405708	-1.330041	-1.033235

C	-1.624489	1.029038	1.365568
H	-1.050816	1.944286	1.468050
H	-2.673811	1.286738	1.509152
H	-1.345720	0.342697	2.164392
C	1.247294	0.855172	-0.016859
C	0.934497	2.285324	-0.456563
H	0.163810	2.764836	0.136651
H	1.846987	2.864057	-0.318837
H	0.671639	2.341676	-1.511294
C	2.403187	0.352664	-0.895347
H	2.087565	0.162672	-1.923039
H	3.158853	1.136033	-0.930689
H	2.878292	-0.535984	-0.495502
C	1.669143	0.825742	1.450787
H	2.521376	1.497170	1.555546
H	0.892792	1.174542	2.125202
H	1.997750	-0.162627	1.763978
C	-1.893120	1.337585	-1.142948
H	-1.461169	2.326205	-1.060117
H	-1.644533	0.924266	-2.122846
H	-2.975212	1.455220	-1.111329
C	0.235031	-1.452438	0.393892
C	-0.192935	-2.744492	-0.232079
C	1.264441	-2.439110	-0.079886
H	0.099460	-1.367337	1.460177
H	-0.582999	-2.725790	-1.240320
H	-0.671740	-3.464472	0.416638
H	1.847791	-2.933977	0.684164
H	1.800248	-2.243880	-0.995403
H	0.025269	-0.295565	-1.269051

35

13H+ SCF Done: -450.308474701 A.U.

N	0.133689	-0.007958	-0.431037
C	-1.285893	-0.607789	-0.025287
C	-1.268984	-2.131392	-0.196469
H	-0.627680	-2.636508	0.522333
H	-2.285512	-2.483494	-0.025195
H	-0.990588	-2.433309	-1.206217
C	-1.679190	-0.256773	1.405601
H	-1.918743	0.796228	1.519111
H	-2.580314	-0.820666	1.644646
H	-0.918893	-0.529828	2.132416
C	0.512326	1.453495	-0.052203
C	-0.313704	2.480664	-0.817353
H	-1.306196	2.615656	-0.393168
H	0.204556	3.437166	-0.742688
H	-0.406234	2.238999	-1.876818
C	0.575779	1.797597	1.434437
H	1.256967	2.643578	1.532346
H	-0.387532	2.110689	1.826848
H	0.958271	0.997578	2.056973
C	-2.311035	-0.038949	-1.013962

H	-2.448713	1.032030	-0.913479
H	-2.041813	-0.266295	-2.047634
H	-3.272447	-0.511665	-0.816292
C	1.338994	-0.979185	-0.304127
C	1.783334	-1.269351	1.120223
H	2.455935	-2.126850	1.083466
H	2.338507	-0.440163	1.553068
H	0.959708	-1.529660	1.780444
C	2.502420	-0.519033	-1.179521
H	3.178323	-1.365773	-1.300093
H	2.177466	-0.215792	-2.176727
H	3.075382	0.291539	-0.730913
H	0.962331	-1.894510	-0.743942
H	1.522209	1.514210	-0.444652
H	0.034898	0.056853	-1.444605

38

14H+ SCF Done: -489.625290012 A.U.

N	0.011241	-0.085242	-0.402916
C	-1.576403	0.023263	-0.110200
C	-2.269997	-1.301058	-0.473682
H	-2.000684	-2.132801	0.170638
H	-3.339077	-1.137362	-0.345935
H	-2.111586	-1.579881	-1.515945
C	-1.885254	0.352367	1.349184
H	-1.456867	1.289052	1.685545
H	-2.968065	0.445339	1.435352
H	-1.573754	-0.434063	2.030560
C	0.985569	1.080290	0.101592
C	0.315893	2.458929	0.022253
H	-0.536790	2.582786	0.678208
H	1.063964	3.184474	0.338131
H	0.032487	2.713157	-0.996975
C	2.179249	1.157992	-0.867077
H	1.855805	1.179533	-1.909354
H	2.698955	2.095174	-0.673671
H	2.900317	0.362579	-0.736426
C	1.473228	0.812482	1.522316
H	2.166772	-0.022089	1.562783
H	2.014503	1.695711	1.860298
H	0.662215	0.632534	2.222687
C	-2.164367	1.081014	-1.060133
H	-1.967854	2.101521	-0.760887
H	-1.815853	0.939010	-2.085430
H	-3.245733	0.953741	-1.070785
C	0.524390	-1.558584	-0.275132
C	0.507995	-2.153764	1.128375
H	0.549481	-3.237444	1.010631
H	1.368273	-1.858915	1.720777
H	-0.391232	-1.926712	1.689790
C	1.853266	-1.844289	-0.967833
H	1.900137	-2.925660	-1.103577
H	1.924738	-1.390072	-1.954928

H	2.716061	-1.557790	-0.372061
H	0.028257	0.056999	-1.412351
H	-0.214582	-2.083987	-0.864645

41

15H+ SCF Done: -528.935847633 A.U.

N	-0.000729	-0.000557	-0.335384
C	1.080843	-1.172973	-0.017170
C	2.535765	-0.720043	-0.228197
H	2.867067	0.066893	0.436100
H	3.151115	-1.590571	-0.006187
H	2.736029	-0.442679	-1.260025
C	0.949224	-1.681534	1.418006
H	-0.012716	-2.127182	1.638980
H	1.699632	-2.460068	1.555825
H	1.145779	-0.904175	2.150943
C	-1.557705	-0.348783	-0.018286
C	-1.893925	-1.833524	-0.236747
H	-1.375535	-2.518494	0.420730
H	-2.954769	-1.932508	-0.011804
H	-1.757193	-2.137651	-1.271408
C	-2.440417	0.387022	-1.050918
H	-2.001910	0.360747	-2.049884
H	-3.389399	-0.142266	-1.111022
H	-2.668775	1.411532	-0.796446
C	-1.934924	0.014261	1.417163
H	-1.834141	1.068470	1.643704
H	-2.987049	-0.239213	1.547509
H	-1.368214	-0.553060	2.150183
C	0.891707	-2.302703	-1.053176
H	0.106222	-3.004595	-0.815508
H	0.720772	-1.905997	-2.055404
H	1.819330	-2.870175	-1.095390
C	0.476313	1.521295	-0.018399
C	0.970733	1.664779	1.420588
H	1.270629	2.703820	1.557366
H	0.193376	1.450303	2.148237
H	1.834849	1.053871	1.650376
C	-0.639795	2.555866	-0.240708
H	-0.193471	3.523589	-0.016962
H	-0.971768	2.589573	-1.275336
H	-1.492298	2.451360	0.417442
H	-0.003621	-0.002035	-1.353558
C	1.562297	1.916647	-1.044813
H	2.564608	1.623250	-0.768840
H	1.343355	1.527498	-2.040758
H	1.562493	3.001801	-1.125232

36

16H+ SCF Done: -488.446618442 A.U.

C	-1.440984	0.031609	-0.024783
C	-1.616810	-1.497098	-0.144128
C	-0.374053	-2.112071	0.468842
C	0.779068	-1.195782	0.049547

H	-1.710724	-1.784767	-1.190272
H	-2.529355	-1.811637	0.358402
H	-0.443464	-2.135774	1.559010
H	-0.192543	-3.131519	0.133493
N	0.118170	0.204274	0.276580
C	-2.205429	0.562054	1.187348
H	-2.028654	1.618693	1.371066
H	-3.271787	0.427569	1.008738
H	-1.949667	0.004218	2.090562
C	-1.872917	0.713463	-1.317662
H	-2.905403	0.414826	-1.501529
H	-1.852421	1.798593	-1.272356
H	-1.284266	0.383822	-2.172432
C	1.976990	-1.419445	0.971119
H	2.160173	-2.493550	1.013524
H	2.889081	-0.945240	0.625543
H	1.769241	-1.084893	1.989207
C	1.144758	-1.387887	-1.419636
H	1.922812	-0.703652	-1.747698
H	1.526839	-2.401376	-1.540261
H	0.289266	-1.275513	-2.081878
C	0.785499	1.457096	-0.306900
H	0.586036	1.420615	-1.374349
C	0.175146	2.716921	0.308541
H	0.680672	3.581174	-0.119826
H	-0.884930	2.836016	0.115887
H	0.338913	2.739524	1.388197
C	2.293787	1.493522	-0.074333
H	2.834313	0.715715	-0.601869
H	2.657026	2.449348	-0.450131
H	2.538000	1.441748	0.987044
H	0.163318	0.331855	1.288150

39

17H+ SCF Done: -527.767047655 A.U.

C	-0.871962	-1.254659	-0.012112
C	-2.208315	-0.610338	0.389119
C	-2.201675	0.768174	-0.229480
C	-0.785345	1.308530	-0.003882
H	-2.283944	-0.543871	1.474303
H	-3.037240	-1.225966	0.044056
H	-2.409449	0.716996	-1.300510
H	-2.935768	1.440268	0.210779
N	0.068230	0.015283	-0.322326
C	-1.018522	-2.019339	-1.329858
H	-0.071313	-2.413382	-1.690437
H	-1.689257	-2.862650	-1.170224
H	-1.456240	-1.393677	-2.110186
C	-0.398773	-2.173483	1.103637
H	-1.196525	-2.902583	1.254413
H	0.503827	-2.728744	0.865512
H	-0.265403	-1.643429	2.043221
C	-0.522794	2.437683	-1.002032

H	-1.380924	3.109410	-0.958445
H	0.361612	3.024414	-0.779254
H	-0.454732	2.066798	-2.026178
C	-0.594914	1.786748	1.430112
H	0.394540	2.204667	1.595881
H	-1.317775	2.582255	1.611634
H	-0.772761	1.001749	2.162514
C	1.619239	-0.047626	-0.030160
C	2.189755	-1.199297	-0.866596
H	3.268549	-1.219101	-0.716254
H	1.805938	-2.171480	-0.574411
H	2.010135	-1.052460	-1.932926
C	2.259235	1.249532	-0.537105
H	2.024334	2.103382	0.091465
H	3.339585	1.114522	-0.507323
H	1.986465	1.471993	-1.568080
C	1.982851	-0.240250	1.445068
H	3.043861	-0.010029	1.542004
H	1.446781	0.426843	2.110427
H	1.846878	-1.259752	1.785433
H	0.068542	0.022795	-1.341400

39

18H+ SCF Done: -370.458599103 A.U.

C	2.629053	-0.000501	0.185998
C	1.944740	-1.211570	-0.400676
C	0.454740	-1.369710	-0.043652
C	0.455245	1.369581	-0.043594
C	1.945217	1.210911	-0.400516
H	2.046135	-1.177340	-1.489267
H	2.424051	-2.136138	-0.077287
H	2.594612	-0.000570	1.276777
H	3.684076	-0.000686	-0.091526
H	2.046687	1.176802	-1.489102
H	2.424850	2.135256	-0.076961
N	-0.346084	0.000067	-0.308151
C	-1.784169	0.000303	0.310558
H	-1.610531	0.000353	1.382186
C	-2.616073	-1.227895	-0.064883
H	-2.269742	-2.150249	0.382243
H	-3.622821	-1.059238	0.312764
H	-2.688468	-1.353229	-1.145480
C	-2.615667	1.228700	-0.065135
H	-3.622599	1.060300	0.312128
H	-2.269251	2.150950	0.382139
H	-2.687634	1.354073	-1.145759
C	0.317075	1.791533	1.417575
H	-0.707829	1.989241	1.716001
H	0.739961	1.061264	2.102993
H	0.872214	2.720612	1.544551
C	-0.058997	2.455033	-1.007805
H	0.714680	3.218730	-1.078497
H	-0.206701	2.059700	-2.014259

H	-0.965198	2.951978	-0.691641
C	0.316570	-1.791607	1.417539
H	-0.708388	-1.988708	1.716168
H	0.871188	-2.721014	1.544402
H	0.740017	-1.061598	2.102886
C	-0.060012	-2.454951	-1.007832
H	-0.207605	-2.059552	-2.014276
H	0.713333	-3.218979	-1.078569
H	-0.966412	-2.951506	-0.691620
H	-0.482368	0.000124	-1.319399

27

19H+ SCF Done: -370.458599103 A.U.

C	2.665505	0.016230	0.164671
C	1.905920	-1.248692	-0.222105
C	0.480717	-1.237688	0.308488
C	0.462779	1.246447	0.233329
C	1.886031	1.240748	-0.303812
H	-0.061995	-2.112295	-0.031789
H	1.887161	-1.356420	-1.310447
H	2.392440	-2.141810	0.172682
H	2.791714	0.053511	1.250853
H	3.662749	0.009502	-0.275833
H	-0.091664	2.089930	-0.162194
H	0.441807	1.288966	1.322358
H	1.859672	1.273332	-1.397721
H	2.361804	2.165452	0.025896
H	0.458844	-1.211100	1.398048
N	-0.285934	-0.013008	-0.152370
C	-1.745492	-0.022017	0.331934
H	-1.669423	-0.079640	1.418026
C	-2.474245	-1.247388	-0.210615
H	-2.091274	-2.184822	0.184842
H	-3.521520	-1.178933	0.079361
H	-2.434864	-1.279211	-1.301728
C	-2.460165	1.262677	-0.071468
H	-3.511373	1.164711	0.195411
H	-2.077673	2.141768	0.441541
H	-2.403677	1.424837	-1.150061
H	-0.317480	-0.038625	-1.175189

30

20H+ SCF Done: -409.790459108 A.U.

C	2.858936	-0.002244	0.193298
C	2.102172	-1.250355	-0.253495
C	0.658956	-1.232386	0.229338
C	0.659603	1.241106	0.222359
C	2.106783	1.249179	-0.251122
H	0.118553	-2.102348	-0.126113
H	2.120609	-1.336398	-1.344188
H	2.567189	-2.155652	0.139330
H	2.954103	-0.003944	1.283231
H	3.868780	-0.002987	-0.217725
H	0.129296	2.108610	-0.152624

H	0.598286	1.231332	1.308586
H	2.129862	1.336824	-1.341660
H	2.574140	2.152246	0.144102
H	0.611357	-1.207133	1.315678
N	-0.066400	0.003306	-0.265350
C	-1.611693	-0.001800	-0.009368
C	-2.199622	-1.200680	-0.759152
H	-1.906423	-2.154677	-0.326547
H	-3.285394	-1.141004	-0.701551
H	-1.925535	-1.189172	-1.816190
C	-2.192725	1.289748	-0.591267
H	-3.278412	1.207165	-0.580916
H	-1.931225	2.169244	-0.006603
H	-1.883196	1.443617	-1.626903
C	-1.891167	-0.096834	1.487031
H	-1.452581	0.731966	2.041214
H	-2.970287	-0.050396	1.630659
H	-1.545028	-1.036502	1.914623
H	0.023247	0.001652	-1.284679

32

21H+ SCF Done: -410.993317114 A.U.

N	-0.000885	-0.000652	-0.387304
C	1.346408	-0.675563	-0.056577
C	2.338958	-0.329659	-1.160876
H	2.563551	0.737460	-1.187197
H	3.272217	-0.859561	-0.974931
H	1.973109	-0.636789	-2.142407
C	1.890478	-0.343325	1.324078
H	1.201182	-0.594093	2.124990
H	2.792671	-0.937307	1.469925
H	2.173607	0.705068	1.411199
C	-1.258269	-0.827943	-0.048590
C	-1.463034	-1.862155	-1.149780
H	-0.657013	-2.596833	-1.173396
H	-2.393110	-2.397206	-0.962480
H	-1.542359	-1.393298	-2.132509
C	-1.234887	-1.461702	1.333473
H	-2.196671	-1.951955	1.483666
H	-0.463229	-2.225670	1.420735
H	-1.109859	-0.736374	2.132061
C	-0.088207	1.503146	-0.054758
C	-0.635788	1.806278	1.331180
H	-0.576631	2.884875	1.476031
H	-1.682904	1.522411	1.428466
H	-0.064507	1.337544	2.126850
C	-0.894577	2.191040	-1.150654
H	-0.888922	3.265178	-0.970004
H	-0.462574	2.019212	-2.138336
H	-1.934604	1.862081	-1.158772
H	0.941438	1.849875	-0.115729
H	-2.072539	-0.107834	-0.097360
H	1.130344	-1.740831	-0.107881

H -0.003498 -0.002092 -1.406775
26
22H+ SCF Done: -332.343185782 A.U.
N 0.202640 -0.607722 -0.318061
C -1.201458 -0.030658 0.008769
C -2.177733 -1.149570 -0.361720
H -2.023732 -2.036242 0.256754
H -3.196336 -0.801654 -0.197635
H -2.084203 -1.429863 -1.412777
C -1.278750 0.288331 1.496175
H -0.623663 1.111333 1.777707
H -2.300934 0.585881 1.727384
H -1.039030 -0.583097 2.106785
C 1.485182 -0.067151 0.310054
C 1.751244 1.375825 -0.083822
H 2.743427 1.649385 0.273038
H 1.738584 1.502950 -1.167880
H 1.041081 2.068328 0.360466
C 2.615117 -1.000546 -0.109994
H 3.534747 -0.686042 0.381482
H 2.419693 -2.034624 0.179341
H 2.779666 -0.960225 -1.188701
H 1.329589 -0.145191 1.384119
C -1.457350 1.197664 -0.860857
H -0.803873 2.029867 -0.616230
H -1.347803 0.963058 -1.921089
H -2.484452 1.522570 -0.698648
H 0.165754 -1.598797 -0.075154
H 0.315499 -0.586955 -1.334170
23
23H+ SCF Done: -293.013703224 A.U.
N 0.000030 -0.631862 -0.000219
C 1.290461 0.143034 -0.248923
C 2.402740 -0.868252 -0.488415
H 2.164364 -1.545405 -1.310162
H 3.315025 -0.334368 -0.750792
H 2.603466 -1.457533 0.409019
C -1.290449 0.142947 0.248853
C -2.402502 -0.868559 0.488454
H -3.315088 -0.334864 0.750148
H -2.602688 -1.458458 -0.408698
H -2.164164 -1.545150 1.310666
C -1.580248 1.080455 -0.910774
H -2.535073 1.571528 -0.727042
H -0.825007 1.857417 -1.017577
H -1.660839 0.532648 -1.852014
H -1.110893 0.710567 1.160096
C 1.579955 1.080283 0.910993
H 0.824873 1.857422 1.017608
H 1.660017 0.532214 1.852132
H 2.535014 1.571099 0.727808
H -0.152910 -1.251997 -0.799403

H	1.111020	0.710814	-1.160087
H	0.152933	-1.252351	0.798698

26

24H+ SCF Done: -791.909762736 A.U.

N	-0.118179	-0.099830	-0.412841
C	-1.311939	0.796626	0.019781
C	-2.334710	0.779123	-1.104312
H	-2.748118	-0.220670	-1.242687
H	-3.152619	1.447725	-0.838684
H	-1.911683	1.129323	-2.047296
C	-1.909921	0.408215	1.360633
H	-1.166070	0.276492	2.140526
H	-2.572040	1.219852	1.662313
H	-2.505304	-0.499446	1.282421
C	1.341032	0.319066	0.020360
C	1.574410	1.702898	-0.594595
H	0.976382	2.483086	-0.129553
H	2.620900	1.956320	-0.432158
H	1.399047	1.709155	-1.671735
C	1.450175	0.341016	1.535061
H	2.491876	0.533106	1.790100
H	0.850808	1.133581	1.978070
H	1.171247	-0.616756	1.973156
H	-0.856341	1.780410	0.090367
Cl	-0.473045	-1.807179	-0.104433
C	2.303908	-0.690512	-0.602252
H	2.229799	-1.670735	-0.137807
H	2.146190	-0.792249	-1.677422
H	3.317483	-0.323280	-0.447843
H	-0.100279	-0.063651	-1.434589

23

25H+ SCF Done: -752.578390376 A.U.

N	-0.008722	0.203054	-0.427222
C	1.316377	-0.609843	-0.388578
C	2.341351	0.143529	-1.218560
H	2.565765	1.113928	-0.774096
H	3.263523	-0.435834	-1.243048
H	2.008680	0.285266	-2.248241
C	1.804240	-0.907373	1.017395
H	1.034189	-1.324689	1.659864
H	2.604099	-1.642478	0.929299
H	2.215724	-0.017316	1.490418
C	-1.312331	-0.617455	-0.509411
C	-1.547908	-1.447519	0.735927
H	-2.503811	-1.956695	0.620457
H	-0.784804	-2.208420	0.880242
H	-1.608724	-0.819933	1.625012
H	1.028802	-1.534248	-0.889663
Cl	-0.067930	1.465057	0.810175
C	-2.470249	0.302217	-0.858035
H	-2.739126	0.945692	-0.021423
H	-2.252743	0.920531	-1.730751

H	-3.332162	-0.319037	-1.099044
H	-1.097038	-1.264742	-1.363162
H	0.024627	0.749299	-1.290705

17

26H+ SCF Done: -1133.48206186 A.U.

N	0.427251	-0.000071	-0.446988
C	-1.104148	0.000023	0.042370
C	-1.715406	-1.259547	-0.560969
H	-1.304709	-2.168409	-0.128301
H	-2.781335	-1.234637	-0.336460
H	-1.607604	-1.289643	-1.646046
C	-1.116082	0.000009	1.556911
H	-2.158670	-0.000165	1.873485
H	-0.640932	-0.889555	1.966828
H	-0.641205	0.889745	1.966742
Cl	1.332730	1.439941	0.004461
C	-1.715451	1.259645	-0.560930
H	-1.304835	2.168487	-0.128136
H	-1.607655	1.289873	-1.645989
H	-2.781367	1.234599	-0.336349
Cl	1.332593	-1.439978	0.004478
H	0.433589	0.000035	-1.473117

29

27H+ SCF Done: -831.231952600 A.U.

N	-0.010999	0.243208	-0.397557
C	1.418787	-0.385610	-0.006243
C	2.406102	0.235963	-0.996921
H	2.483652	1.314880	-0.887996
H	3.389591	-0.188401	-0.800189
H	2.144772	-0.005132	-2.029242
C	1.786912	-0.045432	1.432113
H	1.092925	-0.465759	2.153205
H	2.766012	-0.485351	1.620807
H	1.871755	1.025144	1.598406
C	-1.432224	-0.375330	0.010316
C	-1.483444	-0.610501	1.508664
H	-2.502992	-0.898529	1.764222
H	-0.825862	-1.415433	1.827176
H	-1.245918	0.296361	2.063667
Cl	0.032315	1.985761	-0.045241
C	-2.506745	0.627496	-0.424526
H	-2.541459	1.506109	0.212763
H	-2.378284	0.941078	-1.461130
H	-3.468695	0.123720	-0.346912
C	1.382708	-1.896560	-0.212990
H	0.773856	-2.407147	0.527225
H	1.060631	-2.173372	-1.214499
H	2.406003	-2.250517	-0.089999
C	-1.627589	-1.660640	-0.801918
H	-1.032192	-2.492935	-0.450723
H	-2.674087	-1.944817	-0.699962
H	-1.438779	-1.503600	-1.865276

H	-0.020327	0.226986	-1.420515

51

28H+ SCF Done: -485.014378873 A.U.

C	-2.041166	-1.253259	-0.014918
C	-3.451178	-0.630231	0.030901
C	-3.308987	0.770555	-0.525122
C	-1.968146	1.284570	0.005935
H	-3.816108	-0.601516	1.056577
H	-4.118233	1.434488	-0.226334
N	-1.074664	0.012165	-0.201708
C	-1.886700	-2.132202	-1.254819
H	-0.875406	-2.510028	-1.383497
H	-2.552638	-2.989001	-1.157264
H	-2.175306	-1.591410	-2.158504
C	-1.782799	-2.025788	1.274612
H	-2.591131	-2.751964	1.368071
H	-0.848529	-2.578767	1.285392
H	-1.824835	-1.383752	2.152733
C	-1.516338	2.469349	-0.844129
H	-2.329375	3.196183	-0.837571
H	-0.628645	2.969404	-0.472383
H	-1.352923	2.178004	-1.882323
C	-2.045961	1.646282	1.486867
H	-1.091472	1.982393	1.884163
H	-2.754159	2.467478	1.593335
H	-2.400121	0.822011	2.101730
C	0.277676	-0.077125	0.519408
H	0.013653	-0.232144	1.562165
C	1.151703	1.193923	0.419872
C	1.128626	-1.271221	0.018069
H	0.592061	2.064849	0.744735
C	1.701319	1.416674	-1.000098
C	2.335009	1.014041	1.393976
H	0.550130	-2.188411	0.031290
C	1.669175	-1.034359	-1.402791
C	2.312697	-1.443254	0.990159
H	2.295765	2.333362	-1.004577
H	0.898596	1.569928	-1.723755
C	2.557004	0.216634	-1.425623
H	1.964512	0.880236	2.414193
H	2.931664	1.929293	1.387375
C	3.191110	-0.186667	0.972797
H	0.858391	-0.930377	-2.131537
H	2.236390	-1.916138	-1.709790
H	1.944375	-1.638329	2.001269
H	2.891225	-2.318567	0.685514
H	2.937486	0.378344	-2.436507
C	3.730812	0.040596	-0.448643
H	4.022241	-0.308825	1.670903
H	4.348607	-0.808065	-0.755035
H	4.368468	0.928596	-0.469732
H	-4.149341	-1.238246	-0.541264

H	-3.283699	0.756155	-1.617302
H	-0.862136	0.018541	-1.198837

37

29H+ SCF Done: -526.535101849 A.U.

C	-0.868431	-1.277681	-0.015775
C	-2.233339	-0.656548	0.038555
C	-2.232563	0.659041	0.038131
C	-0.866824	1.278428	-0.015853
H	-3.104893	-1.292038	0.097323
H	-3.103323	1.295655	0.096565
N	0.023303	-0.000229	-0.324905
C	-0.794809	-2.272052	-1.179479
H	0.138092	-2.825562	-1.209068
H	-1.601981	-2.993364	-1.049999
H	-0.949898	-1.777253	-2.139867
C	-0.566623	-1.960251	1.315727
H	-1.354182	-2.696516	1.480790
H	0.380382	-2.494263	1.311760
H	-0.592278	-1.259380	2.147088
C	-0.791286	2.273123	-1.179188
H	-1.600035	2.993044	-1.051947
H	0.140697	2.828308	-1.205963
H	-0.942826	1.778424	-2.140201
C	-0.565453	1.960418	1.316179
H	0.385235	2.487786	1.316107
H	-1.348536	2.702406	1.476993
H	-0.600059	1.260631	2.148135
C	1.573551	-0.000941	-0.024632
C	2.171208	-1.235169	-0.707991
H	3.255629	-1.162561	-0.640134
H	1.879908	-2.164847	-0.228520
H	1.911004	-1.278810	-1.765852
C	2.170834	1.239959	-0.696303
H	1.881921	2.164944	-0.206438
H	3.255273	1.165246	-0.631638
H	1.908220	1.295301	-1.752805
C	1.943351	-0.007889	1.458758
H	3.032785	0.005285	1.505623
H	1.591050	0.865868	1.993519
H	1.613245	-0.898844	1.979186
H	0.023756	-0.000487	-1.345098

49

30H+ SCF Done: -758.907034700 A.U.

C	-2.039944	-1.266210	-0.021576
C	-3.393028	-0.658357	-0.260499
C	-3.392983	0.658476	-0.260185
C	-2.039776	1.266170	-0.021513
H	-4.256009	-1.297079	-0.380431
H	-4.255906	1.297311	-0.379908
N	-1.112921	0.000043	-0.201760
C	-1.732012	-2.316346	-1.089815
H	-0.782387	-2.819779	-0.935960

H	-2.516313	-3.072359	-1.051599
H	-1.748441	-1.879382	-2.089355
C	-1.978882	-1.844895	1.394001
H	-2.759530	-2.602182	1.469502
H	-1.030924	-2.326533	1.619391
H	-2.184252	-1.089432	2.148972
C	-1.732168	2.316316	-1.090038
H	-2.516562	3.072205	-1.051724
H	-0.782556	2.819854	-0.936437
H	-1.748755	1.879062	-2.089438
C	-1.978316	1.845030	1.393923
H	-1.030292	2.326751	1.618958
H	-2.758994	2.602258	1.469639
H	-2.183306	1.089655	2.149098
C	0.229057	0.000017	0.541284
H	-0.043710	0.000181	1.593930
C	1.088158	1.249048	0.235319
C	1.088184	-1.249033	0.235733
H	0.520323	2.155680	0.414364
C	1.614224	1.242372	-1.210436
C	2.281926	1.244917	1.210715
H	0.520460	-2.155671	0.415091
C	1.614288	-1.242881	-1.210026
C	2.281948	-1.244498	1.211149
H	2.192618	2.154213	-1.376031
H	0.796028	1.267341	-1.935715
C	2.482977	-0.000273	-1.446247
H	1.922515	1.274756	2.243097
H	2.867097	2.153141	1.048632
C	3.146859	0.000176	0.978981
H	0.796085	-1.268203	-1.935291
H	2.192747	-2.154753	-1.375225
H	1.922540	-1.273956	2.243542
H	2.867143	-2.152766	1.049405
H	2.851335	-0.000418	-2.474322
C	3.668555	-0.000077	-0.467377
H	3.986890	0.000295	1.677007
H	4.294212	-0.880127	-0.639728
H	4.294166	0.879945	-0.640047
H	-0.890178	-0.000025	-1.197459

55

31H+ SCF Done: -912.623433853 A.U.

C	1.228731	1.275691	0.129152
C	2.607649	0.689785	-0.045610
C	2.607599	-0.689758	-0.045516
C	1.228537	-1.275488	0.129105
N	0.321867	-0.000056	-0.103835
C	0.948381	2.325791	-0.946613
H	-0.017787	2.806697	-0.836813
H	1.708403	3.101802	-0.868544
H	1.025221	1.895345	-1.946054
C	1.081780	1.837954	1.545001

H	1.826612	2.624963	1.658825
H	0.106246	2.277448	1.733970
H	1.287621	1.085256	2.302649
C	0.948418	-2.325688	-0.946872
H	1.708534	-3.101570	-0.868901
H	-0.017708	-2.806721	-0.837106
H	1.025096	-1.895053	-1.946240
C	1.081498	-1.838030	1.544865
H	0.105809	-2.277181	1.733826
H	1.825996	-2.625380	1.658430
H	1.287671	-1.085583	2.302678
C	-1.064915	-0.000010	0.564383
H	-0.848853	-0.000089	1.630094
C	-1.909727	-1.248081	0.214027
C	-1.909716	1.248058	0.214236
H	-1.356827	-2.156323	0.426013
C	-2.358737	-1.242622	-1.257577
C	-3.154647	-1.242687	1.124292
H	-1.356853	2.156302	0.426403
C	-2.358776	1.242845	-1.257368
C	-3.154624	1.242451	1.124542
H	-2.928640	-2.154176	-1.451803
H	-1.504224	-1.269360	-1.939487
C	-3.212774	0.000132	-1.540425
H	-2.850952	-1.269959	2.174664
H	-3.728127	-2.152474	0.932095
C	-4.006903	-0.000085	0.844924
H	-1.504262	1.269691	-1.939249
H	-2.928706	2.154413	-1.451433
H	-2.850927	1.269510	2.174920
H	-3.728112	2.152276	0.932546
H	-3.524460	0.000206	-2.587059
C	-4.449778	0.000063	-0.627332
H	-4.883364	-0.000128	1.496605
H	-5.065175	0.880143	-0.833132
H	-5.065198	-0.879958	-0.833309
C	3.798073	-1.400215	-0.153012
C	3.798153	1.400145	-0.153361
C	4.991335	-0.697283	-0.264317
C	4.991367	0.697114	-0.264533
H	3.805264	-2.482243	-0.147803
H	5.925260	-1.236505	-0.351739
H	5.925299	1.236277	-0.352256
H	3.805409	2.482178	-0.148485
H	0.153115	0.000093	-1.109413

67

32H+ SCF Done: -1069.92041166 A.U.

C	1.100112	1.302265	0.042276
C	2.465246	0.679718	-0.145240
C	2.434377	-0.697296	-0.232250
C	1.060820	-1.287556	0.002591
N	0.157529	0.020764	-0.185614

C	0.768619	2.302879	-1.090911
H	-0.274835	2.586615	-1.023404
H	0.888743	1.779092	-2.043540
C	1.061648	1.890996	1.476342
H	2.011613	2.420503	1.551094
H	1.155495	1.069235	2.186257
C	0.778482	-2.356438	-1.098755
H	1.760455	-2.715255	-1.396477
H	0.408539	-1.843603	-1.989834
C	0.938441	-1.793625	1.457929
H	-0.041338	-2.238377	1.609853
H	0.997689	-0.938374	2.129337
C	-1.233867	0.054534	0.483238
H	-1.013661	0.126481	1.545291
C	-2.081379	-1.211311	0.231137
C	-2.100076	1.266007	0.056253
H	-1.517033	-2.096305	0.488888
C	-2.575379	-1.312691	-1.223218
C	-3.304534	-1.154085	1.169890
H	-1.555830	2.189478	0.186548
C	-2.586482	1.156637	-1.399237
C	-3.324482	1.321711	0.992761
H	-3.147553	-2.236875	-1.333101
H	-1.744229	-1.382874	-1.928549
C	-3.445732	-0.100865	-1.571630
H	-2.976485	-1.107131	2.212047
H	-3.872894	-2.080043	1.054577
C	-4.175844	0.059241	0.826939
H	-1.750744	1.134134	-2.105296
H	-3.160770	2.054287	-1.640238
H	-3.002938	1.425721	2.032671
H	-3.906119	2.213706	0.748343
H	-3.784171	-0.177039	-2.607165
C	-4.658086	-0.043934	-0.628760
H	-5.033876	0.100783	1.501475
H	-5.285168	0.816639	-0.877389
H	-5.272142	-0.939415	-0.758089
C	3.611160	-1.415993	-0.436890
C	3.683482	1.357263	-0.184829
C	4.820348	-0.741503	-0.518985
C	4.857506	0.643133	-0.377099
H	3.598820	-2.493867	-0.512771
H	5.735370	-1.296860	-0.676574
H	5.802922	1.167812	-0.416463
H	3.731574	2.427176	-0.055131
C	1.593771	3.591690	-1.144026
H	2.603505	3.421528	-1.508871
H	1.111913	4.282913	-1.835853
H	1.654790	4.088043	-0.175817
C	-0.025219	2.858286	1.947022
H	-0.239818	3.636763	1.215130
H	-0.956928	2.365108	2.209388

H	0.334100	3.356146	2.848678
C	2.005140	-2.804766	1.884327
H	2.986905	-2.343040	1.969861
H	1.738734	-3.207306	2.861778
H	2.086337	-3.642955	1.192491
C	-0.062650	-3.607367	-0.825855
H	0.179547	-4.067414	0.130579
H	-1.133711	-3.440081	-0.858879
H	0.166026	-4.340904	-1.600067
H	-0.018077	0.038635	-1.189606

41

33H+ SCF Done: -528.954524181 A.U.

N	-0.214404	-0.119568	-0.265809
C	-1.150028	1.200780	-0.044603
C	-2.620988	0.908506	-0.375686
H	-3.079265	0.187155	0.298050
H	-3.157490	1.848115	-0.248419
H	-2.772149	0.599216	-1.409306
C	-1.098163	1.730242	1.384415
H	-0.127822	2.126909	1.661705
H	-1.806663	2.555859	1.446098
H	-1.405154	0.981143	2.110681
C	1.168224	-0.237325	0.530301
C	2.055773	0.983300	0.204960
H	1.560326	1.898940	0.515789
H	2.913903	0.887637	0.873723
C	1.898312	-1.495196	0.044726
H	1.965730	-1.562238	-1.037519
H	2.916107	-1.441569	0.430371
H	1.454874	-2.406676	0.433328
C	1.001801	-0.337590	2.053836
H	0.159964	-0.946008	2.359662
H	1.901473	-0.812745	2.444085
H	0.921473	0.630691	2.534181
C	-0.668002	2.255271	-1.047383
H	0.334192	2.618719	-0.850528
H	-0.715667	1.879425	-2.071568
H	-1.339486	3.110982	-0.987231
C	-1.047245	-1.426832	-0.449178
C	-1.401922	-2.150474	0.837881
H	-2.156195	-2.898397	0.590322
H	-0.556819	-2.674133	1.278572
H	-1.835275	-1.484628	1.582872
C	-0.506585	-2.361283	-1.534870
H	-1.351256	-2.954955	-1.886640
H	-0.123471	-1.811119	-2.396063
H	0.255790	-3.051630	-1.190367
H	-1.966467	-1.050107	-0.865119
C	2.574425	1.111388	-1.228168
H	3.086354	2.066731	-1.346217
H	3.286531	0.324089	-1.471022
H	1.784866	1.084224	-1.982108

H	0.118816	0.046633	-1.214053

44

34H+ SCF Done: -568.281188048 A.U.

N	0.023451	0.166081	-0.118812
C	1.102748	-0.905143	0.463318
C	2.557471	-0.397402	0.289787
H	2.723200	0.499724	0.886981
H	3.161466	-1.162555	0.780171
C	0.872925	-1.168075	1.946835
H	-0.041430	-1.720228	2.135311
H	1.695829	-1.784429	2.306758
H	0.866429	-0.251881	2.533327
C	-1.529764	0.020636	0.267014
C	-2.058130	-1.376576	-0.117659
H	-1.546879	-2.138482	0.463558
H	-3.084865	-1.387363	0.255271
C	-2.313474	1.058080	-0.547711
H	-2.082875	1.030369	-1.609665
H	-3.370369	0.816166	-0.440848
H	-2.177835	2.069716	-0.179186
C	-1.821854	0.240736	1.758961
H	-1.235121	1.027402	2.213325
H	-2.869222	0.530364	1.841104
H	-1.695486	-0.663207	2.344773
C	0.985715	-2.204174	-0.343587
H	0.090325	-2.772598	-0.127812
H	1.041275	-2.031144	-1.417454
H	1.835457	-2.832359	-0.077847
C	0.582212	1.627922	-0.174624
C	0.328177	2.496455	1.045984
H	0.926143	3.399449	0.915774
H	-0.707370	2.806740	1.154795
H	0.660917	2.020822	1.967004
C	0.265880	2.338451	-1.493346
H	1.028662	3.105519	-1.631405
H	0.333342	1.665211	-2.350289
H	-0.700432	2.830729	-1.512180
H	1.644200	1.468231	-0.202754
C	-2.085323	-1.743159	-1.601071
H	-2.391465	-2.783540	-1.712968
H	-2.796357	-1.130497	-2.153768
H	-1.115472	-1.648324	-2.090736
C	3.094176	-0.232024	-1.135686
H	4.085681	0.219710	-1.099447
H	3.191552	-1.194085	-1.636700
H	2.473681	0.403973	-1.768559
H	0.008309	-0.090362	-1.104137

47

35H+ SCF Done: -607.619526563 A.U.

N	-0.777553	0.175820	0.042752
C	0.210562	-0.963559	0.572331
C	1.630852	-0.387971	0.858974

H	1.566404	0.334624	1.669478
H	2.148475	-1.235125	1.314061
C	-0.344437	-1.503759	1.889670
H	-1.354429	-1.889956	1.782072
H	0.287007	-2.328793	2.217569
H	-0.338586	-0.751157	2.676882
C	-2.293270	-0.158798	-0.400520
C	-2.534990	-1.619057	-0.790573
H	-2.404013	-2.314768	0.032378
H	-3.579819	-1.680415	-1.093812
H	-1.938679	-1.931952	-1.641548
C	-2.543449	0.678141	-1.661805
H	-1.872767	0.368888	-2.465891
H	-3.563207	0.494755	-1.998447
H	-2.437123	1.745636	-1.508941
C	-3.268113	0.191378	0.725379
H	-3.310031	1.251076	0.957699
H	-4.263950	-0.099769	0.393943
H	-3.053325	-0.363998	1.637022
C	0.349686	-2.085564	-0.463768
H	-0.446151	-2.815638	-0.408364
H	0.411097	-1.706524	-1.482925
H	1.276230	-2.614859	-0.256272
C	-0.715440	1.398034	0.954129
C	-1.126538	2.733497	0.353171
H	-0.807031	3.505626	1.054061
H	-0.626198	2.927184	-0.595541
H	-2.197431	2.845119	0.212825
H	0.315157	1.489523	1.258275
H	-1.291342	1.166809	1.842375
C	2.602086	0.182205	-0.224392
C	3.686625	0.941046	0.565274
H	4.178410	0.282014	1.284280
H	4.450124	1.331670	-0.109638
H	3.259504	1.782464	1.115469
C	1.954527	1.163897	-1.212041
H	1.235388	0.674694	-1.875655
H	1.473207	2.002521	-0.708644
H	2.720221	1.585976	-1.865043
C	3.302496	-0.927212	-1.030672
H	3.735057	-1.681842	-0.370582
H	2.634038	-1.428288	-1.728235
H	4.114750	-0.494580	-1.618181
H	-0.341709	0.464674	-0.830871

50

36H+ SCF Done: -646.938716823 A.U.

N	-0.759403	0.036349	-0.140278
C	0.271851	-0.945877	0.620904
C	1.640528	-0.246622	0.861784
H	1.478712	0.635700	1.476980
H	2.147367	-0.938379	1.538381
C	-0.269736	-1.359438	1.995309

H	-1.326269	-1.602161	1.983771
H	0.265912	-2.255251	2.308634
H	-0.099341	-0.602997	2.755861
C	-2.275666	-0.433234	-0.426676
C	-2.449447	-1.956512	-0.478444
H	-2.217770	-2.464140	0.451109
H	-3.505847	-2.131812	-0.678229
H	-1.893256	-2.407984	-1.295176
C	-2.642052	0.072610	-1.831966
H	-1.915942	-0.256548	-2.578001
H	-3.601214	-0.366396	-2.103877
H	-2.751168	1.146985	-1.897499
C	-3.229594	0.134238	0.620889
H	-3.332051	1.212197	0.551823
H	-4.214745	-0.292055	0.435429
H	-2.941114	-0.131465	1.635281
C	0.506176	-2.198653	-0.236211
H	-0.271765	-2.940961	-0.127545
H	0.631637	-1.974936	-1.294306
H	1.426900	-2.659946	0.111541
C	-0.568329	1.532053	0.274740
C	-1.109024	2.537666	-0.735883
H	-0.659530	3.499397	-0.485021
H	-0.822217	2.303075	-1.760587
H	-2.187255	2.663402	-0.683984
H	0.504692	1.625326	0.216342
C	2.686442	0.096416	-0.258117
C	3.580215	1.201485	0.336323
H	4.030100	0.875661	1.276803
H	4.387800	1.455046	-0.352702
H	3.009018	2.111974	0.533705
C	2.109113	0.611064	-1.588207
H	1.473254	-0.124856	-2.084473
H	1.556865	1.544486	-1.481272
H	2.928517	0.816840	-2.278995
C	3.587123	-1.114274	-0.572847
H	3.999293	-1.547049	0.340995
H	3.060462	-1.898406	-1.113979
H	4.424289	-0.797608	-1.198023
C	-0.965389	1.915704	1.698066
H	-0.397353	2.812572	1.948340
H	-2.017792	2.162139	1.794500
H	-0.720977	1.163365	2.437624
H	-0.366660	0.030585	-1.079491

47

37H+ SCF Done: -682.685366770 A.U.

N	1.311156	-0.050689	-0.207512
C	2.021751	-1.410295	-0.093883
C	3.071254	-1.557558	-1.188739
H	3.937655	-0.913648	-1.044003
H	3.424321	-2.588471	-1.177960
H	2.649545	-1.365455	-2.176885

H	1.244395	-2.137161	-0.302513
C	2.563511	-1.649875	1.305051
H	1.793894	-1.544784	2.068887
H	2.938825	-2.671644	1.353000
H	3.391998	-0.984137	1.545386
C	2.293081	1.133349	0.034943
C	-0.026173	0.039284	0.551095
H	0.247224	0.204299	1.588998
C	-0.874396	-1.245899	0.456657
C	-0.867546	1.225954	0.031550
H	-0.311724	-2.102738	0.824191
C	-1.354225	-1.490526	-0.984858
C	-2.096030	-1.067736	1.380187
H	-0.288388	2.145111	0.071571
C	-1.349456	0.974073	-1.408644
C	-2.089600	1.385372	0.955785
H	-1.939372	-2.412192	-1.012887
H	-0.511525	-1.644201	-1.667379
C	-2.204936	-0.300753	-1.458106
H	-1.766351	-0.914346	2.411537
H	-2.680036	-1.990940	1.364130
C	-2.949177	0.115888	0.908629
H	-0.512499	0.894023	-2.111499
H	-1.928833	1.838406	-1.740488
H	-1.762253	1.585930	1.979814
H	-2.667158	2.252630	0.627646
H	-2.539892	-0.476448	-2.482461
C	-3.422350	-0.135779	-0.532550
H	-3.812337	0.232121	1.567603
H	-4.040476	0.698316	-0.875545
H	-4.043413	-1.034813	-0.570581
H	3.271412	0.665614	0.000765
C	2.153908	1.788820	1.401322
H	1.236705	2.363901	1.509195
H	2.219384	1.069536	2.215475
H	2.989573	2.480422	1.510120
C	2.221605	2.120856	-1.123338
H	1.270710	2.648276	-1.168007
H	3.009146	2.862710	-0.992092
H	2.394997	1.627015	-2.081085
H	1.059060	0.016443	-1.192964

62

38H+ SCF Done: -954.355990502 A.U.

N	0.067898	0.921251	-0.145903
C	-0.144693	2.458966	-0.052944
C	-0.956772	3.012310	-1.213432
H	-2.012100	2.766389	-1.164721
H	-0.871250	4.099075	-1.185653
H	-0.559907	2.679404	-2.174378
H	0.868877	2.821516	-0.203098
C	-0.589328	2.955810	1.315035
H	-0.062459	2.458481	2.129045

H	-0.343509	4.016597	1.368633
H	-1.657863	2.859531	1.476513
C	1.508202	0.657180	0.423201
H	1.604940	1.401628	1.209654
C	2.541979	0.937529	-0.688461
C	1.811909	-0.713883	1.052267
H	2.316244	1.883166	-1.185914
C	2.559727	-0.198718	-1.727008
C	3.927341	1.050067	-0.025687
H	1.092827	-0.938897	1.841056
C	1.868616	-1.848136	0.013747
C	3.202310	-0.584089	1.718944
H	3.283355	0.049828	-2.506292
H	1.593536	-0.294722	-2.235578
C	2.929378	-1.526555	-1.048978
H	3.931241	1.870983	0.696353
H	4.669705	1.289358	-0.790484
C	4.275054	-0.275549	0.664684
H	0.902767	-2.005135	-0.467831
H	2.113144	-2.781738	0.525318
H	3.182371	0.197099	2.483725
H	3.428398	-1.523995	2.227332
H	2.949684	-2.323625	-1.795171
C	4.308494	-1.401347	-0.381709
H	5.249411	-0.191060	1.150838
H	4.578288	-2.348275	0.093779
H	5.071812	-1.188819	-1.135207
C	-1.008522	-0.141630	0.256598
H	-0.411624	-0.860162	0.794787
C	-1.564325	-0.861797	-0.988721
C	-2.169137	0.209800	1.205737
H	-0.754290	-1.042908	-1.702339
C	-2.696316	-0.077337	-1.668558
C	-2.114585	-2.218634	-0.502813
H	-1.800285	0.757071	2.070140
C	-3.329350	0.956808	0.528918
C	-2.711061	-1.159052	1.693077
H	-3.052207	-0.651063	-2.527557
H	-2.334346	0.871924	-2.063212
C	-3.846700	0.152051	-0.673627
H	-1.316989	-2.811484	-0.047069
H	-2.484555	-2.783522	-1.361396
C	-3.247648	-1.977994	0.507110
H	-3.040893	1.956785	0.216449
H	-4.129754	1.088187	1.261332
H	-1.926122	-1.713633	2.214356
H	-3.508832	-0.978504	2.416980
H	-4.646291	0.709783	-1.166081
C	-4.381353	-1.202679	-0.183152
H	-3.621038	-2.938250	0.869975
H	-5.209043	-1.046605	0.514059
H	-4.772057	-1.781117	-1.024929

H	0.164212	0.759236	-1.147457

69

39H+ SCF Done: -954.355990502 A.U.

N	0.094764	0.406811	-0.160108
C	1.554066	0.374541	0.420316
H	1.522777	1.117414	1.213558
C	2.535339	0.824360	-0.683643
C	2.070635	-0.936007	1.040488
H	2.164304	1.725817	-1.175580
C	2.734648	-0.287239	-1.729814
C	3.883668	1.149725	-0.014979
H	1.394859	-1.279625	1.824510
C	2.309821	-2.038441	-0.006638
C	3.420514	-0.591876	1.714057
H	3.407496	0.079014	-2.508005
H	1.795355	-0.536109	-2.236980
C	3.310388	-1.544220	-1.061046
H	3.756933	1.955958	0.712757
H	4.580702	1.508604	-0.775730
C	4.434690	-0.109275	0.667383
H	1.382168	-2.340378	-0.495044
H	2.695734	-2.926585	0.498716
H	3.273802	0.171832	2.482479
H	3.790878	-1.487102	2.218382
H	3.459321	-2.321505	-1.813533
C	4.649978	-1.207202	-0.386906
H	5.381579	0.124637	1.158855
H	5.064887	-2.103119	0.082897
H	5.372508	-0.870700	-1.135483
C	-0.802957	-0.806996	0.244431
H	-0.097666	-1.429364	0.770807
C	-1.257245	-1.604105	-0.995197
C	-1.989549	-0.638074	1.210326
H	-0.438919	-1.657431	-1.720350
C	-2.508089	-1.008385	-1.658015
C	-1.580514	-3.029300	-0.500258
H	-1.693845	-0.036815	2.067877
C	-3.260848	-0.081901	0.547786
C	-2.305170	-2.072734	1.706191
H	-2.781964	-1.631164	-2.513008
H	-2.306450	-0.013498	-2.054972
C	-3.664768	-0.961832	-0.645253
H	-0.692096	-3.487574	-0.057590
H	-1.870655	-3.648122	-1.352267
C	-2.721894	-2.967978	0.527209
H	-3.131653	0.947462	0.224707
H	-4.061980	-0.069460	1.291040
H	-1.436609	-2.496586	2.217907
H	-3.112622	-2.017503	2.439735
H	-4.549478	-0.537116	-1.124714
C	-3.973221	-2.382083	-0.146373
H	-2.934161	-3.974468	0.894760

H	-4.804581	-2.355323	0.563374
H	-4.280977	-3.017194	-0.981652
C	-0.352743	1.889559	-0.087531
C	-1.239363	2.319048	-1.252188
C	-0.879920	2.362089	1.263709
H	0.594077	2.404945	-0.241458
C	-1.348254	3.851345	-1.255970
H	-2.235851	1.891097	-1.172722
H	-0.810937	1.976443	-2.198044
C	-0.965036	3.895431	1.243623
H	-1.872775	1.967392	1.458153
H	-0.230685	2.029307	2.074961
C	-1.843992	4.385564	0.090119
H	-2.016852	4.159741	-2.061350
H	-0.365964	4.280855	-1.478855
H	-1.360144	4.242716	2.199723
H	0.040367	4.317295	1.144447
H	-1.865477	5.477043	0.072336
H	-2.874217	4.052746	0.255316
H	0.224341	0.254683	-1.160174

54

40H+ SCF Done: -799.465897242 A.U.

N	-0.521324	0.565377	-0.286365
C	-0.718931	2.095765	-0.308382
C	-1.788736	2.511634	-1.308379
H	-2.795145	2.264652	-0.978383
H	-1.733432	3.595165	-1.415522
H	-1.619450	2.074112	-2.293054
H	0.229363	2.440750	-0.708324
C	-0.911006	2.735296	1.063618
H	-0.295243	2.280769	1.836767
H	-0.610801	3.780204	0.980438
H	-1.945546	2.716228	1.392384
C	0.716710	0.099822	0.516270
H	0.361087	0.048709	1.545038
C	1.925984	1.055773	0.435709
C	1.175243	-1.303444	0.050317
H	1.645214	2.059750	0.745798
C	2.533381	1.088781	-0.979228
C	2.987357	0.540027	1.429768
H	0.343668	-2.006656	0.060541
C	1.779962	-1.254538	-1.364220
C	2.240069	-1.805660	1.043859
H	3.375991	1.783764	-0.982482
H	1.820193	1.470996	-1.716899
C	2.995564	-0.318141	-1.384012
H	2.578531	0.531990	2.444015
H	3.829753	1.235612	1.429577
C	3.451976	-0.865372	1.028007
H	1.043317	-0.929816	-2.108153
H	2.067715	-2.266986	-1.655829
H	1.815883	-1.865813	2.049909

H	2.537035	-2.817592	0.758770
H	3.419624	-0.288633	-2.389931
C	4.051885	-0.823661	-0.387163
H	4.201482	-1.221014	1.738393
H	4.392755	-1.820753	-0.678996
H	4.924915	-0.165628	-0.404712
H	-0.298986	0.346794	-1.258167
C	-1.739138	-0.337160	0.028779
C	-2.677834	0.175561	1.115048
C	-2.481882	-0.722714	-1.250954
H	-1.286784	-1.245004	0.417670
C	-3.720971	-0.908806	1.422253
H	-3.197993	1.074205	0.785435
H	-2.122595	0.422669	2.019858
C	-3.538337	-1.788480	-0.935217
H	-2.968932	0.147380	-1.690583
H	-1.775187	-1.110141	-1.990556
C	-4.490889	-1.318835	0.165967
H	-4.404207	-0.534967	2.186675
H	-3.220089	-1.784612	1.847756
H	-4.089499	-2.028163	-1.846069
H	-3.037945	-2.708731	-0.616514
H	-5.206493	-2.108192	0.404299
H	-5.071384	-0.463777	-0.196836

41

41H+ SCF Done: -1063.59826443 A.U.

N	1.300287	0.252407	-0.294959
C	2.594246	-0.613816	0.018162
C	3.728857	-0.066609	-0.846638
H	4.057389	0.918852	-0.526873
H	4.575080	-0.746327	-0.756663
H	3.449548	-0.024593	-1.900861
C	2.912929	-0.506247	1.502512
H	2.127141	-0.922224	2.131314
H	3.819501	-1.081597	1.686178
H	3.103439	0.521689	1.804466
C	-0.027377	-0.273283	0.333290
H	0.323172	-0.938182	1.115958
C	-0.817681	-1.066003	-0.727723
C	-0.946649	0.774797	0.983676
H	-0.167073	-1.769847	-1.244179
C	-1.485820	-0.127387	-1.747118
C	-1.899816	-1.858577	0.035187
H	-0.377200	1.366193	1.700489
C	-1.649182	1.682388	-0.042566
C	-2.024035	-0.033104	1.744523
H	-2.017010	-0.736816	-2.481280
H	-0.743112	0.446976	-2.312758
C	-2.452278	0.829178	-1.032803
H	-1.434468	-2.546593	0.746178
H	-2.456385	-2.466278	-0.681553
C	-2.840452	-0.885951	0.760055

H	-0.937421	2.306742	-0.581739
H	-2.311287	2.362450	0.498530
H	-1.559110	-0.666984	2.504376
H	-2.674903	0.669706	2.268786
H	-2.930382	1.478470	-1.769068
C	-3.520386	0.024359	-0.275473
H	-3.594870	-1.452122	1.310415
H	-4.214543	0.706675	0.222208
H	-4.105350	-0.578510	-0.975510
Cl	1.637664	1.970913	0.009955
C	2.292197	-2.057457	-0.388357
H	1.497485	-2.510892	0.198808
H	2.059461	-2.145432	-1.449532
H	3.198635	-2.632471	-0.202599
H	1.182659	0.235007	-1.309977

50

42H+ SCF Done: -722.000102280 A.U.

N	1.258252	0.152804	-0.252084
C	2.272808	-1.081586	-0.034466
C	3.721140	-0.658995	-0.317105
H	4.132249	0.003869	0.442067
H	4.322274	-1.567196	-0.303255
H	3.840044	-0.209070	-1.303157
C	2.195322	-1.602792	1.399373
H	1.233073	-2.031604	1.660641
H	2.939010	-2.392809	1.501282
H	2.442517	-0.829737	2.124472
C	-0.096507	0.032428	0.501053
H	0.192769	0.053728	1.548377
C	-0.877555	-1.272564	0.216712
C	-1.061076	1.212343	0.227927
H	-0.252712	-2.141130	0.384673
C	-1.444469	-1.302186	-1.213229
C	-2.041409	-1.357752	1.225830
H	-0.566147	2.159464	0.403709
C	-1.630415	1.174147	-1.200527
C	-2.222700	1.115622	1.238588
H	-1.956894	-2.254696	-1.367427
H	-0.647965	-1.260308	-1.961564
C	-2.411041	-0.128207	-1.417683
H	-1.652049	-1.361922	2.247878
H	-2.558122	-2.308707	1.075816
C	-3.004292	-0.183984	1.024533
H	-0.840445	1.260956	-1.950676
H	-2.280072	2.041235	-1.341750
H	-1.835331	1.164250	2.260259
H	-2.872596	1.982995	1.100607
H	-2.807203	-0.153055	-2.435132
C	-3.565267	-0.219543	-0.406362
H	-3.821588	-0.247691	1.746388
H	-4.259804	0.611401	-0.557751
H	-4.127790	-1.143769	-0.564758

C 1.924530 -2.154313 -1.074830
H 0.948336 -2.602770 -0.943755
H 1.988905 -1.749240 -2.086522
H 2.660655 -2.953315 -0.997462
C 1.946592 1.539499 -0.088024
H 2.973812 1.339564 -0.344750
C 1.941813 2.052480 1.344595
H 0.958863 2.351601 1.699911
H 2.355685 1.323774 2.039148
H 2.584384 2.932586 1.376867
C 1.490106 2.556972 -1.132232
H 0.510463 2.986628 -0.952386
H 2.214665 3.371954 -1.119992
H 1.508199 2.129102 -2.136345
H 1.039576 0.094870 -1.245752
5
NH3H+ SCF Done: -57.0257019791 A.U.
N 0.000000 0.000000 0.000130
H 0.000000 0.963051 0.339953
H 0.834026 -0.481525 0.339953
H -0.834026 -0.481525 0.339953
H 0.000000 0.000000 -1.020767
14
NMe3H+ SCF Done: -175.002430567 A.U.
N 0.000321 -0.000008 -0.339654
C -0.821962 1.172275 0.103634
H -0.372037 2.082213 -0.283258
H -0.834012 1.189831 1.190178
C 1.426621 0.125340 0.103650
H 1.989854 -0.717819 -0.286124
H 1.448591 0.123139 1.190193
C -0.604744 -1.297625 0.103652
H -0.619016 -1.314606 1.190223
H -1.616036 -1.363543 -0.287640
H 0.000287 -2.114774 -0.278601
H -1.830868 1.057755 -0.282575
H 1.831350 1.057703 -0.279530
H 0.000152 0.000214 -1.360903
23
NEt3H+ SCF Done: -293.001404450 A.U.
N 0.001849 0.000720 0.003384
C -0.461540 1.373785 0.447933
H 0.399714 2.033772 0.387635
H -0.740186 1.271571 1.495136
C -1.597828 1.907279 -0.402693
H -2.490590 1.285330 -0.350767
H -1.864393 2.899297 -0.039240
H -1.296462 2.004619 -1.446887
C 1.421471 -0.282857 0.452807
H 1.558757 -1.359529 0.406412
H 1.472558 0.021890 1.496717
C 2.453768 0.421611 -0.406012

H	2.351099	1.505830	-0.381030
H	3.445164	0.174178	-0.027341
H	2.398766	0.086607	-1.443100
C	-0.955387	-1.085810	0.452015
H	-0.719766	-1.280410	1.497384
H	-1.956627	-0.666523	0.401598
C	-0.861264	-2.334520	-0.403777
H	0.124281	-2.797271	-0.366555
H	-1.582333	-3.062010	-0.031976
H	-1.111143	-2.120275	-1.444228
H	0.002909	0.000961	-1.019087

12

PyH+ SCF Done: -248.827391169 A.U.

C	-0.663721	1.182330	-0.000199
C	0.713927	1.204900	-0.000129
C	1.409660	-0.000219	0.000061
C	0.713550	-1.205122	0.000202
C	-0.664099	-1.182128	0.000136
N	-1.303507	0.000204	-0.000065
H	2.490922	-0.000378	0.000130
H	-1.280072	2.067938	-0.000350
H	1.228413	2.153905	-0.000229
H	1.227741	-2.154287	0.000359
H	-1.280745	-2.067540	0.000227
H	-2.317615	0.000370	-0.000103

29

1H+ SCF Done: -371.659699457 A.U.

N	0.019883	0.199607	0.205971
C	0.006493	1.503081	-0.566761
C	1.217453	2.381280	-0.312685
H	-0.898553	2.028131	-0.273422
H	-0.078574	1.238619	-1.617711
H	1.064471	3.321795	-0.841778
H	1.333358	2.611705	0.747401
H	2.141351	1.941700	-0.683948
C	1.209634	-0.711176	-0.228577
C	0.844897	-2.171654	-0.452319
H	1.540092	-0.286619	-1.174431
H	1.743378	-2.668390	-0.819292
H	0.552922	-2.669607	0.472214
H	0.068751	-2.314236	-1.199805
C	-1.376397	-0.449213	0.265159
C	-2.217390	0.260983	1.318415
H	-1.190189	-1.460146	0.613441
H	-3.166816	-0.265699	1.411579
H	-1.733273	0.245330	2.296597
H	-2.439064	1.293792	1.049389
C	-2.054682	-0.492097	-1.096269
H	-2.917009	-1.153849	-1.021464
H	-2.416526	0.489342	-1.400508
H	-1.405544	-0.879645	-1.879461
C	2.311821	-0.587422	0.817863

H	2.610572	0.443685	0.995853
H	1.992941	-1.030485	1.764314
H	3.189863	-1.133094	0.474340
H	0.217699	0.447729	1.175933

18

2H+ SCF Done: -252.468169751 A.U.

C	-1.485419	0.080352	0.225356
C	-0.676656	1.297822	-0.228934
C	0.765435	1.220102	0.245093
C	0.628231	-1.294508	0.243419
C	-0.814834	-1.218786	-0.227930
H	1.377861	2.030645	-0.144076
H	-0.691484	1.372922	-1.320196
H	-1.108189	2.221808	0.157036
H	-1.570987	0.085107	1.316001
H	-2.497683	0.135998	-0.175399
H	1.151091	-2.165746	-0.144337
H	0.697268	-1.294008	1.330494
H	-0.844535	-1.298681	-1.318693
H	-1.341319	-2.088914	0.165531
H	0.836531	1.213280	1.332072
N	1.387603	-0.074401	-0.226123
H	1.421528	-0.073454	-1.248447
H	2.356158	-0.128051	0.090846

30

3H+ SCF Done: -409.800943853 A.U.

C	-0.000050	1.952786	0.074589
C	1.256021	1.250608	-0.438569
C	1.348223	-0.224413	-0.031686
C	-1.348256	-0.224409	-0.031680
C	-1.256047	1.250563	-0.438673
H	1.279340	1.318881	-1.530440
H	2.156851	1.746108	-0.074378
H	-0.000104	2.000894	1.164782
H	-0.000049	2.985623	-0.275190
H	-1.279244	1.318736	-1.530557
H	-2.156928	1.746074	-0.074616
N	0.000026	-0.887112	-0.397451
H	-0.000014	-0.987460	-1.414439
C	-1.641087	-0.413427	1.455498
H	-2.675682	-0.123392	1.634507
H	-1.534378	-1.457638	1.753568
H	-1.014989	0.197309	2.097747
C	-2.401483	-0.962268	-0.859643
H	-2.193908	-0.896667	-1.929225
H	-2.467441	-2.014552	-0.576671
H	-3.374392	-0.505362	-0.682668
C	2.401535	-0.962148	-0.859663
H	2.467582	-2.014430	-0.576703
H	2.193962	-0.896556	-1.929233
H	3.374405	-0.505153	-0.682658
C	1.641100	-0.413526	1.455472

H	1.015113	0.197277	2.097754
H	1.534204	-1.457723	1.753484
H	2.675745	-0.123671	1.634459
H	0.000012	-1.841115	-0.031240

21

4H+ SCF Done: -291.795089078 A.U.

C	-1.909491	0.000012	0.269147
C	-1.184917	1.255652	-0.216099
C	0.277891	1.249935	0.197697
C	0.277911	-1.249945	0.197652
C	-1.184931	-1.255655	-0.216057
H	0.823523	2.098393	-0.210614
H	-1.254766	1.336399	-1.304902
H	-1.641473	2.156860	0.195077
H	-1.946378	0.000042	1.362493
H	-2.939807	0.000003	-0.086928
H	0.823497	-2.098392	-0.210751
H	0.388818	-1.251388	1.281997
H	-1.254856	-1.336462	-1.304846
H	-1.641453	-2.156831	0.195227
H	0.388737	1.251282	1.282049
N	0.974834	-0.000004	-0.293513
C	2.427377	0.000011	0.058281
H	2.889725	-0.891027	-0.357411
H	2.889701	0.891054	-0.357424
H	2.520747	0.000021	1.141017
H	0.907112	0.000013	-1.314122

33

5H+ SCF Done: -449.11643974 A.U.

C	-0.000005	2.166809	0.048870
C	1.243150	1.451787	-0.458769
C	1.353189	-0.015758	-0.021080
C	-1.353189	-0.015765	-0.021080
C	-1.243155	1.451780	-0.458771
H	1.257299	1.496323	-1.552407
H	2.149411	1.953871	-0.118253
H	-0.000006	2.227879	1.138467
H	-0.000007	3.195008	-0.315106
H	-1.257300	1.496312	-1.552409
H	-2.149419	1.953861	-0.118260
N	0.000001	-0.727700	-0.392193
C	0.000010	-2.184251	-0.037571
H	-0.880638	-2.654202	-0.457712
H	0.880693	-2.654179	-0.457666
H	-0.000015	-2.298493	1.038698
H	0.000001	-0.693584	-1.413536
C	2.458706	-0.691213	-0.842164
H	2.188677	-0.781784	-1.896368
H	3.348044	-0.064380	-0.786602
H	2.730898	-1.672967	-0.459713
C	1.673501	-0.143546	1.466259
H	2.670510	0.269238	1.618681

H	0.991930	0.410068	2.103528
H	1.701592	-1.179814	1.797868
C	-2.458707	-0.691219	-0.842164
H	-3.348045	-0.064386	-0.786599
H	-2.188679	-0.781784	-1.896369
H	-2.730897	-1.672974	-0.459716
C	-1.673504	-0.143554	1.466259
H	-0.991907	0.410020	2.103532
H	-2.670494	0.269273	1.618687
H	-1.701642	-1.179824	1.797856

39

6H+ SCF Done: -602.790039580 A.U.

N	-0.544887	-0.005951	-0.372476
C	-1.204134	-1.419032	0.014719
C	-0.483325	-2.449315	-0.861224
H	-0.941652	-3.421453	-0.683637
H	-0.590522	-2.223438	-1.923533
H	0.574257	-2.529960	-0.621273
C	-1.236020	1.409114	-0.020189
C	-2.395582	1.626203	-0.998851
H	-2.715788	2.663122	-0.905863
H	-2.085709	1.478709	-2.035473
H	-3.257941	1.003091	-0.795405
C	-1.714617	1.465680	1.422678
H	-2.130429	2.459828	1.588179
H	-2.502173	0.747348	1.632322
H	-0.904722	1.332188	2.136295
C	0.919577	-0.006122	-0.157190
C	1.735796	-0.055034	-1.279377
C	1.469878	0.070086	1.115197
C	3.116701	-0.045674	-1.128953
H	1.304475	-0.098145	-2.272207
C	2.851793	0.080963	1.257670
H	0.846405	0.136785	1.991212
C	3.677422	0.019024	0.140769
H	3.748336	-0.086796	-2.005903
H	3.279665	0.142262	2.249146
H	4.752532	0.026716	0.259936
C	-1.012828	-1.773749	1.485653
H	-1.425079	-1.033871	2.164271
H	-1.547495	-2.707850	1.658030
H	0.029969	-1.950929	1.733959
C	-2.686130	-1.413328	-0.350132
H	-2.852125	-1.137980	-1.390808
H	-3.056356	-2.429571	-0.219851
H	-3.277991	-0.770289	0.294785
C	-0.193674	2.502829	-0.278803
H	0.634157	2.473632	0.423874
H	0.204624	2.455456	-1.292348
H	-0.695698	3.462600	-0.163038
H	-0.645680	-0.019668	-1.387139

40

7H+ SCF Done: -695.066860902 A.U.

N	-1.056909	0.044707	0.377432
C	-1.598924	1.372644	-0.359400
C	-1.195217	2.531297	0.558102
H	-1.602253	3.449001	0.135665
H	-1.607745	2.420718	1.562430
H	-0.116397	2.650488	0.628714
C	-1.574677	-1.445888	0.009231
C	-2.952427	-1.625720	0.653789
H	-3.229332	-2.674518	0.554181
H	-2.929049	-1.400401	1.722468
H	-3.733352	-1.040353	0.183974
C	-1.625555	-1.675385	-1.493056
H	-1.938271	-2.707644	-1.651160
H	-2.348696	-1.037809	-1.993578
H	-0.653581	-1.560366	-1.967623
C	0.399805	0.109241	0.605865
C	1.297390	-0.081113	-0.430986
C	0.851762	0.341269	1.898967
C	2.666240	-0.020342	-0.160454
H	0.967335	-0.289901	-1.434365
C	2.214545	0.401700	2.159699
H	0.148099	0.474905	2.710653
C	3.129871	0.225331	1.134796
H	4.191956	0.271066	1.328667
H	2.558824	0.585408	3.167571
C	-0.981000	1.569282	-1.739237
H	-1.155826	0.736620	-2.412925
H	-1.458004	2.446578	-2.176108
H	0.084032	1.780054	-1.690531
C	-3.120525	1.318352	-0.451567
H	-3.586705	1.161454	0.519907
H	-3.457035	2.286719	-0.820046
H	-3.474865	0.566892	-1.150865
C	-0.613904	-2.440239	0.669658
H	0.370851	-2.451061	0.210698
H	-0.503058	-2.254128	1.737905
H	-1.044661	-3.433327	0.549388
C	3.596844	-0.217560	-1.228171
N	4.345302	-0.375839	-2.089272
H	-1.466389	0.160319	1.304453

40

8H+ SCF Done: -695.065731934 A.U.

N	-0.620871	0.039148	0.145403
C	-1.313036	1.348088	-0.501719
C	-0.472289	2.529262	-0.007584
H	-0.939702	3.444343	-0.368639
H	-0.440944	2.582845	1.081253
H	0.544702	2.506216	-0.393686
C	-1.359805	-1.403159	0.240336
C	-2.383394	-1.319303	1.377423
H	-2.706864	-2.334281	1.604022

H	-1.948531	-0.908160	2.290658
H	-3.267615	-0.748938	1.120140
C	-2.019489	-1.792060	-1.072678
H	-2.458138	-2.780432	-0.931759
H	-2.824194	-1.118715	-1.354051
H	-1.309269	-1.870858	-1.892720
C	0.798637	-0.066236	-0.233781
C	1.782759	0.209078	0.729795
C	1.179883	-0.455892	-1.505712
C	3.136349	0.112928	0.395911
C	2.528888	-0.551893	-1.828346
H	0.441569	-0.701733	-2.249905
C	3.506368	-0.262800	-0.884819
H	3.882038	0.331166	1.146906
H	2.809906	-0.859245	-2.826145
H	4.553642	-0.336531	-1.142117
C	-1.317875	1.318093	-2.025400
H	-1.818586	0.447760	-2.437247
H	-1.871277	2.197659	-2.354394
H	-0.318919	1.396955	-2.445550
C	-2.732007	1.482869	0.040212
H	-2.760578	1.474204	1.128838
H	-3.112066	2.449763	-0.287687
H	-3.404097	0.723938	-0.349590
C	-0.293960	-2.432321	0.630609
H	0.435067	-2.609112	-0.155443
H	0.230422	-2.157532	1.545762
H	-0.809402	-3.372785	0.819928
C	1.407411	0.570904	2.057641
N	1.052108	0.849151	3.118448
H	-0.616462	0.300039	1.133153

40

9H+ SCF Done: -695.067218852 A.U.

N	-1.104403	-0.006007	-0.374394
C	-1.749962	-1.423990	0.042340
C	-1.065530	-2.453459	-0.861901
H	-1.512814	-3.426049	-0.660564
H	-1.223182	-2.231688	-1.918491
H	0.001934	-2.534044	-0.669572
C	-1.780003	1.416609	0.004310
C	-2.968404	1.633368	-0.937548
H	-3.289935	2.668094	-0.826704
H	-2.688825	1.495321	-1.983800
H	-3.820971	1.004666	-0.711882
C	-2.211897	1.469105	1.461304
H	-2.624497	2.462225	1.640213
H	-2.990691	0.749089	1.695392
H	-1.380128	1.338753	2.149699
C	0.362555	-0.006899	-0.211830
C	1.143051	-0.061281	-1.359661
C	0.953423	0.070845	1.043088
C	2.524249	-0.057271	-1.259546

H	0.685246	-0.104774	-2.339629
C	2.334053	0.076631	1.149743
H	0.360639	0.142567	1.939184
C	3.124936	0.007637	-0.000427
H	3.131732	-0.101898	-2.151941
H	2.796794	0.138621	2.124255
C	-1.500133	-1.774397	1.504727
H	-1.876575	-1.029008	2.197929
H	-2.036785	-2.702227	1.702384
H	-0.450170	-1.964567	1.710598
C	-3.243809	-1.411602	-0.266888
H	-3.446104	-1.136931	-1.301253
H	-3.611909	-2.426702	-0.122520
H	-3.809349	-0.766475	0.399037
C	-0.742636	2.506516	-0.286355
H	0.105382	2.479331	0.392260
H	-0.375723	2.461146	-1.311901
H	-1.239455	3.466963	-0.155400
C	4.550522	0.009603	0.111295
N	5.698858	0.010495	0.201648
H	-1.248289	-0.022318	-1.383965

55

10H+ SCF Done: -1061.16454338 A.U.

N	-0.222593	0.023084	-0.171138
C	-1.726545	0.207970	0.171976
C	-2.015145	1.212592	1.286664
C	-2.481059	0.555263	-1.111179
H	-2.056889	-0.775533	0.502844
C	-3.526038	1.235104	1.560669
H	-1.703408	2.215283	0.989733
H	-1.477778	0.963282	2.198564
C	-3.988933	0.614277	-0.839104
H	-2.141766	1.522897	-1.491877
H	-2.270243	-0.192819	-1.878603
C	-4.318666	1.580388	0.299513
H	-3.728073	1.958464	2.352074
H	-3.840707	0.257296	1.939361
H	-4.505035	0.905553	-1.755265
H	-4.344459	-0.388649	-0.581896
H	-5.389505	1.558433	0.510413
H	-4.077281	2.603022	-0.009607
C	0.526204	1.384175	-0.294293
C	1.309495	1.820382	0.937638
C	1.370395	1.445732	-1.568534
H	-0.293675	2.084003	-0.432844
C	1.833752	3.248070	0.718882
H	2.153138	1.150423	1.111939
H	0.680851	1.802439	1.825454
C	1.867644	2.881484	-1.774871
H	2.226367	0.778029	-1.502562
H	0.776073	1.126463	-2.428444
C	2.661869	3.365755	-0.560747

H	2.423145	3.542352	1.588677
H	0.982622	3.935160	0.667777
H	2.480382	2.919047	-2.676997
H	1.012958	3.544907	-1.944693
H	2.980599	4.399726	-0.706078
H	3.571901	2.764433	-0.461201
C	0.404336	-1.156434	0.590625
C	0.293835	-1.072482	2.105407
H	-0.745781	-1.051214	2.420668
H	0.743091	-1.969787	2.519955
H	0.818515	-0.214026	2.505188
C	1.856150	-1.349500	0.070374
C	-0.290554	-2.469149	0.108878
O	2.748761	-1.334909	1.038895
O	2.083972	-1.527507	-1.099695
O	-0.271321	-3.473160	0.765415
O	-0.825549	-2.341057	-1.097566
C	4.128311	-1.572069	0.651585
H	4.694696	-1.519107	1.574742
H	4.214663	-2.555700	0.194800
H	4.450129	-0.805282	-0.050163
C	-1.415116	-3.529343	-1.687330
H	-1.803650	-3.207337	-2.647251
H	-0.647596	-4.290150	-1.810336
H	-2.212106	-3.899258	-1.046131
H	-0.239546	-0.324162	-1.133579

52

11H+ SCF Done: -910.412126116 A.U.

N	0.113821	0.976338	-0.288060
C	0.164799	2.525374	-0.223672
C	-0.858858	3.103290	-1.187948
H	-1.878326	2.929333	-0.846143
H	-0.697665	4.179216	-1.249830
H	-0.750355	2.691806	-2.192551
H	1.160354	2.758580	-0.592738
C	0.005857	3.057978	1.188261
H	0.713966	2.619033	1.889375
H	0.195363	4.130865	1.159882
H	-1.006239	2.911433	1.561779
C	-1.233446	0.414548	-0.057929
C	-1.967220	-0.019288	-1.148108
C	-1.758567	0.315808	1.227143
C	-3.240879	-0.547611	-0.974030
H	-1.562531	0.047861	-2.150308
C	-3.023666	-0.207098	1.407364
H	-1.193368	0.631094	2.090968
C	-3.779323	-0.640824	0.310927
H	-3.793062	-0.879965	-1.839452
H	-3.447546	-0.291675	2.398430
O	-5.002337	-1.133191	0.597051
C	-5.830686	-1.593584	-0.472897
H	-6.750941	-1.935079	-0.007523

H	-6.053030	-0.784564	-1.172635
H	-5.359888	-2.423857	-1.004408
C	1.203639	0.245839	0.523802
H	0.920038	0.386961	1.563536
C	2.610506	0.820767	0.288733
C	1.193155	-1.255809	0.179900
H	2.633808	1.884440	0.523166
C	3.072164	0.578884	-1.159415
C	3.567184	0.096651	1.258062
H	0.190008	-1.657856	0.321096
C	1.659383	-1.487894	-1.268560
C	2.161444	-1.962813	1.146284
H	4.073610	0.995929	-1.284897
H	2.436611	1.106030	-1.879794
C	3.078599	-0.929630	-1.458324
H	3.260594	0.278322	2.291968
H	4.567698	0.519042	1.140896
C	3.580999	-1.408727	0.960143
H	0.972430	-1.034521	-1.992112
H	1.639039	-2.559630	-1.477188
H	1.830008	-1.822766	2.178969
H	2.139954	-3.036107	0.944288
H	3.402116	-1.095347	-2.488023
C	4.039418	-1.640179	-0.489427
H	4.263548	-1.910986	1.649131
H	4.063405	-2.711227	-0.707891
H	5.055291	-1.259509	-0.626095
H	0.330040	0.770207	-1.263735

36

12H+ SCF Done: -488.390275302 A.U.

N	0.003217	-0.131738	-0.262099
C	-1.495921	0.382975	-0.009465
C	-2.453836	-0.814570	-0.076067
H	-2.300255	-1.529360	0.726291
H	-3.462137	-0.416535	0.029918
H	-2.405708	-1.330041	-1.033235
C	-1.624489	1.029038	1.365568
H	-1.050816	1.944286	1.468050
H	-2.673811	1.286738	1.509152
H	-1.345720	0.342697	2.164392
C	1.247294	0.855172	-0.016859
C	0.934497	2.285324	-0.456563
H	0.163810	2.764836	0.136651
H	1.846987	2.864057	-0.318837
H	0.671639	2.341676	-1.511294
C	2.403187	0.352664	-0.895347
H	2.087565	0.162672	-1.923039
H	3.158853	1.136033	-0.930689
H	2.878292	-0.535984	-0.495502
C	1.669143	0.825742	1.450787
H	2.521376	1.497170	1.555546
H	0.892792	1.174542	2.125202

H	1.997750	-0.162627	1.763978
C	-1.893120	1.337585	-1.142948
H	-1.461169	2.326205	-1.060117
H	-1.644533	0.924266	-2.122846
H	-2.975212	1.455220	-1.111329
C	0.235031	-1.452438	0.393892
C	-0.192935	-2.744492	-0.232079
C	1.264441	-2.439110	-0.079886
H	0.099460	-1.367337	1.460177
H	-0.582999	-2.725790	-1.240320
H	-0.671740	-3.464472	0.416638
H	1.847791	-2.933977	0.684164
H	1.800248	-2.243880	-0.995403
H	0.025269	-0.295565	-1.269051

35

13H+ SCF Done: -450.308474701 A.U.

N	0.133689	-0.007958	-0.431037
C	-1.285893	-0.607789	-0.025287
C	-1.268984	-2.131392	-0.196469
H	-0.627680	-2.636508	0.522333
H	-2.285512	-2.483494	-0.025195
H	-0.990588	-2.433309	-1.206217
C	-1.679190	-0.256773	1.405601
H	-1.918743	0.796228	1.519111
H	-2.580314	-0.820666	1.644646
H	-0.918893	-0.529828	2.132416
C	0.512326	1.453495	-0.052203
C	-0.313704	2.480664	-0.817353
H	-1.306196	2.615656	-0.393168
H	0.204556	3.437166	-0.742688
H	-0.406234	2.238999	-1.876818
C	0.575779	1.797597	1.434437
H	1.256967	2.643578	1.532346
H	-0.387532	2.110689	1.826848
H	0.958271	0.997578	2.056973
C	-2.311035	-0.038949	-1.013962
H	-2.448713	1.032030	-0.913479
H	-2.041813	-0.266295	-2.047634
H	-3.272447	-0.511665	-0.816292
C	1.338994	-0.979185	-0.304127
C	1.783334	-1.269351	1.120223
H	2.455935	-2.126850	1.083466
H	2.338507	-0.440163	1.553068
H	0.959708	-1.529660	1.780444
C	2.502420	-0.519033	-1.179521
H	3.178323	-1.365773	-1.300093
H	2.177466	-0.215792	-2.176727
H	3.075382	0.291539	-0.730913
H	0.962331	-1.894510	-0.743942
H	1.522209	1.514210	-0.444652
H	0.034898	0.056853	-1.444605

38

14H+ SCF Done: -489.625290012 A.U.

N	0.011241	-0.085242	-0.402916
C	-1.576403	0.023263	-0.110200
C	-2.269997	-1.301058	-0.473682
H	-2.000684	-2.132801	0.170638
H	-3.339077	-1.137362	-0.345935
H	-2.111586	-1.579881	-1.515945
C	-1.885254	0.352367	1.349184
H	-1.456867	1.289052	1.685545
H	-2.968065	0.445339	1.435352
H	-1.573754	-0.434063	2.030560
C	0.985569	1.080290	0.101592
C	0.315893	2.458929	0.022253
H	-0.536790	2.582786	0.678208
H	1.063964	3.184474	0.338131
H	0.032487	2.713157	-0.996975
C	2.179249	1.157992	-0.867077
H	1.855805	1.179533	-1.909354
H	2.698955	2.095174	-0.673671
H	2.900317	0.362579	-0.736426
C	1.473228	0.812482	1.522316
H	2.166772	-0.022089	1.562783
H	2.014503	1.695711	1.860298
H	0.662215	0.632534	2.222687
C	-2.164367	1.081014	-1.060133
H	-1.967854	2.101521	-0.760887
H	-1.815853	0.939010	-2.085430
H	-3.245733	0.953741	-1.070785
C	0.524390	-1.558584	-0.275132
C	0.507995	-2.153764	1.128375
H	0.549481	-3.237444	1.010631
H	1.368273	-1.858915	1.720777
H	-0.391232	-1.926712	1.689790
C	1.853266	-1.844289	-0.967833
H	1.900137	-2.925660	-1.103577
H	1.924738	-1.390072	-1.954928
H	2.716061	-1.557790	-0.372061
H	0.028257	0.056999	-1.412351
H	-0.214582	-2.083987	-0.864645

41

15H+ SCF Done: -528.935847633 A.U.

N	-0.000729	-0.000557	-0.335384
C	1.080843	-1.172973	-0.017170
C	2.535765	-0.720043	-0.228197
H	2.867067	0.066893	0.436100
H	3.151115	-1.590571	-0.006187
H	2.736029	-0.442679	-1.260025
C	0.949224	-1.681534	1.418006
H	-0.012716	-2.127182	1.638980
H	1.699632	-2.460068	1.555825
H	1.145779	-0.904175	2.150943
C	-1.557705	-0.348783	-0.018286

C	-1.893925	-1.833524	-0.236747
H	-1.375535	-2.518494	0.420730
H	-2.954769	-1.932508	-0.011804
H	-1.757193	-2.137651	-1.271408
C	-2.440417	0.387022	-1.050918
H	-2.001910	0.360747	-2.049884
H	-3.389399	-0.142266	-1.111022
H	-2.668775	1.411532	-0.796446
C	-1.934924	0.014261	1.417163
H	-1.834141	1.068470	1.643704
H	-2.987049	-0.239213	1.547509
H	-1.368214	-0.553060	2.150183
C	0.891707	-2.302703	-1.053176
H	0.106222	-3.004595	-0.815508
H	0.720772	-1.905997	-2.055404
H	1.819330	-2.870175	-1.095390
C	0.476313	1.521295	-0.018399
C	0.970733	1.664779	1.420588
H	1.270629	2.703820	1.557366
H	0.193376	1.450303	2.148237
H	1.834849	1.053871	1.650376
C	-0.639795	2.555866	-0.240708
H	-0.193471	3.523589	-0.016962
H	-0.971768	2.589573	-1.275336
H	-1.492298	2.451360	0.417442
H	-0.003621	-0.002035	-1.353558
C	1.562297	1.916647	-1.044813
H	2.564608	1.623250	-0.768840
H	1.343355	1.527498	-2.040758
H	1.562493	3.001801	-1.125232

36

16H+ SCF Done: -488.446618442 A.U.

C	-1.440984	0.031609	-0.024783
C	-1.616810	-1.497098	-0.144128
C	-0.374053	-2.112071	0.468842
C	0.779068	-1.195782	0.049547
H	-1.710724	-1.784767	-1.190272
H	-2.529355	-1.811637	0.358402
H	-0.443464	-2.135774	1.559010
H	-0.192543	-3.131519	0.133493
N	0.118170	0.204274	0.276580
C	-2.205429	0.562054	1.187348
H	-2.028654	1.618693	1.371066
H	-3.271787	0.427569	1.008738
H	-1.949667	0.004218	2.090562
C	-1.872917	0.713463	-1.317662
H	-2.905403	0.414826	-1.501529
H	-1.852421	1.798593	-1.272356
H	-1.284266	0.383822	-2.172432
C	1.976990	-1.419445	0.971119
H	2.160173	-2.493550	1.013524
H	2.889081	-0.945240	0.625543

H	1.769241	-1.084893	1.989207
C	1.144758	-1.387887	-1.419636
H	1.922812	-0.703652	-1.747698
H	1.526839	-2.401376	-1.540261
H	0.289266	-1.275513	-2.081878
C	0.785499	1.457096	-0.306900
H	0.586036	1.420615	-1.374349
C	0.175146	2.716921	0.308541
H	0.680672	3.581174	-0.119826
H	-0.884930	2.836016	0.115887
H	0.338913	2.739524	1.388197
C	2.293787	1.493522	-0.074333
H	2.834313	0.715715	-0.601869
H	2.657026	2.449348	-0.450131
H	2.538000	1.441748	0.987044
H	0.163318	0.331855	1.288150

39

17H+ SCF Done: -527.767047655 A.U.

C	-0.871962	-1.254659	-0.012112
C	-2.208315	-0.610338	0.389119
C	-2.201675	0.768174	-0.229480
C	-0.785345	1.308530	-0.003882
H	-2.283944	-0.543871	1.474303
H	-3.037240	-1.225966	0.044056
H	-2.409449	0.716996	-1.300510
H	-2.935768	1.440268	0.210779
N	0.068230	0.015283	-0.322326
C	-1.018522	-2.019339	-1.329858
H	-0.071313	-2.413382	-1.690437
H	-1.689257	-2.862650	-1.170224
H	-1.456240	-1.393677	-2.110186
C	-0.398773	-2.173483	1.103637
H	-1.196525	-2.902583	1.254413
H	0.503827	-2.728744	0.865512
H	-0.265403	-1.643429	2.043221
C	-0.522794	2.437683	-1.002032
H	-1.380924	3.109410	-0.958445
H	0.361612	3.024414	-0.779254
H	-0.454732	2.066798	-2.026178
C	-0.594914	1.786748	1.430112
H	0.394540	2.204667	1.595881
H	-1.317775	2.582255	1.611634
H	-0.772761	1.001749	2.162514
C	1.619239	-0.047626	-0.030160
C	2.189755	-1.199297	-0.866596
H	3.268549	-1.219101	-0.716254
H	1.805938	-2.171480	-0.574411
H	2.010135	-1.052460	-1.932926
C	2.259235	1.249532	-0.537105
H	2.024334	2.103382	0.091465
H	3.339585	1.114522	-0.507323
H	1.986465	1.471993	-1.568080

C	1.982851	-0.240250	1.445068
H	3.043861	-0.010029	1.542004
H	1.446781	0.426843	2.110427
H	1.846878	-1.259752	1.785433
H	0.068542	0.022795	-1.341400

39

18H+ SCF Done: -370.458599103 A.U.

C	2.629053	-0.000501	0.185998
C	1.944740	-1.211570	-0.400676
C	0.454740	-1.369710	-0.043652
C	0.455245	1.369581	-0.043594
C	1.945217	1.210911	-0.400516
H	2.046135	-1.177340	-1.489267
H	2.424051	-2.136138	-0.077287
H	2.594612	-0.000570	1.276777
H	3.684076	-0.000686	-0.091526
H	2.046687	1.176802	-1.489102
H	2.424850	2.135256	-0.076961
N	-0.346084	0.000067	-0.308151
C	-1.784169	0.000303	0.310558
H	-1.610531	0.000353	1.382186
C	-2.616073	-1.227895	-0.064883
H	-2.269742	-2.150249	0.382243
H	-3.622821	-1.059238	0.312764
H	-2.688468	-1.353229	-1.145480
C	-2.615667	1.228700	-0.065135
H	-3.622599	1.060300	0.312128
H	-2.269251	2.150950	0.382139
H	-2.687634	1.354073	-1.145759
C	0.317075	1.791533	1.417575
H	-0.707829	1.989241	1.716001
H	0.739961	1.061264	2.102993
H	0.872214	2.720612	1.544551
C	-0.058997	2.455033	-1.007805
H	0.714680	3.218730	-1.078497
H	-0.206701	2.059700	-2.014259
H	-0.965198	2.951978	-0.691641
C	0.316570	-1.791607	1.417539
H	-0.708388	-1.988708	1.716168
H	0.871188	-2.721014	1.544402
H	0.740017	-1.061598	2.102886
C	-0.060012	-2.454951	-1.007832
H	-0.207605	-2.059552	-2.014276
H	0.713333	-3.218979	-1.078569
H	-0.966412	-2.951506	-0.691620
H	-0.482368	0.000124	-1.319399

27

19H+ SCF Done: -370.458599103 A.U.

C	2.665505	0.016230	0.164671
C	1.905920	-1.248692	-0.222105
C	0.480717	-1.237688	0.308488
C	0.462779	1.246447	0.233329

C	1.886031	1.240748	-0.303812
H	-0.061995	-2.112295	-0.031789
H	1.887161	-1.356420	-1.310447
H	2.392440	-2.141810	0.172682
H	2.791714	0.053511	1.250853
H	3.662749	0.009502	-0.275833
H	-0.091664	2.089930	-0.162194
H	0.441807	1.288966	1.322358
H	1.859672	1.273332	-1.397721
H	2.361804	2.165452	0.025896
H	0.458844	-1.211100	1.398048
N	-0.285934	-0.013008	-0.152370
C	-1.745492	-0.022017	0.331934
H	-1.669423	-0.079640	1.418026
C	-2.474245	-1.247388	-0.210615
H	-2.091274	-2.184822	0.184842
H	-3.521520	-1.178933	0.079361
H	-2.434864	-1.279211	-1.301728
C	-2.460165	1.262677	-0.071468
H	-3.511373	1.164711	0.195411
H	-2.077673	2.141768	0.441541
H	-2.403677	1.424837	-1.150061
H	-0.317480	-0.038625	-1.175189

30

20H+ SCF Done: -409.790459108 A.U.

C	2.858936	-0.002244	0.193298
C	2.102172	-1.250355	-0.253495
C	0.658956	-1.232386	0.229338
C	0.659603	1.241106	0.222359
C	2.106783	1.249179	-0.251122
H	0.118553	-2.102348	-0.126113
H	2.120609	-1.336398	-1.344188
H	2.567189	-2.155652	0.139330
H	2.954103	-0.003944	1.283231
H	3.868780	-0.002987	-0.217725
H	0.129296	2.108610	-0.152624
H	0.598286	1.231332	1.308586
H	2.129862	1.336824	-1.341660
H	2.574140	2.152246	0.144102
H	0.611357	-1.207133	1.315678
N	-0.066400	0.003306	-0.265350
C	-1.611693	-0.001800	-0.009368
C	-2.199622	-1.200680	-0.759152
H	-1.906423	-2.154677	-0.326547
H	-3.285394	-1.141004	-0.701551
H	-1.925535	-1.189172	-1.816190
C	-2.192725	1.289748	-0.591267
H	-3.278412	1.207165	-0.580916
H	-1.931225	2.169244	-0.006603
H	-1.883196	1.443617	-1.626903
C	-1.891167	-0.096834	1.487031
H	-1.452581	0.731966	2.041214

H	-2.970287	-0.050396	1.630659
H	-1.545028	-1.036502	1.914623
H	0.023247	0.001652	-1.284679

32

21H+ SCF Done: -410.993317114 A.U.

N	-0.000885	-0.000652	-0.387304
C	1.346408	-0.675563	-0.056577
C	2.338958	-0.329659	-1.160876
H	2.563551	0.737460	-1.187197
H	3.272217	-0.859561	-0.974931
H	1.973109	-0.636789	-2.142407
C	1.890478	-0.343325	1.324078
H	1.201182	-0.594093	2.124990
H	2.792671	-0.937307	1.469925
H	2.173607	0.705068	1.411199
C	-1.258269	-0.827943	-0.048590
C	-1.463034	-1.862155	-1.149780
H	-0.657013	-2.596833	-1.173396
H	-2.393110	-2.397206	-0.962480
H	-1.542359	-1.393298	-2.132509
C	-1.234887	-1.461702	1.333473
H	-2.196671	-1.951955	1.483666
H	-0.463229	-2.225670	1.420735
H	-1.109859	-0.736374	2.132061
C	-0.088207	1.503146	-0.054758
C	-0.635788	1.806278	1.331180
H	-0.576631	2.884875	1.476031
H	-1.682904	1.522411	1.428466
H	-0.064507	1.337544	2.126850
C	-0.894577	2.191040	-1.150654
H	-0.888922	3.265178	-0.970004
H	-0.462574	2.019212	-2.138336
H	-1.934604	1.862081	-1.158772
H	0.941438	1.849875	-0.115729
H	-2.072539	-0.107834	-0.097360
H	1.130344	-1.740831	-0.107881
H	-0.003498	-0.002092	-1.406775

26

22H+ SCF Done: -332.343185782 A.U.

N	0.202640	-0.607722	-0.318061
C	-1.201458	-0.030658	0.008769
C	-2.177733	-1.149570	-0.361720
H	-2.023732	-2.036242	0.256754
H	-3.196336	-0.801654	-0.197635
H	-2.084203	-1.429863	-1.412777
C	-1.278750	0.288331	1.496175
H	-0.623663	1.111333	1.777707
H	-2.300934	0.585881	1.727384
H	-1.039030	-0.583097	2.106785
C	1.485182	-0.067151	0.310054
C	1.751244	1.375825	-0.083822
H	2.743427	1.649385	0.273038

H	1.738584	1.502950	-1.167880
H	1.041081	2.068328	0.360466
C	2.615117	-1.000546	-0.109994
H	3.534747	-0.686042	0.381482
H	2.419693	-2.034624	0.179341
H	2.779666	-0.960225	-1.188701
H	1.329589	-0.145191	1.384119
C	-1.457350	1.197664	-0.860857
H	-0.803873	2.029867	-0.616230
H	-1.347803	0.963058	-1.921089
H	-2.484452	1.522570	-0.698648
H	0.165754	-1.598797	-0.075154
H	0.315499	-0.586955	-1.334170

23

23H+ SCF Done: -293.013703224 A.U.

N	0.000030	-0.631862	-0.000219
C	1.290461	0.143034	-0.248923
C	2.402740	-0.868252	-0.488415
H	2.164364	-1.545405	-1.310162
H	3.315025	-0.334368	-0.750792
H	2.603466	-1.457533	0.409019
C	-1.290449	0.142947	0.248853
C	-2.402502	-0.868559	0.488454
H	-3.315088	-0.334864	0.750148
H	-2.602688	-1.458458	-0.408698
H	-2.164164	-1.545150	1.310666
C	-1.580248	1.080455	-0.910774
H	-2.535073	1.571528	-0.727042
H	-0.825007	1.857417	-1.017577
H	-1.660839	0.532648	-1.852014
H	-1.110893	0.710567	1.160096
C	1.579955	1.080283	0.910993
H	0.824873	1.857422	1.017608
H	1.660017	0.532214	1.852132
H	2.535014	1.571099	0.727808
H	-0.152910	-1.251997	-0.799403
H	1.111020	0.710814	-1.160087
H	0.152933	-1.252351	0.798698

26

24H+ SCF Done: -791.909762736 A.U.

N	-0.118179	-0.099830	-0.412841
C	-1.311939	0.796626	0.019781
C	-2.334710	0.779123	-1.104312
H	-2.748118	-0.220670	-1.242687
H	-3.152619	1.447725	-0.838684
H	-1.911683	1.129323	-2.047296
C	-1.909921	0.408215	1.360633
H	-1.166070	0.276492	2.140526
H	-2.572040	1.219852	1.662313
H	-2.505304	-0.499446	1.282421
C	1.341032	0.319066	0.020360
C	1.574410	1.702898	-0.594595

H	0.976382	2.483086	-0.129553
H	2.620900	1.956320	-0.432158
H	1.399047	1.709155	-1.671735
C	1.450175	0.341016	1.535061
H	2.491876	0.533106	1.790100
H	0.850808	1.133581	1.978070
H	1.171247	-0.616756	1.973156
H	-0.856341	1.780410	0.090367
Cl	-0.473045	-1.807179	-0.104433
C	2.303908	-0.690512	-0.602252
H	2.229799	-1.670735	-0.137807
H	2.146190	-0.792249	-1.677422
H	3.317483	-0.323280	-0.447843
H	-0.100279	-0.063651	-1.434589

23

25H+ SCF Done: -752.578390376 A.U.

N	-0.008722	0.203054	-0.427222
C	1.316377	-0.609843	-0.388578
C	2.341351	0.143529	-1.218560
H	2.565765	1.113928	-0.774096
H	3.263523	-0.435834	-1.243048
H	2.008680	0.285266	-2.248241
C	1.804240	-0.907373	1.017395
H	1.034189	-1.324689	1.659864
H	2.604099	-1.642478	0.929299
H	2.215724	-0.017316	1.490418
C	-1.312331	-0.617455	-0.509411
C	-1.547908	-1.447519	0.735927
H	-2.503811	-1.956695	0.620457
H	-0.784804	-2.208420	0.880242
H	-1.608724	-0.819933	1.625012
H	1.028802	-1.534248	-0.889663
Cl	-0.067930	1.465057	0.810175
C	-2.470249	0.302217	-0.858035
H	-2.739126	0.945692	-0.021423
H	-2.252743	0.920531	-1.730751
H	-3.332162	-0.319037	-1.099044
H	-1.097038	-1.264742	-1.363162
H	0.024627	0.749299	-1.290705

17

26H+ SCF Done: -1133.48206186 A.U.

N	0.427251	-0.000071	-0.446988
C	-1.104148	0.000023	0.042370
C	-1.715406	-1.259547	-0.560969
H	-1.304709	-2.168409	-0.128301
H	-2.781335	-1.234637	-0.336460
H	-1.607604	-1.289643	-1.646046
C	-1.116082	0.000009	1.556911
H	-2.158670	-0.000165	1.873485
H	-0.640932	-0.889555	1.966828
H	-0.641205	0.889745	1.966742
Cl	1.332730	1.439941	0.004461

C	-1.715451	1.259645	-0.560930
H	-1.304835	2.168487	-0.128136
H	-1.607655	1.289873	-1.645989
H	-2.781367	1.234599	-0.336349
Cl	1.332593	-1.439978	0.004478
H	0.433589	0.000035	-1.473117

29

27H+ SCF Done: -831.231952600 A.U.

N	-0.010999	0.243208	-0.397557
C	1.418787	-0.385610	-0.006243
C	2.406102	0.235963	-0.996921
H	2.483652	1.314880	-0.887996
H	3.389591	-0.188401	-0.800189
H	2.144772	-0.005132	-2.029242
C	1.786912	-0.045432	1.432113
H	1.092925	-0.465759	2.153205
H	2.766012	-0.485351	1.620807
H	1.871755	1.025144	1.598406
C	-1.432224	-0.375330	0.010316
C	-1.483444	-0.610501	1.508664
H	-2.502992	-0.898529	1.764222
H	-0.825862	-1.415433	1.827176
H	-1.245918	0.296361	2.063667
Cl	0.032315	1.985761	-0.045241
C	-2.506745	0.627496	-0.424526
H	-2.541459	1.506109	0.212763
H	-2.378284	0.941078	-1.461130
H	-3.468695	0.123720	-0.346912
C	1.382708	-1.896560	-0.212990
H	0.773856	-2.407147	0.527225
H	1.060631	-2.173372	-1.214499
H	2.406003	-2.250517	-0.089999
C	-1.627589	-1.660640	-0.801918
H	-1.032192	-2.492935	-0.450723
H	-2.674087	-1.944817	-0.699962
H	-1.438779	-1.503600	-1.865276
H	-0.020327	0.226986	-1.420515

51

28H+ SCF Done: -485.014378873 A.U.

C	-2.041166	-1.253259	-0.014918
C	-3.451178	-0.630231	0.030901
C	-3.308987	0.770555	-0.525122
C	-1.968146	1.284570	0.005935
H	-3.816108	-0.601516	1.056577
H	-4.118233	1.434488	-0.226334
N	-1.074664	0.012165	-0.201708
C	-1.886700	-2.132202	-1.254819
H	-0.875406	-2.510028	-1.383497
H	-2.552638	-2.989001	-1.157264
H	-2.175306	-1.591410	-2.158504
C	-1.782799	-2.025788	1.274612
H	-2.591131	-2.751964	1.368071

H	-0.848529	-2.578767	1.285392
H	-1.824835	-1.383752	2.152733
C	-1.516338	2.469349	-0.844129
H	-2.329375	3.196183	-0.837571
H	-0.628645	2.969404	-0.472383
H	-1.352923	2.178004	-1.882323
C	-2.045961	1.646282	1.486867
H	-1.091472	1.982393	1.884163
H	-2.754159	2.467478	1.593335
H	-2.400121	0.822011	2.101730
C	0.277676	-0.077125	0.519408
H	0.013653	-0.232144	1.562165
C	1.151703	1.193923	0.419872
C	1.128626	-1.271221	0.018069
H	0.592061	2.064849	0.744735
C	1.701319	1.416674	-1.000098
C	2.335009	1.014041	1.393976
H	0.550130	-2.188411	0.031290
C	1.669175	-1.034359	-1.402791
C	2.312697	-1.443254	0.990159
H	2.295765	2.333362	-1.004577
H	0.898596	1.569928	-1.723755
C	2.557004	0.216634	-1.425623
H	1.964512	0.880236	2.414193
H	2.931664	1.929293	1.387375
C	3.191110	-0.186667	0.972797
H	0.858391	-0.930377	-2.131537
H	2.236390	-1.916138	-1.709790
H	1.944375	-1.638329	2.001269
H	2.891225	-2.318567	0.685514
H	2.937486	0.378344	-2.436507
C	3.730812	0.040596	-0.448643
H	4.022241	-0.308825	1.670903
H	4.348607	-0.808065	-0.755035
H	4.368468	0.928596	-0.469732
H	-4.149341	-1.238246	-0.541264
H	-3.283699	0.756155	-1.617302
H	-0.862136	0.018541	-1.198837

37

29H+ SCF Done: -526.535101849 A.U.

C	-0.868431	-1.277681	-0.015775
C	-2.233339	-0.656548	0.038555
C	-2.232563	0.659041	0.038131
C	-0.866824	1.278428	-0.015853
H	-3.104893	-1.292038	0.097323
H	-3.103323	1.295655	0.096565
N	0.023303	-0.000229	-0.324905
C	-0.794809	-2.272052	-1.179479
H	0.138092	-2.825562	-1.209068
H	-1.601981	-2.993364	-1.049999
H	-0.949898	-1.777253	-2.139867
C	-0.566623	-1.960251	1.315727

H -1.354182 -2.696516 1.480790
H 0.380382 -2.494263 1.311760
H -0.592278 -1.259380 2.147088
C -0.791286 2.273123 -1.179188
H -1.600035 2.993044 -1.051947
H 0.140697 2.828308 -1.205963
H -0.942826 1.778424 -2.140201
C -0.565453 1.960418 1.316179
H 0.385235 2.487786 1.316107
H -1.348536 2.702406 1.476993
H -0.600059 1.260631 2.148135
C 1.573551 -0.000941 -0.024632
C 2.171208 -1.235169 -0.707991
H 3.255629 -1.162561 -0.640134
H 1.879908 -2.164847 -0.228520
H 1.911004 -1.278810 -1.765852
C 2.170834 1.239959 -0.696303
H 1.881921 2.164944 -0.206438
H 3.255273 1.165246 -0.631638
H 1.908220 1.295301 -1.752805
C 1.943351 -0.007889 1.458758
H 3.032785 0.005285 1.505623
H 1.591050 0.865868 1.993519
H 1.613245 -0.898844 1.979186
H 0.023756 -0.000487 -1.345098
49
30H+ SCF Done: -758.907034700 A.U.
C -2.039944 -1.266210 -0.021576
C -3.393028 -0.658357 -0.260499
C -3.392983 0.658476 -0.260185
C -2.039776 1.266170 -0.021513
H -4.256009 -1.297079 -0.380431
H -4.255906 1.297311 -0.379908
N -1.112921 0.000043 -0.201760
C -1.732012 -2.316346 -1.089815
H -0.782387 -2.819779 -0.935960
H -2.516313 -3.072359 -1.051599
H -1.748441 -1.879382 -2.089355
C -1.978882 -1.844895 1.394001
H -2.759530 -2.602182 1.469502
H -1.030924 -2.326533 1.619391
H -2.184252 -1.089432 2.148972
C -1.732168 2.316316 -1.090038
H -2.516562 3.072205 -1.051724
H -0.782556 2.819854 -0.936437
H -1.748755 1.879062 -2.089438
C -1.978316 1.845030 1.393923
H -1.030292 2.326751 1.618958
H -2.758994 2.602258 1.469639
H -2.183306 1.089655 2.149098
C 0.229057 0.000017 0.541284
H -0.043710 0.000181 1.593930

C	1.088158	1.249048	0.235319
C	1.088184	-1.249033	0.235733
H	0.520323	2.155680	0.414364
C	1.614224	1.242372	-1.210436
C	2.281926	1.244917	1.210715
H	0.520460	-2.155671	0.415091
C	1.614288	-1.242881	-1.210026
C	2.281948	-1.244498	1.211149
H	2.192618	2.154213	-1.376031
H	0.796028	1.267341	-1.935715
C	2.482977	-0.000273	-1.446247
H	1.922515	1.274756	2.243097
H	2.867097	2.153141	1.048632
C	3.146859	0.000176	0.978981
H	0.796085	-1.268203	-1.935291
H	2.192747	-2.154753	-1.375225
H	1.922540	-1.273956	2.243542
H	2.867143	-2.152766	1.049405
H	2.851335	-0.000418	-2.474322
C	3.668555	-0.000077	-0.467377
H	3.986890	0.000295	1.677007
H	4.294212	-0.880127	-0.639728
H	4.294166	0.879945	-0.640047
H	-0.890178	-0.000025	-1.197459

55

31H+ SCF Done: -912.623433853 A.U.

C	1.228731	1.275691	0.129152
C	2.607649	0.689785	-0.045610
C	2.607599	-0.689758	-0.045516
C	1.228537	-1.275488	0.129105
N	0.321867	-0.000056	-0.103835
C	0.948381	2.325791	-0.946613
H	-0.017787	2.806697	-0.836813
H	1.708403	3.101802	-0.868544
H	1.025221	1.895345	-1.946054
C	1.081780	1.837954	1.545001
H	1.826612	2.624963	1.658825
H	0.106246	2.277448	1.733970
H	1.287621	1.085256	2.302649
C	0.948418	-2.325688	-0.946872
H	1.708534	-3.101570	-0.868901
H	-0.017708	-2.806721	-0.837106
H	1.025096	-1.895053	-1.946240
C	1.081498	-1.838030	1.544865
H	0.105809	-2.277181	1.733826
H	1.825996	-2.625380	1.658430
H	1.287671	-1.085583	2.302678
C	-1.064915	-0.000010	0.564383
H	-0.848853	-0.000089	1.630094
C	-1.909727	-1.248081	0.214027
C	-1.909716	1.248058	0.214236
H	-1.356827	-2.156323	0.426013

C	-2.358737	-1.242622	-1.257577
C	-3.154647	-1.242687	1.124292
H	-1.356853	2.156302	0.426403
C	-2.358776	1.242845	-1.257368
C	-3.154624	1.242451	1.124542
H	-2.928640	-2.154176	-1.451803
H	-1.504224	-1.269360	-1.939487
C	-3.212774	0.000132	-1.540425
H	-2.850952	-1.269959	2.174664
H	-3.728127	-2.152474	0.932095
C	-4.006903	-0.000085	0.844924
H	-1.504262	1.269691	-1.939249
H	-2.928706	2.154413	-1.451433
H	-2.850927	1.269510	2.174920
H	-3.728112	2.152276	0.932546
H	-3.524460	0.000206	-2.587059
C	-4.449778	0.000063	-0.627332
H	-4.883364	-0.000128	1.496605
H	-5.065175	0.880143	-0.833132
H	-5.065198	-0.879958	-0.833309
C	3.798073	-1.400215	-0.153012
C	3.798153	1.400145	-0.153361
C	4.991335	-0.697283	-0.264317
C	4.991367	0.697114	-0.264533
H	3.805264	-2.482243	-0.147803
H	5.925260	-1.236505	-0.351739
H	5.925299	1.236277	-0.352256
H	3.805409	2.482178	-0.148485
H	0.153115	0.000093	-1.109413

67

32H+ SCF Done: -1069.92041166 A.U.

C	1.100112	1.302265	0.042276
C	2.465246	0.679718	-0.145240
C	2.434377	-0.697296	-0.232250
C	1.060820	-1.287556	0.002591
N	0.157529	0.020764	-0.185614
C	0.768619	2.302879	-1.090911
H	-0.274835	2.586615	-1.023404
H	0.888743	1.779092	-2.043540
C	1.061648	1.890996	1.476342
H	2.011613	2.420503	1.551094
H	1.155495	1.069235	2.186257
C	0.778482	-2.356438	-1.098755
H	1.760455	-2.715255	-1.396477
H	0.408539	-1.843603	-1.989834
C	0.938441	-1.793625	1.457929
H	-0.041338	-2.238377	1.609853
H	0.997689	-0.938374	2.129337
C	-1.233867	0.054534	0.483238
H	-1.013661	0.126481	1.545291
C	-2.081379	-1.211311	0.231137
C	-2.100076	1.266007	0.056253

H	-1.517033	-2.096305	0.488888
C	-2.575379	-1.312691	-1.223218
C	-3.304534	-1.154085	1.169890
H	-1.555830	2.189478	0.186548
C	-2.586482	1.156637	-1.399237
C	-3.324482	1.321711	0.992761
H	-3.147553	-2.236875	-1.333101
H	-1.744229	-1.382874	-1.928549
C	-3.445732	-0.100865	-1.571630
H	-2.976485	-1.107131	2.212047
H	-3.872894	-2.080043	1.054577
C	-4.175844	0.059241	0.826939
H	-1.750744	1.134134	-2.105296
H	-3.160770	2.054287	-1.640238
H	-3.002938	1.425721	2.032671
H	-3.906119	2.213706	0.748343
H	-3.784171	-0.177039	-2.607165
C	-4.658086	-0.043934	-0.628760
H	-5.033876	0.100783	1.501475
H	-5.285168	0.816639	-0.877389
H	-5.272142	-0.939415	-0.758089
C	3.611160	-1.415993	-0.436890
C	3.683482	1.357263	-0.184829
C	4.820348	-0.741503	-0.518985
C	4.857506	0.643133	-0.377099
H	3.598820	-2.493867	-0.512771
H	5.735370	-1.296860	-0.676574
H	5.802922	1.167812	-0.416463
H	3.731574	2.427176	-0.055131
C	1.593771	3.591690	-1.144026
H	2.603505	3.421528	-1.508871
H	1.111913	4.282913	-1.835853
H	1.654790	4.088043	-0.175817
C	-0.025219	2.858286	1.947022
H	-0.239818	3.636763	1.215130
H	-0.956928	2.365108	2.209388
H	0.334100	3.356146	2.848678
C	2.005140	-2.804766	1.884327
H	2.986905	-2.343040	1.969861
H	1.738734	-3.207306	2.861778
H	2.086337	-3.642955	1.192491
C	-0.062650	-3.607367	-0.825855
H	0.179547	-4.067414	0.130579
H	-1.133711	-3.440081	-0.858879
H	0.166026	-4.340904	-1.600067
H	-0.018077	0.038635	-1.189606

41

33H+ SCF Done: -528.954524181 A.U.

N	-0.214404	-0.119568	-0.265809
C	-1.150028	1.200780	-0.044603
C	-2.620988	0.908506	-0.375686
H	-3.079265	0.187155	0.298050

H	-3.157490	1.848115	-0.248419
H	-2.772149	0.599216	-1.409306
C	-1.098163	1.730242	1.384415
H	-0.127822	2.126909	1.661705
H	-1.806663	2.555859	1.446098
H	-1.405154	0.981143	2.110681
C	1.168224	-0.237325	0.530301
C	2.055773	0.983300	0.204960
H	1.560326	1.898940	0.515789
H	2.913903	0.887637	0.873723
C	1.898312	-1.495196	0.044726
H	1.965730	-1.562238	-1.037519
H	2.916107	-1.441569	0.430371
H	1.454874	-2.406676	0.433328
C	1.001801	-0.337590	2.053836
H	0.159964	-0.946008	2.359662
H	1.901473	-0.812745	2.444085
H	0.921473	0.630691	2.534181
C	-0.668002	2.255271	-1.047383
H	0.334192	2.618719	-0.850528
H	-0.715667	1.879425	-2.071568
H	-1.339486	3.110982	-0.987231
C	-1.047245	-1.426832	-0.449178
C	-1.401922	-2.150474	0.837881
H	-2.156195	-2.898397	0.590322
H	-0.556819	-2.674133	1.278572
H	-1.835275	-1.484628	1.582872
C	-0.506585	-2.361283	-1.534870
H	-1.351256	-2.954955	-1.886640
H	-0.123471	-1.811119	-2.396063
H	0.255790	-3.051630	-1.190367
H	-1.966467	-1.050107	-0.865119
C	2.574425	1.111388	-1.228168
H	3.086354	2.066731	-1.346217
H	3.286531	0.324089	-1.471022
H	1.784866	1.084224	-1.982108
H	0.118816	0.046633	-1.214053

44

34H+ SCF Done: -568.281188048 A.U.

N	0.023451	0.166081	-0.118812
C	1.102748	-0.905143	0.463318
C	2.557471	-0.397402	0.289787
H	2.723200	0.499724	0.886981
H	3.161466	-1.162555	0.780171
C	0.872925	-1.168075	1.946835
H	-0.041430	-1.720228	2.135311
H	1.695829	-1.784429	2.306758
H	0.866429	-0.251881	2.533327
C	-1.529764	0.020636	0.267014
C	-2.058130	-1.376576	-0.117659
H	-1.546879	-2.138482	0.463558
H	-3.084865	-1.387363	0.255271

C	-2.313474	1.058080	-0.547711
H	-2.082875	1.030369	-1.609665
H	-3.370369	0.816166	-0.440848
H	-2.177835	2.069716	-0.179186
C	-1.821854	0.240736	1.758961
H	-1.235121	1.027402	2.213325
H	-2.869222	0.530364	1.841104
H	-1.695486	-0.663207	2.344773
C	0.985715	-2.204174	-0.343587
H	0.090325	-2.772598	-0.127812
H	1.041275	-2.031144	-1.417454
H	1.835457	-2.832359	-0.077847
C	0.582212	1.627922	-0.174624
C	0.328177	2.496455	1.045984
H	0.926143	3.399449	0.915774
H	-0.707370	2.806740	1.154795
H	0.660917	2.020822	1.967004
C	0.265880	2.338451	-1.493346
H	1.028662	3.105519	-1.631405
H	0.333342	1.665211	-2.350289
H	-0.700432	2.830729	-1.512180
H	1.644200	1.468231	-0.202754
C	-2.085323	-1.743159	-1.601071
H	-2.391465	-2.783540	-1.712968
H	-2.796357	-1.130497	-2.153768
H	-1.115472	-1.648324	-2.090736
C	3.094176	-0.232024	-1.135686
H	4.085681	0.219710	-1.099447
H	3.191552	-1.194085	-1.636700
H	2.473681	0.403973	-1.768559
H	0.008309	-0.090362	-1.104137

47

35H+ SCF Done: -607.619526563 A.U.

N	-0.777553	0.175820	0.042752
C	0.210562	-0.963559	0.572331
C	1.630852	-0.387971	0.858974
H	1.566404	0.334624	1.669478
H	2.148475	-1.235125	1.314061
C	-0.344437	-1.503759	1.889670
H	-1.354429	-1.889956	1.782072
H	0.287007	-2.328793	2.217569
H	-0.338586	-0.751157	2.676882
C	-2.293270	-0.158798	-0.400520
C	-2.534990	-1.619057	-0.790573
H	-2.404013	-2.314768	0.032378
H	-3.579819	-1.680415	-1.093812
H	-1.938679	-1.931952	-1.641548
C	-2.543449	0.678141	-1.661805
H	-1.872767	0.368888	-2.465891
H	-3.563207	0.494755	-1.998447
H	-2.437123	1.745636	-1.508941
C	-3.268113	0.191378	0.725379

H	-3.310031	1.251076	0.957699
H	-4.263950	-0.099769	0.393943
H	-3.053325	-0.363998	1.637022
C	0.349686	-2.085564	-0.463768
H	-0.446151	-2.815638	-0.408364
H	0.411097	-1.706524	-1.482925
H	1.276230	-2.614859	-0.256272
C	-0.715440	1.398034	0.954129
C	-1.126538	2.733497	0.353171
H	-0.807031	3.505626	1.054061
H	-0.626198	2.927184	-0.595541
H	-2.197431	2.845119	0.212825
H	0.315157	1.489523	1.258275
H	-1.291342	1.166809	1.842375
C	2.602086	0.182205	-0.224392
C	3.686625	0.941046	0.565274
H	4.178410	0.282014	1.284280
H	4.450124	1.331670	-0.109638
H	3.259504	1.782464	1.115469
C	1.954527	1.163897	-1.212041
H	1.235388	0.674694	-1.875655
H	1.473207	2.002521	-0.708644
H	2.720221	1.585976	-1.865043
C	3.302496	-0.927212	-1.030672
H	3.735057	-1.681842	-0.370582
H	2.634038	-1.428288	-1.728235
H	4.114750	-0.494580	-1.618181
H	-0.341709	0.464674	-0.830871

50

36H+ SCF Done: -646.938716823 A.U.

N	-0.759403	0.036349	-0.140278
C	0.271851	-0.945877	0.620904
C	1.640528	-0.246622	0.861784
H	1.478712	0.635700	1.476980
H	2.147367	-0.938379	1.538381
C	-0.269736	-1.359438	1.995309
H	-1.326269	-1.602161	1.983771
H	0.265912	-2.255251	2.308634
H	-0.099341	-0.602997	2.755861
C	-2.275666	-0.433234	-0.426676
C	-2.449447	-1.956512	-0.478444
H	-2.217770	-2.464140	0.451109
H	-3.505847	-2.131812	-0.678229
H	-1.893256	-2.407984	-1.295176
C	-2.642052	0.072610	-1.831966
H	-1.915942	-0.256548	-2.578001
H	-3.601214	-0.366396	-2.103877
H	-2.751168	1.146985	-1.897499
C	-3.229594	0.134238	0.620889
H	-3.332051	1.212197	0.551823
H	-4.214745	-0.292055	0.435429
H	-2.941114	-0.131465	1.635281

C	0.506176	-2.198653	-0.236211
H	-0.271765	-2.940961	-0.127545
H	0.631637	-1.974936	-1.294306
H	1.426900	-2.659946	0.111541
C	-0.568329	1.532053	0.274740
C	-1.109024	2.537666	-0.735883
H	-0.659530	3.499397	-0.485021
H	-0.822217	2.303075	-1.760587
H	-2.187255	2.663402	-0.683984
H	0.504692	1.625326	0.216342
C	2.686442	0.096416	-0.258117
C	3.580215	1.201485	0.336323
H	4.030100	0.875661	1.276803
H	4.387800	1.455046	-0.352702
H	3.009018	2.111974	0.533705
C	2.109113	0.611064	-1.588207
H	1.473254	-0.124856	-2.084473
H	1.556865	1.544486	-1.481272
H	2.928517	0.816840	-2.278995
C	3.587123	-1.114274	-0.572847
H	3.999293	-1.547049	0.340995
H	3.060462	-1.898406	-1.113979
H	4.424289	-0.797608	-1.198023
C	-0.965389	1.915704	1.698066
H	-0.397353	2.812572	1.948340
H	-2.017792	2.162139	1.794500
H	-0.720977	1.163365	2.437624
H	-0.366660	0.030585	-1.079491

47

37H+ SCF Done: -682.685366770 A.U.

N	1.311156	-0.050689	-0.207512
C	2.021751	-1.410295	-0.093883
C	3.071254	-1.557558	-1.188739
H	3.937655	-0.913648	-1.044003
H	3.424321	-2.588471	-1.177960
H	2.649545	-1.365455	-2.176885
H	1.244395	-2.137161	-0.302513
C	2.563511	-1.649875	1.305051
H	1.793894	-1.544784	2.068887
H	2.938825	-2.671644	1.353000
H	3.391998	-0.984137	1.545386
C	2.293081	1.133349	0.034943
C	-0.026173	0.039284	0.551095
H	0.247224	0.204299	1.588998
C	-0.874396	-1.245899	0.456657
C	-0.867546	1.225954	0.031550
H	-0.311724	-2.102738	0.824191
C	-1.354225	-1.490526	-0.984858
C	-2.096030	-1.067736	1.380187
H	-0.288388	2.145111	0.071571
C	-1.349456	0.974073	-1.408644
C	-2.089600	1.385372	0.955785

H	-1.939372	-2.412192	-1.012887
H	-0.511525	-1.644201	-1.667379
C	-2.204936	-0.300753	-1.458106
H	-1.766351	-0.914346	2.411537
H	-2.680036	-1.990940	1.364130
C	-2.949177	0.115888	0.908629
H	-0.512499	0.894023	-2.111499
H	-1.928833	1.838406	-1.740488
H	-1.762253	1.585930	1.979814
H	-2.667158	2.252630	0.627646
H	-2.539892	-0.476448	-2.482461
C	-3.422350	-0.135779	-0.532550
H	-3.812337	0.232121	1.567603
H	-4.040476	0.698316	-0.875545
H	-4.043413	-1.034813	-0.570581
H	3.271412	0.665614	0.000765
C	2.153908	1.788820	1.401322
H	1.236705	2.363901	1.509195
H	2.219384	1.069536	2.215475
H	2.989573	2.480422	1.510120
C	2.221605	2.120856	-1.123338
H	1.270710	2.648276	-1.168007
H	3.009146	2.862710	-0.992092
H	2.394997	1.627015	-2.081085
H	1.059060	0.016443	-1.192964

62

38H+ SCF Done: -954.355990502 A.U.

N	0.067898	0.921251	-0.145903
C	-0.144693	2.458966	-0.052944
C	-0.956772	3.012310	-1.213432
H	-2.012100	2.766389	-1.164721
H	-0.871250	4.099075	-1.185653
H	-0.559907	2.679404	-2.174378
H	0.868877	2.821516	-0.203098
C	-0.589328	2.955810	1.315035
H	-0.062459	2.458481	2.129045
H	-0.343509	4.016597	1.368633
H	-1.657863	2.859531	1.476513
C	1.508202	0.657180	0.423201
H	1.604940	1.401628	1.209654
C	2.541979	0.937529	-0.688461
C	1.811909	-0.713883	1.052267
H	2.316244	1.883166	-1.185914
C	2.559727	-0.198718	-1.727008
C	3.927341	1.050067	-0.025687
H	1.092827	-0.938897	1.841056
C	1.868616	-1.848136	0.013747
C	3.202310	-0.584089	1.718944
H	3.283355	0.049828	-2.506292
H	1.593536	-0.294722	-2.235578
C	2.929378	-1.526555	-1.048978
H	3.931241	1.870983	0.696353

H	4.669705	1.289358	-0.790484
C	4.275054	-0.275549	0.664684
H	0.902767	-2.005135	-0.467831
H	2.113144	-2.781738	0.525318
H	3.182371	0.197099	2.483725
H	3.428398	-1.523995	2.227332
H	2.949684	-2.323625	-1.795171
C	4.308494	-1.401347	-0.381709
H	5.249411	-0.191060	1.150838
H	4.578288	-2.348275	0.093779
H	5.071812	-1.188819	-1.135207
C	-1.008522	-0.141630	0.256598
H	-0.411624	-0.860162	0.794787
C	-1.564325	-0.861797	-0.988721
C	-2.169137	0.209800	1.205737
H	-0.754290	-1.042908	-1.702339
C	-2.696316	-0.077337	-1.668558
C	-2.114585	-2.218634	-0.502813
H	-1.800285	0.757071	2.070140
C	-3.329350	0.956808	0.528918
C	-2.711061	-1.159052	1.693077
H	-3.052207	-0.651063	-2.527557
H	-2.334346	0.871924	-2.063212
C	-3.846700	0.152051	-0.673627
H	-1.316989	-2.811484	-0.047069
H	-2.484555	-2.783522	-1.361396
C	-3.247648	-1.977994	0.507110
H	-3.040893	1.956785	0.216449
H	-4.129754	1.088187	1.261332
H	-1.926122	-1.713633	2.214356
H	-3.508832	-0.978504	2.416980
H	-4.646291	0.709783	-1.166081
C	-4.381353	-1.202679	-0.183152
H	-3.621038	-2.938250	0.869975
H	-5.209043	-1.046605	0.514059
H	-4.772057	-1.781117	-1.024929
H	0.164212	0.759236	-1.147457

69

39H+ SCF Done: -954.355990502 A.U.

N	0.094764	0.406811	-0.160108
C	1.554066	0.374541	0.420316
H	1.522777	1.117414	1.213558
C	2.535339	0.824360	-0.683643
C	2.070635	-0.936007	1.040488
H	2.164304	1.725817	-1.175580
C	2.734648	-0.287239	-1.729814
C	3.883668	1.149725	-0.014979
H	1.394859	-1.279625	1.824510
C	2.309821	-2.038441	-0.006638
C	3.420514	-0.591876	1.714057
H	3.407496	0.079014	-2.508005
H	1.795355	-0.536109	-2.236980

C	3.310388	-1.544220	-1.061046
H	3.756933	1.955958	0.712757
H	4.580702	1.508604	-0.775730
C	4.434690	-0.109275	0.667383
H	1.382168	-2.340378	-0.495044
H	2.695734	-2.926585	0.498716
H	3.273802	0.171832	2.482479
H	3.790878	-1.487102	2.218382
H	3.459321	-2.321505	-1.813533
C	4.649978	-1.207202	-0.386906
H	5.381579	0.124637	1.158855
H	5.064887	-2.103119	0.082897
H	5.372508	-0.870700	-1.135483
C	-0.802957	-0.806996	0.244431
H	-0.097666	-1.429364	0.770807
C	-1.257245	-1.604105	-0.995197
C	-1.989549	-0.638074	1.210326
H	-0.438919	-1.657431	-1.720350
C	-2.508089	-1.008385	-1.658015
C	-1.580514	-3.029300	-0.500258
H	-1.693845	-0.036815	2.067877
C	-3.260848	-0.081901	0.547786
C	-2.305170	-2.072734	1.706191
H	-2.781964	-1.631164	-2.513008
H	-2.306450	-0.013498	-2.054972
C	-3.664768	-0.961832	-0.645253
H	-0.692096	-3.487574	-0.057590
H	-1.870655	-3.648122	-1.352267
C	-2.721894	-2.967978	0.527209
H	-3.131653	0.947462	0.224707
H	-4.061980	-0.069460	1.291040
H	-1.436609	-2.496586	2.217907
H	-3.112622	-2.017503	2.439735
H	-4.549478	-0.537116	-1.124714
C	-3.973221	-2.382083	-0.146373
H	-2.934161	-3.974468	0.894760
H	-4.804581	-2.355323	0.563374
H	-4.280977	-3.017194	-0.981652
C	-0.352743	1.889559	-0.087531
C	-1.239363	2.319048	-1.252188
C	-0.879920	2.362089	1.263709
H	0.594077	2.404945	-0.241458
C	-1.348254	3.851345	-1.255970
H	-2.235851	1.891097	-1.172722
H	-0.810937	1.976443	-2.198044
C	-0.965036	3.895431	1.243623
H	-1.872775	1.967392	1.458153
H	-0.230685	2.029307	2.074961
C	-1.843992	4.385564	0.090119
H	-2.016852	4.159741	-2.061350
H	-0.365964	4.280855	-1.478855
H	-1.360144	4.242716	2.199723

H	0.040367	4.317295	1.144447
H	-1.865477	5.477043	0.072336
H	-2.874217	4.052746	0.255316
H	0.224341	0.254683	-1.160174

54

40H+ SCF Done: -799.465897242 A.U.

N	-0.521324	0.565377	-0.286365
C	-0.718931	2.095765	-0.308382
C	-1.788736	2.511634	-1.308379
H	-2.795145	2.264652	-0.978383
H	-1.733432	3.595165	-1.415522
H	-1.619450	2.074112	-2.293054
H	0.229363	2.440750	-0.708324
C	-0.911006	2.735296	1.063618
H	-0.295243	2.280769	1.836767
H	-0.610801	3.780204	0.980438
H	-1.945546	2.716228	1.392384
C	0.716710	0.099822	0.516270
H	0.361087	0.048709	1.545038
C	1.925984	1.055773	0.435709
C	1.175243	-1.303444	0.050317
H	1.645214	2.059750	0.745798
C	2.533381	1.088781	-0.979228
C	2.987357	0.540027	1.429768
H	0.343668	-2.006656	0.060541
C	1.779962	-1.254538	-1.364220
C	2.240069	-1.805660	1.043859
H	3.375991	1.783764	-0.982482
H	1.820193	1.470996	-1.716899
C	2.995564	-0.318141	-1.384012
H	2.578531	0.531990	2.444015
H	3.829753	1.235612	1.429577
C	3.451976	-0.865372	1.028007
H	1.043317	-0.929816	-2.108153
H	2.067715	-2.266986	-1.655829
H	1.815883	-1.865813	2.049909
H	2.537035	-2.817592	0.758770
H	3.419624	-0.288633	-2.389931
C	4.051885	-0.823661	-0.387163
H	4.201482	-1.221014	1.738393
H	4.392755	-1.820753	-0.678996
H	4.924915	-0.165628	-0.404712
H	-0.298986	0.346794	-1.258167
C	-1.739138	-0.337160	0.028779
C	-2.677834	0.175561	1.115048
C	-2.481882	-0.722714	-1.250954
H	-1.286784	-1.245004	0.417670
C	-3.720971	-0.908806	1.422253
H	-3.197993	1.074205	0.785435
H	-2.122595	0.422669	2.019858
C	-3.538337	-1.788480	-0.935217
H	-2.968932	0.147380	-1.690583

H	-1.775187	-1.110141	-1.990556
C	-4.490889	-1.318835	0.165967
H	-4.404207	-0.534967	2.186675
H	-3.220089	-1.784612	1.847756
H	-4.089499	-2.028163	-1.846069
H	-3.037945	-2.708731	-0.616514
H	-5.206493	-2.108192	0.404299
H	-5.071384	-0.463777	-0.196836

41

41H+ SCF Done: -1063.59826443 A.U.

N	1.300287	0.252407	-0.294959
C	2.594246	-0.613816	0.018162
C	3.728857	-0.066609	-0.846638
H	4.057389	0.918852	-0.526873
H	4.575080	-0.746327	-0.756663
H	3.449548	-0.024593	-1.900861
C	2.912929	-0.506247	1.502512
H	2.127141	-0.922224	2.131314
H	3.819501	-1.081597	1.686178
H	3.103439	0.521689	1.804466
C	-0.027377	-0.273283	0.333290
H	0.323172	-0.938182	1.115958
C	-0.817681	-1.066003	-0.727723
C	-0.946649	0.774797	0.983676
H	-0.167073	-1.769847	-1.244179
C	-1.485820	-0.127387	-1.747118
C	-1.899816	-1.858577	0.035187
H	-0.377200	1.366193	1.700489
C	-1.649182	1.682388	-0.042566
C	-2.024035	-0.033104	1.744523
H	-2.017010	-0.736816	-2.481280
H	-0.743112	0.446976	-2.312758
C	-2.452278	0.829178	-1.032803
H	-1.434468	-2.546593	0.746178
H	-2.456385	-2.466278	-0.681553
C	-2.840452	-0.885951	0.760055
H	-0.937421	2.306742	-0.581739
H	-2.311287	2.362450	0.498530
H	-1.559110	-0.666984	2.504376
H	-2.674903	0.669706	2.268786
H	-2.930382	1.478470	-1.769068
C	-3.520386	0.024359	-0.275473
H	-3.594870	-1.452122	1.310415
H	-4.214543	0.706675	0.222208
H	-4.105350	-0.578510	-0.975510
Cl	1.637664	1.970913	0.009955
C	2.292197	-2.057457	-0.388357
H	1.497485	-2.510892	0.198808
H	2.059461	-2.145432	-1.449532
H	3.198635	-2.632471	-0.202599
H	1.182659	0.235007	-1.309977

50

42H+ SCF Done: -722.000102280 A.U.

N	1.258252	0.152804	-0.252084
C	2.272808	-1.081586	-0.034466
C	3.721140	-0.658995	-0.317105
H	4.132249	0.003869	0.442067
H	4.322274	-1.567196	-0.303255
H	3.840044	-0.209070	-1.303157
C	2.195322	-1.602792	1.399373
H	1.233073	-2.031604	1.660641
H	2.939010	-2.392809	1.501282
H	2.442517	-0.829737	2.124472
C	-0.096507	0.032428	0.501053
H	0.192769	0.053728	1.548377
C	-0.877555	-1.272564	0.216712
C	-1.061076	1.212343	0.227927
H	-0.252712	-2.141130	0.384673
C	-1.444469	-1.302186	-1.213229
C	-2.041409	-1.357752	1.225830
H	-0.566147	2.159464	0.403709
C	-1.630415	1.174147	-1.200527
C	-2.222700	1.115622	1.238588
H	-1.956894	-2.254696	-1.367427
H	-0.647965	-1.260308	-1.961564
C	-2.411041	-0.128207	-1.417683
H	-1.652049	-1.361922	2.247878
H	-2.558122	-2.308707	1.075816
C	-3.004292	-0.183984	1.024533
H	-0.840445	1.260956	-1.950676
H	-2.280072	2.041235	-1.341750
H	-1.835331	1.164250	2.260259
H	-2.872596	1.982995	1.100607
H	-2.807203	-0.153055	-2.435132
C	-3.565267	-0.219543	-0.406362
H	-3.821588	-0.247691	1.746388
H	-4.259804	0.611401	-0.557751
H	-4.127790	-1.143769	-0.564758
C	1.924530	-2.154313	-1.074830
H	0.948336	-2.602770	-0.943755
H	1.988905	-1.749240	-2.086522
H	2.660655	-2.953315	-0.997462
C	1.946592	1.539499	-0.088024
H	2.973812	1.339564	-0.344750
C	1.941813	2.052480	1.344595
H	0.958863	2.351601	1.699911
H	2.355685	1.323774	2.039148
H	2.584384	2.932586	1.376867
C	1.490106	2.556972	-1.132232
H	0.510463	2.986628	-0.952386
H	2.214665	3.371954	-1.119992
H	1.508199	2.129102	-2.136345
H	1.039576	0.094870	-1.245752

5

NH3H+ SCF Done: -57.0257019791 A.U.
N 0.000000 0.000000 0.000130
H 0.000000 0.963051 0.339953
H 0.834026 -0.481525 0.339953
H -0.834026 -0.481525 0.339953
H 0.000000 0.000000 -1.020767
14
NMe3H+ SCF Done: -175.002430567 A.U.
N 0.000321 -0.000008 -0.339654
C -0.821962 1.172275 0.103634
H -0.372037 2.082213 -0.283258
H -0.834012 1.189831 1.190178
C 1.426621 0.125340 0.103650
H 1.989854 -0.717819 -0.286124
H 1.448591 0.123139 1.190193
C -0.604744 -1.297625 0.103652
H -0.619016 -1.314606 1.190223
H -1.616036 -1.363543 -0.287640
H 0.000287 -2.114774 -0.278601
H -1.830868 1.057755 -0.282575
H 1.831350 1.057703 -0.279530
H 0.000152 0.000214 -1.360903
23
NEt3H+ SCF Done: -293.001404450 A.U.
N 0.001849 0.000720 0.003384
C -0.461540 1.373785 0.447933
H 0.399714 2.033772 0.387635
H -0.740186 1.271571 1.495136
C -1.597828 1.907279 -0.402693
H -2.490590 1.285330 -0.350767
H -1.864393 2.899297 -0.039240
H -1.296462 2.004619 -1.446887
C 1.421471 -0.282857 0.452807
H 1.558757 -1.359529 0.406412
H 1.472558 0.021890 1.496717
C 2.453768 0.421611 -0.406012
H 2.351099 1.505830 -0.381030
H 3.445164 0.174178 -0.027341
H 2.398766 0.086607 -1.443100
C -0.955387 -1.085810 0.452015
H -0.719766 -1.280410 1.497384
H -1.956627 -0.666523 0.401598
C -0.861264 -2.334520 -0.403777
H 0.124281 -2.797271 -0.366555
H -1.582333 -3.062010 -0.031976
H -1.111143 -2.120275 -1.444228
H 0.002909 0.000961 -1.019087
12
PyH+ SCF Done: -248.827391169 A.U.
C -0.663721 1.182330 -0.000199
C 0.713927 1.204900 -0.000129
C 1.409660 -0.000219 0.000061

C	0.713550	-1.205122	0.000202
C	-0.664099	-1.182128	0.000136
N	-1.303507	0.000204	-0.000065
H	2.490922	-0.000378	0.000130
H	-1.280072	2.067938	-0.000350
H	1.228413	2.153905	-0.000229
H	1.227741	-2.154287	0.000359
H	-1.280745	-2.067540	0.000227
H	-2.317615	0.000370	-0.000103

30

1Au SCF Done: -967.308478776 A.U.

N	-1.297545	0.044279	0.163804
C	-1.771287	-0.105849	1.598470
C	-0.719187	0.087758	2.679257
H	-2.184946	-1.102668	1.709251
H	-2.599871	0.586188	1.750542
H	-1.209861	0.034778	3.654106
H	0.034873	-0.699084	2.640017
H	-0.213427	1.048616	2.606394
C	-1.760500	1.399119	-0.368313
C	-1.464838	1.590521	-1.853891
H	-2.844279	1.422383	-0.228835
H	-1.772638	2.596537	-2.140047
H	-0.396715	1.495054	-2.059001
H	-2.003659	0.893218	-2.492056
C	-1.870869	-1.094003	-0.688515
C	-1.375012	-2.466227	-0.234044
H	-1.461727	-0.925187	-1.681183
H	-1.645636	-3.197970	-0.996098
H	-0.290959	-2.486900	-0.118483
H	-1.827994	-2.790022	0.703337
C	-3.398580	-1.083024	-0.783746
H	-3.711306	-1.923181	-1.404594
H	-3.868974	-1.203165	0.192823
H	-3.787572	-0.176429	-1.244118
C	-1.172130	2.558370	0.433736
H	-1.432602	2.517970	1.488996
H	-0.084568	2.589092	0.345690
H	-1.571406	3.491848	0.035915
Au	0.877192	-0.062208	-0.042208
Cl	3.167786	-0.169801	-0.310151

19

2Au SCF Done: -848.124306722 A.U.

N	1.188129	-0.000041	-0.344393
C	4.038195	0.000069	0.403684
C	3.344638	-1.255328	-0.128158
C	1.851130	-1.235892	0.180625
C	1.851050	1.235864	0.180615
C	3.344548	1.255396	-0.128199
H	1.342521	-2.094469	-0.254120
H	3.486750	-1.326345	-1.211963
H	3.779045	-2.157761	0.307213

H	3.998442	0.000088	1.498068
H	5.093201	0.000101	0.123753
H	1.342361	2.094410	-0.254096
H	1.676850	1.254221	1.257078
H	3.486631	1.326379	-1.212014
H	3.778911	2.157876	0.307115
H	1.676905	-1.254286	1.257082
H	1.314220	-0.000048	-1.355491
Au	-0.907004	-0.000018	-0.064036
Cl	-3.189221	0.000050	0.232679

31

3Au SCF Done: -1005.45531568 A.U.

N	0.949470	-0.000031	-0.395572
C	3.825093	-0.000254	0.156278
C	3.124205	-1.246330	-0.370896
C	1.629212	-1.315634	-0.007638
C	1.629465	1.315684	-0.007583
C	3.124435	1.245987	-0.370839
H	3.218940	-1.267889	-1.461855
H	3.604833	-2.152756	0.002362
H	3.845203	-0.000271	1.248282
H	4.867533	-0.000330	-0.168302
H	3.219131	1.267572	-1.461801
H	3.605307	2.152284	0.002428
H	0.991634	0.000032	-1.414249
C	1.432457	1.642847	1.474150
H	1.763764	2.666880	1.650322
H	0.380483	1.574012	1.751985
H	2.003490	0.996824	2.134263
C	0.971076	2.416137	-0.846193
H	0.997190	2.175563	-1.911278
H	-0.067591	2.575892	-0.554457
H	1.513032	3.351342	-0.701611
C	0.970547	-2.416042	-0.846171
H	-0.068305	-2.575138	-0.554725
H	0.997092	-2.175678	-1.911303
H	1.511994	-3.351475	-0.701222
C	1.432269	-1.642843	1.474146
H	2.003878	-0.997260	2.134219
H	0.380396	-1.573397	1.752198
H	1.763006	-2.667081	1.650152
Au	-1.178337	0.000052	-0.080755
Cl	-3.465957	-0.000019	0.219512

22

4Au SCF Done: -887.448306561 A.U.

N	-1.232723	0.863280	0.000013
C	-2.942858	-1.555254	-0.000006
C	-2.212999	-1.078710	-1.256143
C	-1.981661	0.425355	-1.222087
C	-1.981383	0.425148	1.222248
C	-2.212758	-1.078915	1.256085
H	-1.416197	0.758926	-2.090828

H	-1.251993	-1.591941	-1.348675
H	-2.789056	-1.314243	-2.153321
H	-3.961968	-1.154601	0.000121
H	-3.028278	-2.643230	-0.000082
H	-1.415671	0.758543	2.090888
H	-2.945064	0.949500	1.223878
H	-1.251783	-1.592228	1.348406
H	-2.788707	-1.314530	2.153316
H	-2.945348	0.949692	-1.223366
C	-1.142264	2.347862	0.000141
H	-0.606365	2.678616	0.886667
H	-0.606245	2.678762	-0.886262
H	-2.145341	2.785205	0.000097
Au	0.768954	0.087691	-0.000028
Cl	2.933956	-0.695995	-0.000010

34

5Au SCF Done: -1044.75791926 A.U.

N	1.003163	0.000001	-0.588620
C	2.201286	-0.000082	2.099480
C	1.575383	1.253597	1.501939
C	1.629834	1.309973	-0.033268
C	1.629815	-1.309954	-0.033314
C	1.575270	-1.253668	1.501876
H	0.529445	1.317986	1.815862
H	2.072725	2.147373	1.884429
H	3.282095	-0.000119	1.941930
H	2.054647	-0.000098	3.181525
H	0.529313	-1.318024	1.815744
H	2.072564	-2.147501	1.884298
C	1.012222	0.000029	-2.079841
H	0.490123	-0.875865	-2.447892
H	0.489797	0.875733	-2.447863
H	2.022917	0.000227	-2.484694
C	3.073620	-1.571420	-0.505203
H	3.811495	-0.941941	-0.021979
H	3.321355	-2.603440	-0.256141
H	3.184126	-1.467689	-1.582967
C	0.797693	-2.515081	-0.501749
H	0.933499	-2.729068	-1.560988
H	1.127016	-3.394074	0.052703
H	-0.266761	-2.377702	-0.306536
H	3.183643	1.468470	-1.583237
C	3.073517	1.571518	-0.505350
H	3.321370	2.603375	-0.255710
H	3.811483	0.941654	-0.022801
C	0.797622	2.515090	-0.501547
H	-0.266784	2.377667	-0.306116
H	1.127017	3.394093	0.052838
H	0.933226	2.729090	-1.560808
Au	-1.151678	0.000003	-0.076256
Cl	-3.411850	-0.000023	0.393812

40

6Au SCF Done: -1198.42348702 A.U.

N	0.743997	0.569134	0.018837
C	0.878839	1.298924	1.416061
C	0.494500	0.284921	2.506619
H	0.620876	0.760260	3.479690
H	-0.546334	-0.030519	2.421155
H	1.127297	-0.599818	2.482846
C	0.839447	1.421261	-1.322327
C	-0.474973	2.169214	-1.611841
H	-0.338051	2.738450	-2.532179
H	-1.304466	1.481223	-1.779496
H	-0.759471	2.869492	-0.836862
C	1.960804	2.470860	-1.358773
H	1.957092	2.918136	-2.353405
H	1.796628	3.275556	-0.646397
H	2.952399	2.058227	-1.206653
C	1.691597	-0.576533	-0.018839
C	1.212660	-1.881209	0.069438
C	3.072759	-0.404380	-0.143952
C	2.068651	-2.975862	0.036424
C	3.932106	-1.495142	-0.183333
H	3.502956	0.576879	-0.222339
C	3.437773	-2.790090	-0.091892
H	1.653572	-3.972680	0.109024
H	4.995665	-1.322698	-0.287282
H	4.108787	-3.638634	-0.121932
C	2.294153	1.797242	1.767785
H	2.720435	2.467447	1.030201
H	2.216233	2.354685	2.701785
H	2.984885	0.976715	1.943757
C	-0.083536	2.486198	1.511029
H	-1.103332	2.207272	1.247490
H	-0.093979	2.829218	2.545666
H	0.227694	3.326988	0.895895
C	1.041705	0.434968	-2.485071
H	2.037183	-0.001141	-2.503417
H	0.310129	-0.373041	-2.451561
H	0.897242	0.977522	-3.419291
Au	-1.381901	-0.245814	-0.008866
Cl	-3.549898	-1.048987	-0.037859
H	0.148088	-2.053643	0.167536

41

7Au SCF Done: -1290.70526649 A.U.

N	0.355887	0.670339	0.045291
C	0.386661	1.417827	1.442605
C	0.067533	0.379503	2.530780
H	0.114025	0.873760	3.501285
H	-0.933579	-0.038033	2.415796
H	0.785447	-0.438254	2.542156
C	0.410071	1.519511	-1.303709
C	-0.964260	2.128285	-1.634261
H	-0.860272	2.703133	-2.555244

H	-1.714861	1.358833	-1.818877
H	-1.338991	2.800135	-0.872710
C	1.421107	2.675896	-1.316892
H	1.394305	3.118985	-2.312902
H	1.161858	3.461081	-0.611260
H	2.447470	2.368720	-1.144797
C	1.403973	-0.377319	0.046640
C	2.758756	-0.070188	-0.054377
C	1.056415	-1.722581	0.154199
C	3.723512	-1.080355	-0.054756
H	3.096530	0.944744	-0.144958
C	2.013228	-2.730885	0.160394
H	0.012036	-1.995764	0.236235
C	3.357631	-2.423802	0.054272
H	4.111566	-3.198345	0.053965
H	1.696714	-3.761275	0.246315
C	1.741920	2.044159	1.822369
H	2.125792	2.742494	1.087723
H	1.590670	2.601759	2.746986
H	2.499479	1.292108	2.029821
C	-0.683259	2.510885	1.498268
H	-1.665531	2.134573	1.214633
H	-0.751096	2.862705	2.527639
H	-0.436593	3.370079	0.879681
C	0.743518	0.548840	-2.448945
H	1.781061	0.223850	-2.445073
H	0.102790	-0.333214	-2.421923
H	0.562205	1.063039	-3.392379
C	5.102352	-0.720246	-0.169114
N	6.213434	-0.427157	-0.260752
Au	-1.685489	-0.345060	-0.034309
Cl	-3.755743	-1.364402	-0.112253

41

8Au SCF Done: -1290.69089082 A.U.

N	-0.738309	-0.659346	0.091744
C	-0.876506	-1.189558	1.585888
C	-0.524043	-0.031489	2.532188
H	-0.599038	-0.393966	3.557346
H	0.493204	0.328007	2.379083
H	-1.211225	0.806517	2.434530
C	-0.799161	-1.729105	-1.115445
C	0.556895	-2.439173	-1.301668
H	0.422730	-3.204456	-2.067435
H	1.327230	-1.758870	-1.663304
H	0.920825	-2.934458	-0.410630
C	-1.839756	-2.854698	-0.975575
H	-1.795925	-3.441944	-1.893194
H	-1.609302	-3.529292	-0.155240
H	-2.866657	-2.523549	-0.875927
C	-1.736595	0.420954	-0.108954
C	-1.432981	1.796596	-0.037961
C	-3.072322	0.102423	-0.360339

C	-2.427954	2.765477	-0.264710
C	-4.050596	1.058438	-0.582127
H	-3.372821	-0.925551	-0.395687
C	-3.729976	2.406832	-0.547179
H	-2.148805	3.807844	-0.203773
H	-5.064192	0.737586	-0.782576
H	-4.479301	3.165993	-0.724181
C	-2.291178	-1.658066	1.970835
H	-2.669706	-2.463069	1.351592
H	-2.237998	-2.036114	2.992140
H	-3.009988	-0.842156	1.971315
C	0.106726	-2.332005	1.854187
H	1.129807	-2.055951	1.601367
H	0.080135	-2.550955	2.921529
H	-0.156424	-3.247673	1.331893
C	-1.053736	-0.949169	-2.415346
H	-2.082489	-0.619454	-2.529251
H	-0.398565	-0.080140	-2.487804
H	-0.823357	-1.607452	-3.252791
C	-0.178607	2.387714	0.329813
N	0.670541	3.095522	0.666137
Au	1.383565	0.120611	-0.083981
Cl	3.613138	0.720973	-0.303018

41

9Au SCF Done: -1290.70530252 A.U.

N	0.115631	0.936965	0.018526
C	-0.066452	1.669035	1.413717
C	-0.011747	0.593237	2.510680
H	-0.112307	1.084120	3.478778
H	-0.824441	-0.127983	2.415288
H	0.932984	0.053155	2.510433
C	-0.132120	1.745767	-1.334281
C	-1.635302	1.881237	-1.635518
H	-1.738358	2.451352	-2.559604
H	-2.104165	0.910832	-1.802574
H	-2.189897	2.404913	-0.867436
C	0.457226	3.163551	-1.374245
H	0.273555	3.563130	-2.372083
H	-0.027306	3.833424	-0.668355
H	1.530415	3.199878	-1.219602
C	1.443792	0.280941	-0.001250
C	1.540882	-1.104239	0.118376
C	2.633984	1.003206	-0.133882
C	2.763250	-1.755326	0.106702
H	0.641421	-1.696556	0.225483
C	3.864431	0.370080	-0.153506
H	2.623906	2.072235	-0.233153
C	3.940121	-1.019147	-0.031915
H	2.800206	-2.831368	0.202393
H	4.766458	0.955703	-0.263885
C	1.021043	2.703066	1.761590
H	1.137482	3.484665	1.019819

H	0.719399	3.185350	2.691766
H	1.986739	2.239213	1.946370
C	-1.429950	2.360105	1.490005
H	-2.244989	1.685055	1.231158
H	-1.585478	2.682779	2.519417
H	-1.485314	3.246141	0.862635
C	0.468557	0.918940	-2.483287
H	1.555537	0.933558	-2.497172
H	0.134229	-0.118334	-2.442407
H	0.120049	1.343695	-3.424338
C	5.207768	-1.677312	-0.050336
N	6.231331	-2.207430	-0.064714
Au	-1.498101	-0.675779	-0.012194
Cl	-3.140104	-2.298471	-0.040201

53

11Au SCF Done: -1506.04937719 A.U.

N	0.187121	0.240204	0.820911
C	0.171396	-0.038096	2.345150
C	1.145939	-1.153061	2.712150
H	2.181322	-0.830759	2.601472
H	0.984135	-1.424906	3.755757
H	0.995861	-2.045446	2.104452
H	-0.838868	-0.395342	2.533589
C	0.422113	1.174704	3.243319
H	-0.255476	2.004788	3.053641
H	0.261335	0.857053	4.274447
H	1.448264	1.531766	3.168944
C	1.574066	0.501427	0.364321
C	2.287920	-0.470982	-0.320155
C	2.219137	1.716298	0.625466
C	3.600974	-0.267903	-0.734049
H	1.817840	-1.420282	-0.542685
C	3.521953	1.935554	0.218680
H	1.712579	2.518686	1.137804
C	4.231239	0.944329	-0.464238
H	4.107697	-1.059867	-1.264309
H	4.008031	2.880613	0.421649
O	5.502783	1.255191	-0.821357
C	6.272468	0.279286	-1.520885
H	7.241966	0.736946	-1.699749
H	6.402197	-0.625873	-0.921841
H	5.809948	0.021150	-2.477115
C	-0.685704	1.452581	0.478793
H	-0.209678	2.279243	1.000831
C	-2.146005	1.438021	0.987547
C	-0.689575	1.843098	-1.016927
H	-2.189513	1.099891	2.022348
C	-3.093062	0.587451	0.126469
C	-2.626877	2.905998	0.928308
H	0.322878	1.779650	-1.415450
C	-1.636722	0.984960	-1.869932
C	-1.169926	3.308318	-1.080137

H	-4.104157	0.665620	0.536267
H	-2.822229	-0.470146	0.170522
C	-3.070303	1.079721	-1.327922
H	-1.996108	3.537077	1.561689
H	-3.642857	2.966522	1.327289
C	-2.600482	3.411122	-0.524296
H	-1.308531	-0.057211	-1.890691
H	-1.599025	1.342489	-2.902964
H	-0.493207	3.953465	-0.511931
H	-1.145611	3.652707	-2.117451
H	-3.732547	0.456422	-1.933868
C	-3.539565	2.543280	-1.379039
H	-2.930335	4.453039	-0.553267
H	-3.542185	2.901040	-2.413037
H	-4.565435	2.622098	-1.006710
Au	-0.606972	-1.633612	-0.041049
Cl	-1.437265	-3.626747	-0.865175

37

12Au SCF Done: -1084.03303658 A.U.

N	1.084338	-0.007677	0.134995
C	1.573985	1.461062	-0.234577
C	1.147276	2.426623	0.886882
H	1.701527	2.276186	1.808494
H	1.348804	3.441004	0.543595
H	0.081195	2.356249	1.097944
C	3.097748	1.576055	-0.379040
H	3.479508	1.062152	-1.256833
H	3.343284	2.632506	-0.492439
H	3.627943	1.217358	0.501796
C	1.659795	-1.208330	-0.734331
C	1.611521	-0.902217	-2.233160
H	2.332637	-0.151274	-2.541743
H	1.857695	-1.819186	-2.768485
H	0.613559	-0.597525	-2.545737
C	0.791914	-2.463508	-0.524941
H	-0.245381	-2.296449	-0.816344
H	1.189692	-3.254643	-1.161510
H	0.816705	-2.826154	0.495914
C	3.095790	-1.607600	-0.342467
H	3.412090	-2.401599	-1.019772
H	3.816120	-0.802980	-0.431993
H	3.143776	-2.010772	0.667299
C	0.912186	1.996223	-1.516456
H	1.195781	1.471215	-2.417991
H	-0.175524	1.984571	-1.437479
H	1.220766	3.035146	-1.637785
C	1.451845	-0.267780	1.552496
C	0.641893	0.147951	2.745430
C	0.796988	-1.306469	2.419888
H	2.517385	-0.174543	1.709114
H	-0.337745	0.574199	2.583600
H	1.186342	0.576364	3.576501

H 1.443929 -1.933916 3.018601
H -0.091709 -1.797038 2.052086
Au -1.110623 0.000594 -0.072183
Cl -3.412656 0.019639 -0.271040
36
13Au SCF Done: -1045.94627478 A.U.
N 1.062677 0.088802 0.005913
C 1.558020 -1.293194 -0.533923
C 1.285787 -1.379440 -2.047989
H 1.909754 -0.727036 -2.650878
H 1.488684 -2.400955 -2.367632
H 0.238950 -1.168750 -2.271745
C 3.045535 -1.564965 -0.250493
H 3.218191 -1.757925 0.806500
H 3.337338 -2.464943 -0.793297
H 3.703328 -0.762869 -0.565683
C 1.425078 0.471575 1.454649
C 1.012803 -0.549642 2.515740
H 1.709790 -1.383071 2.588621
H 1.017880 -0.038914 3.479567
H 0.007596 -0.935742 2.355329
C 2.859022 0.939697 1.745112
H 2.904147 1.190525 2.806322
H 3.613015 0.181592 1.557936
H 3.127296 1.838931 1.200916
C 0.768139 -2.465142 0.083205
H 0.899621 -2.562161 1.152808
H -0.298454 -2.389938 -0.130465
H 1.131620 -3.387184 -0.372225
C 1.366992 1.255623 -0.966865
C 2.809068 1.453991 -1.463665
H 2.783057 2.274220 -2.183440
H 3.508368 1.731683 -0.681494
H 3.209598 0.591798 -1.985747
C 0.835331 2.597788 -0.453373
H 0.831877 3.297405 -1.289778
H -0.188338 2.525527 -0.084529
H 1.459325 3.028309 0.328919
H 0.776491 1.011065 -1.844865
H 0.791525 1.337609 1.626778
Au -1.158173 0.037239 -0.007704
Cl -3.465788 0.035858 -0.035829
39
14Au SCF Done: -1045.94627478 A.U.
N -1.029823 -0.012547 -0.074608
C -1.527544 -0.960430 1.085714
C -1.375462 -0.310696 2.470880
H -2.036721 0.531197 2.642113
H -1.628565 -1.066777 3.213496
H -0.346526 -0.007021 2.656120
C -2.984112 -1.427351 0.914919
H -3.079889 -2.153653 0.111917

H	-3.288420	-1.929195	1.834082
H	-3.683171	-0.618236	0.737689
C	-1.326138	-0.531624	-1.513888
C	-0.762523	-1.912724	-1.863960
H	-1.396227	-2.728833	-1.521983
H	-0.724068	-1.971762	-2.952931
H	0.250292	-2.063699	-1.494697
C	-2.764258	-0.468630	-2.057810
H	-2.698042	-0.665814	-3.129718
H	-3.421746	-1.220798	-1.633626
H	-3.236817	0.500075	-1.950116
C	-0.660378	-2.235229	1.179020
H	-0.712865	-2.869825	0.306406
H	0.385650	-1.997460	1.374659
H	-1.027113	-2.820251	2.023319
C	-1.445615	1.526273	0.033185
C	-0.592541	2.288883	1.071243
H	-0.936481	3.323973	1.083320
H	-0.669444	1.914734	2.081095
H	0.460500	2.299028	0.788899
C	-2.925638	1.768104	0.386515
H	-3.109418	2.841202	0.322116
H	-3.619371	1.283213	-0.290467
H	-3.171557	1.464909	1.399586
C	-1.145052	2.250001	-1.297634
H	-1.271650	3.317919	-1.123018
H	-0.112273	2.096257	-1.612849
H	-1.810145	1.982660	-2.113369
Au	1.193667	0.001587	-0.025552
Cl	3.505758	0.026161	-0.031870
H	-0.749196	0.156383	-2.118457

42

15Au SCF Done: -1124.56555587 A.U.

N	0.992022	-0.004066	-0.002072
C	1.416690	0.336961	-1.517392
C	1.227087	1.826568	-1.863409
H	1.944342	2.488570	-1.393551
H	1.376702	1.920475	-2.938413
H	0.216466	2.165601	-1.650223
C	2.877676	-0.015995	-1.860173
H	3.071710	-1.082571	-1.882141
H	3.081134	0.361511	-2.862912
H	3.594810	0.448075	-1.192989
C	1.393462	-1.500348	0.456538
C	1.185362	-2.551945	-0.652972
H	1.846714	-2.454403	-1.504858
H	1.396543	-3.521705	-0.203370
H	0.152198	-2.579050	-0.990950
C	0.459543	-2.037202	1.571640
H	-0.553157	-2.180249	1.194476
H	0.836627	-3.018527	1.863729
H	0.400223	-1.441413	2.466278

C	2.853023	-1.641351	0.930791
H	3.039349	-2.699408	1.116995
H	3.573630	-1.315559	0.189251
H	3.056187	-1.121363	1.860377
C	0.490697	-0.351737	-2.551203
H	0.433530	-1.424976	-2.490337
H	-0.522538	0.046159	-2.490656
H	0.875779	-0.108023	-3.542383
C	1.435788	1.135579	1.058450
C	0.581799	2.425002	0.930425
H	0.727432	3.002668	1.842608
H	0.855071	3.069472	0.108606
H	-0.482478	2.200673	0.857893
C	2.914607	1.558073	0.966054
H	3.089695	2.296576	1.749624
H	3.609807	0.743294	1.132392
H	3.163868	2.038130	0.026475
C	1.178333	0.706946	2.516805
H	1.452356	1.551391	3.147393
H	0.123370	0.503522	2.692728
H	1.772803	-0.132520	2.853341
Au	-1.226861	0.008947	0.000908
Cl	-3.543456	0.013059	0.004122

37

16Au SCF Done: -1084.08453796 A.U.

N	-1.056278	0.022848	0.217959
C	-1.710081	-1.265466	-0.440912
C	-2.155190	-0.788348	-1.847501
C	-1.605049	0.614301	-2.020237
C	-1.616685	1.215780	-0.614457
H	-3.243147	-0.779555	-1.911654
H	-1.798432	-1.470728	-2.617569
H	-0.579757	0.591288	-2.394916
H	-2.193343	1.220208	-2.709002
C	-0.693830	-2.402444	-0.590215
H	-0.245871	-2.701543	0.354110
H	-1.208604	-3.270704	-1.006821
H	0.106325	-2.132448	-1.279755
C	-2.934622	-1.808112	0.315386
H	-3.382649	-2.579941	-0.311767
H	-2.686226	-2.271934	1.266855
H	-3.697194	-1.052914	0.488641
C	-0.749601	2.479223	-0.608622
H	-1.124400	3.135657	-1.396275
H	-0.785072	3.033693	0.322982
H	0.292486	2.253237	-0.836088
C	-3.050246	1.604809	-0.214190
H	-3.110811	1.979182	0.805350
H	-3.379321	2.408012	-0.874108
H	-3.759342	0.788709	-0.319207
C	-1.313319	0.110057	1.716333
H	-2.391078	0.036925	1.859149

C	-0.648814	-1.028557	2.496614
H	-0.987285	-0.975968	3.532355
H	-0.889779	-2.018104	2.126264
H	0.437409	-0.920778	2.491006
C	-0.852833	1.425541	2.347412
H	-1.445692	2.281827	2.043426
H	-0.952475	1.333254	3.429240
H	0.195991	1.627700	2.125034
Au	1.136708	-0.012735	-0.057287
Cl	3.435522	-0.035862	-0.319114

40

17Au SCF Done: -1123.34304724 A.U.

N	0.972303	0.067270	0.015365
C	1.333465	-0.859751	-1.250787
C	1.767758	-2.183569	-0.602040
C	1.127188	-2.199889	0.766320
C	1.297646	-0.778028	1.312326
H	2.853625	-2.223844	-0.518183
H	1.459921	-3.022092	-1.224373
H	0.064484	-2.442434	0.697562
H	1.580347	-2.920550	1.444350
C	0.033680	-1.047748	-2.036844
H	-0.351289	-0.112038	-2.435261
H	0.228695	-1.711226	-2.878454
H	-0.741880	-1.511969	-1.424498
C	2.447778	-0.348537	-2.150792
H	2.636256	-1.139481	-2.878536
H	2.189231	0.546910	-2.708496
H	3.373856	-0.185606	-1.605451
C	0.273421	-0.550474	2.425611
H	0.334232	-1.408062	3.096692
H	0.457705	0.339564	3.017160
H	-0.746307	-0.515161	2.038350
C	2.716774	-0.541814	1.813899
H	2.843367	0.451690	2.235892
H	2.909016	-1.259947	2.611168
H	3.467125	-0.694076	1.040559
C	1.214581	1.627117	-0.040542
C	0.378567	2.172305	-1.204720
H	0.493860	3.255472	-1.220510
H	0.698737	1.800178	-2.172625
H	-0.683544	1.957659	-1.075371
C	0.666194	2.247622	1.249207
H	1.288298	2.031674	2.112741
H	0.662809	3.328636	1.116282
H	-0.358756	1.940879	1.454603
C	2.680040	2.039105	-0.208803
H	2.738524	3.102253	0.024511
H	3.351757	1.523723	0.468150
H	3.041016	1.916357	-1.222880
Au	-1.006584	0.003175	-0.001988
Cl	-3.335273	-0.072250	-0.022408

28
18Au SCF Done: -966.111201103 A.U.

N	-1.262552	0.428594	0.000068
C	-2.567534	-2.268893	-0.000024
C	-1.914921	-1.678580	-1.245712
C	-1.924670	-0.156105	-1.218797
C	-1.924573	-0.156212	1.218934
C	-1.914915	-1.678701	1.245714
H	-1.414676	0.227490	-2.095939
H	-0.883932	-2.033525	-1.330960
H	-2.436066	-1.997222	-2.151119
H	-3.636573	-2.031248	-0.000016
H	-2.481559	-3.356925	-0.000064
H	-1.414537	0.227239	2.096117
H	-2.958831	0.208030	1.238882
H	-0.883945	-2.033705	1.330948
H	-2.436082	-1.997395	2.151090
H	-2.958935	0.208122	-1.238612
C	-1.465722	1.939505	0.000094
H	-2.552637	2.083827	0.000216
C	-0.892465	2.604111	-1.250249
H	-1.373029	2.275228	-2.168793
H	-1.046205	3.680503	-1.172142
H	0.180858	2.421724	-1.332717
C	-0.892132	2.604231	1.250211
H	-1.046707	3.680532	1.172442
H	-1.371853	2.274837	2.169009
H	0.181366	2.422580	1.331964
Au	0.835616	-0.077123	-0.000022
Cl	3.078770	-0.613158	-0.000006

40
19Au SCF Done: -1123.39515508 A.U.

N	0.955993	0.360578	0.000070
C	2.313561	-2.354901	-0.000262
C	1.599584	-1.820480	1.217109
C	1.556748	-0.283408	1.334420
C	1.556785	-0.283072	-1.334430
C	1.599633	-1.820184	-1.217536
H	0.571737	-2.196239	1.217713
H	2.065012	-2.188874	2.132509
H	3.368612	-2.071569	-0.000186
H	2.286729	-3.446784	-0.000400
H	0.571797	-2.195972	-1.218295
H	2.065128	-2.188315	-2.133010
C	1.151421	1.895546	0.000315
H	2.227733	2.061677	0.000578
C	0.579194	2.611224	1.230844
H	1.165119	2.461615	2.129065
H	0.589537	3.681411	1.030642
H	-0.453486	2.320825	1.426654
C	0.579796	2.611581	-1.230264
H	0.590530	3.681733	-1.029889

H	1.165884	2.461877	-2.128356
H	-0.452951	2.321610	-1.426362
C	2.961226	0.240197	1.677575
H	2.988442	1.318027	1.817474
H	3.259835	-0.210202	2.624970
H	3.714159	-0.026053	0.943544
C	2.961320	0.240481	-1.677470
H	3.714439	-0.027116	-0.944105
H	3.259352	-0.208808	-2.625572
H	2.988977	1.318477	-1.815947
C	0.656942	-0.043528	-2.570799
H	0.772228	0.927909	-3.031314
H	0.935355	-0.783628	-3.321234
H	-0.397968	-0.195764	-2.342568
C	0.656881	-0.044271	2.570842
H	-0.397971	-0.196989	2.342666
H	0.935680	-0.784259	3.321247
H	0.771747	0.927212	3.031358
Au	-1.202762	-0.075583	-0.000005
Cl	-3.471643	-0.529992	-0.000198

31

20Au SCF Done: -1005.43780000 A.U.

N	1.216343	-0.012901	0.000471
C	1.584334	2.956992	-0.000896
C	1.167128	2.185782	-1.249663
C	1.680651	0.751681	-1.209456
C	1.653292	0.747729	1.224034
C	1.157167	2.189626	1.246579
H	1.357451	0.214963	-2.095534
H	0.077918	2.187272	-1.346455
H	1.566939	2.655704	-2.151065
H	2.672531	3.080599	0.002068
H	1.149003	3.957882	-0.002878
H	1.285596	0.222860	2.099166
H	2.745620	0.748326	1.260184
H	0.067308	2.200013	1.335540
H	1.552925	2.660009	2.149567
H	2.772816	0.775497	-1.203089
C	1.786870	-1.459868	0.001485
C	1.189637	-2.248250	-1.171144
H	1.436848	-1.813284	-2.137734
H	1.599345	-3.258557	-1.155970
H	0.104584	-2.323432	-1.091302
C	1.395944	-2.160098	1.309668
H	1.624122	-3.221775	1.217747
H	1.951342	-1.781533	2.166344
H	0.328095	-2.062933	1.511888
C	3.319766	-1.479203	-0.129390
H	3.814341	-0.881793	0.635078
H	3.657562	-2.507968	-0.003253
H	3.657943	-1.145543	-1.109094
Au	-0.961691	-0.077419	-0.002959

Cl -3.268931 -0.141542 0.001292
33
21Au SCF Done: -1006.62223145 A.U.

N	1.172750	0.000014	0.000025
C	1.594227	-1.185064	0.875890
C	0.664476	-2.389897	0.708052
H	0.621401	-2.724297	-0.329412
H	1.044798	-3.219531	1.305780
H	-0.350690	-2.177075	1.042624
C	3.035608	-1.691116	0.720822
H	3.791940	-0.922485	0.822474
H	3.211812	-2.423975	1.509259
H	3.178066	-2.202033	-0.231833
C	1.595486	1.350951	0.587759
C	0.665930	1.809241	1.714619
H	0.622729	1.078910	2.523882
H	1.046380	2.742143	2.133107
H	-0.349251	1.992292	1.363013
C	3.037125	1.469350	1.103088
H	3.213650	2.518671	1.342975
H	3.180270	0.900391	2.022191
H	3.793091	1.172773	0.386367
C	1.593849	-0.166348	-1.464255
C	3.035709	0.219438	-1.825274
H	3.211643	-0.098049	-2.853855
H	3.179651	1.299812	-1.792402
H	3.791507	-0.253269	-1.210076
C	0.664454	0.582235	-2.423559
H	1.044397	0.478718	-3.440978
H	-0.351142	0.187331	-2.406202
H	0.622501	1.648115	-2.194801
H	1.471080	-1.232157	-1.652184
H	1.473879	2.046320	-0.241575
H	1.472312	-0.815016	1.892913
Au	-1.073907	0.000191	0.000508
Cl	-3.388931	-0.000326	-0.000851

27
22Au SCF Done: -927.998398050 A.U.

N	1.231520	0.195846	-0.430037
C	2.080193	-1.011958	-0.010123
C	1.697399	-2.147381	-0.966864
H	1.933930	-1.890558	-2.002281
H	2.255414	-3.047476	-0.709678
H	0.633037	-2.377411	-0.901323
C	3.575213	-0.709668	-0.166299
H	3.926253	0.025912	0.556909
H	4.139036	-1.627613	0.000680
H	3.808900	-0.353679	-1.171482
C	1.734215	1.582909	-0.097826
C	1.635716	1.919711	1.382933
H	1.895552	2.969291	1.526080
H	0.618840	1.765856	1.749199

H	2.316912	1.327947	1.990215
C	0.977828	2.598480	-0.950384
H	1.444242	3.578033	-0.840197
H	1.002656	2.332824	-2.009415
H	-0.065576	2.680532	-0.641039
H	2.781916	1.622218	-0.399129
C	1.775549	-1.439758	1.426790
H	2.015912	-0.665958	2.150759
H	0.724017	-1.703666	1.543112
H	2.374681	-2.319144	1.667000
H	1.258909	0.166375	-1.447631
Au	-0.874969	-0.044577	-0.078311
Cl	-3.142316	-0.299225	0.260334

24

23Au SCF Done: -888.668659383 A.U.

N	-1.341645	0.003207	-0.366903
C	-1.934604	1.365296	-0.107637
C	-1.274187	2.398381	-1.012776
H	-1.301130	2.087590	-2.059321
H	-1.810483	3.344444	-0.929871
H	-0.233748	2.569101	-0.731264
C	-2.056595	-1.151719	0.300669
C	-1.511134	-2.464962	-0.251279
H	-2.003589	-3.302033	0.243587
H	-1.706313	-2.545923	-1.323855
H	-0.437165	-2.558196	-0.089312
C	-3.573546	-1.088326	0.123852
H	-4.017028	-1.991148	0.544314
H	-4.023205	-0.237013	0.632309
H	-3.843418	-1.044312	-0.934313
H	-1.812921	-1.084518	1.360258
C	-1.835721	1.744834	1.362313
H	-2.364104	1.042691	2.006880
H	-0.791990	1.786670	1.680216
H	-2.278887	2.730196	1.509936
H	-1.439203	-0.152459	-1.369956
H	-2.988023	1.310082	-0.389101
Au	0.778918	-0.058259	-0.058382
Cl	3.060171	-0.129187	0.267006

27

24Au SCF Done: -1387.56602110 A.U.

N	1.144471	0.102445	0.062065
C	1.708048	1.272837	-0.765000
C	0.700122	2.412201	-0.855624
H	0.427282	2.766755	0.139518
H	1.158593	3.243380	-1.393671
H	-0.204086	2.123771	-1.390356
C	3.055238	1.839589	-0.306729
H	3.781974	1.088938	-0.021464
H	3.470186	2.409695	-1.139208
H	2.922137	2.521897	0.531527
C	1.721073	-1.301141	-0.310908

C 1.230467 -1.634948 -1.728106
H 1.616837 -0.953257 -2.483136
H 1.591213 -2.633004 -1.976912
H 0.142143 -1.644947 -1.787510
C 3.249598 -1.351444 -0.274612
H 3.556016 -2.385579 -0.431793
H 3.702004 -0.754072 -1.063534
H 3.640158 -1.034599 0.691431
H 1.824462 0.832246 -1.751818
Cl 1.506010 0.455139 1.798138
C 1.162049 -2.344064 0.661793
H 1.565342 -2.223429 1.664576
H 0.073398 -2.303121 0.714805
H 1.444095 -3.333222 0.301781
Au -1.065299 0.021987 0.005594
Cl -3.360361 -0.074771 -0.033756
24
25Au SCF Done: -1348.24544627 A.U.
N 1.221888 0.060839 0.102044
C 1.712674 1.477741 -0.196891
C 1.498022 1.809731 -1.673229
H 0.450481 1.687639 -1.953582
H 1.765299 2.855817 -1.822284
H 2.114372 1.214013 -2.342462
C 1.020713 2.512182 0.682390
H 1.099454 2.276497 1.740788
H 1.500099 3.477363 0.515566
H -0.034509 2.607771 0.421433
C 1.866977 -0.997532 -0.796143
C 3.390018 -0.925947 -0.843569
H 3.745604 -1.711140 -1.510803
H 3.762809 0.021793 -1.226789
H 3.823750 -1.101852 0.140559
H 2.779264 1.482853 0.028767
Cl 1.719231 -0.305823 1.806378
C 1.394181 -2.402080 -0.444069
H 1.826854 -2.747032 0.493997
H 0.307824 -2.458799 -0.371099
H 1.714645 -3.079255 -1.236220
H 1.457982 -0.742465 -1.773599
Au -0.942892 -0.061584 -0.002215
Cl -3.229478 -0.175855 -0.160176
18
26Au SCF Done: -1729.16323893 A.U.
N -1.158870 -0.320352 -0.000002
C -2.023461 0.990240 -0.000021
C -1.627082 1.771627 -1.254175
H -1.914686 1.255071 -2.166945
H -2.150674 2.727135 -1.226875
H -0.556223 1.973501 -1.280265
C -3.515160 0.674458 -0.000009
H -4.057741 1.619472 -0.000019

H	-3.811878	0.117890	-0.886970
H	-3.811867	0.117915	0.886971
Cl	-1.567828	-1.330791	1.426368
C	-1.627074	1.771631	1.254125
H	-1.914664	1.255070	2.166897
H	-0.556215	1.973510	1.280201
H	-2.150673	2.727135	1.226830
Cl	-1.567818	-1.330876	-1.426317
Au	1.006696	-0.030247	-0.000004
Cl	3.268844	0.286226	0.000010
30			
27Au SCF Done: -1426.88839268 A.U.			
N	-1.073104	-0.003293	-0.178740
C	-1.657418	1.361118	0.371162
C	-0.826501	2.498588	-0.243770
H	-0.877014	2.508544	-1.329375
H	-1.225126	3.445377	0.119896
H	0.219860	2.440967	0.057897
C	-3.132273	1.603823	0.023448
H	-3.808740	0.936757	0.544829
H	-3.375703	2.619481	0.336130
H	-3.320899	1.535997	-1.045222
C	-1.603461	-1.398105	0.348222
C	-3.127460	-1.513186	0.314857
H	-3.388819	-2.561029	0.464755
H	-3.601625	-0.944877	1.110860
H	-3.537168	-1.203337	-0.645020
Cl	-1.392366	0.019902	-1.968134
C	-1.005542	-2.504417	-0.538820
H	-1.471367	-2.548105	-1.519111
H	0.070946	-2.384326	-0.666567
H	-1.178257	-3.459513	-0.044235
C	-1.479821	1.417217	1.891379
H	-2.213662	0.813242	2.417263
H	-0.476942	1.124172	2.199107
H	-1.632648	2.451579	2.199149
C	-1.095184	-1.642948	1.777745
H	-1.556487	-1.001848	2.517712
H	-1.349735	-2.670544	2.037169
H	-0.011998	-1.541889	1.846995
Au	1.130329	0.012006	-0.024331
Cl	3.422785	0.014298	0.139060
52			
28Au SCF Done: -1426.88839268 A.U.			
N	-0.104809	1.083084	-0.015318
C	-0.531042	2.009646	1.208112
C	-1.614949	2.947223	0.616516
C	-1.889505	2.464506	-0.795135
C	-0.562138	1.877529	-1.280772
H	-1.248414	3.973478	0.601191
H	-2.227475	3.261554	-1.457217
C	-1.129251	1.187787	2.354841

H	-0.456050	0.414971	2.718920
H	-1.346240	1.861903	3.186088
H	-2.066893	0.715590	2.061318
C	0.603815	2.874236	1.788092
H	0.144746	3.561591	2.499803
H	1.352379	2.303400	2.331809
H	1.107709	3.480242	1.039521
C	-0.830078	1.017752	-2.521661
H	-1.296434	1.659452	-3.271747
H	0.061179	0.588590	-2.967323
H	-1.525422	0.206618	-2.304970
C	0.402506	3.013876	-1.665093
H	1.378323	2.650552	-1.978990
H	-0.029539	3.551150	-2.509722
H	0.549394	3.734759	-0.865835
C	1.394117	0.777275	0.020324
H	1.862245	1.756093	0.096125
C	2.028042	0.123879	-1.235064
C	1.891626	-0.037529	1.248676
H	1.688729	0.620382	-2.135754
C	1.761570	-1.383870	-1.347929
C	3.552227	0.352124	-1.135191
H	1.426561	0.324615	2.159575
C	1.647441	-1.551144	1.138407
C	3.414220	0.193388	1.353982
H	2.236719	-1.758864	-2.259138
H	0.692061	-1.585253	-1.445863
C	2.326226	-2.108922	-0.117523
H	3.770079	1.423688	-1.092176
H	4.028245	-0.034970	-2.040374
C	4.114078	-0.358387	0.102483
H	0.579011	-1.778485	1.120940
H	2.050335	-2.033452	2.033874
H	3.630402	1.259839	1.469374
H	3.790977	-0.308695	2.249220
H	2.125769	-3.180126	-0.198847
C	3.842012	-1.867200	-0.020560
H	5.190404	-0.180243	0.174414
H	4.249085	-2.394585	0.847395
H	4.345401	-2.265724	-0.906683
Au	-1.351038	-0.741699	0.021278
Cl	-2.703363	-2.625623	0.033053
H	-2.511074	2.940538	1.235472
H	-2.654927	1.686615	-0.801082

38

29Au SCF Done: -1122.16499704 A.U.

N	1.050245	-0.025237	0.000003
C	1.450099	0.847997	1.247289
C	1.378732	2.229459	0.658551
C	1.378709	2.229618	-0.658021
C	1.450090	0.848299	-1.247087
H	1.364083	3.098245	1.301625

H	1.364044	3.098558	-1.300884
C	0.482567	0.750832	2.437154
H	0.459913	-0.234156	2.891120
H	0.820146	1.458222	3.197213
H	-0.533446	1.030055	2.157930
C	2.874688	0.641239	1.797776
H	3.087750	1.470395	2.474133
H	2.963017	-0.276096	2.376685
H	3.632225	0.654278	1.020616
C	0.482560	0.751398	-2.436979
H	0.820065	1.459056	-3.196819
H	0.460000	-0.233450	-2.891252
H	-0.533479	1.030438	-2.157664
C	2.874686	0.641727	-1.797634
H	2.963136	-0.275590	-2.376553
H	3.087607	1.470919	-2.473992
H	3.632248	0.654894	-1.020497
C	1.454903	-1.532583	-0.000185
C	0.858584	-2.211560	1.243499
H	0.994677	-3.288620	1.147449
H	1.360491	-1.904412	2.157797
H	-0.209485	-2.018170	1.346055
C	0.858812	-2.211143	-1.244205
H	1.361011	-1.903836	-2.158289
H	0.994711	-3.288246	-1.148396
H	-0.209198	-2.017563	-1.346993
C	2.962283	-1.881681	-0.000076
H	3.028390	-2.970589	-0.000701
H	3.489284	-1.536457	-0.880248
H	3.488856	-1.537508	0.880770
Au	-1.217957	-0.004500	0.000007
Cl	-3.532908	-0.001755	-0.000012

56

31Au SCF Done: -1508.25829760 A.U.

N	0.209625	-0.730711	0.000094
C	1.043407	-1.202583	1.252140
C	2.442932	-1.206954	0.690672
C	2.442904	-1.207049	-0.690503
C	1.043353	-1.202772	-1.251906
C	0.944891	-0.237871	2.442814
H	-0.078479	-0.044180	2.747210
H	1.463227	-0.681353	3.294245
H	1.425072	0.714328	2.221876
C	0.708290	-2.628318	1.743522
H	1.453694	-2.896820	2.491982
H	-0.268864	-2.694964	2.216136
H	0.767336	-3.371540	0.954510
C	0.944743	-0.238250	-2.442724
H	1.463052	-0.681844	-3.294114
H	-0.078654	-0.044651	-2.747095
H	1.424895	0.714004	-2.221959
C	0.708211	-2.628585	-1.743041

H	-0.268976	-2.695310	-2.215577
H	1.453565	-2.897202	-2.491510
H	0.767315	-3.371680	-0.953914
C	-1.211562	-1.295304	0.000118
H	-1.069542	-2.374685	0.000169
C	-2.084162	-0.981364	-1.246072
C	-2.084227	-0.981247	1.246237
H	-1.518060	-1.132839	-2.157984
C	-2.671255	0.438017	-1.247130
C	-3.249808	-1.993156	-1.247581
H	-1.518179	-1.132642	2.158193
C	-2.671328	0.438131	1.247137
C	-3.249872	-1.993039	1.247776
H	-3.269969	0.568672	-2.153193
H	-1.879119	1.189797	-1.285349
C	-3.539661	0.649769	-0.000031
H	-2.862566	-3.016057	-1.281908
H	-3.845965	-1.845816	-2.152200
C	-4.121072	-1.794443	0.000065
H	-1.879198	1.189918	1.285338
H	-3.270096	0.568861	2.153153
H	-2.862632	-3.015936	1.282218
H	-3.846078	-1.845616	2.152350
H	-3.940312	1.666410	-0.000089
C	-4.693648	-0.366845	-0.000017
H	-4.937331	-2.521696	0.000077
H	-5.325485	-0.217017	0.880639
H	-5.325435	-0.217099	-0.880724
C	3.637062	-1.239088	-1.399926
C	3.637121	-1.238895	1.400049
C	4.837369	-1.270283	-0.696986
C	4.837398	-1.270187	0.697063
H	3.641762	-1.235771	-2.482291
H	5.775844	-1.289458	-1.236074
H	5.775896	-1.289288	1.236114
H	3.641867	-1.235425	2.482413
Au	0.303249	1.475531	-0.000099
Cl	0.450638	3.787461	-0.000323

42

33Au SCF Done: -1124.58482650 A.U.

N	-1.023883	-0.197283	0.030588
C	-1.440236	-0.359134	-1.501497
C	-1.066152	-1.772287	-2.003918
H	-1.680900	-2.570417	-1.600033
H	-1.206279	-1.781885	-3.084046
H	-0.016646	-2.000649	-1.813917
C	-2.936006	-0.125509	-1.789165
H	-3.228168	0.913562	-1.667517
H	-3.117347	-0.386860	-2.832470
H	-3.596397	-0.735730	-1.184498
C	-1.568712	1.084962	0.792587
C	-1.519450	2.393366	-0.049356

H	-2.058743	2.272599	-0.981843
H	-2.122782	3.101578	0.522100
C	-0.735836	1.381661	2.057679
H	0.335606	1.383007	1.863479
H	-1.007837	2.379077	2.405496
H	-0.935674	0.697807	2.871673
C	-3.022711	0.908704	1.271375
H	-3.101439	0.148145	2.041598
H	-3.346478	1.845375	1.725465
H	-3.719625	0.672919	0.474768
C	-0.645241	0.584478	-2.428359
H	-0.827899	1.636040	-2.263440
H	0.427759	0.405094	-2.357248
H	-0.945028	0.366907	-3.453924
C	-1.271946	-1.549006	0.776314
C	-2.677562	-2.177458	0.776480
H	-2.560243	-3.187977	1.173415
H	-3.388668	-1.663387	1.414709
H	-3.116529	-2.282094	-0.208271
C	-0.728048	-1.637457	2.206294
H	-0.619840	-2.698236	2.438071
H	0.252483	-1.179667	2.318972
H	-1.407003	-1.216563	2.945120
H	-0.649847	-2.228177	0.207389
C	-0.166288	3.065970	-0.296275
H	-0.305638	3.921299	-0.960543
H	0.269855	3.439131	0.629619
H	0.567379	2.407593	-0.757523
Au	1.204530	-0.133767	0.014000
Cl	3.516729	-0.147038	-0.003511

45

34Au SCF Done: -1163.90977543 A.U.

N	1.038761	0.038851	0.154358
C	1.488835	0.966226	-1.075165
C	1.295517	2.491566	-0.780467
H	1.922213	2.809038	0.049284
H	1.734670	2.975640	-1.654825
C	2.971430	0.813029	-1.480389
H	3.229355	-0.193407	-1.789009
H	3.145372	1.458874	-2.341417
H	3.667354	1.120119	-0.706835
C	1.544103	-1.466265	0.136808
C	1.457517	-2.152760	-1.259652
H	2.007341	-1.590185	-2.004767
H	2.030337	-3.074227	-1.137420
C	0.701275	-2.351650	1.079182
H	-0.369082	-2.247374	0.910127
H	0.965910	-3.390977	0.878949
H	0.900791	-2.169916	2.126773
C	2.999473	-1.599749	0.630883
H	3.074524	-1.400763	1.695195
H	3.317904	-2.631745	0.483432

H 3.704190 -0.961282 0.111528
C 0.658107 0.677590 -2.340821
H 0.907564 -0.260990 -2.818448
H -0.412856 0.685355 -2.146596
H 0.863202 1.468263 -3.063454
C 1.307737 0.791496 1.490543
C 2.742612 1.217141 1.856776
H 2.649475 2.027929 2.582587
H 3.315269 0.426712 2.332816
H 3.322512 1.602552 1.028272
C 0.678852 0.188959 2.752144
H 0.609158 0.994070 3.485856
H -0.328065 -0.191136 2.588360
H 1.287882 -0.595206 3.196429
H 0.760988 1.709660 1.330568
C 0.089554 -2.555980 -1.814883
H 0.223679 -2.996544 -2.805076
H -0.396137 -3.302829 -1.188059
H -0.599713 -1.720700 -1.919176
C -0.109042 3.074549 -0.612368
H -0.033547 4.162972 -0.566272
H -0.760547 2.826126 -1.449181
H -0.610218 2.748191 0.299161
Au -1.197916 -0.010380 0.104751
Cl -3.513018 -0.017946 0.096460
48
35Au SCF Done: -1203.25568496 A.U.
N 0.287594 1.150336 0.225267
C -1.224047 1.166845 -0.349091
C -2.078872 0.240975 0.584022
H -1.432948 -0.567801 0.933268
H -2.345717 0.831292 1.460322
C -1.818405 2.579983 -0.321704
H -1.316120 3.254317 -1.009932
H -2.859388 2.523892 -0.631670
H -1.804968 3.018592 0.674634
C 1.337898 2.146601 -0.417028
C 1.203579 2.296461 -1.936883
H 0.326186 2.858442 -2.243408
H 2.074189 2.854697 -2.282176
H 1.218272 1.331071 -2.437624
C 2.765312 1.593042 -0.215901
H 2.943168 0.732974 -0.861696
H 3.473122 2.372386 -0.504032
H 2.993862 1.304491 0.800628
C 1.270822 3.555379 0.205338
H 1.597278 3.577681 1.242052
H 1.949517 4.199577 -0.354013
H 0.282046 3.999259 0.146064
C -1.275992 0.639659 -1.785266
H -0.837220 1.312851 -2.505544
H -0.798357 -0.334411 -1.881694

H	-2.317318	0.531336	-2.067995
C	0.201528	1.405699	1.722007
C	1.431693	1.212997	2.603071
H	1.082471	1.249405	3.637226
H	1.894167	0.237574	2.447181
H	2.189668	1.983437	2.492287
H	-0.530803	0.713597	2.104816
H	-0.198942	2.405639	1.882647
C	-3.400817	-0.480548	0.164084
C	-3.960461	-1.046467	1.488710
H	-4.187608	-0.246966	2.197604
H	-4.880653	-1.604889	1.305680
H	-3.244982	-1.724252	1.960295
C	-3.165811	-1.684470	-0.767761
H	-2.927971	-1.399261	-1.789229
H	-2.349918	-2.308759	-0.396851
H	-4.067800	-2.299491	-0.806379
C	-4.471123	0.449630	-0.424581
H	-4.677598	1.286414	0.246432
H	-4.193196	0.855717	-1.397130
H	-5.403756	-0.102533	-0.561836
Au	0.975332	-0.917375	-0.028021
Cl	1.670299	-3.110631	-0.262190

38

37Au SCF Done: -1122.16499704 A.U.

N	1.050245	-0.025237	0.000003
C	1.450099	0.847997	1.247289
C	1.378732	2.229459	0.658551
C	1.378709	2.229618	-0.658021
C	1.450090	0.848299	-1.247087
H	1.364083	3.098245	1.301625
H	1.364044	3.098558	-1.300884
C	0.482567	0.750832	2.437154
H	0.459913	-0.234156	2.891120
H	0.820146	1.458222	3.197213
H	-0.533446	1.030055	2.157930
C	2.874688	0.641239	1.797776
H	3.087750	1.470395	2.474133
H	2.963017	-0.276096	2.376685
H	3.632225	0.654278	1.020616
C	0.482560	0.751398	-2.436979
H	0.820065	1.459056	-3.196819
H	0.460000	-0.233450	-2.891252
H	-0.533479	1.030438	-2.157664
C	2.874686	0.641727	-1.797634
H	2.963136	-0.275590	-2.376553
H	3.087607	1.470919	-2.473992
H	3.632248	0.654894	-1.020497
C	1.454903	-1.532583	-0.000185
C	0.858584	-2.211560	1.243499
H	0.994677	-3.288620	1.147449
H	1.360491	-1.904412	2.157797

H	-0.209485	-2.018170	1.346055
C	0.858812	-2.211143	-1.244205
H	1.361011	-1.903836	-2.158289
H	0.994711	-3.288246	-1.148396
H	-0.209198	-2.017563	-1.346993
C	2.962283	-1.881681	-0.000076
H	3.028390	-2.970589	-0.000701
H	3.489284	-1.536457	-0.880248
H	3.488856	-1.537508	0.880770
Au	-1.217957	-0.004500	0.000007
Cl	-3.532908	-0.001755	-0.000012

63

38Au SCF Done: -1549.99235965 A.U.

N	-0.153291	-0.327583	0.611591
C	-0.255568	-0.206795	2.139744
C	-1.152581	0.917217	2.652022
H	-2.210039	0.685668	2.587219
H	-0.917481	1.070448	3.706779
H	-0.969438	1.857516	2.132167
H	0.752551	0.100262	2.403188
C	-0.544628	-1.498105	2.922933
H	-0.011568	-2.367446	2.546871
H	-0.213909	-1.336998	3.950628
H	-1.601759	-1.741132	2.959744
C	0.907180	-1.419724	0.277794
H	0.357274	-2.348498	0.440386
C	2.168135	-1.500097	1.180616
C	1.410976	-1.476073	-1.196620
H	1.910129	-1.416493	2.231278
C	3.229756	-0.442234	0.840146
C	2.775559	-2.904734	0.973514
H	0.586525	-1.348106	-1.896285
C	2.499451	-0.446158	-1.539100
C	2.008942	-2.885028	-1.405119
H	4.070845	-0.555829	1.530079
H	2.835901	0.566941	0.986977
C	3.703860	-0.616526	-0.609402
H	2.045031	-3.672789	1.245033
H	3.629234	-3.023410	1.646450
C	3.222737	-3.078892	-0.482732
H	2.112213	0.572711	-1.471439
H	2.797603	-0.593891	-2.581199
H	1.254818	-3.652301	-1.206154
H	2.309052	-2.993313	-2.450731
H	4.459076	0.138342	-0.842007
C	4.294567	-2.023183	-0.803237
H	3.635232	-4.081293	-0.625217
H	4.643082	-2.141420	-1.833575
H	5.161544	-2.162948	-0.150399
C	-1.402987	-0.728567	-0.213412
H	-0.917655	-1.175222	-1.068249
C	-2.269209	0.383794	-0.855271

C	-2.355039	-1.837483	0.307241
H	-1.625135	1.213458	-1.155488
C	-3.412292	0.900857	0.025786
C	-2.887612	-0.247829	-2.124085
H	-1.793082	-2.604780	0.838740
C	-3.512612	-1.321054	1.181451
C	-2.980251	-2.476014	-0.957333
H	-3.981442	1.643961	-0.540165
H	-3.029953	1.413340	0.905405
C	-4.331655	-0.265784	0.423134
H	-2.097229	-0.581975	-2.802414
H	-3.468308	0.509185	-2.657769
C	-3.795160	-1.426070	-1.732781
H	-3.157420	-0.905087	2.119769
H	-4.152352	-2.168273	1.446042
H	-2.195489	-2.895188	-1.593669
H	-3.628861	-3.304570	-0.659687
H	-5.134177	0.102020	1.067926
C	-4.931842	-0.903144	-0.839803
H	-4.211802	-1.883059	-2.634430
H	-5.601469	-1.723339	-0.563686
H	-5.529202	-0.167240	-1.386225
Au	0.508667	1.667142	-0.019352
Cl	1.202213	3.798199	-0.610070

70

39Au SCF Done: -1666.77903213 A.U.

N	0.128360	0.325292	0.254526
C	-0.956217	1.415227	0.000487
H	-0.398473	2.345893	0.115967
C	-2.139907	1.491704	1.003896
C	-1.580471	1.469240	-1.426958
H	-1.796298	1.408613	2.029663
C	-3.220326	0.428277	0.752852
C	-2.769498	2.892610	0.847530
H	-0.817223	1.350634	-2.194157
C	-2.686010	0.431890	-1.678698
C	-2.201777	2.874735	-1.584752
H	-4.002923	0.538971	1.508908
H	-2.809536	-0.578069	0.869412
C	-3.811624	0.596870	-0.653538
H	-2.022857	3.664783	1.056480
H	-3.564616	3.007582	1.589345
C	-3.336994	3.062609	-0.566674
H	-2.287494	-0.584207	-1.641238
H	-3.069848	0.575818	-2.692927
H	-1.437789	3.645895	-1.448146
H	-2.586523	2.980934	-2.602550
H	-4.578923	-0.162644	-0.822856
C	-4.424704	1.999933	-0.798853
H	-3.765994	4.062326	-0.675330
H	-4.856496	2.115860	-1.797431
H	-5.236633	2.134376	-0.077653

C	1.297655	0.694443	-0.689926
H	0.732969	1.084669	-1.522669
C	2.129210	-0.434948	-1.351588
C	2.267727	1.844796	-0.311609
H	1.481832	-1.293107	-1.545762
C	3.369660	-0.880919	-0.567890
C	2.604628	0.145990	-2.704369
H	1.740150	2.623795	0.238457
C	3.515813	1.393271	0.469908
C	2.752651	2.435173	-1.658288
H	3.900590	-1.634632	-1.156561
H	3.095030	-1.361605	0.367685
C	4.288987	0.325142	-0.317485
H	1.743340	0.424955	-3.318155
H	3.153762	-0.622445	-3.254909
C	3.512153	1.364203	-2.461364
H	3.260594	1.008782	1.453717
H	4.153772	2.266074	0.638176
H	1.900578	2.810637	-2.232133
H	3.409286	3.287826	-1.464193
H	5.160668	0.008683	0.261492
C	4.745252	0.913912	-1.661614
H	3.825308	1.784363	-3.420958
H	5.414298	1.762953	-1.491657
H	5.307824	0.166307	-2.228690
C	0.371049	0.233983	1.764880
C	1.327228	-0.871571	2.220099
C	0.709699	1.537489	2.514276
H	-0.605020	-0.082899	2.128466
C	1.160996	-1.111155	3.725749
H	2.360482	-0.597516	2.023606
H	1.128122	-1.795949	1.674548
C	0.527336	1.314310	4.022027
H	1.739374	1.834039	2.331918
H	0.084319	2.368079	2.193539
C	1.405209	0.168385	4.528154
H	1.848278	-1.897929	4.044672
H	0.148212	-1.479626	3.921607
H	0.760889	2.238113	4.556357
H	-0.524545	1.088485	4.230962
H	1.222901	-0.008949	5.590882
H	2.458122	0.456241	4.431968
Au	-0.592924	-1.679841	-0.268860
Cl	-1.345492	-3.819373	-0.747596

42

41Au SCF Done: -1659.25364012 A.U.

N	-0.028593	1.058680	0.193898
C	-0.474419	2.364712	-0.546425
C	-1.868986	2.783480	-0.073633
H	-1.874398	3.086934	0.970326
H	-2.190843	3.634712	-0.673424
H	-2.596644	1.982575	-0.209270

C	0.505537	3.516754	-0.294166
H	1.504739	3.323480	-0.681006
H	0.126810	4.402989	-0.802866
H	0.581982	3.747542	0.767002
C	1.400374	0.645949	-0.260131
H	1.653980	1.422590	-0.970685
C	1.446382	-0.686247	-1.036898
C	2.540202	0.655625	0.779477
H	0.601242	-0.735656	-1.725903
C	1.453413	-1.915446	-0.112871
C	2.759202	-0.667212	-1.849641
H	2.486441	1.564496	1.377375
C	2.542896	-0.588259	1.685544
C	3.854673	0.674758	-0.033934
H	1.485084	-2.818352	-0.728213
H	0.530833	-1.970241	0.470981
C	2.660483	-1.858990	0.832090
H	2.765733	0.182199	-2.539227
H	2.814324	-1.573530	-2.457952
C	3.963007	-0.592513	-0.896947
H	1.638640	-0.634512	2.291673
H	3.387393	-0.514636	2.376626
H	3.897401	1.569187	-0.662503
H	4.697957	0.730238	0.659319
H	2.653426	-2.736320	1.483658
C	3.962688	-1.827909	0.017245
H	4.887804	-0.559929	-1.478658
H	4.825460	-1.797221	0.689404
H	4.052017	-2.738347	-0.582753
Cl	-0.068462	1.410594	1.973362
C	-0.547202	2.030340	-2.043960
H	0.415845	1.759783	-2.471882
H	-1.252136	1.221230	-2.235434
H	-0.900477	2.918296	-2.568037
Au	-1.583091	-0.489734	0.007947
Cl	-3.232585	-2.081155	-0.166193

51

42Au SCF Done: -1317.63369593 A.U.

N	-0.051414	1.155751	0.135989
C	-0.441138	2.122044	-1.068412
C	-1.696972	2.947937	-0.734550
H	-1.500933	3.783182	-0.063606
H	-2.069019	3.371193	-1.667893
H	-2.491687	2.326082	-0.319914
C	0.676099	3.116707	-1.432499
H	1.570948	2.643426	-1.828030
H	0.291976	3.776840	-2.210669
H	0.959014	3.747038	-0.594187
C	1.431034	0.725969	0.057086
H	1.972881	1.664962	0.150205
C	1.940475	0.075439	-1.258022
C	1.940724	-0.175027	1.217549

H 1.604121 0.635733 -2.121413
C 1.533110 -1.398352 -1.408259
C 3.482490 0.160136 -1.246016
H 1.592883 0.198154 2.172115
C 1.538235 -1.650176 1.073294
C 3.481942 -0.088733 1.228298
H 1.922520 -1.776916 -2.357796
H 0.446392 -1.504258 -1.449834
C 2.092787 -2.219831 -0.238635
H 3.801568 1.204477 -1.173153
H 3.861802 -0.224269 -2.196858
C 4.051010 -0.653653 -0.078444
H 0.451987 -1.765401 1.103189
H 1.935009 -2.207577 1.926873
H 3.801460 0.949050 1.364367
H 3.860504 -0.654953 2.083747
H 1.786720 -3.263522 -0.344566
C 3.627822 -2.124585 -0.229294
H 5.141838 -0.579791 -0.070881
H 4.034821 -2.717825 0.595119
H 4.035306 -2.537505 -1.157081
C -0.829188 1.307082 -2.322048
H -0.160802 0.489132 -2.555454
H -1.832768 0.894392 -2.219092
H -0.838807 1.981423 -3.180351
C -0.309758 1.857572 1.499136
H -1.153527 2.499930 1.304009
C 0.809756 2.773006 2.016183
H 1.665967 2.229766 2.411556
H 1.168210 3.476054 1.270406
H 0.394887 3.361864 2.835388
C -0.804546 0.930958 2.619111
H -0.146726 0.095710 2.838343
H -0.900173 1.526454 3.529665
H -1.790819 0.528393 2.388811
Au -1.477541 -0.513191 0.025388
Cl -3.027838 -2.231670 -0.093378
6
NH3Au SCF Done: -652.692648287 A.U.
N 2.263090 -0.000204 0.000400
H 2.631105 -0.097682 0.942730
H 2.632069 0.864545 -0.385621
H 2.632842 -0.767124 -0.554638
Au 0.159672 0.000044 -0.000125
Cl -2.138336 -0.000106 0.000273
15
NMe3Au SCF Done: -770.656127494 A.U.
N -1.763923 -0.000062 0.000166
C -2.267880 -1.355961 0.345543
H -1.907362 -2.073963 -0.386894
H -3.362615 -1.355499 0.346611
C -2.269269 0.379009 -1.346001

H -1.908155 1.372012 -1.601872
H -3.363958 0.378629 -1.344626
C -2.267791 0.976724 1.001992
H -3.362523 0.975971 1.002530
H -1.905429 0.702144 1.989547
H -1.907018 1.971263 0.751564
H -1.905319 -1.636869 1.331228
H -1.908128 -0.334490 -2.082516
Au 0.371065 0.000047 -0.000326
Cl 2.670202 -0.000063 0.000580
24
NEt3Au SCF Done: -888.652710406 A.U.
N -1.477238 0.000108 -0.000229
C -1.992101 -0.981990 -1.017138
H -1.573831 -0.690257 -1.976087
H -3.079304 -0.855588 -1.075457
C -1.651158 -2.439803 -0.750586
H -2.180238 -2.847265 0.109462
H -1.943349 -3.024158 -1.624139
H -0.579116 -2.576151 -0.598918
C -1.992279 1.371572 -0.343287
H -1.577249 2.056271 0.390589
H -3.079991 1.357465 -0.208586
C -1.646771 1.870625 -1.737703
H -2.173309 1.330166 -2.522731
H -1.938378 2.919455 -1.807226
H -0.574079 1.807245 -1.928094
C -1.993141 -0.388731 1.358713
H -3.080632 -0.500145 1.278432
H -1.576517 -1.365801 1.585675
C -1.650635 0.570529 2.488036
H -2.178386 1.519827 2.411041
H -1.943371 0.106480 3.430858
H -0.578314 0.768627 2.530168
Au 0.674726 -0.000082 0.000100
Cl 2.979407 -0.000803 0.000618
13
PyAu SCF Done: -844.489995929 A.U.
N 1.300910 -0.000281 -0.000548
C 1.985768 1.158170 -0.000340
C 3.368784 1.194567 0.000321
C 4.076987 0.000359 0.000647
C 3.369334 -1.194138 0.000260
C 1.986273 -1.158344 -0.000287
H 5.158683 0.000604 0.001174
H 1.399691 2.064894 -0.000772
H 3.873263 2.150193 0.000588
H 3.874211 -2.149553 0.000364
H 1.400586 -2.065328 -0.000506
Au -0.776200 -0.000079 -0.000138
Cl -3.071521 0.000216 0.000604
42

1Pd SCF Done: -960.392467857 A.U.

N	1.861381	-0.037360	0.133730
C	2.429744	0.112512	1.514728
C	1.420364	-0.034179	2.645063
H	2.885848	1.095682	1.600221
H	3.246598	-0.601768	1.647385
H	1.929398	0.087958	3.605375
H	0.642143	0.727156	2.566979
H	0.931860	-1.007397	2.637400
C	2.279276	-1.364188	-0.444225
C	1.851742	-1.533634	-1.902252
H	3.374791	-1.421505	-0.401722
H	2.067151	-2.554623	-2.220045
H	0.776745	-1.360524	-2.004541
H	2.377464	-0.863899	-2.581225
C	2.287725	1.115390	-0.744129
C	1.730838	2.444816	-0.234957
H	1.807555	0.933682	-1.703334
H	1.891629	3.213565	-0.992855
H	0.656910	2.360747	-0.048179
H	2.216502	2.782022	0.682141
C	3.799884	1.211527	-0.980177
H	4.005937	2.047853	-1.650215
H	4.345222	1.391036	-0.051977
H	4.205160	0.312165	-1.443702
C	1.727798	-2.525378	0.384767
H	2.078619	-2.510138	1.415216
H	0.634956	-2.499415	0.392748
H	2.052187	-3.468704	-0.057491
Pd	-0.500600	0.028358	0.062992
P	-2.686493	0.075992	-0.112730
C	-3.662502	0.265185	1.439322
H	-3.382767	1.196829	1.931603
H	-4.735955	0.274764	1.232287
H	-3.433649	-0.559914	2.114119
C	-3.518496	-1.396615	-0.844425
H	-4.602928	-1.261928	-0.878931
H	-3.143801	-1.559109	-1.855202
H	-3.284069	-2.278100	-0.247325
C	-3.433689	1.415823	-1.134120
H	-4.524435	1.342435	-1.146079
H	-3.142811	2.384927	-0.728225
H	-3.057052	1.345828	-2.154803

31

2Pd SCF Done: -841.205864999 A.U.

N	-1.776050	0.000027	-0.374620
C	-4.608873	-0.000022	0.454777
C	-3.933426	1.256581	-0.099395
C	-2.426259	1.226723	0.154281
C	-2.426234	-1.226693	0.154306
C	-3.933398	-1.256604	-0.099387
H	-1.933024	2.088141	-0.295580

H	-4.113454	1.324774	-1.178205
H	-4.357181	2.157956	0.350675
H	-4.528413	-0.000019	1.547603
H	-5.674794	-0.000046	0.216022
H	-1.933008	-2.088102	-0.295575
H	-2.223960	-1.260443	1.227235
H	-4.113412	-1.324803	-1.178200
H	-4.357129	-2.157989	0.350685
H	-2.223966	1.260431	1.227205
H	-1.902115	0.000047	-1.384878
Pd	0.426454	0.000000	-0.145905
P	2.616383	-0.000013	0.069199
C	3.296223	-0.001085	1.781099
H	4.389282	-0.000945	1.771281
H	2.941006	0.882128	2.312367
H	2.941218	-0.885063	2.311229
C	3.543412	-1.415115	-0.659583
H	3.349941	-1.461628	-1.731443
H	4.618394	-1.310601	-0.491332
H	3.197404	-2.345501	-0.208890
C	3.543208	1.416208	-0.657679
H	3.196943	2.345936	-0.205825
H	4.618184	1.311691	-0.489385
H	3.349890	1.464068	-1.729504

43

3Pd SCF Done: -998.538018724 A.U.

N	1.435629	-0.000247	-0.422661
C	4.285742	0.000697	0.266965
C	3.615490	-1.246847	-0.299695
C	2.099107	-1.299786	-0.029200
C	2.098420	1.299685	-0.029257
C	3.614729	1.247619	-0.300200
H	3.774773	-1.265832	-1.383652
H	4.075116	-2.153576	0.101144
H	4.237458	0.000904	1.358713
H	5.347182	0.000944	0.008461
H	3.773740	1.266181	-1.384206
H	4.073931	2.154786	0.100131
H	1.455833	-0.000262	-1.440622
C	1.813919	1.646997	1.436940
H	2.130794	2.673282	1.631008
H	0.744634	1.566602	1.639954
H	2.342375	1.003976	2.136024
C	1.474377	2.387647	-0.910150
H	1.615652	2.165278	-1.970960
H	0.402582	2.472523	-0.718794
H	1.946123	3.349594	-0.703190
C	1.476221	-2.388135	-0.910440
H	0.404475	-2.473767	-0.719303
H	1.617621	-2.165484	-1.971180
H	1.948736	-3.349716	-0.703521
C	1.814131	-1.647315	1.436810

H	2.341429	-1.003666	2.136180
H	0.744656	-1.568179	1.639231
H	2.131934	-2.673258	1.631168
Pd	-0.825427	0.000273	-0.153283
P	-3.013682	-0.000056	0.055107
C	-3.831265	1.599387	0.465408
H	-3.440861	1.973487	1.412048
H	-3.605392	2.331094	-0.310538
H	-4.915034	1.479902	0.544156
C	-4.010192	-0.514310	-1.407126
H	-3.736986	-1.530489	-1.692244
H	-5.080697	-0.476376	-1.188740
H	-3.790580	0.146842	-2.245660
C	-3.744908	-1.086504	1.351256
H	-3.349221	-0.802966	2.326715
H	-4.834931	-1.004757	1.366390
H	-3.464482	-2.121838	1.156466

34

4Pd SCF Done: -880.531143935 A.U.

N	1.892545	0.852752	-0.000016
C	3.273312	-1.752384	-0.000374
C	2.612353	-1.181272	1.254789
C	2.578826	0.341557	1.212720
C	2.578164	0.341375	-1.213025
C	2.611178	-1.181433	-1.254989
H	2.057382	0.740387	2.083255
H	1.587157	-1.554451	1.336071
H	3.147803	-1.496197	2.153707
H	4.337152	-1.490057	-0.000936
H	3.215307	-2.843072	-0.000256
H	2.056495	0.740397	-2.083316
H	3.607880	0.732960	-1.232176
H	1.585856	-1.554482	-1.335393
H	3.145698	-1.496612	-2.154369
H	3.608390	0.733544	1.231485
C	1.939373	2.326643	-0.000202
H	1.432055	2.709676	-0.884155
H	1.433691	2.709822	0.884595
H	2.977236	2.689765	-0.001232
Pd	-0.307073	0.282358	0.000014
P	-2.452584	-0.183649	-0.000238
C	-3.605606	1.102313	-0.640968
H	-4.642947	0.760908	-0.592325
H	-3.498097	2.012258	-0.050313
H	-3.351307	1.333871	-1.675464
C	-3.004813	-1.642436	-0.980494
H	-2.502471	-2.537315	-0.612671
H	-4.086758	-1.779823	-0.906699
H	-2.730398	-1.501547	-2.026108
C	-3.240036	-0.557362	1.622744
H	-3.120779	0.297214	2.289006
H	-4.304387	-0.775902	1.502857

H	-2.745195	-1.416362	2.076281

46

5Pd SCF Done: -1037.8444668 A.U.

N	1.535558	-0.000583	-0.607395
C	2.498847	0.002891	2.154456
C	1.910032	1.248675	1.507102
C	2.085491	1.296228	-0.020603
C	2.090862	-1.294196	-0.018832
C	1.913668	-1.245376	1.508549
H	0.837128	1.286837	1.723364
H	2.357308	2.151737	1.929760
H	3.588592	0.004361	2.070080
H	2.277911	0.003258	3.224673
H	0.840674	-1.286165	1.723955
H	2.362949	-2.146655	1.932870
C	1.604608	-0.001655	-2.083628
H	1.093325	-0.874793	-2.475827
H	1.093501	0.870940	-2.477162
H	2.628409	-0.002042	-2.470309
Pd	-0.824443	-0.002077	-0.199879
P	-2.995579	-0.000376	0.102372
C	-4.055627	-0.566571	-1.294486
H	-5.115156	-0.521792	-1.028586
H	-3.876244	0.064821	-2.164869
H	-3.793302	-1.591949	-1.555816
C	-3.665534	-1.042851	1.466129
H	-3.230848	-0.723222	2.413400
H	-4.754465	-0.964683	1.524244
H	-3.387851	-2.083303	1.296384
C	-3.800959	1.609871	0.494367
H	-3.612000	2.315529	-0.314847
H	-4.879816	1.490572	0.624962
H	-3.370403	2.016423	1.409706
C	3.562768	-1.593692	-0.373134
H	4.271106	-0.962288	0.153947
H	3.781688	-2.626222	-0.096510
H	3.751100	-1.498569	-1.442106
C	1.256518	-2.476762	-0.541587
H	1.493366	-2.731409	-1.574526
H	1.468104	-3.356417	0.068171
H	0.186854	-2.253639	-0.469742
H	4.267201	0.973441	0.149113
C	3.555543	1.601811	-0.377110
H	3.742617	1.507030	-1.446343
H	3.770618	2.635337	-0.101200
C	1.245073	2.474388	-0.543539
H	0.176327	2.247255	-0.469262
H	1.454148	3.355915	0.064379
H	1.478572	2.728403	-1.577379

52

6Pd SCF Done: -1191.51800169 A.U.

N	-1.335648	-0.549791	0.005727

C	-1.535794	-1.245871	1.364539
C	-0.928486	-0.323598	2.437158
H	-0.998989	-0.808585	3.411881
H	0.127256	-0.125228	2.221296
H	-1.449911	0.630340	2.497529
C	-1.530148	-1.311712	-1.324569
C	-0.355285	-2.271859	-1.592345
H	-0.390864	-2.585947	-2.636629
H	0.604506	-1.767446	-1.421438
H	-0.382331	-3.169255	-0.984432
C	-2.831916	-2.122637	-1.442106
H	-2.873205	-2.570024	-2.437082
H	-2.872064	-2.936964	-0.721573
H	-3.722305	-1.508340	-1.327618
C	-1.996965	0.755144	-0.004851
C	-3.383921	0.919855	-0.077608
C	-1.210406	1.904644	0.042993
C	-3.955087	2.187551	-0.100887
H	-4.033424	0.061974	-0.129581
C	-1.775353	3.174218	0.025604
H	-0.127384	1.789402	0.090055
C	-3.155310	3.323345	-0.046656
H	-3.601866	4.309500	-0.063653
H	-1.132462	4.044768	0.064760
C	-2.999348	-1.513087	1.778138
H	-3.548480	-2.102773	1.049817
H	-2.991922	-2.073217	2.714875
H	-3.544206	-0.589664	1.962100
C	-0.775630	-2.575669	1.425872
H	0.261024	-2.453547	1.109524
H	-0.773823	-2.924303	2.459202
H	-1.245363	-3.351572	0.825326
C	-1.495792	-0.278457	-2.468032
H	-2.358659	0.383701	-2.465558
H	-0.592639	0.331243	-2.412380
H	-1.488104	-0.811974	-3.418583
Pd	1.187974	-0.064653	0.005588
P	3.344021	0.344536	-0.010145
C	4.400936	-0.704672	-1.094176
H	5.452152	-0.412690	-1.023966
H	4.296766	-1.749012	-0.799165
H	4.068259	-0.606494	-2.127671
C	4.251729	0.192712	1.585486
H	5.313745	0.419151	1.458593
H	3.821307	0.880577	2.313439
H	4.143506	-0.822233	1.968285
C	3.886299	2.022568	-0.542527
H	3.446089	2.771034	0.116620
H	4.975491	2.112857	-0.514707
H	3.535214	2.211360	-1.557148
H	-5.032036	2.283025	-0.163279

53

7Pd SCF Done: -1283.80207164 A.U.

N	-0.899793	0.819972	-0.019727
C	-0.989746	1.544094	-1.378292
C	-0.492052	0.557956	-2.450489
H	-0.474795	1.059401	-3.418949
H	0.521602	0.214097	-2.217184
H	-1.138641	-0.313937	-2.538727
C	-1.016148	1.588813	1.318105
C	0.275987	2.371953	1.615771
H	0.255463	2.697099	2.656948
H	1.158171	1.733142	1.477448
H	0.396402	3.257927	1.002848
C	-2.194345	2.571603	1.418486
H	-2.192413	3.012731	2.416839
H	-2.106658	3.388494	0.705353
H	-3.160883	2.090675	1.283791
C	-1.717429	-0.386513	-0.034343
C	-3.109789	-0.369217	0.030973
C	-1.086709	-1.628528	-0.105345
C	-3.838254	-1.563137	0.027679
H	-3.649382	0.559637	0.099060
C	-1.806274	-2.817463	-0.115083
H	0.002794	-1.647960	-0.148338
C	-3.189479	-2.799069	-0.048029
H	-3.762526	-3.715912	-0.051437
H	-1.280466	-3.761351	-0.171509
C	-2.401792	1.998960	-1.804088
H	-2.877198	2.655405	-1.081177
H	-2.312824	2.552950	-2.739965
H	-3.061305	1.155250	-1.998275
C	-0.062150	2.763048	-1.414936
H	0.947100	2.500964	-1.094536
H	-0.006506	3.122597	-2.442841
H	-0.430921	3.584982	-0.805587
C	-1.147316	0.549495	2.449016
H	-2.098999	0.022600	2.434474
H	-0.343170	-0.186405	2.396576
H	-1.072097	1.066289	3.405472
C	-5.264372	-1.509554	0.104114
N	-6.415099	-1.463430	0.165314
Pd	1.547324	0.012804	0.014285
P	3.618889	-0.720545	0.029951
C	4.961741	0.532057	-0.104958
H	5.946523	0.057820	-0.085025
H	4.847908	1.086449	-1.036668
H	4.884343	1.235952	0.723865
C	4.117165	-1.890743	-1.301658
H	5.164418	-2.186190	-1.196512
H	3.486418	-2.778784	-1.257340
H	3.972650	-1.416657	-2.272603
C	4.176048	-1.649570	1.518865
H	3.546928	-2.529537	1.653887

H	5.218611	-1.962925	1.419631
H	4.072917	-1.016523	2.400288

65

11Pd SCF Done: -1499.14058112 A.U.

N	-0.614317	-0.547695	0.896623
C	-0.523787	-0.447503	2.409208
C	-0.784297	0.980322	2.880975
H	-1.827385	1.266125	2.738738
H	-0.552122	1.054574	3.944726
H	-0.152462	1.688362	2.337159
H	0.517778	-0.675764	2.632745
C	-1.401547	-1.423663	3.201956
H	-1.258824	-2.462407	2.905590
H	-1.137445	-1.346409	4.258180
H	-2.459783	-1.178731	3.109794
C	-1.893818	-0.039675	0.400057
C	-1.941724	1.203283	-0.214689
C	-3.092578	-0.754601	0.512858
C	-3.131010	1.745225	-0.695070
H	-1.012337	1.760807	-0.320542
C	-4.283810	-0.234662	0.037212
H	-3.107426	-1.736282	0.962592
C	-4.316278	1.023816	-0.568861
H	-3.112646	2.717449	-1.164868
H	-5.205700	-0.794885	0.125514
O	-5.535264	1.448369	-1.002983
C	-5.625155	2.722891	-1.631885
H	-6.672712	2.855846	-1.890857
H	-5.314377	3.523336	-0.954690
H	-5.018951	2.761461	-2.541230
C	-0.394511	-1.955387	0.403148
H	-1.229594	-2.560522	0.767962
C	0.886396	-2.653314	0.915981
C	-0.419303	-2.067566	-1.139486
H	0.958051	-2.566486	2.000089
C	2.156523	-2.086335	0.267498
C	0.767133	-4.149130	0.558438
H	-1.290308	-1.537581	-1.526555
C	0.854954	-1.502671	-1.782336
C	-0.537239	-3.561425	-1.497230
H	3.031521	-2.606758	0.669394
H	2.270337	-1.021466	0.513243
C	2.086073	-2.255672	-1.257736
H	-0.118455	-4.581612	1.034599
H	1.636342	-4.683748	0.952570
C	0.688468	-4.322209	-0.966280
H	0.956883	-0.431582	-1.559617
H	0.783514	-1.599761	-2.870235
H	-1.455215	-3.980660	-1.073338
H	-0.605972	-3.671012	-2.583475
H	2.990028	-1.840385	-1.712379
C	1.963624	-3.748024	-1.607643

H	0.597367	-5.384340	-1.211030
H	1.926703	-3.877398	-2.693995
H	2.841580	-4.293239	-1.246578
Pd	1.154263	1.045507	0.249302
P	2.724263	2.495282	-0.253075
C	2.461993	4.271914	0.156820
H	3.314002	4.881048	-0.156924
H	1.561072	4.628755	-0.342812
H	2.320593	4.377931	1.232627
C	3.174041	2.628831	-2.034723
H	3.977599	3.354083	-2.188227
H	3.495006	1.652415	-2.398349
H	2.298292	2.934930	-2.607292
C	4.390244	2.227032	0.486583
H	4.754012	1.238544	0.205084
H	5.101671	2.984065	0.145919
H	4.315303	2.266792	1.573422

49

12Pd SCF Done: -1077.11966109 A.U.

N	1.584718	-0.007019	0.127608
C	2.006853	1.452673	-0.218031
C	1.602398	2.385498	0.941883
H	2.177072	2.210568	1.847126
H	1.787921	3.412857	0.628105
H	0.540942	2.290610	1.170485
C	3.517876	1.637593	-0.435656
H	3.875937	1.144059	-1.335699
H	3.725938	2.703137	-0.547681
H	4.099993	1.279994	0.414037
C	2.107445	-1.153229	-0.788293
C	1.982981	-0.810730	-2.277727
H	2.673875	-0.036473	-2.598481
H	2.221226	-1.706367	-2.852184
H	0.965302	-0.513239	-2.529376
C	1.232759	-2.405632	-0.573018
H	0.169412	-2.150750	-0.630972
H	1.456799	-3.130294	-1.357264
H	1.424024	-2.888386	0.379929
C	3.563202	-1.576928	-0.501179
H	3.816892	-2.399745	-1.171884
H	4.285430	-0.784209	-0.670728
H	3.683996	-1.944471	0.516677
C	1.246559	1.990593	-1.446322
H	1.611492	1.613976	-2.393859
H	0.180793	1.751240	-1.363087
H	1.349881	3.076276	-1.471090
C	1.969450	-0.305644	1.516873
C	1.132377	0.059221	2.710605
C	1.315282	-1.380701	2.345106
H	3.034138	-0.207353	1.701747
H	0.149799	0.471047	2.521965
H	1.646858	0.471253	3.569376

H	1.959934	-2.016158	2.938785
H	0.439873	-1.868462	1.943225
Pd	-0.816183	-0.015291	-0.040633
P	-3.009333	0.008168	-0.109880
C	-3.885082	1.174699	1.016086
H	-4.969444	1.099190	0.898472
H	-3.618747	0.949665	2.048991
H	-3.569367	2.194839	0.796538
C	-3.893355	-1.558973	0.288329
H	-4.977074	-1.431031	0.221431
H	-3.579322	-2.337744	-0.407044
H	-3.629937	-1.876095	1.297613
C	-3.808750	0.437453	-1.713814
H	-3.496306	-0.275896	-2.476785
H	-4.898589	0.420633	-1.629417
H	-3.487315	1.431972	-2.023951

48

13Pd SCF Done: -1039.03284967 A.U.

N	1.574986	0.101367	0.002750
C	2.039602	-1.250005	-0.556500
C	1.752975	-1.311923	-2.071354
H	2.365682	-0.639409	-2.665734
H	1.961251	-2.324143	-2.417607
H	0.700859	-1.100650	-2.273188
C	3.526468	-1.569165	-0.308321
H	3.722725	-1.737066	0.749329
H	3.783470	-2.490940	-0.833994
H	4.194574	-0.787757	-0.659840
C	1.910632	0.456905	1.435739
C	1.471165	-0.575729	2.476585
H	2.155700	-1.420374	2.548415
H	1.460450	-0.085118	3.451605
H	0.464517	-0.942732	2.275979
C	3.343273	0.919434	1.763374
H	3.387637	1.147247	2.830689
H	4.098405	0.165426	1.557564
H	3.615031	1.828953	1.235747
C	1.218247	-2.410794	0.045934
H	1.463616	-2.619934	1.080662
H	0.146365	-2.196072	-0.020575
H	1.423376	-3.318309	-0.524259
C	1.797456	1.271045	-0.940958
C	3.223426	1.563792	-1.448789
H	3.152550	2.358481	-2.195097
H	3.897280	1.908875	-0.669194
H	3.685096	0.710493	-1.936952
C	1.184043	2.570406	-0.409127
H	1.127328	3.286228	-1.230847
H	0.168324	2.400940	-0.040869
H	1.778508	3.028189	0.382472
H	1.211990	1.016711	-1.820790
H	1.275901	1.320116	1.623983

Pd	-0.847904	0.014303	0.012150
P	-3.037644	0.024981	-0.015559
C	-3.898767	1.215324	1.096566
H	-3.595527	2.231458	0.843593
H	-4.984604	1.128723	1.002808
H	-3.610284	1.019661	2.129496
C	-3.902141	-1.536898	0.441125
H	-4.987713	-1.417288	0.391736
H	-3.596226	-2.331745	-0.239429
H	-3.617637	-1.825337	1.453265
C	-3.869669	0.406058	-1.614875
H	-4.957557	0.382980	-1.508972
H	-3.562225	1.394449	-1.956850
H	-3.565764	-0.324984	-2.364326

51

14Pd SCF Done: -1078.34760676 A.U.

N	1.533705	-0.007592	0.086360
C	1.751365	-0.551573	1.496077
C	1.133945	-1.922859	1.792845
H	1.753002	-2.754093	1.458922
H	1.039929	-2.009069	2.877446
H	0.135021	-2.015660	1.364297
C	3.167642	-0.529928	2.106927
H	3.668187	0.427101	2.004341
H	3.065596	-0.722293	3.177739
H	3.820922	-1.298423	1.702479
C	1.878362	1.512039	0.015096
C	1.511037	2.207763	1.347155
H	2.136539	1.917904	2.187557
H	1.643592	3.280644	1.210916
H	0.463666	2.034569	1.602772
C	1.004772	2.240398	-1.035728
H	-0.037193	1.906676	-0.961134
H	1.031970	3.311369	-0.831069
H	1.336458	2.105593	-2.057545
C	3.354353	1.849240	-0.285620
H	3.463650	2.935575	-0.288916
H	4.040896	1.455961	0.458772
H	3.676789	1.494287	-1.260067
C	2.031100	-0.908616	-1.065788
C	1.131630	-2.159722	-1.201983
H	1.355449	-2.647575	-2.151773
H	1.285953	-2.895569	-0.423324
H	0.074404	-1.872798	-1.204956
C	3.483254	-1.402855	-0.907434
H	3.785679	-1.909453	-1.825944
H	4.188388	-0.596654	-0.724612
H	3.572738	-2.127358	-0.101436
C	1.905454	-0.226350	-2.440855
H	2.173415	-0.960852	-3.200100
H	0.879768	0.085818	-2.630823
H	2.569437	0.620144	-2.577110

Pd	-0.915824	-0.029716	-0.013128
P	-3.106773	0.009692	0.020601
C	-3.944585	-0.220084	1.645875
H	-3.623176	0.563167	2.332689
H	-5.032031	-0.182210	1.538991
H	-3.657586	-1.183517	2.067668
C	-3.998667	-1.247822	-0.989291
H	-5.082073	-1.129866	-0.901854
H	-3.708844	-1.143359	-2.035068
H	-3.717240	-2.246550	-0.654861
C	-3.939117	1.546858	-0.562958
H	-3.651111	1.743856	-1.595839
H	-5.026927	1.455384	-0.503135
H	-3.614479	2.388873	0.048700
H	1.159505	0.124157	2.100704

54

15Pd SCF Done: -1117.65437837 A.U.

N	-1.501798	-0.000118	-0.000207
C	-1.868632	-0.816091	-1.292257
C	-1.636894	-2.334442	-1.128332
H	-2.293147	-2.813190	-0.412463
H	-1.845636	-2.795654	-2.092864
H	-0.600859	-2.554189	-0.878003
C	-3.325430	-0.656366	-1.779431
H	-3.568783	0.357915	-2.077752
H	-3.462698	-1.286213	-2.660671
H	-4.051388	-0.972055	-1.035154
C	-1.872212	1.526060	-0.061236
C	-1.640844	2.143304	-1.458137
H	-2.294770	1.760408	-2.231465
H	-1.852876	3.208627	-1.376108
H	-0.603978	2.039268	-1.771996
C	-0.920993	2.373989	0.830032
H	0.101646	1.981509	0.777764
H	-0.901472	3.393941	0.446622
H	-1.213302	2.438772	1.869517
C	-3.329821	1.864924	0.320454
H	-3.469555	2.942646	0.215008
H	-4.054973	1.376331	-0.324506
H	-3.572240	1.616314	1.348288
C	-0.914552	-0.466711	-2.469736
H	-1.206336	0.401157	-3.045864
H	0.107310	-0.314976	-2.101234
H	-0.892511	-1.308322	-3.161785
C	-1.871077	-0.711273	1.351704
C	-0.917815	-1.905699	1.639552
H	-0.896893	-2.083712	2.714558
H	-1.209233	-2.838736	1.176179
H	0.104387	-1.663016	1.324748
C	-3.327958	-1.213486	1.454577
H	-3.466992	-1.660617	2.440926
H	-4.054412	-0.411865	1.353488

H	-3.569230	-1.980127	0.725825
C	-1.640912	0.190001	2.584839
H	-1.850443	-0.414686	3.466330
H	-0.604961	0.516766	2.650912
H	-2.297347	1.049247	2.640925
Pd	0.988055	0.001052	0.002115
P	3.182175	0.000443	0.000013
C	4.034484	-0.164544	-1.625694
H	3.734442	0.657292	-2.276179
H	5.121262	-0.152634	-1.506824
H	3.734416	-1.100387	-2.097722
C	4.037235	-1.324942	0.953326
H	5.123818	-1.227783	0.880882
H	3.739704	-1.266385	2.000539
H	3.736541	-2.299028	0.566988
C	4.038182	1.489082	0.669298
H	3.739851	1.641321	1.706785
H	5.124720	1.378443	0.618724
H	3.738787	2.366605	0.095769

49

16Pd SCF Done: -1077.17015557 A.U.

N	-1.572465	0.012045	0.206088
C	-2.162885	-1.226792	-0.518852
C	-2.508721	-0.711847	-1.941375
C	-1.923048	0.683407	-2.027427
C	-2.026262	1.231122	-0.600961
H	-3.590549	-0.679565	-2.076546
H	-2.114336	-1.379637	-2.706783
H	-0.869419	0.646285	-2.314471
H	-2.442483	1.326532	-2.738896
C	-1.115003	-2.339991	-0.635264
H	-0.801655	-2.729213	0.330117
H	-1.531573	-3.168264	-1.214046
H	-0.223710	-1.969418	-1.149786
C	-3.438842	-1.808633	0.116307
H	-3.829244	-2.573227	-0.557752
H	-3.263794	-2.286692	1.077900
H	-4.217897	-1.059019	0.244416
C	-1.097758	2.447482	-0.488399
H	-1.273604	3.091625	-1.353006
H	-1.263007	3.046178	0.402459
H	-0.049762	2.128291	-0.503577
C	-3.471077	1.673646	-0.304577
H	-3.601000	1.981261	0.731855
H	-3.713008	2.532647	-0.932845
H	-4.200829	0.895328	-0.517546
C	-1.864501	0.046012	1.679716
H	-2.946431	-0.024981	1.830544
C	-1.202091	-1.121773	2.420409
H	-1.421125	-1.037523	3.486101
H	-1.546083	-2.098086	2.095366
H	-0.117582	-1.080580	2.282236

C	-1.394590	1.332575	2.365323
H	-1.964694	2.209063	2.073218
H	-1.516758	1.214384	3.442797
H	-0.337795	1.515893	2.159928
Pd	0.847891	-0.030626	0.015623
P	3.036671	-0.014432	-0.121830
C	3.841056	1.506762	-0.782537
H	4.929301	1.403388	-0.804003
H	3.478005	1.698274	-1.792523
H	3.572470	2.357806	-0.156264
C	3.993181	-0.236449	1.438122
H	3.730062	-1.192398	1.891373
H	5.069704	-0.211144	1.248725
H	3.732485	0.557165	2.138633
C	3.838401	-1.293009	-1.180389
H	3.475870	-1.194626	-2.203896
H	4.926761	-1.189177	-1.171726
H	3.568717	-2.284666	-0.816385

52

17Pd SCF Done: -1116.48577685 A.U.

N	1.574903	0.056038	0.016943
C	2.029455	-0.769704	-1.212100
C	2.234393	-2.199371	-0.651331
C	1.574052	-2.196019	0.706567
C	1.856917	-0.793260	1.258248
H	3.299457	-2.414171	-0.550448
H	1.821312	-2.947806	-1.327109
H	0.492650	-2.327746	0.614177
H	1.951245	-2.970827	1.374757
C	0.924218	-0.816523	-2.278459
H	0.729762	0.152821	-2.729060
H	1.219514	-1.501972	-3.076592
H	-0.015277	-1.178463	-1.844251
C	3.336974	-0.351807	-1.903913
H	3.578967	-1.120316	-2.641322
H	3.257205	0.591584	-2.439864
H	4.171292	-0.292755	-1.207885
C	0.905769	-0.570947	2.443836
H	0.954670	-1.455322	3.083325
H	1.160173	0.287226	3.058733
H	-0.127932	-0.460940	2.096098
C	3.301627	-0.722024	1.789762
H	3.494294	0.221297	2.297537
H	3.448970	-1.517257	2.523462
H	4.046506	-0.847426	1.005901
C	1.861471	1.553234	0.035524
C	1.206104	2.208080	-1.193483
H	1.317648	3.290979	-1.120920
H	1.663330	1.898495	-2.129525
H	0.138318	1.972657	-1.231223
C	1.198383	2.193985	1.268167
H	1.739498	1.975932	2.185861

H	1.200774	3.277179	1.142518
H	0.162006	1.867329	1.374786
C	3.344342	2.008500	0.059540
H	3.360623	3.082800	0.253807
H	3.920780	1.531557	0.844615
H	3.855010	1.851867	-0.883983
Pd	-0.999167	0.004036	-0.054628
P	-3.190950	-0.014624	-0.016469
C	-4.010710	-0.961220	1.335526
H	-5.099576	-0.899806	1.258080
H	-3.704661	-2.006240	1.282704
H	-3.695176	-0.561087	2.299301
C	-4.069366	-0.707976	-1.480667
H	-5.153894	-0.662062	-1.350082
H	-3.789672	-0.143518	-2.370431
H	-3.768169	-1.745649	-1.625556
C	-4.054942	1.604955	0.145766
H	-3.778192	2.247924	-0.689982
H	-5.140440	1.474354	0.156381
H	-3.741526	2.091407	1.069640

40

18Pd SCF Done: -959.193651184 A.U.

N	1.847801	0.314196	-0.000739
C	2.664990	-2.554261	-0.001259
C	2.130606	-1.853835	1.244029
C	2.408510	-0.355604	1.207193
C	2.402710	-0.356989	-1.210558
C	2.124090	-1.855120	-1.244437
H	1.974853	0.106791	2.088750
H	1.049428	-2.008132	1.318682
H	2.582016	-2.264155	2.150892
H	3.759926	-2.510955	-0.004216
H	2.387385	-3.610767	0.000028
H	1.965125	0.104625	-2.090568
H	3.490037	-0.184556	-1.246322
H	1.042496	-2.009259	-1.313334
H	2.570563	-2.266538	-2.153249
H	3.495921	-0.182675	1.237768
C	2.241955	1.767098	-0.002497
H	3.342215	1.801209	-0.005482
C	1.746306	2.499039	1.246280
H	2.208555	2.138584	2.163268
H	1.985823	3.559403	1.157629
H	0.662178	2.395948	1.339226
C	1.739582	2.497855	-1.249266
H	1.980167	3.558195	-1.163168
H	2.196411	2.136108	-2.168457
H	0.654916	2.395459	-1.336133
Pd	-0.441756	0.083049	0.002958
P	-2.627544	-0.130455	0.000397
C	-3.351360	-1.431384	-1.085369
H	-2.954229	-2.405049	-0.797667

H	-4.441651	-1.449063	-1.007657
H	-3.067428	-1.238651	-2.120115
C	-3.447289	-0.540823	1.598758
H	-3.225309	0.236687	2.329973
H	-4.530567	-0.622620	1.476715
H	-3.055331	-1.485941	1.975046
C	-3.628285	1.327124	-0.518444
H	-3.353261	1.612160	-1.534135
H	-4.698038	1.104543	-0.483251
H	-3.413780	2.167251	0.142340

52

19Pd SCF Done: -1116.48577685 A.U.

N	-1.442367	0.328781	-0.003315
C	-2.538621	-2.488574	0.019083
C	-1.860429	-1.903332	-1.194783
C	-1.942735	-0.365785	-1.314626
C	-1.950399	-0.341744	1.317390
C	-1.867572	-1.881142	1.226002
H	-0.802817	-2.185298	-1.172779
H	-2.277881	-2.314869	-2.115694
H	-3.613047	-2.288821	0.014098
H	-2.425115	-3.575640	0.029386
H	-0.809898	-2.163415	1.215365
H	-2.290344	-2.275867	2.151834
C	-1.755518	1.825627	-0.017583
H	-2.842462	1.940791	-0.021202
C	-1.221852	2.576910	-1.246402
H	-1.763995	2.361204	-2.158972
H	-1.333656	3.645630	-1.065502
H	-0.161749	2.370045	-1.402630
C	-1.227654	2.598670	1.200282
H	-1.337324	3.664053	0.999467
H	-1.774916	2.400112	2.113694
H	-0.168658	2.393877	1.366223
Pd	0.929360	0.066533	0.003847
P	3.111241	-0.175886	0.002647
C	3.846729	-1.297840	1.266305
H	3.433132	-2.299327	1.145603
H	4.934900	-1.343002	1.170895
H	3.587494	-0.939077	2.262645
C	3.892424	-0.846758	-1.525941
H	3.661923	-0.191267	-2.366085
H	4.977363	-0.925190	-1.416253
H	3.480146	-1.833145	-1.739607
C	4.141806	1.329723	0.262317
H	3.894225	1.776354	1.225494
H	5.207728	1.087490	0.240321
H	3.922108	2.057942	-0.518659
C	-3.379068	0.020233	-1.715199
H	-3.499296	1.090659	-1.867532
H	-3.615277	-0.466022	-2.663346
H	-4.122668	-0.300467	-0.990182

C	-3.389032	0.051765	1.702497
H	-4.128325	-0.279945	0.978031
H	-3.631749	-0.418778	2.656907
H	-3.509157	1.124647	1.836496
C	-1.020598	-0.021011	2.515188
H	-1.252732	0.902247	3.031202
H	-1.116441	-0.823468	3.247717
H	0.024385	0.011486	2.187636
C	-1.006184	-0.067003	-2.512775
H	0.037451	-0.032641	-2.181060
H	-1.101093	-0.880912	-3.232713
H	-1.232643	0.848500	-3.044792

43

20Pd SCF Done: -998.521498671 A.U.

N	-1.748779	-0.033801	0.000234
C	-2.044114	2.923203	-0.001507
C	-1.652769	2.137116	1.246806
C	-2.202711	0.714469	1.205168
C	-2.198692	0.714081	-1.206460
C	-1.646921	2.135944	-1.247238
H	-1.875126	0.179102	2.091337
H	-0.561572	2.089556	1.325954
H	-2.026504	2.625164	2.150729
H	-3.127653	3.087284	-0.004254
H	-1.572869	3.908812	-0.000771
H	-1.869800	0.177430	-2.091390
H	-3.295253	0.762113	-1.230835
H	-0.555337	2.087378	-1.321091
H	-2.015758	2.623513	-2.153423
H	-3.299417	0.760790	1.226920
C	-2.238183	-1.481566	-0.000522
C	-1.692527	-2.195849	1.245361
H	-2.165173	-1.851932	2.164630
H	-1.886698	-3.265667	1.161168
H	-0.613110	-2.042709	1.327591
C	-1.678307	-2.198921	-1.238248
H	-1.877465	-3.267992	-1.156033
H	-2.136934	-1.853934	-2.164179
H	-0.597321	-2.049540	-1.306195
C	-3.772859	-1.602330	-0.009119
H	-4.216122	-1.155544	-0.898936
H	-4.043689	-2.659200	-0.005434
H	-4.226785	-1.146642	0.870775
Pd	0.594230	-0.045683	0.001411
P	2.788974	-0.076978	-0.000087
C	3.640179	-0.319271	1.616231
H	4.726662	-0.316853	1.494717
H	3.353018	0.479767	2.300023
H	3.328760	-1.269412	2.050663
C	3.624591	-1.372443	-1.009524
H	3.326129	-1.267107	-2.052755
H	4.712292	-1.291045	-0.935518

H 3.313019 -2.357953 -0.662782
C 3.661006 1.427983 -0.608503
H 3.370704 2.284789 -0.000112
H 4.746016 1.302767 -0.561383
H 3.366098 1.625279 -1.639337
45
21Pd SCF Done: -999.713120547 A.U.
N 1.782392 -0.000153 -0.000291
C 2.142233 -1.346719 -0.550465
C 1.147657 -1.790511 -1.625261
H 1.204408 -1.155990 -2.512002
H 1.364137 -2.815081 -1.935757
H 0.119062 -1.748120 -1.246720
C 3.567266 -1.525926 -1.101786
H 4.341766 -1.252807 -0.391077
H 3.711601 -2.576979 -1.359782
H 3.715323 -0.946105 -2.014240
C 2.142444 0.196144 1.441064
C 1.149043 -0.514931 2.362763
H 1.207118 -1.599859 2.255151
H 1.366192 -0.272588 3.405379
H 0.119889 -0.209129 2.138082
C 3.568015 -0.190772 1.871113
H 3.712955 0.111906 2.910106
H 3.716441 -1.270867 1.825662
H 4.341875 0.288282 1.278485
C 2.141799 1.150236 -0.890733
C 3.567300 1.716486 -0.771455
H 3.711213 2.465383 -1.552839
H 3.716087 2.216806 0.186784
H 4.341534 0.964254 -0.890868
C 1.148653 2.304079 -0.735316
H 1.365475 3.085586 -1.466948
H 0.119249 1.957249 -0.886609
H 1.207508 2.754001 0.257828
H 2.024716 0.765315 -1.904610
H 2.025871 1.266749 1.614135
H 2.026744 -2.031525 0.290591
Pd -0.779735 0.000547 0.000299
P -2.974240 -0.000096 0.000221
C -3.831438 1.281003 1.010909
H -4.917895 1.185609 0.932923
H -3.535285 1.176443 2.054919
H -3.531992 2.271891 0.668754
C -3.831125 -1.516702 0.602819
H -4.917588 -1.402740 0.555984
H -3.532108 -2.368332 -0.008722
H -3.534458 -1.715299 1.632897
C -3.829628 0.234889 -1.615274
H -3.528912 -0.556387 -2.302339
H -4.916167 0.214310 -1.494673
H -3.533413 1.191707 -2.045798

39
22Pd SCF Done: -921.081252336 A.U.

N	1.789850	0.173697	-0.432037
C	2.542986	-1.070272	-0.010601
C	2.114504	-2.170728	-0.990360
H	2.424530	-1.930904	-2.011013
H	2.572954	-3.120971	-0.714544
H	1.029007	-2.291197	-0.979084
C	4.064473	-0.889184	-0.099510
H	4.436566	-0.175975	0.636674
H	4.555660	-1.844318	0.092150
H	4.365087	-0.549986	-1.093299
C	2.354281	1.513953	-0.084640
C	2.193771	1.857557	1.392160
H	2.497900	2.892130	1.561641
H	1.149791	1.745447	1.693785
H	2.807589	1.226466	2.032203
C	1.674921	2.563353	-0.962644
H	2.117230	3.544007	-0.780292
H	1.795115	2.332124	-2.023887
H	0.605381	2.613489	-0.744674
H	3.421026	1.528068	-0.328408
C	2.147985	-1.494974	1.408235
H	2.461053	-0.771160	2.157345
H	1.064595	-1.610573	1.477937
H	2.620626	-2.449408	1.648824
H	1.795884	0.146866	-1.448960
Pd	-0.462762	0.033963	-0.148096
P	-2.647480	-0.103178	0.057126
C	-3.680491	0.966076	-1.031082
H	-4.745575	0.811348	-0.839518
H	-3.431498	2.012397	-0.853094
H	-3.465632	0.736305	-2.074876
C	-3.428823	-1.742787	-0.253989
H	-3.015792	-2.477571	0.437416
H	-4.513191	-1.694733	-0.123852
H	-3.202350	-2.064998	-1.270548
C	-3.373985	0.301426	1.701327
H	-4.462252	0.198813	1.687993
H	-2.958683	-0.367529	2.455329
H	-3.112447	1.324833	1.971138

36
23Pd SCF Done: -881.751302260 A.U.

N	-1.927127	-0.008926	-0.370319
C	-2.524891	1.326955	-0.081167
C	-1.906071	2.367497	-1.008806
H	-2.036107	2.090013	-2.057683
H	-2.385574	3.335747	-0.854637
H	-0.835872	2.467973	-0.813629
C	-2.580416	-1.173011	0.305580
C	-1.982331	-2.461523	-0.251150
H	-2.383110	-3.325420	0.280939

H	-2.227486	-2.574698	-1.311277
H	-0.894738	-2.457327	-0.150713
C	-4.105593	-1.189119	0.173822
H	-4.502060	-2.103943	0.617188
H	-4.577909	-0.348119	0.681021
H	-4.403786	-1.167640	-0.878078
H	-2.318888	-1.102198	1.362428
C	-2.340200	1.704550	1.383881
H	-2.840563	1.004536	2.053977
H	-1.276878	1.720031	1.635115
H	-2.758720	2.695856	1.565731
H	-2.033866	-0.161360	-1.371967
H	-3.598026	1.292160	-0.297441
Pd	0.329208	-0.040631	-0.144138
P	2.517032	-0.063712	0.075078
C	3.314394	1.440310	0.780118
H	2.912987	1.629879	1.775812
H	4.398918	1.319341	0.845232
H	3.085328	2.299955	0.149997
C	3.527317	-0.288833	-1.449395
H	4.595960	-0.280874	-1.219196
H	3.267777	-1.237686	-1.919323
H	3.304982	0.513525	-2.153172
C	3.252084	-1.364597	1.153114
H	2.982122	-2.348380	0.768646
H	4.341208	-1.278187	1.189769
H	2.849372	-1.269367	2.161713

39

24Pd SCF Done: -1380.66083353 A.U.

N	-1.645005	0.115945	-0.088236
C	-2.024975	1.279727	0.797627
C	-1.268760	2.536455	0.371653
H	-1.701947	2.979094	-0.525188
H	-1.323490	3.270989	1.176347
H	-0.215506	2.308392	0.179948
C	-3.515820	1.582031	0.954100
H	-4.032223	0.842110	1.560137
H	-3.626902	2.546143	1.451800
H	-4.008304	1.648093	-0.016328
C	-2.195990	-1.272931	0.261498
C	-1.876364	-1.519505	1.743117
H	-2.489211	-0.909387	2.405721
H	-2.085635	-2.565152	1.969582
H	-0.821980	-1.328476	1.952466
C	-3.698314	-1.474966	0.012593
H	-3.932725	-2.525459	0.189661
H	-4.323634	-0.885306	0.675136
H	-3.966871	-1.244931	-1.016969
H	-1.619583	0.973801	1.761998
Cl	-2.116579	0.523576	-1.782372
C	-1.424350	-2.291031	-0.588064
H	-1.647520	-2.176006	-1.647759

H	-0.345974	-2.169177	-0.446429
H	-1.707131	-3.300816	-0.288432
Pd	0.740376	0.036517	-0.078881
P	2.928691	-0.027912	0.056130
C	3.829802	1.577280	0.003197
H	3.497061	2.206434	0.828907
H	4.909904	1.425711	0.077159
H	3.601325	2.090019	-0.931267
C	3.664143	-0.774571	1.570473
H	3.326225	-1.806521	1.666206
H	4.756366	-0.754816	1.527785
H	3.328031	-0.221300	2.447521
C	3.831371	-0.961927	-1.249365
H	4.911370	-0.925209	-1.084190
H	3.501410	-2.000901	-1.244819
H	3.601703	-0.534209	-2.225398

36

25Pd SCF Done: -1341.33667328 A.U.

N	-1.745818	0.050920	-0.086059
C	-2.251878	1.433320	0.254337
C	-1.960371	1.743343	1.725068
H	-0.899892	1.598554	1.941928
H	-2.208893	2.788531	1.910452
H	-2.547414	1.138031	2.413136
C	-1.601179	2.487008	-0.636211
H	-1.806729	2.317087	-1.690653
H	-1.993758	3.468947	-0.368025
H	-0.518189	2.488761	-0.489715
C	-2.296454	-1.036078	0.807487
C	-3.818389	-1.067862	0.930332
H	-4.097451	-1.867849	1.617120
H	-4.229864	-0.138947	1.323072
H	-4.284534	-1.274267	-0.033371
H	-3.332242	1.450723	0.090074
Cl	-2.302464	-0.304426	-1.778140
C	-1.737663	-2.399791	0.419687
H	-2.191413	-2.772921	-0.498160
H	-0.655161	-2.343628	0.280229
H	-1.949939	-3.110565	1.219639
H	-1.863800	-0.774363	1.773753
Pd	0.563382	-0.030973	-0.077088
P	2.755905	-0.075467	0.057910
C	3.498260	-0.416835	1.707935
H	3.154934	-1.387450	2.066129
H	4.590138	-0.417025	1.655220
H	3.172209	0.344744	2.416472
C	3.665344	1.455958	-0.409425
H	4.745040	1.319914	-0.305883
H	3.432319	1.714240	-1.442567
H	3.343086	2.277988	0.229917
C	3.639460	-1.319327	-0.973153
H	4.721129	-1.251715	-0.830588

H	3.302247	-2.319926	-0.702158
H	3.403860	-1.151721	-2.024182

30

26Pd SCF Done: -1722.26232188 A.U.

N	-1.671315	-0.306050	0.000154
C	-2.440458	1.034769	-0.000413
C	-1.974329	1.787602	-1.251443
H	-2.329681	1.316793	-2.165619
H	-2.380449	2.798531	-1.209035
H	-0.885176	1.848857	-1.283215
C	-3.955319	0.855298	-0.001781
H	-4.419928	1.841465	-0.001145
H	-4.295041	0.323015	-0.889026
H	-4.296315	0.321297	0.883957
Cl	-2.142208	-1.293242	1.428629
C	-1.976844	1.787560	1.251597
H	-2.335211	1.317521	2.164989
H	-0.887724	1.847533	1.286201
H	-2.381726	2.798921	1.207689
Cl	-2.141816	-1.294320	-1.427830
Pd	0.627026	-0.166750	-0.000136
P	2.809793	0.089867	-0.000156
C	3.577262	0.745150	1.538318
H	4.661792	0.828458	1.430632
H	3.160743	1.727502	1.761037
H	3.347689	0.078775	2.369777
C	3.521830	1.231980	-1.254388
H	4.610054	1.282174	-1.165203
H	3.257676	0.883495	-2.252820
H	3.102434	2.228607	-1.116654
C	3.833214	-1.412886	-0.282786
H	4.898677	-1.169816	-0.262719
H	3.616830	-2.149731	0.490727
H	3.580732	-1.846865	-1.250255

42

27Pd SCF Done: -1419.98160849 A.U.

N	-1.567730	-0.004481	-0.173265
C	-2.125275	1.332344	0.381533
C	-1.300458	2.457460	-0.267349
H	-1.496333	2.548452	-1.333033
H	-1.559877	3.404744	0.206185
H	-0.230129	2.275999	-0.124965
C	-3.613663	1.594016	0.104313
H	-4.268708	0.946114	0.678664
H	-3.834676	2.622051	0.395025
H	-3.855241	1.490295	-0.951488
C	-2.040771	-1.382941	0.355875
C	-3.560004	-1.567163	0.369881
H	-3.782296	-2.619413	0.553173
H	-4.035590	-0.991570	1.160961
H	-4.003683	-1.292278	-0.586625
Cl	-1.911029	0.022491	-1.956625

C	-1.411539	-2.469098	-0.537280
H	-1.893869	-2.540080	-1.508502
H	-0.345984	-2.273505	-0.684642
H	-1.519390	-3.431941	-0.038371
C	-1.882626	1.404488	1.895301
H	-2.582121	0.791574	2.457026
H	-0.861787	1.124617	2.153897
H	-2.039405	2.437663	2.205953
C	-1.470187	-1.605914	1.768646
H	-1.979291	-1.034509	2.535476
H	-1.594767	-2.660508	2.015141
H	-0.403609	-1.370692	1.794664
Pd	0.816997	0.028201	-0.088907
P	3.006090	0.012167	0.042342
C	3.925442	1.231273	-0.987550
H	5.004866	1.132836	-0.845260
H	3.685001	1.071506	-2.038717
H	3.616986	2.240903	-0.715682
C	3.761408	0.331037	1.691433
H	3.415683	-0.422006	2.399778
H	4.852864	0.301250	1.636830
H	3.445538	1.310320	2.051362
C	3.873548	-1.543064	-0.427135
H	4.956828	-1.435726	-0.326657
H	3.531275	-2.356026	0.213414
H	3.630844	-1.795402	-1.459519

64

28Pd SCF Done: -1348.85887785 A.U.

N	0.890376	1.157837	-0.011806
C	0.965933	2.144686	1.185610
C	0.446513	3.490537	0.612994
C	-0.064460	3.181765	-0.779805
C	0.816404	2.028005	-1.271176
H	1.259653	4.216141	0.568204
H	-0.014010	4.035641	-1.456727
C	0.038463	1.688995	2.318410
H	0.327739	0.730113	2.742183
H	0.052987	2.430491	3.121176
H	-0.989754	1.592671	1.952557
C	2.374108	2.383654	1.766088
H	2.297974	3.192879	2.494648
H	2.784690	1.520833	2.286051
H	3.089404	2.696867	1.007490
C	0.099808	1.367173	-2.456582
H	-0.186905	2.151234	-3.161309
H	0.704801	0.646966	-2.999852
H	-0.812674	0.865671	-2.114345
C	2.179316	2.570713	-1.742404
H	2.862200	1.775763	-2.037686
H	2.022382	3.206517	-2.615391
H	2.674227	3.172820	-0.983011
C	2.006046	0.143975	0.005437

H	2.944340	0.704405	0.044536
C	2.124902	-0.772255	-1.241023
C	2.008055	-0.800231	1.242029
H	2.116142	-0.176275	-2.146295
C	1.012911	-1.828581	-1.307142
C	3.490563	-1.486263	-1.182318
H	1.889746	-0.227283	2.155454
C	0.902279	-1.861322	1.177654
C	3.374040	-1.510825	1.304265
H	1.130033	-2.419731	-2.221119
H	0.028533	-1.349443	-1.350969
C	1.082105	-2.734765	-0.069713
H	4.299773	-0.749159	-1.166503
H	3.619177	-2.083477	-2.090129
C	3.566890	-2.384773	0.057560
H	-0.087268	-1.382695	1.152169
H	0.939123	-2.474505	2.083861
H	4.181015	-0.774687	1.377977
H	3.417652	-2.126249	2.207851
H	0.281860	-3.478990	-0.115462
C	2.448098	-3.438563	-0.011200
H	4.541056	-2.880635	0.098554
H	2.495575	-4.092907	0.865120
H	2.585073	-4.070591	-0.894593
Pd	-1.381492	0.159180	0.060587
P	-3.397439	-0.703505	0.020059
C	-4.599915	-0.216054	1.329252
H	-4.770787	0.859649	1.280524
H	-5.552886	-0.738266	1.208029
H	-4.182841	-0.451402	2.308730
C	-3.486931	-2.539574	0.160459
H	-4.521020	-2.893108	0.127852
H	-2.925682	-2.990503	-0.658284
H	-3.028600	-2.851775	1.099137
C	-4.430956	-0.439746	-1.483133
H	-3.901160	-0.821010	-2.356480
H	-5.395877	-0.946705	-1.396676
H	-4.597340	0.628438	-1.624486
H	-0.323914	3.918161	1.254299
H	-1.101888	2.840252	-0.743899

50

29Pd SCF Done: -1115.25669845 A.U.

N	1.595025	-0.024692	0.000284
C	1.941665	0.813290	1.238633
C	1.920670	2.204300	0.666126
C	1.922257	2.208552	-0.650360
C	1.944633	0.821259	-1.231771
H	1.937156	3.070136	1.314000
H	1.940250	3.078530	-1.292624
C	0.894510	0.729521	2.363087
H	0.949435	-0.194831	2.930885
H	1.062521	1.554720	3.059081

H	-0.120153	0.824669	1.955432
C	3.334359	0.590362	1.867784
H	3.516487	1.388330	2.590715
H	3.395035	-0.353172	2.407966
H	4.128047	0.628755	1.124584
C	0.900159	0.744768	-2.359212
H	1.069871	1.574355	-3.049539
H	0.956399	-0.175981	-2.932724
H	-0.115570	0.837317	-1.953518
C	3.338809	0.602447	-1.859127
H	3.400649	-0.337419	-2.405542
H	3.522707	1.405258	-2.576225
H	4.130753	0.635747	-1.113820
C	1.903511	-1.513784	-0.004065
C	1.252113	-2.165816	1.227964
H	1.311668	-3.250307	1.129042
H	1.757959	-1.898394	2.153004
H	0.198541	-1.884944	1.305371
C	1.253811	-2.157965	-1.241068
H	1.761163	-1.884970	-2.163652
H	1.312751	-3.243070	-1.148820
H	0.200427	-1.876238	-1.318409
C	3.391295	-1.947898	-0.004333
H	3.418647	-3.039537	-0.007141
H	3.932087	-1.614466	-0.883081
H	3.931076	-1.618996	0.876744
Pd	-0.978786	0.028565	-0.002978
P	-3.170868	-0.000587	0.000129
C	-4.004824	-1.636764	-0.153962
H	-5.092780	-1.528534	-0.140222
H	-3.701842	-2.111036	-1.087614
H	-3.696664	-2.279026	0.671257
C	-4.032355	0.665611	1.486829
H	-3.748923	1.708217	1.632234
H	-5.118281	0.598640	1.378851
H	-3.722427	0.101374	2.366685
C	-4.040129	0.936293	-1.327727
H	-5.125542	0.851673	-1.227053
H	-3.754804	1.987167	-1.274850
H	-3.737539	0.548656	-2.300740

54

33Pd SCF Done: -1117.67266676 A.U.

N	-1.503442	-0.227596	0.025610
C	-1.867838	-0.359296	-1.488870
C	-1.487154	-1.760597	-2.024994
H	-2.100931	-2.568008	-1.633885
H	-1.628622	-1.751225	-3.105462
H	-0.435856	-1.984166	-1.834909
C	-3.352785	-0.131246	-1.846891
H	-3.687771	0.881274	-1.640694
H	-3.475026	-0.295266	-2.919193
H	-4.022593	-0.817508	-1.336362

C	-2.067639	0.991005	0.801407
C	-2.014925	2.327832	0.003722
H	-2.528089	2.231003	-0.946100
H	-2.631272	3.022635	0.578679
C	-1.232807	1.262032	2.073152
H	-0.161534	1.213415	1.864870
H	-1.465431	2.265893	2.432278
H	-1.457708	0.578324	2.882250
C	-3.527135	0.799894	1.269247
H	-3.604575	0.013222	2.013819
H	-3.869234	1.719653	1.746611
H	-4.212960	0.572880	0.458442
C	-1.021071	0.599618	-2.354787
H	-1.282058	1.644545	-2.251015
H	0.041582	0.483958	-2.115906
H	-1.163621	0.338654	-3.404495
C	-1.641000	-1.579728	0.727920
C	-3.023831	-2.262345	0.809027
H	-2.852829	-3.325138	0.997761
H	-3.637814	-1.888008	1.624652
H	-3.601771	-2.190989	-0.106082
C	-0.978100	-1.710353	2.104688
H	-0.816708	-2.775580	2.284687
H	-0.004684	-1.218067	2.133657
H	-1.598204	-1.340140	2.918909
H	-1.035946	-2.222207	0.102508
C	-0.644992	2.986503	-0.193166
H	-0.707990	3.762048	-0.959585
H	-0.296926	3.459653	0.725127
H	0.124258	2.271917	-0.494519
Pd	0.946043	-0.080197	0.020224
P	3.137227	-0.091908	-0.002994
C	3.997529	1.220422	0.963351
H	3.712091	1.142830	2.012629
H	5.083518	1.128681	0.876910
H	3.688704	2.199661	0.596877
C	4.002670	-1.589431	0.632505
H	3.697456	-2.458507	0.049346
H	5.088228	-1.475732	0.569391
H	3.718221	-1.757945	1.671326
C	3.972465	0.101518	-1.634295
H	5.060416	0.091782	-1.526427
H	3.668966	-0.711190	-2.294559
H	3.664083	1.043555	-2.088268

60

35Pd SCF Done: -1196.34040233 A.U.

N	-1.255500	1.034310	0.223781
C	-2.001893	-0.228224	-0.356048
C	-1.638743	-1.437761	0.578263
H	-0.622359	-1.255785	0.944641
H	-2.305719	-1.390109	1.439509
C	-3.527227	-0.044275	-0.368045

H	-3.836662	0.745910	-1.048938
H	-4.003903	-0.964087	-0.702654
H	-3.918708	0.182126	0.623583
C	-1.487648	2.423406	-0.442178
C	-1.663821	2.385424	-1.968012
H	-2.595891	1.925968	-2.285328
H	-1.685446	3.417615	-2.319015
H	-0.826384	1.892292	-2.456466
C	-0.223486	3.283823	-0.233240
H	0.645998	2.779943	-0.665648
H	-0.357754	4.241822	-0.740064
H	-0.002556	3.492458	0.805187
C	-2.702791	3.183041	0.131579
H	-2.577614	3.455437	1.177360
H	-2.824453	4.112883	-0.425821
H	-3.627808	2.618562	0.033536
C	-1.523928	-0.548916	-1.776660
H	-1.902393	0.133557	-2.522889
H	-0.432346	-0.557102	-1.823262
H	-1.888495	-1.530489	-2.056586
C	-1.549053	1.122033	1.697104
C	-0.663561	2.003310	2.576244
H	-0.844380	1.716358	3.614984
H	0.393251	1.833035	2.358392
H	-0.872759	3.067729	2.496309
H	-1.428014	0.128674	2.096896
H	-2.595418	1.389498	1.864301
C	-1.641767	-2.942825	0.159487
C	-1.454589	-3.708759	1.488750
H	-2.278735	-3.513789	2.178942
H	-1.414428	-4.785284	1.308478
H	-0.524595	-3.415254	1.981727
C	-0.451614	-3.330695	-0.739692
H	-0.565774	-3.004631	-1.770370
H	0.474474	-2.896839	-0.358067
H	-0.339242	-4.417702	-0.754297
C	-2.961500	-3.421317	-0.462695
H	-3.807924	-3.191530	0.188642
H	-3.155697	-2.973785	-1.437310
H	-2.934899	-4.504866	-0.603390
Pd	1.039826	0.422700	0.034859
P	3.124317	-0.244767	-0.104472
C	4.205271	-0.012277	1.370381
H	5.216104	-0.384708	1.183542
H	4.252852	1.047783	1.620450
H	3.777185	-0.544790	2.219967
C	3.424711	-2.030854	-0.447180
H	2.939937	-2.307416	-1.383614
H	4.494275	-2.246392	-0.516102
H	2.988640	-2.631028	0.351719
C	4.181456	0.518600	-1.407555
H	4.226917	1.596532	-1.250968

H	5.194559	0.108125	-1.386537
H	3.739010	0.334004	-2.386747

63

36Pd SCF Done: -1235.65765411 A.U.

N	1.489500	-0.607043	0.033397
C	1.892579	0.509599	-0.987359
C	1.920239	1.975965	-0.389383
H	2.681034	2.014691	0.391972
H	2.362278	2.537877	-1.215009
C	3.321070	0.343033	-1.564392
H	3.454322	-0.610459	-2.064435
H	3.488908	1.117209	-2.314430
H	4.097779	0.448382	-0.809838
C	1.792222	-2.076226	-0.339220
C	1.387468	-2.401470	-1.789255
H	2.040364	-1.955511	-2.533021
H	1.451969	-3.481668	-1.920875
H	0.358482	-2.103504	-1.987794
C	0.923403	-3.016407	0.522844
H	-0.132820	-2.731034	0.468388
H	1.023961	-4.032744	0.139020
H	1.225891	-3.037292	1.562975
C	3.255790	-2.543818	-0.140461
H	3.487299	-2.702479	0.909002
H	3.379904	-3.506167	-0.640656
H	3.993895	-1.863850	-0.550187
C	0.942257	0.523360	-2.203115
H	1.191048	-0.232335	-2.938618
H	-0.097360	0.371813	-1.898803
H	1.016678	1.486619	-2.704424
C	1.869576	-0.207694	1.443694
C	1.009324	-0.789350	2.574572
H	1.033020	-0.082170	3.407295
H	-0.032991	-0.904232	2.262860
H	1.372927	-1.742158	2.954664
H	1.608595	0.832037	1.452391
C	0.736987	2.896480	0.078292
C	1.398641	4.265991	0.348430
H	1.860105	4.671090	-0.555652
H	0.654241	4.985973	0.695716
H	2.172072	4.187610	1.116529
C	0.028435	2.469833	1.372408
H	-0.467599	1.499971	1.258490
H	0.715802	2.420723	2.219984
H	-0.742573	3.203240	1.622199
C	-0.336974	3.108196	-1.000961
H	0.102777	3.411305	-1.953168
H	-0.929775	2.209367	-1.160432
H	-1.018560	3.901681	-0.683641
C	3.347788	-0.244920	1.877646
H	3.462066	0.438025	2.723363
H	3.666404	-1.227954	2.217345

H	4.031397	0.083365	1.100153
Pd	-0.999297	-0.532735	0.027948
P	-3.182566	-0.312368	-0.009935
C	-4.229886	-1.521600	0.904328
H	-3.949652	-1.518606	1.957905
H	-5.291443	-1.276437	0.812623
H	-4.056751	-2.522305	0.507720
C	-3.834861	1.272157	0.667622
H	-3.402393	2.104413	0.111935
H	-4.924881	1.317270	0.598166
H	-3.535398	1.369090	1.711353
C	-4.015689	-0.333545	-1.653414
H	-3.838889	-1.294484	-2.137001
H	-5.092550	-0.171906	-1.555556
H	-3.590155	0.448678	-2.282509

60

37Pd SCF Done: -1271.40626630 A.U.

N	0.865350	1.327082	-0.035242
C	0.763082	2.058579	-1.356286
C	-0.476114	2.951658	-1.453137
H	-0.377660	3.880995	-0.889974
H	-0.632441	3.221033	-2.499558
H	-1.364557	2.417777	-1.098487
H	0.609325	1.270512	-2.089403
C	2.004773	2.856836	-1.776730
H	2.919517	2.265433	-1.745457
H	1.871940	3.202965	-2.803500
H	2.143766	3.739160	-1.150658
C	0.956271	2.309855	1.118457
C	2.016290	0.350438	-0.050983
H	2.936445	0.941114	-0.044379
C	2.112972	-0.557307	-1.306130
C	2.090963	-0.579084	1.188656
H	2.052895	0.038851	-2.214856
C	1.021880	-1.636932	-1.324019
C	3.495493	-1.239649	-1.294991
H	1.997539	0.006162	2.098977
C	1.006949	-1.664369	1.171488
C	3.473995	-1.258917	1.197356
H	1.115286	-2.232912	-2.237423
H	0.026539	-1.174150	-1.334878
C	1.155972	-2.534377	-0.084174
H	4.287760	-0.484481	-1.309810
H	3.605819	-1.837603	-2.204631
C	3.636028	-2.131849	-0.055015
H	0.007916	-1.207972	1.187062
H	1.095918	-2.278137	2.073638
H	4.266607	-0.504776	1.236613
H	3.568331	-1.870816	2.099361
H	0.370524	-3.295345	-0.097948
C	2.537856	-3.209175	-0.078175
H	4.621124	-2.607296	-0.050305

H	2.633687	-3.860058	0.796759
H	2.653572	-3.840343	-0.965170
H	0.492037	3.216073	0.738621
C	2.366555	2.716616	1.576850
H	2.880633	1.920158	2.114426
H	3.002864	3.035475	0.754172
H	2.269951	3.560048	2.263189
C	0.107371	1.894722	2.322707
H	0.513005	1.031798	2.848394
H	0.053223	2.724717	3.031800
H	-0.909658	1.643175	2.003503
Pd	-1.352389	0.293317	0.048915
P	-3.340412	-0.633710	-0.013861
C	-4.401790	-0.524461	1.488956
H	-4.602804	0.523039	1.714726
H	-5.349298	-1.050538	1.344529
H	-3.873382	-0.961129	2.336747
C	-4.537911	-0.077281	-1.299411
H	-4.741884	0.985655	-1.167991
H	-4.100230	-0.221285	-2.287515
H	-5.476187	-0.635041	-1.235784
C	-3.368532	-2.453903	-0.304893
H	-4.390692	-2.841757	-0.315455
H	-2.889925	-2.673033	-1.259684
H	-2.801526	-2.952333	0.481726

82

39Pd SCF Done: -1659.8669346 A.U.

N	0.537135	0.579582	0.202374
C	0.052191	1.978514	-0.188770
H	0.952425	2.604292	-0.225018
C	-0.923303	2.683654	0.792782
C	-0.603975	2.073373	-1.599876
H	-0.545630	2.647955	1.810745
C	-2.332897	2.072792	0.752830
C	-1.005135	4.172203	0.398063
H	0.003169	1.555064	-2.341440
C	-2.021045	1.488162	-1.643222
C	-0.676170	3.561719	-1.994569
H	-2.963911	2.577031	1.491505
H	-2.299402	1.010830	1.019714
C	-2.928690	2.225134	-0.654248
H	-0.011505	4.628081	0.451138
H	-1.637153	4.696606	1.121129
C	-1.584325	4.317633	-1.013549
H	-1.997475	0.415600	-1.399859
H	-2.412086	1.576164	-2.662041
H	0.325116	4.004139	-1.998332
H	-1.068005	3.644550	-3.012587
H	-3.930944	1.787664	-0.676975
C	-2.998774	3.712365	-1.039089
H	-1.628764	5.375029	-1.289873
H	-3.434589	3.819068	-2.037502

H	-3.647596	4.254267	-0.343331
C	1.679352	0.250376	-0.746295
H	1.413792	0.858393	-1.599782
C	1.716363	-1.176761	-1.345449
C	3.141106	0.684611	-0.448323
H	0.682562	-1.527702	-1.467082
C	2.514432	-2.194959	-0.521377
C	2.392208	-1.047663	-2.727855
H	3.150324	1.657924	0.043441
C	3.949833	-0.338427	0.369663
C	3.830066	0.820342	-1.827838
H	2.515654	-3.153772	-1.049552
H	2.039629	-2.374978	0.439861
C	3.959714	-1.702595	-0.339247
H	1.820386	-0.366276	-3.365287
H	2.401345	-2.021020	-3.227227
C	3.834435	-0.536946	-2.554943
H	3.556723	-0.448106	1.377021
H	4.975432	0.028945	0.478448
H	3.314653	1.573100	-2.431680
H	4.858454	1.168323	-1.690857
H	4.520472	-2.421270	0.265362
C	4.626180	-1.553575	-1.716292
H	4.303594	-0.421784	-3.536423
H	5.661814	-1.219049	-1.598870
H	4.654394	-2.520853	-2.227603
C	0.734913	0.513013	1.693580
C	0.966024	-0.885216	2.273324
C	1.719234	1.509073	2.342682
H	-0.250528	0.779740	2.072077
C	0.743880	-0.866845	3.789498
H	1.974843	-1.238177	2.072655
H	0.271420	-1.587962	1.796661
C	1.488560	1.547502	3.858606
H	2.749596	1.211234	2.157904
H	1.604786	2.509898	1.925700
C	1.647479	0.157841	4.480621
H	0.920493	-1.864931	4.198602
H	-0.304914	-0.624549	3.995471
H	2.182922	2.252960	4.322345
H	0.478599	1.922373	4.062133
H	1.432785	0.193794	5.552079
H	2.691526	-0.160116	4.378951
Pd	-1.154643	-1.157018	-0.067609
P	-2.744851	-2.667061	-0.153241
C	-2.594709	-4.033838	-1.380810
C	-3.111522	-3.637158	1.370406
C	-4.431947	-2.045618	-0.560665
H	-4.734564	-1.311199	0.186154
H	-4.407888	-1.551578	-1.532353
H	-5.163062	-2.858143	-0.586104
H	-3.375422	-2.954316	2.178271

H	-2.220819	-4.190526	1.668849
H	-3.934513	-4.338319	1.207061
H	-1.687626	-4.603910	-1.178785
H	-2.516641	-3.612045	-2.383157
H	-3.458349	-4.702978	-1.337386

63

42Pd SCF Done: -1310.72149111 A.U.

N	0.822097	1.260955	0.135615
C	0.800351	2.286136	-1.042862
C	0.015501	3.562524	-0.677565
H	0.546859	4.211519	0.018160
H	-0.137741	4.134930	-1.592783
H	-0.969143	3.323580	-0.271869
C	2.198561	2.743237	-1.509216
H	2.792576	1.942718	-1.943748
H	2.073261	3.504158	-2.281112
H	2.773953	3.192304	-0.702134
C	1.999745	0.301284	0.034452
H	2.903260	0.916049	0.071726
C	2.101545	-0.542189	-1.267418
C	2.127952	-0.710390	1.206778
H	2.021907	0.093725	-2.140463
C	1.032948	-1.642208	-1.333392
C	3.496580	-1.196165	-1.324973
H	2.066578	-0.194529	2.156030
C	1.058789	-1.809827	1.148905
C	3.522636	-1.364561	1.147416
H	1.123334	-2.178378	-2.283558
H	0.027279	-1.203757	-1.300229
C	1.205218	-2.608280	-0.153577
H	4.274524	-0.425984	-1.305170
H	3.597711	-1.728796	-2.275404
C	3.678898	-2.162634	-0.150627
H	0.054548	-1.371667	1.196467
H	1.168819	-2.468506	2.016389
H	4.301306	-0.598066	1.216476
H	3.641949	-2.021134	2.014619
H	0.433698	-3.382223	-0.198378
C	2.598701	-3.256164	-0.212867
H	4.672132	-2.619069	-0.192491
H	2.724875	-3.953575	0.621488
H	2.705214	-3.834300	-1.136393
C	0.030051	1.696701	-2.242180
H	0.448838	0.778723	-2.634360
H	-1.004308	1.485186	-1.949852
H	0.018252	2.427107	-3.054160
C	0.800483	1.965522	1.488458
H	0.170269	2.825613	1.324876
C	2.141002	2.515200	1.998537
H	2.854287	1.734812	2.261162
H	2.616157	3.177628	1.277626
H	1.952666	3.099218	2.901613

C	0.054679	1.218668	2.605461
H	0.613577	0.412954	3.073518
H	-0.192861	1.942558	3.386010
H	-0.884134	0.803398	2.219867
Pd	-1.388181	0.221522	0.039113
P	-3.390467	-0.673474	-0.019871
C	-4.469074	-0.508035	1.465482
H	-5.424315	-1.020397	1.322547
H	-3.958849	-0.932525	2.330493
H	-4.653482	0.548299	1.662619
C	-3.444770	-2.500231	-0.263302
H	-4.473221	-2.871018	-0.276216
H	-2.959002	-2.752199	-1.206312
H	-2.895703	-2.987354	0.542836
C	-4.564043	-0.134151	-1.334820
H	-4.114936	-0.309096	-2.312836
H	-5.510646	-0.677515	-1.270145
H	-4.755381	0.934420	-1.233474

61

43Pd SCF Done: -1309.50019967 A.U.

N	0.886181	1.285499	0.146801
C	0.786682	2.305121	-1.017512
C	-0.133944	3.462816	-0.592176
H	0.266597	4.026844	0.247582
H	-0.242409	4.147718	-1.433676
H	-1.126052	3.091673	-0.331846
C	2.144234	2.897647	-1.428697
H	2.854391	2.138602	-1.752797
H	1.989454	3.577719	-2.267772
H	2.594522	3.473334	-0.622003
C	1.004236	1.992876	1.441905
C	-0.210400	1.954981	2.319134
C	2.119903	2.588509	1.857001
H	-0.469391	0.918098	2.552447
H	3.037020	2.602300	1.288521
C	2.048362	0.305471	0.067558
H	2.961540	0.898760	0.152902
C	2.217436	-0.530345	-1.222947
C	2.056126	-0.690477	1.260640
H	2.209293	0.114749	-2.095460
C	1.149360	-1.623120	-1.364833
C	3.610132	-1.193980	-1.164689
H	1.890726	-0.146848	2.189412
C	0.993938	-1.788924	1.115073
C	3.445349	-1.353211	1.319816
H	1.305332	-2.162644	-2.304321
H	0.147216	-1.180191	-1.404531
C	1.231600	-2.587666	-0.172083
H	4.388513	-0.427574	-1.094917
H	3.781574	-1.740134	-2.097030
C	3.696626	-2.150386	0.031219
H	-0.014323	-1.350302	1.094297

H	1.036742	-2.447680	1.988326
H	4.222220	-0.593485	1.451908
H	3.492944	-2.016045	2.188931
H	0.462294	-3.358479	-0.272939
C	2.622421	-3.241315	-0.119397
H	4.688736	-2.609604	0.067022
H	2.676384	-3.939455	0.721957
H	2.803195	-3.819304	-1.031431
C	0.110394	1.666198	-2.242892
H	0.707963	0.903997	-2.729541
H	-0.845693	1.213435	-1.952049
H	-0.090909	2.445775	-2.979944
H	2.146114	3.075259	2.822697
H	-0.036722	2.493215	3.251913
H	-1.080772	2.385086	1.821957
Pd	-1.351772	0.170135	0.054305
P	-3.356249	-0.717632	-0.016771
C	-4.576757	-0.237702	1.278087
H	-5.525530	-0.764342	1.143670
H	-4.170948	-0.472968	2.262257
H	-4.752083	0.837220	1.229120
C	-4.370622	-0.456415	-1.533087
H	-3.827748	-0.834606	-2.399666
H	-5.334332	-0.967654	-1.459660
H	-4.539668	0.611185	-1.675492
C	-3.438176	-2.554159	0.119772
H	-4.470216	-2.912141	0.073953
H	-2.865220	-3.001447	-0.692812
H	-2.990194	-2.865810	1.063598

18

NH3Pd SCF Done: -645.777999369 A.U.

N	3.145208	-0.000266	-0.000356
H	3.518727	0.917480	-0.222017
H	3.517746	-0.651127	-0.684872
H	3.519484	-0.267656	0.904851
Pd	0.958325	0.000309	0.000184
P	-1.244991	0.000006	0.000088
C	-2.092242	-1.627407	0.163521
H	-3.178835	-1.509346	0.151833
H	-1.791952	-2.276114	-0.659648
H	-1.791901	-2.100449	1.098620
C	-2.094095	0.954968	1.326286
H	-1.792864	2.001127	1.269069
H	-3.180409	0.886448	1.226789
H	-1.796479	0.566029	2.300286
C	-2.093644	0.671291	-1.490276
H	-3.180041	0.621103	-1.380814
H	-1.794862	1.708861	-1.640949
H	-1.793271	0.098090	-2.367621

27

NMe3Pd SCF Done: -763.739553540 A.U.

N	2.459064	-0.000830	0.000610

C	2.951687	1.233027	-0.640153
H	2.585322	1.285067	-1.663911
H	4.051269	1.257408	-0.652550
H	2.584863	2.100338	-0.094093
C	2.950123	-1.173145	-0.747848
H	2.583747	-2.085501	-0.280574
H	4.049694	-1.196798	-0.763747
H	2.582570	-1.133751	-1.771709
C	2.950749	-0.063172	1.389777
H	4.050331	-0.064364	1.417708
H	2.583851	0.797196	1.946693
H	2.584052	-0.970021	1.867349
Pd	0.211874	0.001759	-0.001062
P	-1.984775	-0.000193	0.000122
C	-2.835950	1.480134	-0.690251
H	-3.922200	1.369283	-0.638752
H	-2.537469	2.365066	-0.127907
H	-2.537249	1.618309	-1.729536
C	-2.831627	-0.143676	1.629470
H	-2.533772	0.688670	2.267378
H	-3.918174	-0.136206	1.510395
H	-2.529126	-1.071903	2.114557
C	-2.832119	-1.340870	-0.936418
H	-3.918623	-1.242540	-0.868143
H	-2.531872	-1.297419	-1.983500
H	-2.532437	-2.308861	-0.534383

36

NEt3Pd SCF Done: -881.735113201 A.U.

N	2.074441	0.001506	-0.000346
C	2.572923	1.294840	-0.546192
H	2.194229	1.380024	-1.561693
H	3.671210	1.256091	-0.608348
C	2.144374	2.533180	0.230438
H	2.634174	2.615131	1.200340
H	2.408548	3.420750	-0.347347
H	1.062878	2.533094	0.384945
C	2.576111	-1.116498	-0.847367
H	2.200800	-2.039731	-0.413062
H	3.674570	-1.147457	-0.783659
C	2.145320	-1.064264	-2.307496
H	2.633822	-0.265465	-2.864816
H	2.408517	-2.008670	-2.787294
H	1.063586	-0.930409	-2.381984
C	2.574372	-0.172254	1.392043
H	3.672566	-0.097569	1.389103
H	2.194800	0.663513	1.974486
C	2.147580	-1.465356	2.074774
H	2.641709	-2.344705	1.662559
H	2.407192	-1.408132	3.133393
H	1.066716	-1.602195	1.992672
Pd	-0.212339	-0.004050	0.001664
P	-2.409255	0.000243	-0.000089

C	-3.255417	1.615260	-0.265645
H	-4.342266	1.498933	-0.248217
H	-2.955788	2.313729	0.515913
H	-2.952937	2.028729	-1.227937
C	-3.265595	-0.573546	1.526970
H	-2.964050	0.051404	2.367800
H	-4.351600	-0.524407	1.412156
H	-2.972792	-1.601313	1.742342
C	-3.262169	-1.032299	-1.265460
H	-4.348514	-0.952279	-1.173290
H	-2.960662	-0.707055	-2.261358
H	-2.966376	-2.074263	-1.141872

25

PyPd SCF Done: -837.575700508 A.U.

N	1.904174	-0.000468	0.003181
C	2.597902	1.150340	0.001266
C	3.983683	1.194301	-0.002604
C	4.694773	0.001303	-0.004466
C	3.985243	-1.192542	-0.002287
C	2.599328	-1.150355	0.001562
H	5.776902	0.002010	-0.007494
H	2.013032	2.059702	0.002851
H	4.488346	2.150574	-0.004088
H	4.491120	-2.148173	-0.003515
H	2.015576	-2.060423	0.003369
Pd	-0.252631	-0.001132	0.002985
P	-2.462189	0.000331	-0.001298
C	-3.304231	1.602632	-0.337401
H	-2.999473	1.973710	-1.316066
H	-4.391010	1.488342	-0.317087
H	-3.005165	2.333434	0.414195
C	-3.304231	-1.089432	-1.223098
H	-3.003893	-2.123573	-1.053668
H	-4.391028	-1.010729	-1.137479
H	-3.000823	-0.805702	-2.230979
C	-3.310735	-0.510198	1.550640
H	-3.011166	0.153068	2.362228
H	-4.396995	-0.472436	1.435708
H	-3.011154	-1.525709	1.810242

44

1PdCl2 SCF Done: -1880.95347780 A.U.

N	-1.902487	0.208480	-0.052029
C	-2.308116	1.633532	-0.291713
C	-2.008125	2.235316	-1.658425
H	-1.815521	2.236957	0.463633
H	-3.386186	1.712345	-0.129069
H	-2.268762	3.294901	-1.614845
H	-0.953953	2.166639	-1.913356
H	-2.602172	1.787197	-2.454249
C	-2.547806	-0.656608	-1.139989
C	-3.035726	-2.046148	-0.716813
H	-3.433691	-0.101445	-1.463243

H	-3.455248	-2.523232	-1.604833
H	-2.217182	-2.665269	-0.360844
H	-3.820393	-2.025848	0.035369
C	-2.347023	-0.191688	1.342698
C	-1.501133	0.454093	2.439935
H	-2.155969	-1.259235	1.392702
H	-1.859025	0.098205	3.407977
H	-0.448909	0.183312	2.351382
H	-1.575358	1.541241	2.437578
C	-3.834275	0.063238	1.642982
H	-4.129315	-0.557367	2.490585
H	-4.016672	1.102470	1.919024
H	-4.489501	-0.181991	0.808793
C	-1.625558	-0.830565	-2.352726
H	-1.110102	0.083115	-2.631828
H	-0.876710	-1.594943	-2.150188
H	-2.221170	-1.155052	-3.208997
Pd	0.454363	0.000566	0.011055
P	2.714748	-0.164009	-0.004655
C	3.481298	0.544067	1.484015
H	3.130429	-0.003019	2.358654
H	4.567159	0.460643	1.415202
H	3.189005	1.587532	1.574064
C	3.458825	0.710745	-1.415938
H	4.545449	0.621544	-1.368912
H	3.099572	0.262622	-2.342000
H	3.165875	1.757028	-1.388081
C	3.433181	-1.830061	-0.115482
H	4.519867	-1.738621	-0.163480
H	3.145330	-2.415178	0.754296
H	3.063250	-2.332587	-1.006753
Cl	0.272591	-2.327658	0.307525
Cl	0.851654	2.334556	0.104572

33

2PdCl2 SCF Done: -1761.78490405 A.U.

N	1.816865	-0.368598	-0.001353
C	4.678937	0.291371	0.002013
C	3.972641	-0.226981	1.256428
C	2.485117	0.120090	1.230727
C	2.486686	0.125842	-1.230309
C	3.974263	-0.221016	-1.255750
H	1.964039	-0.311009	2.085663
H	4.087701	-1.314420	1.321305
H	4.419739	0.194901	2.159628
H	4.662461	1.386594	0.004612
H	5.728346	-0.010992	0.001954
H	1.966729	-0.301286	-2.087904
H	2.341141	1.206226	-1.256787
H	4.089582	-1.308101	-1.325709
H	4.422470	0.205265	-2.156336
H	2.339546	1.200378	1.262016
H	1.857847	-1.386320	-0.003572

Pd	-0.341358	-0.045842	-0.001382
P	-2.605064	0.142501	0.000345
C	-3.302800	1.820101	-0.001492
H	-4.392408	1.759035	-0.000050
H	-2.958822	2.358148	0.879512
H	-2.961094	2.355379	-0.885098
C	-3.362502	-0.669060	-1.441017
H	-3.048098	-1.710757	-1.463975
H	-4.450145	-0.607610	-1.376014
H	-3.024466	-0.175269	-2.351513
C	-3.358102	-0.664203	1.446707
H	-3.016608	-0.167947	2.354564
H	-4.445906	-0.602287	1.385259
H	-3.044290	-1.706049	1.471655
Cl	-0.575142	-2.397139	-0.001202
Cl	-0.008554	2.281295	0.001484

45

3PdCl2 SCF Done: -1919.10891763 A.U.

C	-4.472434	-0.163298	0.007513
C	-3.694973	-0.581468	-1.236202
C	-2.275309	0.018078	-1.302611
C	-2.270700	0.068103	1.301670
C	-3.690194	-0.534106	1.263235
H	-3.609576	-1.673862	-1.247557
H	-4.232083	-0.298229	-2.144560
H	-4.678377	0.909418	-0.012482
H	-5.445215	-0.660162	0.018799
H	-3.604315	-1.625211	1.315418
H	-4.224075	-0.216965	2.162229
N	-1.554284	-0.260440	0.003953
H	-1.478912	-1.276932	0.023513
C	-2.317897	1.570856	1.587826
H	-2.640786	1.715986	2.620213
H	-1.334925	2.018443	1.463931
H	-3.017916	2.105209	0.951964
C	-1.484033	-0.608622	2.430811
H	-1.323066	-1.668241	2.227554
H	-0.509563	-0.138885	2.571848
H	-2.042826	-0.518495	3.363612
C	-1.493554	-0.701584	-2.408446
H	-0.521839	-0.234480	-2.575357
H	-1.327497	-1.751533	-2.163459
H	-2.058920	-0.651123	-3.340291
C	-2.321863	1.509000	-1.645214
H	-3.022964	2.066771	-1.030965
H	-1.338799	1.960663	-1.536304
H	-2.642766	1.615471	-2.682893
Pd	0.706069	-0.060416	-0.006785
P	2.974644	0.006204	-0.001207
C	3.628479	0.841852	1.475672
H	3.229510	1.853059	1.516068
H	3.312279	0.297128	2.364904

H	4.718562	0.868790	1.434459
C	3.843279	-1.589878	-0.033112
H	3.560596	-2.143158	-0.926408
H	4.919312	-1.407829	-0.032280
H	3.563640	-2.178625	0.837700
C	3.667612	0.908304	-1.420443
H	3.265989	1.918715	-1.432644
H	4.755811	0.936657	-1.341739
H	3.384153	0.399990	-2.341616
Cl	0.816041	2.297677	-0.031889
Cl	0.678975	-2.423373	0.038636

36

4PdCl2 SCF Done: -1801.10364369 A.U.

N	1.969212	0.055753	-0.781987
C	3.473849	-0.204715	1.752927
C	2.822625	1.107216	1.315128
C	2.731201	1.196543	-0.201389
C	2.674040	-1.204685	-0.417187
C	2.770549	-1.389618	1.090867
H	2.244863	2.118131	-0.505956
H	1.817242	1.188739	1.735822
H	3.392025	1.965243	1.680044
H	4.529208	-0.199140	1.458844
H	3.447788	-0.302522	2.840469
H	2.142988	-2.034406	-0.874362
H	3.682393	-1.163966	-0.852562
H	1.763060	-1.505280	1.496574
H	3.304723	-2.321132	1.292325
H	3.740550	1.182626	-0.636152
C	1.938409	0.189097	-2.252553
H	1.396414	-0.651599	-2.682717
H	1.435344	1.116042	-2.521829
H	2.955416	0.202810	-2.662607
Pd	-0.259425	0.055361	-0.210086
P	-2.466184	-0.021556	0.304800
C	-3.465361	-0.771718	-1.016162
H	-4.510366	-0.820726	-0.705470
H	-3.382670	-0.162303	-1.915606
H	-3.088143	-1.770044	-1.225104
C	-2.784237	-1.018365	1.792905
H	-2.278168	-0.561984	2.643289
H	-3.857489	-1.055916	1.987162
H	-2.394253	-2.022099	1.642279
C	-3.305904	1.552198	0.651190
H	-3.254691	2.197490	-0.222566
H	-4.347246	1.345455	0.904293
H	-2.814932	2.056639	1.480586
Cl	-0.299017	2.405802	-0.209820
Cl	-0.401762	-2.297055	-0.383431

67

11PdCl2 SCF Done: -2419.69575358 A.U.

N	0.694036	0.587559	0.819594

C	0.747818	0.652315	2.356598
C	1.139320	-0.666246	3.019050
H	2.204638	-0.873269	2.922546
H	0.909240	-0.583493	4.082582
H	0.575699	-1.509756	2.629383
H	-0.276698	0.852700	2.647058
C	1.652319	1.764628	2.899309
H	1.334024	2.764169	2.609286
H	1.629387	1.722026	3.989180
H	2.686922	1.625800	2.583241
C	2.028728	0.192942	0.325762
C	2.393988	-1.149635	0.351429
C	2.995259	1.101714	-0.116878
C	3.658228	-1.585176	-0.021699
H	1.671865	-1.890259	0.654657
C	4.256938	0.683530	-0.511104
H	2.790551	2.158470	-0.172065
C	4.606400	-0.664052	-0.462594
H	3.876805	-2.641819	0.016978
H	4.986524	1.402921	-0.859462
O	5.868828	-0.978611	-0.865219
C	6.264992	-2.346182	-0.845543
H	7.292149	-2.365768	-1.201577
H	6.224441	-2.757875	0.166877
H	5.639796	-2.950988	-1.508191
C	0.305979	1.965857	0.282084
H	1.154762	2.592344	0.543612
C	-0.896609	2.708222	0.924759
C	0.162972	2.082767	-1.256152
H	-0.900335	2.584837	2.005484
C	-2.271061	2.307513	0.366920
C	-0.665223	4.204988	0.607769
H	0.915367	1.465871	-1.745013
C	-1.226265	1.695350	-1.793186
C	0.396875	3.572483	-1.591042
H	-3.037464	2.903619	0.872462
H	-2.488970	1.264637	0.588031
C	-2.317831	2.550838	-1.145962
H	0.286479	4.539092	1.032004
H	-1.451628	4.799293	1.081592
C	-0.684812	4.434758	-0.913257
H	-1.430045	0.642176	-1.613260
H	-1.225557	1.828720	-2.879505
H	1.392270	3.887936	-1.263610
H	0.358810	3.713917	-2.674809
H	-3.295997	2.252258	-1.534684
C	-2.066641	4.034128	-1.454704
H	-0.495288	5.490832	-1.125056
H	-2.111396	4.205654	-2.534758
H	-2.843047	4.654105	-0.995372
Pd	-0.927577	-1.047923	0.153897
P	-2.588071	-2.452279	-0.474641

C	-2.824890	-3.852297	0.660444
H	-3.666119	-4.457883	0.319019
H	-1.920781	-4.460571	0.667483
H	-3.012759	-3.477750	1.663203
C	-2.467591	-3.241276	-2.106480
H	-3.354024	-3.860577	-2.256247
H	-2.406611	-2.480705	-2.881035
H	-1.571146	-3.854836	-2.155935
C	-4.176711	-1.568896	-0.537212
H	-4.103167	-0.760125	-1.264067
H	-4.969087	-2.256367	-0.837800
H	-4.392569	-1.149221	0.442149
Cl	0.197325	-1.472719	-1.879137
Cl	-2.160230	-0.855817	2.172116

42

18PdCl2 SCF Done: -1879.76284893 A.U.

N	-1.937266	-0.019414	0.318123
C	-2.951982	0.038218	-2.502811
C	-2.406054	1.274966	-1.797766
C	-2.586980	1.184734	-0.288616
C	-2.517710	-1.220323	-0.359130
C	-2.339810	-1.207219	-1.871529
H	-2.173767	2.071696	0.174094
H	-1.344653	1.399343	-2.022578
H	-2.911159	2.180513	-2.143142
H	-4.041826	0.005996	-2.394437
H	-2.738458	0.075298	-3.573461
H	-2.045710	-2.106380	0.045234
H	-3.588628	-1.259981	-0.114844
H	-1.273779	-1.261127	-2.104019
H	-2.796519	-2.116363	-2.270668
H	-3.659131	1.141640	-0.051576
C	-2.291822	-0.065967	1.791883
H	-3.388587	-0.011554	1.832217
C	-1.732508	1.119315	2.577956
H	-2.021755	2.083382	2.168150
H	-2.111997	1.065538	3.599457
H	-0.643721	1.085287	2.613727
C	-1.862870	-1.364139	2.475837
H	-2.115929	-1.294332	3.534553
H	-2.366117	-2.243027	2.078871
H	-0.787999	-1.518406	2.388823
Pd	0.378495	0.042433	0.052378
P	2.636373	0.006846	-0.186669
C	3.148491	-0.816333	-1.726087
H	2.750497	-0.260545	-2.574787
H	4.237772	-0.840617	-1.787905
H	2.748338	-1.827088	-1.741947
C	3.515032	1.596862	-0.236849
H	3.334457	2.148029	0.683189
H	4.582569	1.400746	-0.352376
H	3.152925	2.192830	-1.071394

C	3.459642	-0.894771	1.161859
H	3.070860	-1.909124	1.205510
H	4.537207	-0.909951	0.989915
H	3.251535	-0.390915	2.105414
Cl	0.546765	-2.317176	-0.021907
Cl	0.414853	2.394139	0.115982

45

20PdCl2 SCF Done: -1919.08894473 A.U.

N	-1.856046	-0.050464	-0.017903
C	-2.388261	2.887092	-0.267189
C	-1.942408	2.239524	1.039230
C	-2.386841	0.782967	1.105290
C	-2.317453	0.577111	-1.294315
C	-1.900356	2.037559	-1.435262
H	-2.071183	0.341809	2.043732
H	-0.857406	2.299543	1.137976
H	-2.368714	2.758520	1.901640
H	-3.481964	2.951283	-0.289940
H	-2.006968	3.907856	-0.345608
H	-1.908678	0.014445	-2.123734
H	-3.410408	0.516497	-1.345113
H	-0.812117	2.092861	-1.507467
H	-2.298373	2.406893	-2.383927
H	-3.480144	0.766338	1.069637
C	-2.358244	-1.507430	0.098620
C	-1.645108	-2.211071	1.258958
H	-1.756989	-1.681868	2.202136
H	-2.070790	-3.208654	1.378586
H	-0.578483	-2.316327	1.063489
C	-2.045064	-2.285541	-1.186907
H	-2.240382	-3.342772	-1.003802
H	-2.674169	-1.979575	-2.022118
H	-1.003194	-2.176820	-1.480325
C	-3.877972	-1.586071	0.348502
H	-4.450440	-1.011289	-0.379233
H	-4.187357	-2.627667	0.255751
H	-4.151525	-1.253210	1.348784
Pd	0.521141	-0.030573	0.044525
P	2.788882	-0.032674	0.009893
C	3.668497	0.085923	1.595969
H	4.742135	0.070480	1.399265
H	3.395070	1.007975	2.103447
H	3.394051	-0.751362	2.233497
C	3.470908	-1.541933	-0.742597
H	3.080951	-1.649551	-1.751524
H	4.560189	-1.479190	-0.763802
H	3.169862	-2.404011	-0.147985
C	3.458045	1.354036	-0.958194
H	3.152197	2.291603	-0.494764
H	4.547645	1.296886	-0.975744
H	3.062888	1.309384	-1.970043
Cl	0.560580	0.184352	2.389878

Cl	0.702740	-0.236903	-2.307328

47

21PdCl2 SCF Done: -1920.27271758 A.U.

N	1.840709	0.014128	-0.070789
C	2.147663	0.582189	1.300088
C	1.544957	-0.262890	2.421836
H	2.040648	-1.229316	2.520290
H	1.675175	0.271553	3.364636
H	0.478546	-0.439612	2.277759
C	3.624060	0.891691	1.661170
H	4.271415	1.012531	0.798236
H	3.652732	1.822681	2.230278
H	4.057474	0.115365	2.290142
C	2.292307	0.916876	-1.218268
C	1.301151	0.922883	-2.393522
H	0.460031	1.578971	-2.172889
H	1.808766	1.311421	-3.279600
H	0.910340	-0.057310	-2.640024
C	2.564499	2.387418	-0.871418
H	2.851852	2.887897	-1.798104
H	1.670167	2.877164	-0.496149
H	3.373336	2.538140	-0.164457
C	2.370759	-1.397355	-0.256100
C	3.877300	-1.593390	-0.015796
H	4.136488	-2.613042	-0.304783
H	4.489370	-0.921046	-0.618533
H	4.157550	-1.474254	1.025429
C	2.030318	-2.011289	-1.616105
H	2.268398	-3.074852	-1.570954
H	0.975810	-1.928219	-1.856483
H	2.623174	-1.577473	-2.422855
H	1.841255	-1.986087	0.486648
H	3.235647	0.499711	-1.577991
H	1.614191	1.527380	1.301378
Pd	-0.622714	-0.074917	0.036073
P	-2.881545	0.006256	-0.002780
C	-3.787936	-1.552403	-0.227776
H	-4.853892	-1.325226	-0.289297
H	-3.459362	-2.041607	-1.142208
H	-3.594360	-2.218509	0.608967
C	-3.484974	1.045490	-1.368351
H	-4.575774	1.070325	-1.350664
H	-3.082903	2.049813	-1.264813
H	-3.148567	0.620654	-2.313752
C	-3.582356	0.709626	1.519226
H	-3.166373	1.701391	1.679401
H	-4.668797	0.765657	1.432233
H	-3.318262	0.069243	2.360299
Cl	-0.728164	-2.419091	0.213917
Cl	-0.749925	2.277153	0.265836

41

22PdCl2 SCF Done: -1841.65129599 A.U.

N	-1.825434	-0.347194	0.261826
C	-2.655954	-0.439274	-1.008180
C	-2.013600	-1.541279	-1.861869
H	-2.012922	-2.500723	-1.341009
H	-2.571483	-1.657240	-2.791526
H	-0.981182	-1.292817	-2.110005
C	-4.102859	-0.845484	-0.690007
H	-4.643768	-0.062783	-0.158607
H	-4.634642	-1.036520	-1.622954
H	-4.135710	-1.758739	-0.091894
C	-2.457195	0.240895	1.493266
C	-2.780283	1.724985	1.384560
H	-3.098386	2.087946	2.363561
H	-1.903889	2.288330	1.069497
H	-3.591021	1.922841	0.686118
C	-1.547797	-0.028186	2.691855
H	-2.076861	0.228603	3.610909
H	-1.252927	-1.077027	2.746792
H	-0.639955	0.575941	2.641979
H	-3.389435	-0.302646	1.671230
C	-2.640442	0.863888	-1.809304
H	-3.084653	1.694166	-1.267406
H	-1.624808	1.145420	-2.075684
H	-3.214847	0.717325	-2.726015
H	-1.665295	-1.320173	0.516263
Pd	0.401225	0.009562	0.021341
P	2.663336	0.068490	-0.142150
C	3.460361	-0.362786	1.434902
H	4.544882	-0.373934	1.314437
H	3.186497	0.375958	2.187680
H	3.108391	-1.341815	1.752905
C	3.288562	-1.136733	-1.352957
H	2.906713	-0.881705	-2.341188
H	4.379585	-1.115303	-1.368811
H	2.936457	-2.129302	-1.081142
C	3.447503	1.632210	-0.630298
H	4.528573	1.488018	-0.670557
H	3.076027	1.939320	-1.605763
H	3.199996	2.408182	0.090448
Cl	0.268421	2.299390	-0.482385
Cl	0.616081	-2.282187	0.600426

38

23PdCl2 SCF Done: -1802.32638831 A.U.

N	-1.931813	0.379666	-0.104412
C	-2.594622	0.584026	1.225853
C	-1.828074	1.619985	2.042655
H	-1.644658	2.528740	1.468933
H	-2.409057	1.880574	2.928785
H	-0.862549	1.230053	2.369990
C	-2.653355	-0.526387	-1.053149
C	-1.969111	-0.468337	-2.416102
H	-2.474233	-1.137796	-3.113145

H	-2.007303	0.543157	-2.829206
H	-0.924343	-0.771583	-2.348956
C	-4.140793	-0.192496	-1.189383
H	-4.587207	-0.839152	-1.946019
H	-4.693011	-0.344156	-0.262900
H	-4.279940	0.844007	-1.508268
H	-2.541341	-1.533671	-0.654129
C	-2.756168	-0.716896	2.000269
H	-3.359240	-1.448707	1.463259
H	-1.786186	-1.166777	2.206251
H	-3.255419	-0.505219	2.947177
H	-1.894517	1.298962	-0.542929
H	-3.588882	0.997095	1.030148
Pd	0.276546	0.014841	-0.033367
P	2.537562	-0.158170	0.019867
C	3.263345	0.969167	1.249449
H	2.909076	0.693895	2.242511
H	4.352299	0.903879	1.220487
H	2.942845	1.984974	1.027124
C	3.304747	0.298108	-1.564757
H	4.391897	0.261320	-1.477357
H	2.978463	-0.399887	-2.335219
H	2.984356	1.301968	-1.835784
C	3.250486	-1.781706	0.415014
H	2.928764	-2.511301	-0.325136
H	4.339053	-1.704642	0.415570
H	2.899581	-2.108052	1.391876
Cl	0.539945	2.298849	-0.604002
Cl	-0.006949	-2.279923	0.379039

38

25PdCl2 SCF Done: -2261.90040185 A.U.

N	1.903836	-0.159625	-0.004468
C	2.402895	-0.372663	1.415526
C	2.270631	0.902961	2.246898
H	1.244113	1.264879	2.248931
H	2.555278	0.661265	3.271626
H	2.918630	1.707452	1.905564
C	1.659977	-1.513111	2.103561
H	1.623891	-2.411834	1.493973
H	2.174315	-1.746956	3.036953
H	0.635477	-1.224729	2.342194
C	2.531057	1.036685	-0.687900
C	4.058106	1.070713	-0.658051
H	4.388462	1.999400	-1.125271
H	4.464788	1.050163	0.351600
H	4.484943	0.240802	-1.220947
H	3.458787	-0.641561	1.341390
Cl	2.417317	-1.611959	-0.932579
C	2.007864	1.196750	-2.110234
H	2.429863	0.448662	-2.780572
H	0.921232	1.128515	-2.143136
H	2.293062	2.183600	-2.477173

H	2.139261	1.866848	-0.104840
Pd	-0.499351	0.025991	-0.034218
P	-2.752505	0.130743	-0.046186
C	-3.512863	1.769037	0.141681
H	-3.185105	2.424313	-0.661849
H	-4.597356	1.649193	0.108771
H	-3.214688	2.209403	1.090012
C	-3.490350	-0.848987	1.294336
H	-4.577977	-0.778424	1.238450
H	-3.173676	-1.884422	1.199144
H	-3.150583	-0.455169	2.251811
C	-3.460347	-0.502534	-1.595137
H	-4.549280	-0.451385	-1.542984
H	-3.108221	0.110096	-2.424472
H	-3.137468	-1.529394	-1.745380
Cl	-0.363646	2.351144	0.300135
Cl	-0.813008	-2.285843	-0.324013

62

35PdCl2 SCF Done: -2116.89794651 A.U.

N	-1.459352	0.986858	0.317758
C	-2.235775	-0.195836	-0.407477
C	-1.935876	-1.513896	0.383831
H	-0.927549	-1.439054	0.781284
H	-2.605593	-1.529453	1.244419
C	-3.759657	0.021996	-0.409835
H	-4.059304	0.864905	-1.026450
H	-4.241274	-0.862573	-0.820801
H	-4.157559	0.165001	0.593776
C	-1.771668	2.451452	-0.140961
C	-1.998548	2.639912	-1.650799
H	-2.931735	2.209568	-2.004916
H	-2.058948	3.714729	-1.830423
H	-1.168671	2.259472	-2.234524
C	-0.553327	3.344732	0.180405
H	0.255845	3.146441	-0.516646
H	-0.847152	4.390303	0.065212
H	-0.171278	3.221208	1.184211
C	-3.020558	3.052716	0.552019
H	-2.903421	3.188167	1.622999
H	-3.194741	4.041446	0.125925
H	-3.917512	2.465002	0.379748
C	-1.767057	-0.366206	-1.852691
H	-1.985777	0.484337	-2.479217
H	-0.701386	-0.558473	-1.911253
H	-2.292040	-1.213026	-2.285393
C	-1.741749	0.870336	1.795148
C	-1.028814	1.773477	2.800689
H	-1.242327	1.362136	3.790380
H	0.050374	1.751573	2.672401
H	-1.366049	2.806559	2.801253
H	-1.462454	-0.129056	2.083819
H	-2.817368	0.955084	1.964782

C	-2.002552	-2.959416	-0.207997
C	-1.846128	-3.879245	1.024207
H	-2.667627	-3.737825	1.730318
H	-1.839732	-4.928711	0.721200
H	-0.911078	-3.672907	1.550062
C	-0.827264	-3.283736	-1.150698
H	-0.925004	-2.824643	-2.130970
H	0.117697	-2.949939	-0.716160
H	-0.763281	-4.364050	-1.302886
C	-3.333774	-3.319059	-0.882437
H	-4.176284	-3.133894	-0.212263
H	-3.504939	-2.761715	-1.803265
H	-3.343522	-4.380966	-1.141273
Pd	1.022798	0.348580	0.038873
P	3.156806	-0.385448	-0.124622
C	4.223254	0.263836	1.197387
H	5.232478	-0.133181	1.075214
H	4.251876	1.350788	1.125711
H	3.820257	-0.021660	2.165096
C	3.254838	-2.199068	-0.030436
H	2.702101	-2.626291	-0.866663
H	4.299387	-2.508973	-0.092523
H	2.814311	-2.540098	0.902352
C	4.085501	0.002759	-1.637470
H	4.158519	1.079931	-1.764820
H	5.082625	-0.431475	-1.541972
H	3.577988	-0.414668	-2.503570
Cl	1.119524	-0.715653	2.158512
Cl	1.244580	1.322505	-2.094666

77

38PdCl2 SCF Done: -2463.63539802 A.U.

N	0.756256	0.284105	0.710886
C	0.788006	0.176600	2.227771
C	0.826821	-1.249329	2.767215
H	1.841507	-1.585653	2.960506
H	0.292535	-1.265300	3.717746
H	0.333252	-1.957669	2.105222
H	-0.185415	0.549641	2.511572
C	1.797738	1.055708	2.991277
H	1.522967	2.109288	2.982975
H	1.790595	0.735436	4.034862
H	2.819044	0.971311	2.632237
C	0.779563	1.794911	0.398151
H	1.664622	2.146571	0.923640
C	-0.404453	2.618512	0.977454
C	1.000771	2.291378	-1.058488
H	-0.673239	2.262389	1.968651
C	-1.659989	2.593520	0.091025
C	0.075883	4.080840	1.092922
H	1.783271	1.721949	-1.550584
C	-0.260530	2.279321	-1.933598
C	1.495018	3.752482	-0.944530

H	-2.444867	3.181102	0.577343
H	-2.046629	1.576614	0.002685
C	-1.348037	3.154758	-1.299733
H	0.951021	4.143085	1.746705
H	-0.711143	4.684287	1.554627
C	0.413485	4.632674	-0.300351
H	-0.624275	1.265368	-2.071689
H	0.000185	2.661765	-2.926047
H	2.418666	3.794973	-0.359168
H	1.733330	4.125669	-1.944842
H	-2.250097	3.125454	-1.918050
C	-0.849891	4.603075	-1.176238
H	0.779360	5.659639	-0.211314
H	-0.628584	5.010695	-2.167744
H	-1.627434	5.233380	-0.732975
C	1.852189	-0.389999	-0.121145
H	1.520103	-0.082810	-1.104248
C	1.932210	-1.941410	-0.291490
C	3.319330	0.118507	0.012541
H	0.930235	-2.364499	-0.286917
C	2.836015	-2.726009	0.677769
C	2.558951	-2.141621	-1.699497
H	3.337396	1.179488	0.261181
C	4.166165	-0.658149	1.041193
C	3.969725	-0.075225	-1.376410
H	2.862423	-3.767271	0.340366
H	2.444844	-2.748407	1.687033
C	4.254609	-2.145271	0.672240
H	1.936507	-1.665451	-2.456516
H	2.585641	-3.210374	-1.932172
C	3.988699	-1.569129	-1.740084
H	3.755513	-0.567631	2.045254
H	5.167872	-0.217836	1.071797
H	3.422745	0.491710	-2.134183
H	4.991565	0.316343	-1.362466
H	4.869818	-2.666292	1.411595
C	4.869180	-2.309265	-0.723668
H	4.398754	-1.692774	-2.746713
H	5.887148	-1.906786	-0.742109
H	4.935288	-3.371019	-0.980765
Pd	-1.418737	-0.744248	0.085766
P	-3.453212	-1.637088	-0.370213
C	-3.897421	-2.989286	0.762447
H	-3.879886	-2.628328	1.787013
H	-4.891282	-3.362433	0.509424
H	-3.171042	-3.794224	0.652440
C	-4.775016	-0.394517	-0.240403
H	-4.588478	0.394730	-0.968415
H	-5.738391	-0.861626	-0.451300
H	-4.773129	0.032854	0.758738
C	-3.723996	-2.368298	-2.012206
H	-4.745754	-2.750821	-2.051187

H	-3.577569	-1.615795	-2.782912
H	-3.017068	-3.177316	-2.180479
Cl	-2.398987	-0.311501	2.211353
Cl	-0.757824	-1.246094	-2.112180

84

39PdCl2 SCF Done: -2580.41989982 A.U.

N	0.717571	0.298020	0.312127
C	0.639294	1.811651	0.007292
H	1.613601	2.175936	0.321919
C	-0.393313	2.621771	0.841149
C	0.526570	2.321850	-1.459047
H	-0.438753	2.257020	1.863523
C	-1.813021	2.604129	0.251562
C	0.102935	4.083487	0.868544
H	1.176478	1.756487	-2.121270
C	-0.897057	2.332268	-2.035954
C	1.039799	3.781884	-1.437879
H	-2.472120	3.179537	0.909188
H	-2.205803	1.586573	0.232504
C	-1.814461	3.194022	-1.162580
H	1.098515	4.139406	1.318867
H	-0.564761	4.680011	1.497103
C	0.130846	4.653891	-0.557088
H	-1.289849	1.324142	-2.117392
H	-0.854829	2.736727	-3.052802
H	2.070441	3.816212	-1.071851
H	1.050889	4.169969	-2.460531
H	-2.830911	3.175097	-1.566931
C	-1.296027	4.640006	-1.129353
H	0.512789	5.678669	-0.536814
H	-1.300438	5.063304	-2.138782
H	-1.951755	5.263739	-0.513463
C	1.724146	-0.316915	-0.669889
H	1.276797	0.028946	-1.590521
C	1.808492	-1.855315	-0.942482
C	3.186042	0.220236	-0.681843
H	0.822037	-2.300373	-0.837961
C	2.842447	-2.683738	-0.157644
C	2.255166	-1.947764	-2.429622
H	3.216426	1.262588	-0.364956
C	4.169740	-0.598917	0.178372
C	3.659959	0.132632	-2.150581
H	2.851344	-3.692192	-0.583918
H	2.577284	-2.808758	0.885605
C	4.236468	-2.058617	-0.293373
H	1.533843	-1.440051	-3.069092
H	2.271780	-2.997790	-2.736305
C	3.657933	-1.335435	-2.610599
H	3.893159	-0.575372	1.230412
H	5.160280	-0.138459	0.107182
H	3.012775	0.734360	-2.793766
H	4.669320	0.546497	-2.238343

H	4.950578	-2.607876	0.327022
C	4.673756	-2.114911	-1.763270
H	3.939442	-1.385313	-3.666552
H	5.673140	-1.683604	-1.880633
H	4.727989	-3.155541	-2.097753
C	0.967732	0.174058	1.808340
C	1.164865	-1.257890	2.291486
C	2.035004	1.077114	2.467795
H	0.024851	0.488838	2.238463
C	1.005720	-1.349330	3.811618
H	2.169934	-1.587037	2.049627
H	0.464780	-1.927736	1.791576
C	1.908671	0.990817	3.996580
H	3.042026	0.788278	2.171019
H	1.915025	2.120248	2.185994
C	2.018006	-0.443274	4.513332
H	1.137985	-2.386744	4.130600
H	-0.010932	-1.057060	4.087846
H	2.672070	1.622929	4.457860
H	0.938542	1.405502	4.293416
H	1.871698	-0.468246	5.596556
H	3.030360	-0.818370	4.322336
Pd	-1.471842	-0.781582	-0.061479
Cl	-1.090646	-1.165138	-2.350703
Cl	-2.175880	-0.499147	2.192320
P	-3.538833	-1.683875	-0.307300
C	-4.018688	-2.308775	-1.945470
C	-3.821265	-3.115078	0.779339
C	-4.845730	-0.477376	0.074005
H	-4.728671	-0.126305	1.095646
H	-4.753547	0.367755	-0.607808
H	-5.823279	-0.944088	-0.057840
H	-3.673938	-2.821266	1.814937
H	-3.108507	-3.898623	0.523165
H	-4.836207	-3.488156	0.632032
H	-3.337558	-3.096187	-2.259026
H	-3.977811	-1.505242	-2.676614
H	-5.035534	-2.699676	-1.874761

69

40PdCl2 SCF Done: -2308.74858042 A.U.

N	1.035092	-0.447967	0.727518
C	1.038917	-0.640070	2.240849
C	0.178307	-1.801289	2.753743
H	0.725063	-2.741006	2.787606
H	-0.132795	-1.563475	3.771966
H	-0.724754	-1.943268	2.166262
H	0.550787	0.257628	2.597509
C	2.403141	-0.710882	2.946873
H	3.096197	0.074103	2.650890
H	2.222478	-0.592758	4.017365
H	2.891879	-1.673057	2.805984
C	1.746596	0.879387	0.418957

H	2.695865	0.787712	0.942392
C	1.103551	2.176277	0.983598
C	2.150151	1.180138	-1.053660
H	0.708003	2.005488	1.982226
C	-0.014483	2.758752	0.102506
C	2.236291	3.223235	1.068032
H	2.540055	0.292374	-1.543271
C	1.020320	1.764073	-1.915295
C	3.291432	2.219866	-0.978543
H	-0.401108	3.663253	0.582307
H	-0.851994	2.065692	0.033208
C	0.511211	3.072733	-1.300008
H	3.038679	2.862230	1.718461
H	1.847782	4.141240	1.518505
C	2.782115	3.520690	-0.338597
H	0.205355	1.050240	-2.017404
H	1.410321	1.945201	-2.922201
H	4.132630	1.817213	-0.405850
H	3.660216	2.421499	-1.988482
H	-0.299337	3.476846	-1.913797
C	1.653745	4.095915	-1.210222
H	3.601697	4.241686	-0.269377
H	2.033840	4.327166	-2.210349
H	1.286331	5.032359	-0.778671
C	1.698827	-1.553108	-0.071894
C	3.236753	-1.682147	0.021558
C	1.105099	-2.956118	0.107666
H	1.469967	-1.280719	-1.097893
C	3.740748	-2.563851	-1.128394
H	3.523021	-2.145690	0.965529
H	3.738154	-0.717052	-0.016743
C	1.576802	-3.863325	-1.034943
H	1.434495	-3.392004	1.051197
H	0.018491	-2.917547	0.121106
C	3.103406	-3.954346	-1.087111
H	4.829937	-2.643548	-1.080589
H	3.503042	-2.080900	-2.083057
H	1.143526	-4.859623	-0.912947
H	1.195444	-3.465523	-1.979880
H	3.421592	-4.544882	-1.950441
H	3.461777	-4.481445	-0.195224
Pd	-1.300811	-0.331512	0.102203
P	-3.494852	-0.091055	-0.423880
C	-4.604869	-0.939097	0.740711
H	-5.641790	-0.756743	0.453196
H	-4.403424	-2.009525	0.706581
H	-4.422671	-0.572330	1.747270
C	-3.986845	1.660356	-0.416770
H	-3.396216	2.196308	-1.159428
H	-5.045793	1.744748	-0.666481
H	-3.794912	2.084550	0.565424
C	-4.060505	-0.681912	-2.046671

H	-5.130180	-0.481064	-2.130280
H	-3.520647	-0.167535	-2.837966
H	-3.873424	-1.749094	-2.138907
Cl	-1.954317	0.672462	2.167612
Cl	-0.939646	-1.330654	-1.999130

20

NH3PdCl2 SCF Done: -1566.35089704 A.U.

N	-2.846957	0.253904	-0.007569
H	-3.121681	1.092957	0.493038
H	-3.289685	-0.553477	0.419422
H	-3.189220	0.331932	-0.960236
Pd	-0.691022	0.030020	-0.004246
P	1.568662	-0.119930	-0.002193
C	2.280423	-1.790655	-0.024622
H	3.369366	-1.719734	-0.020890
H	1.940028	-2.343046	0.848870
H	1.944773	-2.317642	-0.915620
C	2.315120	0.718576	-1.432739
H	1.982816	1.754762	-1.446291
H	3.403369	0.674742	-1.364650
H	1.988153	0.227795	-2.348904
C	2.304444	0.676209	1.457567
H	3.393070	0.627453	1.399998
H	1.977938	1.713772	1.494166
H	1.964479	0.164104	2.357192
Cl	-0.494818	2.377100	0.009677
Cl	-1.009742	-2.296965	0.009373

29

NMe3PdCl2 SCF Done: -1684.31190788 A.U.

N	2.446985	0.034848	-0.001652
C	2.875820	0.789424	1.196559
H	2.429849	1.779363	1.186113
H	3.968201	0.881967	1.216179
H	2.549598	0.260352	2.090647
C	2.866476	0.754207	-1.224552
H	2.537274	0.197149	-2.100405
H	3.958404	0.850155	-1.253570
H	2.416805	1.742637	-1.241104
C	3.104627	-1.287760	0.016172
H	4.193353	-1.160694	0.013616
H	2.805325	-1.836119	0.904859
H	2.803985	-1.861232	-0.855870
Pd	0.169979	-0.058798	-0.002114
P	-2.097603	0.002135	-0.001617
C	-2.749784	0.840417	1.474744
H	-3.839552	0.873223	1.428860
H	-2.440602	0.292090	2.364148
H	-2.345293	1.849137	1.518693
C	-2.991950	-1.578857	-0.043342
H	-2.729892	-2.174548	0.827971
H	-4.063916	-1.373802	-0.048992
H	-2.714606	-2.135545	-0.935842

C	-2.771004	0.918038	-1.421714
H	-3.859560	0.953868	-1.350867
H	-2.361776	1.925406	-1.425402
H	-2.484328	0.412345	-2.343288
Cl	0.142126	-2.405147	0.004767
Cl	0.074799	2.307511	0.004063

38

NEt3PdCl2 SCF Done: -1802.29796307 A.U.

N	2.136826	0.092431	-0.004762
C	2.528010	1.147805	0.976702
H	2.065503	2.076497	0.662947
H	3.617095	1.278250	0.920003
C	2.114849	0.860307	2.412553
H	2.631343	0.000848	2.838412
H	2.354278	1.730147	3.026551
H	1.039649	0.688968	2.480464
C	2.640699	0.465606	-1.363885
H	2.328168	-0.323447	-2.043179
H	3.738241	0.448624	-1.328898
C	2.187456	1.797807	-1.940269
H	2.517701	2.651013	-1.349678
H	2.627938	1.894304	-2.934914
H	1.105775	1.851962	-2.035324
C	2.815595	-1.185174	0.414600
H	3.805166	-0.916047	0.803991
H	2.239843	-1.599488	1.236202
C	2.999491	-2.252621	-0.657342
H	3.778796	-1.992653	-1.373258
H	3.303782	-3.175953	-0.160754
H	2.075693	-2.457917	-1.191119
Pd	-0.202310	-0.062883	0.006159
P	-2.470468	-0.087186	-0.083271
C	-3.235412	0.630465	1.401952
H	-4.321472	0.625510	1.295424
H	-2.953872	0.033982	2.269296
H	-2.874722	1.647505	1.534851
C	-3.304537	-1.692234	-0.265074
H	-3.087012	-2.323016	0.593124
H	-4.379323	-1.515841	-0.338806
H	-2.948731	-2.195163	-1.161708
C	-3.106585	0.874621	-1.490982
H	-4.197397	0.844990	-1.488602
H	-2.757330	1.901050	-1.415874
H	-2.735683	0.436913	-2.417438
Cl	-0.204029	-2.404986	0.172728
Cl	-0.466873	2.291036	0.159498

27

PyPdCl2 SCF Done: -1758.14783499 A.U.

N	1.969114	-0.002291	0.002310
C	2.629150	-0.944548	0.689954
C	4.013181	-1.008172	0.718229
C	4.744202	-0.072934	-0.002013

C	4.059268	0.898214	-0.719988
C	2.673780	0.905211	-0.687412
H	5.825935	-0.100396	-0.003699
H	2.022944	-1.666961	1.216329
H	4.499805	-1.782882	1.294067
H	4.582917	1.647204	-1.297366
H	2.102619	1.657420	-1.211119
Pd	-0.207974	0.041487	0.007681
P	-2.474271	-0.003960	0.001097
C	-3.145222	-1.103804	1.284189
H	-2.854598	-0.729853	2.265442
H	-4.233706	-1.133443	1.213920
H	-2.733240	-2.101045	1.146468
C	-3.339223	1.571568	0.262134
H	-3.049221	2.281237	-0.509518
H	-4.415945	1.397358	0.225834
H	-3.061870	1.984455	1.229872
C	-3.134885	-0.630839	-1.572757
H	-2.721047	-1.619416	-1.760598
H	-4.223943	-0.681955	-1.524924
H	-2.837848	0.039400	-2.378847
Cl	-0.148881	2.371683	0.304603
Cl	-0.263310	-2.295934	-0.312004

15

PMe3Au SCF Done: -1057.32503158 A.U.

Au	0.553526	-0.000156	-0.000281
P	-1.713755	0.000223	0.000001
C	-2.449548	1.564510	-0.568427
H	-3.538406	1.499005	-0.544460
H	-2.118651	2.375075	0.079676
H	-2.118445	1.771384	-1.585325
C	-2.450599	-1.274731	-1.069378
H	-2.119739	-2.258562	-0.739018
H	-3.539568	-1.221066	-1.024049
H	-2.120210	-1.120205	-2.095790
C	-2.449538	-0.289165	1.638996
H	-3.538396	-0.276400	1.570138
H	-2.119201	-1.255935	2.016889
H	-2.118161	0.488014	2.326616
Cl	2.906274	0.000235	0.000612

14

PMe3Pd SCF Done: -589.154108576 A.U.

Pd	1.311172	-0.000014	0.000065
P	-0.858343	-0.000050	0.000082
C	-1.702419	1.185985	-1.126985
H	-2.788837	1.097614	-1.042374
H	-1.402711	2.202931	-0.874225
H	-1.403358	0.984893	-2.155602
C	-1.702762	-1.568876	-0.463691
H	-1.403142	-2.359675	0.223894
H	-2.789122	-1.451353	-0.428369
H	-1.403962	-1.857710	-1.471265

C -1.703166 0.383018 1.590282
H -2.789501 0.353656 1.470834
H -1.403597 -0.344184 2.344824
H -1.404450 1.374481 1.930441
16
PMe3PdCl2 SCF Done: -1509.72399417 A.U.
Pd 0.465694 -0.741999 -0.000078
P -0.684541 1.136089 -0.000012
C 0.318588 2.642429 -0.000294
H -0.352262 3.503499 -0.000120
H 0.951998 2.659687 0.883572
H 0.951480 2.659665 -0.884534
C -1.761421 1.251966 -1.451289
H -2.430348 0.394451 -1.467558
H -2.335961 2.178375 -1.388936
H -1.155479 1.256149 -2.355981
C -1.760713 1.252012 1.451789
H -2.335088 2.178555 1.389891
H -2.429799 0.394623 1.468242
H -1.154322 1.255906 2.356182
Cl -1.487065 -1.999934 0.000051
Cl 2.566904 0.219419 0.000053
8
CH3COOH SCF Done: -229.198361583 A.U.
H -1.736060 -0.809982 0.000859
O -0.790216 -1.030289 -0.000123
C -0.089229 0.124347 -0.000118
O -0.624418 1.206811 -0.000021
C 1.389786 -0.125017 -0.000182
H 1.662468 -0.720509 -0.872415
H 1.922986 0.820830 -0.011792
H 1.664336 -0.698489 0.886299
7
CH3COO SCF Done: -228.714509669 A.U.
O 0.709128 1.147272 0.001129
C 0.194662 -0.000013 -0.006117
O 0.804510 -1.098584 0.001183
C -1.348318 -0.045583 -0.002562
H -1.747069 0.647498 -0.746048
H -1.728598 -1.048999 -0.194639
H -1.711501 0.285573 0.974262
13
PMe3 SCF Done: -461.188336333 A.U.
P -0.000012 0.000043 -0.596499
C 0.209971 -1.617222 0.275551
H 0.194099 -1.496407 1.361967
H -0.592124 -2.296973 -0.016643
H 1.159343 -2.069273 -0.016316
C 1.295708 0.990392 0.275586
H 1.211854 2.038805 -0.015302
H 1.200010 0.915214 1.362034
H 2.285376 0.636392 -0.017692

C	-1.505662	0.626762	0.275594
H	-1.393230	0.580396	1.362039
H	-1.693498	1.661170	-0.016817
H	-2.371745	0.030450	-0.016174
